# Vedolizumab in Japanese patients with ulcerative colitis: A Phase 3, randomized, double-blind, placebo-controlled study

**DOI:** 10.1371/journal.pone.0212989

**Published:** 2019-02-26

**Authors:** Satoshi Motoya, Kenji Watanabe, Haruhiko Ogata, Takanori Kanai, Toshiyuki Matsui, Yasuo Suzuki, Mitsuhiro Shikamura, Kenkichi Sugiura, Kazunori Oda, Tetsuharu Hori, Takahiro Araki, Mamoru Watanabe, Toshifumi Hibi

**Affiliations:** 1 IBD Center, Hokkaido Prefectural Welfare Federation of Agricultural Cooperative, Sapporo-Kosei General Hospital, Sapporo, Japan; 2 Department of Intestinal Inflammation Research, Hyogo College of Medicine, Hyogo, Japan; 3 Endoscopic Center, Keio University School of Medicine, Tokyo, Japan; 4 Division of Gastroenterology & Hepatology, Department of Internal Medicine, Keio University School of Medicine, Tokyo, Japan; 5 Department of Gastroenterology, Fukuoka University Chikushi Hospital, Fukuoka, Japan; 6 Department of Internal Medicine, Toho University Medical Center Sakura Hospital, Chiba, Japan; 7 Takeda Development Center Japan, Takeda Pharmaceutical Company, Osaka, Japan; 8 Regenerative Medicine Unit, Takeda Pharmaceutical Company, Kanagawa, Japan; 9 Department of Gastroenterology and Hepatology, Tokyo Medical and Dental University, Tokyo, Japan; 10 Center for Advanced Inflammatory Bowel Disease Research and Treatment, Kitasato University Kitasato Institute Hospital, Tokyo, Japan; University Hospital Llandough, UNITED KINGDOM

## Abstract

**Background:**

Vedolizumab safety and efficacy have been established in many populations all over the world, but have never been studied in Japan. We report results from a Phase 3, randomized, double-blind, placebo-controlled study of vedolizumab in Japanese patients with active ulcerative colitis (UC).

**Methods:**

Patients with moderate-to-severe UC were enrolled into Cohort 1 (double-blinded) or Cohort 2 (open-label) in the induction phase. Cohort 1 was randomized 2:1 to receive 300 mg vedolizumab or placebo, while Cohort 2 received vedolizumab 300 mg only, at Weeks 0, 2, and 6. Patients from Cohorts 1 and 2 showing a clinical response to vedolizumab at Week 10 were randomized 1:1 to receive vedolizumab or placebo (double-blinded) at Week 14 and then every 8 weeks up to Week 54 as the maintenance phase. The primary endpoint was clinical response at Week 10, for the induction phase, and clinical remission at Week 60, for the maintenance phase.

**Results:**

A total of 292 patients were enrolled into the induction phase (246 in Cohort 1, 46 in Cohort 2); 83 patients achieved response to vedolizumab and were subsequently enrolled into the maintenance phase. Clinical response rates at Week 10 were 39.6% (65/164) and 32.9% (27/82) in the vedolizumab and placebo groups in Cohort 1, respectively (adjusted odds ratio [AOR] = 1.37, 95% CI 0.779–2.399; p = 0.2722). In the maintenance phase, clinical remission rate at Week 60 was significantly higher in the vedolizumab group, at 56.1% (23/41), versus 31.0% (13/42) for placebo (AOR = 2.88, 95% CI 1.168–7.108; p = 0.0210). Most adverse events were mild to moderate in intensity, and no deaths occurred during the study period.

**Conclusions:**

Vedolizumab showed numerically greater efficacy compared with placebo as induction therapy, but the difference was not statistically significant. Vedolizumab was significantly superior to placebo as maintenance therapy in Japanese patients with UC. Vedolizumab has favourable safety and tolerability in these patients.

**Trial registration:**

ClinicalTrials.gov: NCT02039505.

## Introduction

Ulcerative colitis (UC) is an inflammatory bowel disease (IBD) with an unpredictable, relapsing/remitting clinical course [1]. Although the prevalence of UC is lower in Japan than in Western countries, it has been steadily increasing [2], with 170,781 patients receiving treatment for UC in Japan in December 2014 [3].

There is currently no treatment which can cure UC; symptoms may have a profound negative impact on the quality of life of the patient [1, 3–6]. Treatment goals with pharmacological therapies are to treat acute and active disease and to prevent relapse when the patient is in remission. More recently, the treatment paradigm for UC has been shifting from resolving symptoms toward objective measures such as mucosal healing [7]. Available treatments for mild to severe UC are aminosalicylates, steroids, immunomodulators and biological therapies such as tumor necrosis factor alpha (TNFα) antagonists [1, 8]. However, these treatments have limitations: 5-aminosalicylic acids (5-ASAs) have moderate efficacy; corticosteroids impose serious side effects and are inappropriate for long-term maintenance; immunomodulators and systemically acting biological drugs such as TNFα antagonists, while effective, have safety concerns such as increased risks of serious infections and malignancies [8–12]. Furthermore, around 10–30% of IBD patients do not respond to the initial anti-TNF treatment, while 23–46% of those who respond lose response over time [13].

Vedolizumab, a new type of biologic, is a humanized monoclonal antibody with a novel mode of action: it blocks lymphocyte infiltration to the gut tissue by selectively binding to α_4_β_7_ integrin without inducing systemic immunosuppression [14]. Vedolizumab has shown efficacy in Phase 3 trials in patients with UC (GEMINI 1) [15] and Crohn’s disease (GEMINI 2 and 3) [16, 17] and is widely approved for the treatment of moderate-to-severe IBD, with extensive real world experience outside Japan. As Japanese patients with UC were not included in GEMINI 1, efficacy and safety of vedolizumab remains to be confirmed in this population.

Differences in IBD susceptibility due to genetic polymorphisms have been reported in different populations [18, 19]. As such, response to IBD treatment may vary in different ethnic populations [20, 21]. A separate trial in the Japanese population was therefore warranted. The pharmacokinetics and tolerability of vedolizumab in Japanese patients with UC were assessed in a Phase 1 study, highlighting a pharmacokinetic and pharmacodynamic profile similar to that observed in non-Japanese patients [22]. However, further investigation was required to determine efficacy and safety profile of vedolizumab in Japanese patients. Furthermore, exploratory analyses of GEMINI 1 suggested that greater efficacy may have been obtained with a longer induction treatment [14]; however, the efficacy of vedolizumab at > 6 weeks’ induction has not been investigated as a primary endpoint. Here we report results from a Phase 3 study evaluating efficacy and safety of vedolizumab as induction and maintenance therapy in Japanese patients with UC.

## Patients and methods

### Study design

This Phase 3 study (ClinicalTrials.gov: NCT02039505) consisted of two sequential and integrated, randomized, double-blind, placebo-controlled, parallel-group, multicenter trials plus an open-label cohort, to investigate the efficacy, safety, and PK of vedolizumab as induction and maintenance therapy in Japanese patients with moderate-to-severe UC. Patients were recruited from 100 centers across all of Japan (see Acknowledgments).

For the induction phase, two cohorts were enrolled. Patients meeting the eligibility criteria were either enrolled into Cohort 1, and then randomized in a 2:1 ratio to receive 300 mg of vedolizumab (intravenous infusion) or placebo at Weeks 0, 2, and 6 in a double-blind fashion; or into Cohort 2, where they received open-label vedolizumab 300 mg at the same time points as Cohort 1 (S1 Fig). Enrollment into Cohort 2 started after the enrollment into Cohort 1 was completed.

After the primary efficacy evaluation at Week 10, all patients from Cohorts 1 and 2 showing clinical response to vedolizumab entered the maintenance phase at Week 14 and were randomized 1:1 to receive vedolizumab 300 mg or placebo at Weeks 14, 22, 30, 38, 46, and 54. The primary efficacy evaluation in the maintenance phase was clinical remission at Week 60.

Patients could switch to the open-label cohort, and receive vedolizumab 300 mg re-induction (at Weeks 0, 2, and 6 from initiation of the open-label cohort) and then every 8 weeks up to 94 weeks, if they: had no clinical response to vedolizumab or placebo at Week 10; experienced disease worsening; received rescue treatment during the maintenance phase; completed Week 60 in the maintenance phase. The end-of-study evaluation was performed in each patient 16 weeks after the last dose of the study drug.

### Randomization and blinding

Randomization schedules were generated by sponsor-designated personnel and kept in a secure area. In the induction phase (Cohort 1 only), dynamic randomization was performed with prior use of TNFα antagonist (yes/no), concomitant immunomodulator use (yes/no), concomitant corticosteroid use (yes/no), and the study sites as stratification factors. In the maintenance phase (Cohort 1 or 2 responders to vedolizumab induction at week 10), dynamic randomization was performed with the previous TNFα antagonist use (yes/no), concomitant immunomodulator use (yes/no), concomitant corticosteroid use (yes/no), the study sites, the cohort in the induction phase (Cohort 1/2), and remission status at Week 10 (remission/non-remission) as stratification factors.

The blinding was maintained in the induction (Cohort 1) and maintenance phases (Cohort 1 and 2 responders to vedolizumab after randomization); only the study site pharmacists were aware of treatment assignments.

### Patients

Eligible patients were aged 15–80 years with total or left-sided UC diagnosis ≥6 months before enrollment into the study, based on the Japanese Diagnostic Criteria for UC (2012 revision) [23]. Patients had moderate-to-severely-active UC, defined as baseline full Mayo [24] score of 6–12 with an endoscopic subscore ≥2. Patients were enrolled in both the inpatient and outpatient settings.

Another eligibility criterion was treatment failure with corticosteroids, immunomodulators (azathioprine [AZA] or 6-mercaptopurine), or TNFα antagonist, within 5 years before informed consent. The number of patients with no previous use of TNFα antagonist was planned to be 50% of the total study population. Patients with a ≥3-point decrease in partial Mayo score between screening and the start of study drug administration; suspected abdominal abscess or toxic megacolon; or history of colectomy, or recent enterectomy; or previously treated with vedolizumab, natalizumab, efalizumab, or rituximab were excluded from the study.

Treatment with 5-ASA enemas/suppositories and corticosteroids enemas/suppositories/intravenous infusion had to be discontinued ≥14 days before the first dose of the study drug. Tacrolimus, cyclosporine, methotrexate, tofacitinib, any low-molecular-weight study drug for UC, and adalimumab had to be discontinued ≥28 days before the study drug initiation. Other biologic agents had to be discontinued ≥56 days before the first dose. Patients were allowed concomitant treatment with oral 5-ASA, probiotics, oral corticosteroids, AZA, and 6-mercaptopurine under specific conditions.

Before patients were recruited, the study protocol was reviewed and approved by an institutional review board of each study site (named in the Acknowledgments). This study was conducted in compliance with all Institutional Review Board regulations and in accordance with the ethical principles that have their origin in the Declaration of Helsinki. All patients provided written informed consent. For patients aged <20 years, written informed consent was additionally obtained from their parents or legal guardians. All authors had access to the study data and reviewed and approved this manuscript.

### Endpoints and assessments

The primary endpoint for the induction phase (Cohort 1 only) was clinical response at Week 10, defined as a reduction of ≥3 points and ≥30% from baseline in the full Mayo score [24], and ≥1 point decrease on the rectal bleeding subscore or an absolute rectal bleeding subscore ≤1 [15]. Secondary endpoints were clinical remission (defined as a full Mayo score ≤2 and no subscore >1) [15], and mucosal healing (defined as an endoscopic subscore ≤1) at Week 10.

In the maintenance phase (Cohort 1 and 2 responders to vedolizumab induction), the primary endpoint was clinical remission at Week 60; secondary endpoints were durable clinical response (defined as a clinical response at both Weeks 10 and 60), mucosal healing, durable remission (defined as a clinical remission at both Weeks 10 and 60), and corticosteroid-free remission at Week 60 (defined as a clinical remission at Weeks 60 without corticosteroid in patients receiving concomitant corticosteroid therapy at Week 0).

The study investigators received training before and during the study to standardize primary endpoint assessment. Endoscopy was performed in the rectum and sigmoid colon ≤9 days before the full Mayo score evaluation (at Week 0, 10, and 60). The investigator identified the most active site at baseline, and scored the identical site for the endoscopy findings throughout the study period. The full Mayo score system has a possible total of 12 points, comprising 4 sub-domains with respective totals of 3 points each. The endoscopy findings were scored by the same physician throughout the study, where possible. Additionally, the Clinical Endpoint Committee (CEC) independently assessed subscores for endoscopy findings for adjudication of endoscopic scores. The CEC’s assessment of each potential endpoint was also used in the endpoint analysis.

PK and immunogenicity endpoints were serum vedolizumab and anti-vedolizumab antibody (AVA), including neutralizing antibody, respectively. Adverse events (AEs) were classified with the use of the Medical Dictionary for Regulatory Activities (MedDRA) v19.0.

Patients were seen at Weeks 0, 2, and every 4 weeks thereafter until Week 58, and at Week 60. The full Mayo score was recorded at Weeks 0, 10, and 60; at all other visits the partial Mayo score was recorded. Blood samples were collected at Weeks 0, 10, 30, and 60 for immunogenicity tests, and at Weeks 2, 6, 10, 14, 22, 30, and 60 for PK analysis.

### Statistical analysis

Analyzes were conducted separately for the induction and maintenance phases, with no adjustments for multiplicity between phases. The full analysis set was defined as all randomized patients who received ≥1 dose of the study drug in the induction phase. Treatment comparisons were done two-sided at the 5% significance level.

Clinical response rate at Week 10 and clinical remission rate at Week 60 were compared between the vedolizumab and placebo treatment arms by using the Cochran-Mantel-Haenszel test, and were adjusted for the stratification factor of previous exposure to TNFα antagonists. The level of significance was set at 5% for both the clinical response rate and the clinical remission rate. Rates of clinical remission and mucosal healing at Week 10, and of durable response, mucosal healing, and corticosteroid-free remission at Week 60, were analyzed in a similar fashion. Clinical response, clinical remission, and mucosal healing were considered as no-response, no-remission, or no-mucosal healing, when adjudication for these endpoints were missing at the time of evaluation. The investigator-assessed Mayo endoscopic subscore was used for primary analysis, while the CEC-assessed Mayo endoscopic subscore was used for the sensitivity analysis.

Clinical response rate at Week 10 was expected to be 57.1% and 35.5% for the vedolizumab and placebo groups, respectively. Clinical response rate at Week 60 was expected to be 43.3% and 15.9%, respectively. A sample size of approximately 246 patients (164 in the vedolizumab group and 82 in the placebo group) was estimated to provide 90% power to detect a statistically significant difference in clinical response at Week 10 between the vedolizumab and placebo groups in the induction phase. The attrition rate was not considered separately in this study, as the expected clinical response rate derived from the GEMINI 1 data (on which the calculation for sample size was based on) considered patients who discontinued treatment (i.e. missing data) as a non-response. For the maintenance phase, it was estimated that a sample size of 84–110 patients would provide 80–90% power to detect a statistically significant difference in clinical response at Week 60 between groups. The Cohort 2 was included in the study design to supplement patient numbers in the maintenance phase, and ensure sufficient power after patient discontinuations in the induction phase. All statistical analyses were performed using SAS v9.4 (SAS Institute Inc., Cary, NC, USA).

## Results

### Patient disposition and baseline characteristics

**Fig 1:**
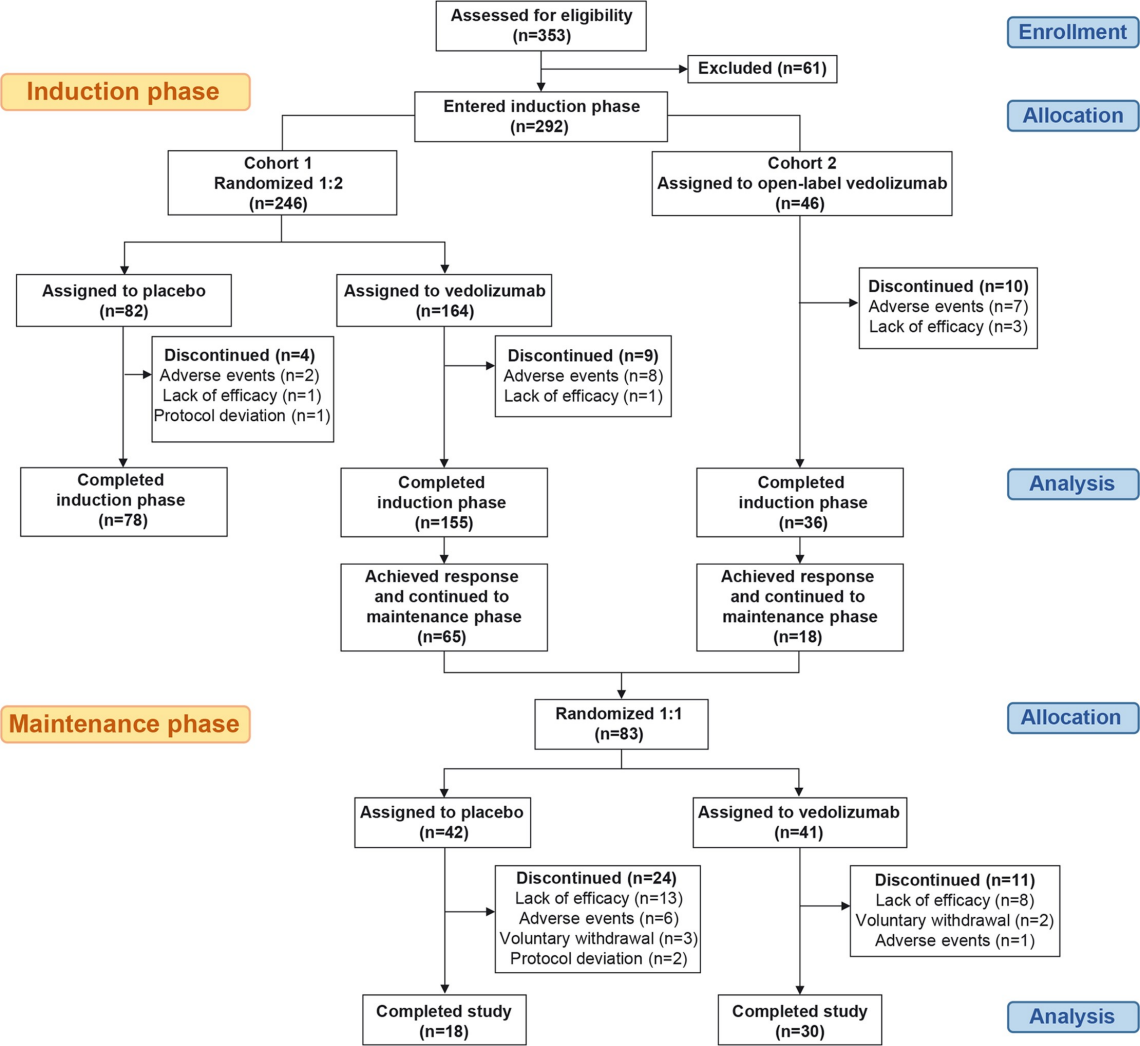
Patient disposition. Baseline characteristics were generally similar across all groups. However, patients with C-reactive protein (CRP) levels ≥3 mg/L were more frequent in the vedolizumab group compared with placebo, especially those with prior anti-TNF exposure, indicating a higher inflammatory status for this patient group (Cohort 1; Table 1).

Of the 353 screened patients, 292 were enrolled into the induction phase across 86 study sites in Japan from February 2014 to November 2015. Of these, 246 patients were assigned to Cohort 1 and 46 to Cohort 2. Patients in Cohort 1 were randomized to receive vedolizumab (n = 164) or placebo (n = 82). Patients in Cohort 2 received open-label vedolizumab. Planned study drug infusions were completed in 155 and 78 patients in the vedolizumab and placebo groups, respectively, in Cohort 1, and in 36 patients in Cohort 2. The main reason for discontinuation in all groups was occurrence of AEs (Fig 1).

**Table 1:** Patient demographics and baseline characteristics (Induction phase).

	Cohort 1		Cohort 2
	Vedolizumab (n = 164)	Placebo (n = 82)	Vedolizumab (n = 46)
Age, y, mean (SD)	42.3 (14.4)	44.0 (16.0)	42.4 (15.6)
Male sex, n (%)	99 (60.4)	55 (67.1)	26 (56.5)
BMI, kg/m^2^, mean (SD)	21.7 (3.4)	21.8 (3.7)	21.1 (2.7)
Duration of UC, y, mean (SD)	7.2 (6.2)	8.6 (8.0)	9.2 (7.7)
Range	0.6–32.7	0.5–53.5	0.7–29.8
Full Mayo score, mean (SD)	8.3 (1.5)	8.1 (1.5)	8.3 (1.7)
Mayo endoscopic subscore = 2, n (%)^a^	110 (67.1)	54 (65.9)	–
In patients with prior use of TNFα antagonist^b^	47 of 85 (55.3)	27 of 41 (65.9)	
Mayo endoscopic subscore = 3, n (%)^a^	54 (32.9)	28 (34.1)	–
In patients with prior use of TNFα antagonist^b^	38 of 85 (44.7)	14 of 41 (34.1)	
Site of disease, n (%)			
Total colitis	101 (61.6)	51 (62.2)	32 (69.6)
Left-sided colitis	63 (38.4)	31 (37.8)	14 (30.4)
Concomitant medication for UC, n (%)			
5-ASA	145 (88.4)	75 (91.5)	39 (84.4)
OC only	31 (18.9)	11 (13.4)	13 (28.3)
Immunomodulators only	59 (36.0)	29 (35.4)	17 (37.0)
OC and immunomodulators	21 (12.8)	14 (17.1)	6 (13.0)
No OC or immunomodulators	53 (32.2)	28 (34.1)	10 (21.7)
Prior TNFα antagonist therapy, n (%)	85 (51.8)	41 (50.0)	24 (52.2)
Prior failure of TNFα antagonist, n (%)	84 (51.2)	41 (50.0)	23 (50.0)
Inadequate response^c^	50 of 84 (59.5)	22 of 41 (53.7)	10 of 23 (43.5)
Loss of response^c^	32 of 84 (38.1)	18 of 41 (43.9)	12 of 23 (52.2)
Intolerance^c^	2 of 84 (2.4)	1 of 41 (2.4)	1 of 23 (4.3)
Prior failure of corticosteroid therapy, n (%)	109 (66.5)	57 (69.5)	34 (73.9)
Prior failure of immunomodulator therapy, n (%)	99 (60.4)	52 (63.4)	31 (67.4)
Extraintestinal manifestations, n (%)	53 (32.3)	16 (19.5)	14 (30.4)
C-reactive protein level <3 mg/L, n (%)	76 (46.3)	50 (61.0)	–
In patients with prior use of TNFα antagonist^b^	33 of 85 (38.8)	25 of 41 (61.0)	
C-reactive protein level ≥3 mg/L, n (%)	88 (53.7)	32 (39.0)	–
In patients with prior use of TNFα antagonist^b^	52 of 85 (61.2)	16 of 41 (39.0)	

^a^Mayo endoscopic subscore 2 = moderate disease (marked erythema, lack of vascular pattern, friability, erosions); 3 = severe disease (spontaneous bleeding, ulceration).

^b^Percentage calculated based on patients with prior use of TNFα antagonist: n = 85 for vedolizumab and n = 41 for placebo

^c^Percentage calculated based on patients with prior failure of TNFα antagonist: n = 84 for vedolizumab (cohort 1), n = 41 for placebo, and n = 23 for vedolizumab (cohort 2).

5-ASA, 5-aminosalicylic acid; BMI, body mass index; OC, oral corticosteroids; SD, standard deviation; TNFα, tumor necrosis factor alpha; UC, ulcerative colitis.

Of the 65 and 18 (83 in total) patients in Cohorts 1 and 2, respectively, with a clinical response to vedolizumab at Week 10, 41 and 42 patients were randomized to receive vedolizumab or placebo in the maintenance phase, respectively. There were 11 discontinuations in the vedolizumab group and 24 in the placebo group; the main reason in both groups was lack of efficacy (Fig 1).

Baseline characteristics for patients enrolled into the maintenance phase were similar to those for patients in the induction phase (S1 Table).

### Efficacy

#### Outcomes in the induction phase

In Cohort 1, clinical response rates at Week 10 were 39.6% and 32.9% in the vedolizumab and placebo groups, respectively (Table 2). The response rate in the vedolizumab group was numerically higher than in the placebo group, but the difference was not statistically significant (adjusted odds ratio [AOR] = 1.37, 95% confidence interval [CI] 0.779–2.399; p = 0.2722). In patients without prior use of TNFα antagonist, clinical response rate at Week 10 was numerically higher in the vedolizumab group, at 53.2%, compared with placebo, at 36.6% (Table 2). However, in patients with prior use of TNFα antagonist, response rates were comparable between the vedolizumab and placebo groups.

**Table 2:** Efficacy outcomes at weeks 10 and 60 (Induction and maintenance phases).

	Vedolizumab, n (%)	Placebo, n (%)	Difference: vedolizumab-placebo, % (95% CI)	AOR (95% CI)	p-value
**Induction phase (at Week 10)**	**n = 164**	**n = 82**			
Clinical response	65 (39.6)	27 (32.9)	6.7 (-5.922–19.337)	1.37 (0.779–2.399)	0.2722
Clinical remission	30 (18.3)	10 (12.2)	6.1 (-3.131–15.326)	1.66 (0.762–3.596)	0.1980
Mucosal healing	60 (36.6)	25 (30.5)	6.1 (-6.297–18.492)	1.33 (0.755–2.356)	0.3168
*In patients without prior use of TNFα antagonist*	n = 79	n = 41			
Clinical response	42 (53.2)	15 (36.6)	16.6 (-1.818–34.976)	–	–
Clinical remission	22 (27.8)	6 (14.6)	13.2 (-1.440–27.868)	–	–
Mucosal healing	38 (48.1)	13 (31.7)	16.4 (-1.614–34.402)	–	–
*In patients with prior use of TNFα antagonist*	n = 85	n = 41			
Clinical response	23 (27.1)	12 (29.3)	-2.2 (-19.037–14.618)	–	–
Clinical remission	8 (9.4)	4 (9.8)	-0.3 (-11.345–10.657)	–	–
Mucosal healing	22 (25.9)	12 (29.3)	-3.4 (-20.139–13.367)	–	–
**Maintenance phase (at Week 60)**	**n = 41**	**n = 42**			
Clinical remission	23 (56.1)	13 (31.0)	25.1 (4.500–45.790)	2.88 (1.168–7.108)	0.021
Durable response	27 (65.9)	15 (35.7)	30.1 (9.629–50.650)	3.48 (1.407–8.626)	0.0067
Mucosal healing	26 (63.4)	14 (33.3)	30.1 (9.572–50.590)	3.49 (1.409–8.642)	0.0066
Durable remission	11 (26.8)	7 (16.7)	10.2 (-7.472–27.797)	2.02 (0.677–6.033)	0.209
Corticosteroid-free remission^a^	6 (46.2)	3 (20.0)	26.2 (-7.671–59.979)	3.38 (0.636–17.981)	0.1571
*In patients without prior use of TNFα antagonist*	n = 24	n = 28			
Clinical remission	13 (54.2)	10 (35.7)	18.5 (-8.238–45.142)	–	–
Durable response	16 (66.7)	10 (35.7)	31.0 (5.055–56.850)	–	–
Mucosal healing	15 (62.5)	10 (35.7)	26.8 (0.515–53.056)	–	–
Durable remission	8 (33.3)	6 (21.4)	11.9 (-12.317–36.126)	–	–
Corticosteroid-free remission^b^	4 (44.4)	2 (22.2)	22.2 (-20.105–64.550)	–	–
*In patients with prior use of TNFα antagonist*	n = 17	n = 14			
Clinical remission	10 (58.8)	3 (21.4)	37.4 (5.625–69.165)	–	–
Durable response	11 (64.7)	5 (35.7)	29.0 (-4.861–62.845)	–	–
Mucosal healing	11 (64.7)	4 (28.6)	36.1 (3.332–68.937)	–	–
Durable remission	3 (17.6)	1 (7.1)	10.5 (-12.088–33.096)	–	–
Corticosteroid-free remission^c^	2 (50.0)	1 (16.7)	33.3 (-24.026–90.693)	–	–

^a^In patients receiving concomitant corticosteroid therapy at Week 0, and who had clinical response at Week 10, after which the dose reduction of corticosteroid was initiated. Vedolizumab, n = 13; placebo, n = 15.

^b^Vedolizumab, n = 9; placebo, n = 9.

^c^Vedolizumab, n = 4; placebo, n = 6.

AOR, adjusted odds ratio; CI, confidence interval; TNFα, tumor necrosis factor alpha.

Cochran-Mantel-Haenszel estimates and test with stratification according to prior TNFα antagonist use (yes or no)

Clinical remission at Week 10 was observed in 18.3% and 12.2% of patients receiving vedolizumab or placebo, respectively; the difference of 6.1% was not statistically significant (AOR, 1.66; 95% CI, 0.762–3.596; p = 0.1980) (Table 2). Mucosal healing rate at Week 10 was numerically higher in the vedolizumab (36.6%) versus placebo (30.5%) group, but the difference was not statistically significant (AOR, 1.33; 95% CI, 0.755–2.356; p = 0.3168) (Table 2). Stratification by prior use TNFα antagonist for these endpoints showed similar trends to those observed for clinical response rate at Week 10 (Table 2).

The results of post hoc, sub-group analysis of clinical response rate at Week 10 in patients with prior TNFα antagonist use, stratified by MES and CRP level, are shown in S2 Table. Higher clinical response rates for placebo were observed in patients with less active disease (40.7%, MES = 2; 36.0%, CRP level <3 mg/L). In patients with more active disease, vedolizumab efficacy was evident owing to lower clinical placebo response rates in the placebo group (21.1% vs 7.1% for MES = 3; 28.8% vs 18.8% for CRP level ≥3 mg/L, respectively). Values based on assessment by the CEC at Week 10 were similar to those based on investigators' assessment: clinical response rates were 39.6% (65/164) and 30.5% (25/82) for vedolizumab and placebo, respectively (p = 0.1379); clinical remission rates were 18.9% (31/164) and 12.2% (10/82) (p = 0.1607); and mucosal healing rates were 35.4% (58/164) and 35.4% (29/82), respectively (p = 0.9569).

Rates of clinical response, clinical remission, and mucosal healing at Week 10 in Cohort 2 were 41.3% (19/46), 15.2% (7/46) and 28.3% (13/46), respectively.

#### Outcomes in the maintenance phase

In responders to vedolizumab induction therapy (from both Cohorts 1 and 2), clinical remission rate at Week 60 was significantly higher in the vedolizumab group (56.1%) versus the placebo group (31.0%) (AOR = 2.88, 95% CI 1.168–7.108; p = 0.0210) (Table 2). Durable clinical response rate was also significantly higher in the vedolizumab (65.9%) versus placebo (35.7%) groups (AOR, 3.48; 95% CI, 1.407–8.626; p = 0.0067). Likewise, mucosal healing rate in the vedolizumab group (63.4%) was significantly higher than in the placebo group (33.3%) (AOR, 3.49; 95% CI, 1.409–8.642; p = 0.0066). Durable remission rate was numerically higher in the vedolizumab group (26.8%), though not statistically significantly compared with placebo (16.7%) (AOR, 2.02; 95% CI, 0.677–6.033; p = 0.2090). Corticosteroid-free remission rate at Week 60 was also higher in patients receiving vedolizumab, at 46.2%, compared with placebo, at 20.0%, but the difference was not statistically significant (AOR, 3.38; 95% CI, 0.636–17.981; p = 0.1571). In addition, the rates of all the endpoints analyzed in the maintenance phase were numerically higher in the vedolizumab group than the placebo group regardless of prior TNFα antagonist use (Table 2).

Results for the maintenance phase based on assessment by the CEC were similar to those based on investigators' assessment: clinical remission rates were 51.2% (21/41) and 31.0% (13/42) for vedolizumab and placebo, respectively (p = 0.0644); durable clinical response rates were 63.4% (26/41) and 35.7% (15/42) (p = 0.0119); mucosal healing rates were 61.0% (25/41) and 35.7% (15/42) (p = 0.3245); durable remission rates were 24.4% (10/41) and 16.7% (7/42) (p = 0.3245), and corticosteroid-free remission rates were 46.2% (6/13) and 20.0% (3/15), respectively (p = 0.1571).

### Pharmacokinetics and immunogenicity

The mean (standard deviation [SD]) vedolizumab concentrations at Week 10 were 31.4 (14.5) μg/mL in patients in Cohort 1 receiving vedolizumab, and 36.6 (16.1) μg/mL in Cohort 2. At steady state (Week 30), the mean (SD) trough vedolizumab concentration was 13.8 (6.4) μg/mL. The mean vedolizumab concentration was 21.2 (8.9) μg/mL at Week 60 in patients receiving vedolizumab.

Of the 167 patients who received continuous doses of vedolizumab in the study and with available blood samples, 5 (3.0%) were AVA-positive at any time in the induction or maintenance phases, or in the open-label cohort. All 5 AVA-positive patients were transiently positive (i.e., at least one positive sample but no consecutive positive samples). One of the 5 AVA-positive patients was also positive for neutralizing antibodies.

### Safety

Planned infusions in the induction phase were completed by 95.7% (157 of 162 patients) and 95.1% (78/82) of patients in the vedolizumab and placebo groups in Cohort 1, respectively, and by 84.8% (39/46) of patients in Cohort 2. In the maintenance phase, 70.7% (29/41) and 45.2% (19/42) of patients in the vedolizumab and placebo groups, respectively, completed planned infusions.

No substantial differences in AEs were observed between the treatment groups; the incidence of AEs in the induction phase was similar in the vedolizumab and placebo groups at 50.0% and 52.4%, respectively, in Cohort 1, and 71.7% in Cohort 2 (Table 3). The most frequent AE was nasopharyngitis (Table 4). Drug-related AEs occurred in 10.4% of patients in the vedolizumab group, 14.6% of patients in the placebo group (Cohort 1), and 15.2% of patients receiving vedolizumab in Cohort 2. Most AEs were mild to moderate and no deaths occurred. Serious AEs were reported in 6.1%, 4.9%, and 13.0% of patients in the vedolizumab and placebo groups (Cohort 1) and Cohort 2, respectively (Table 3); drug-related AEs occurred in 0.6%, 2.4%, and 2.2% of patients, and 4.3%, 1.2%, and 8.7%, resulted in study drug discontinuations. Serious AEs of infections/infestations were low in the vedolizumab group compared with the placebo group (0.6% vs 2.4% [Cohort 1]). Infusion reactions occurred in 3.0% in the vedolizumab group and in 2.4% of patients in the placebo group (Cohort 1). One patient in the vedolizumab group in Cohort 1 was positive for ‘visual field disorder’ on the subjective progressive multifocal leukoencephalopathy (PML) checklist at Week 6, but no findings were reported for the objective PML checklist. No cases of PML were reported in the induction phase.

**Table 3:** Overview of adverse events (Safety analysis set).

	Induction phase			Maintenance phase^a^	
	Cohort 1		Cohort 2		
	Vedolizumab (n = 164)	Placebo (n = 82)	Vedolizumab (n = 46)	Vedolizumab (n = 41)	Placebo (n = 42)
Overall AEs, n (%)	82 (50.0)	43 (52.4)	33 (71.7)	36 (87.8)	33 (78.6)
Related to study drug	17 (10.4)	12 (14.6)	7 (15.2)	4 (9.8)	6 (14.3)
Leading to study drug discontinuation	8 (4.9)	2 (2.4)	6 (13.0)	2 (4.9)	6 (14.3)
Overall serious AEs, n (%)	10 (6.1)	4 (4.9)	6 (13.0)	4 (9.8)	3 (7.1)
Related to study drug	1 (0.6)	2 (2.4)	1 (2.2)	1 (2.4)	0 (0.0)
Leading to study drug discontinuation	7 (4.3)	1 (1.2)	4 (8.7)	1 (2.4)	2 (4.8)
Deaths, n (%)	0 (0.0)	0 (0.0)	0 (0.0)	0 (0.0)	0 (0.0)

^a^Patients randomized and received ≥1 dose in maintenance phase only. AEs, adverse events.

**Table 4:** Adverse events by preferred term with an incidence of ≥5% in any treatment group (Safety analysis set).

	Induction phase			Maintenance phase^a^	
	Cohort 1		Cohort 2		
Adverse event by preferred term, n (%)	Vedolizumab (n = 164)	Placebo (n = 82)	Vedolizumab (n = 46)	Vedolizumab (n = 41)	Placebo (n = 42)
Nasopharyngitis	23 (14.0)	9 (11.0)	12 (26.1)	18 (43.9)	9 (21.4)
Ulcerative colitis	7 (4.3)	3 (3.7)	5 (10.9)	2 (4.9)	6 (14.3)
Dental caries	4 (2.4)	–	1 (2.2)	4 (9.8)	–
Arthralgia	3 (1.8)	1 (1.2)	1 (2.2)	4 (9.8)	–
Hemorrhoids	1 (0.6)	1 (1.2)	–	–	–
Vomiting	1 (0.6)	2 (2.4)	1 (2.2)	3 (7.3)	1 (2.4)
Upper respiratory tract inflammation	5 (3.0)	4 (4.9)	2 (4.3)	3 (7.3)	1 (2.4)
Cough	4 (2.4)	2 (2.4)	–	3 (7.3)	–
Eczema	1 (0.6)	1 (1.2)	–	–	3 (7.1)
Headache	6 (3.7)	2 (2.4)	3 (6.5)	2 (4.9)	1 (2.4)
Nausea	2 (1.2)	–	3 (6.5)	–	–
Gastroenteritis	–	–	3 (6.5)	2 (4.9)	2 (4.8)

^a^Randomized patients only.

In the maintenance phase, 87.8% and 78.6% of patients in the vedolizumab and placebo groups, respectively, experienced AEs (Table 3). The most frequent AE was nasopharyngitis, reported in 43.9% and 21.4% of patients in the vedolizumab and placebo groups, respectively (Table 4). Most AEs were mild to moderate in intensity and no deaths occurred. Drug-related AEs were observed in 9.8% of patients in the vedolizumab group, versus 14.3% in the placebo group (Table 3). Rates of serious AEs were 9.8% and 7.1% in the vedolizumab and placebo groups, respectively, with only one drug-related case in the vedolizumab group (pyrexia, myalgia and arthralgia). One (2.4%) patient in the vedolizumab group and two (4.8%) patients in the placebo group had serious AEs leading to study drug discontinuation. Serious AEs of infections/infestations occurred in 2.4% of patients in each treatment group. No infusion reactions were reported in either treatment group. No cases of PML or positive findings on the PML checklists were reported in the maintenance phase.

## Discussion

This is the first randomized, placebo-controlled, Phase 3 study of vedolizumab in Japanese patients with moderate-to-severely-active UC. The study design was similar to GEMINI 1, a global Phase 3 study of vedolizumab, conducted in 34 countries. In GEMINI 1, efficacy endpoints for the induction phase were assessed at Week 6; however, exploratory analyses suggested that greater efficacy may have been obtained with a longer induction treatment [15]. Therefore, the present study was designed to assess the efficacy of induction therapy at Week 10.

In GEMINI 1, clinical response rates at Week 6 were 47.1% for vedolizumab and 25.5% for placebo; this difference was statistically significant [15]. In the present study, clinical response rates at Week 10 were 39.6% for vedolizumab and 32.9% for placebo; the difference between treatment groups, while showing a higher numerical rate for vedolizumab, was not statistically significant.

In patients without prior use of TNFα antagonist, clinical response was higher in the vedolizumab group compared with the placebo group at comparable rates in both studies (53.1% vs 26.3% at Week 6 in GEMINI 1 [15], and 53.2% vs 36.6% at Week 10 in the present study). In patients with prior use of TNFα antagonist, the clinical response rate at Week 6 was significantly higher in the vedolizumab group compared with placebo in GEMINI 1 (39.0% vs 20.6%) [25]; however, the clinical response rates in both treatment groups at Week 10 were comparable in the present study (27.1% vs 29.3%). The reason for the higher placebo rates in this study has not been identified, but it may be due to an imbalance in the baseline disease activity between the treatment groups especially in patients with prior use of TNFα antagonist. A higher proportion of patients in the placebo group had a Mayo endoscopic subscore (MES) of 2 (65.9%), compared with the vedolizumab group (55.3%). Similarly, more patients in the placebo group had levels of CRP, an inflammatory biomarker for UC [26, 27], of <3 mg/L compared with the vedolizumab group (61.0% vs 38.8%). Sub-group analysis of clinical response rate at Week 10 in patients with prior TNFα antagonist use, stratified by MES and CRP level, indicated higher clinical response rates for placebo in patients with less active disease (S2 Table). In patients with more active disease, vedolizumab efficacy was evident owing to lower clinical placebo response rates in the placebo.

A meta-analysis supports this hypothesis, in which trials enrolling patients with endoscopically less-active disease were associated with significantly higher response rates in the placebo group [28]. As stratified randomized allocation for MES or CRP levels at baseline was not conducted in the present study, such imbalance of baseline disease activity occurred by coincidence. Also, the proportion of patients with MES = 3 was lower in the present study (~30%; Table 1) compared with GEMINI 1 (~50%; unpublished data), which may have contributed to the discrepancy observed between the studies.

Additionally, higher rate of clinical response was observed at Week 10 in the placebo group among patients concomitantly taking corticosteroids (44.0%, 11/25) versus not (28.1%, 16/57). This observation is consistent with the short-term effects of corticosteroids and to what has been observed in other studies including Japanese UC patients [29]. This phenomenon was not noted for clinical remission at week 60, which is also consistent with the lack of long-term efficacy of corticosteroids [30].

Standard treatments for UC are not substantially different in and outside Japan, but a few exceptions exist. Leukocytapheresis, the therapeutic removal of peripheral leukocytes from blood, is an effective [21], approved treatment in Japan [31], but not in Europe or North America [20]. Tacrolimus, a calcineurin inhibitor, is approved for patients with severe or refractory UC in Japan and is recommended by Japanese guidelines [32]. In this study, 15 patients received prior leukocytapheresis (1 vedolizumab patient) or tacrolimus (9 vedolizumab and 5 placebo patients) and thus the numbers were insufficient to assess potential impact on the results; these data were collected from the time of, or 4 weeks before informed consent. Nevertheless, the study results were not determined to have been affected by prior use of leukocytapheresis or tacrolimus because treatment histories were unclear prior to the data collection.

Further investigation is required to determine the efficacy of vedolizumab as induction therapy in Japanese patients, particularly in those with prior use of TNFα antagonists.

In the maintenance phase, rates of clinical remission, durable response and mucosal healing at Week 60 were 56.1%, 65.9% and 63.4% in patients receiving vedolizumab, respectively, and 31.0%, 35.7% and 33.3% in those receiving placebo; the differences between vedolizumab and placebo were statistically significant, consistent with data reported for the GEMINI 1 study [15]. Although the vedolizumab-placebo differences in the rates of durable remission and corticosteroid-free remission were not statistically significant, the rates observed for patients treated with vedolizumab were higher than those of patients receiving placebo. These results are encouraging, as a critical objective of therapy in UC includes maintenance of remission, which improves patients’ quality of life and work productivity, and lowers the risk of colon cancer [33]. Our study showed that in addition to a superior clinical remission rate, vedolizumab treatment resulted in a significantly higher mucosal healing rate, which has been associated with a lower rate of disease relapse, a lower risk of disease progression, including dysplasia and colorectal cancer [34].

The Mayo endoscopic subscore results assessed by the investigators (primary analysis) and the CEC (sensitivity analysis) were similar; the primary analysis results were considered valid and reliable.

Vedolizumab therapy in Japanese patients was well tolerated, as most AEs were mild to moderate in intensity; AEs were comparable to previous studies [15, 22, 35]. Additionally, no clinically significant differences were reported in the vedolizumab versus placebo group. Rates of serious AEs were slightly higher in the vedolizumab group in the maintenance phase, but those related to study drug and leading to drug discontinuation were higher in the placebo group. The most frequent AE in both phases was nasopharyngitis, which occurred with a higher rate in the vedolizumab group in the maintenance phase. Importantly, no cases of PML—a known toxicity with nonspecific α_4_ integrin inhibition [36, 37]—were reported in this study. This adds to the body of evidence that vedolizumab does not cause PML, as no cases have been reported so far in vedolizumab-treated patients in clinical trials [15, 22, 35]. PK and immunogenicity results were as expected, and consistent with previous studies in non-Japanese patients [15] and the Phase 1 study in Japanese patients [22].

Vedolizumab represents a new and effective treatment option for Japanese patients with moderate-to-severely-active UC. The gut-selective mechanism of action of vedolizumab supports long-term clinical response, clinical remission and mucosal healing for up to 60 weeks without the safety consequences of a systemic immunosuppression. Results from this study are consistent with previously reported clinical study (GEMINI 1) and real-world vedolizumab experience from patient cohorts outside Japan.

A limitation of this study is the small number of patients per treatment subgroup after stratification for prior TNFα antagonist use. Furthermore, the study was not powered to detect statistically significant differences between treatment subgroups. Consequently, the results of the subgroup analysis should be interpreted with caution.

In conclusion, vedolizumab showed numerically greater efficacy compared with placebo as induction therapy and was significantly superior to placebo as maintenance therapy in Japanese patients with UC. Vedolizumab is safe and well tolerated in these patients, and constitutes a new treatment option for moderate-to-severely-active UC in Japan.

## Supporting information

S1 Fig Study design. Footnotes: ^a^The blindness was maintained until after completion of the database lock at Week 60. ^b^Randomization schedules were generated by sponsor-designated personnel and kept in a secure area. ^c^Dynamic randomization was performed with the previous TNFα antagonist use, concomitant immunomodulator use, concomitant corticosteroid use, and study site as stratification factors. ^d^Dynamic randomization was performed with the previous TNFα antagonist use, concomitant immunomodulator use, concomitant corticosteroid use, study site, Cohort in the induction phase, and remission status at Week 10 as stratification factors. Enrollment into the Open-label cohort was defined as Week 0x.
(TIF)

S1 Table Patient demographics and baseline characteristics (Patients who entered the maintenance phase). (DOCX)

S2 Table Subgroup analysis of clinical response in the induction phase (at Week 10). (DOCX)

S1 File CONSORT checklist. (DOCX)

S2 File Original study protocol (Japanese). (PDF)

S3 File Translated study protocol (English). (PDF)
