# Correction: Vedolizumab in Japanese patients with ulcerative colitis: A Phase 3, randomized, double-blind, placebo-controlled study

**DOI:** 10.1371/journal.pone.0215491

**Published:** 2019-04-11

**Authors:** Satoshi Motoya, Kenji Watanabe, Haruhiko Ogata, Takanori Kanai, Toshiyuki Matsui, Yasuo Suzuki, Mitsuhiro Shikamura, Kenkichi Sugiura, Kazunori Oda, Tetsuharu Hori, Takahiro Araki, Mamoru Watanabe, Toshifumi Hibi

## Notice of Republication

An incorrect version of S2 File was published in error. This article was republished on April 2, 2019 to correct for this error. Please download this article again to view the correct version.
