# Supplementary figures and images for: Vedolizumab in Japanese patients with ulcerative colitis: A Phase 3, randomized, double-blind, placebo-controlled study
Source: PLoS One. 2019 Feb 26;14(2):e0212989. doi: 10.1371/journal.pone.0212989 (PMC6391030; doi:10.1371/journal.pone.0212989)

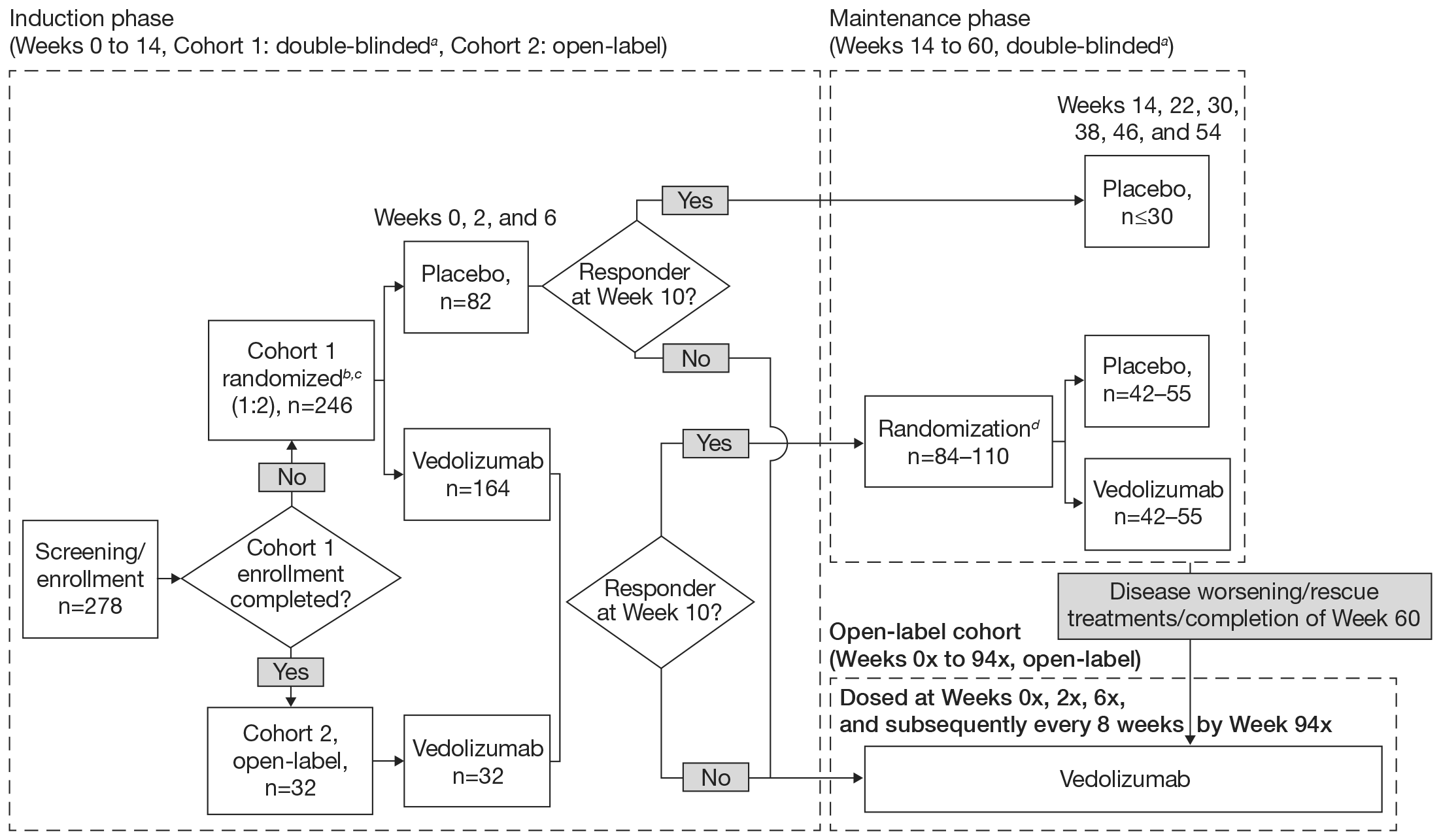

Supplement: S1 Fig — Footnotes: aThe blindness was maintained until after completion of the database lock at Week 60. bRandomization schedules were generated by sponsor-designated personnel and kept in a secure area. cDynamic randomization was performed with the previous TNFα antagonist use, concomitant immunomodulator use, concomitant corticosteroid use, and study site as stratification factors. dDynamic randomization was performed with the previous TNFα antagonist use, concomitant immunomodulator use, concomitant corticosteroid use, study site, Cohort in the induction phase, and remission status at Week 10 as stratification factors. Enrollment into the Open-label cohort was defined as Week 0x. (TIF) [file pone.0212989.s001.tif]
