# Supplementary material for: Vedolizumab in Japanese patients with ulcerative colitis: A Phase 3, randomized, double-blind, placebo-controlled study
Source: PLoS One. 2019 Feb 26;14(2):e0212989. doi: 10.1371/journal.pone.0212989 (PMC6391030; doi:10.1371/journal.pone.0212989)
Supplement: S1 Table — (DOCX) [file pone.0212989.s002.docx]

## S1 Table. Patient demographics and baseline characteristics (patients who entered the maintenance phase).

|  | **Vedolizumab**  **(n = 41)** | **Placebo**  **(n = 42)** |
| --- | --- | --- |
| Age, y, mean (SD) | 43.0 (14.3) | 42.6 (14.4) |
| Male sex, n (%) | 21 (51.2) | 23 (54.8) |
| BMI, kg/m^2^, mean (SD) | 21.7 (3.2) | 21.4 (2.6) |
| Duration of UC, y, mean (SD)  Range | 8.6 (7.8)  0.8–32.7 | 8.7 (7.0)  0.7–29.6 |
| Full Mayo score at Week 0, mean (SD) | 8.1 (1.6) | 7.9 (1.6) |
| Site of disease, n (%)  Total colitis  Left-sided colitis | 28 (68.3)  13 (31.7) | 23 (54.8)  19 (45.2) |
| Concomitant medication for UC at Week 0, n (%)  5-ASA  OC only  Immunomodulators only  OC and immunomodulators  No OC or immunomodulators | 39 (95.1)  9 (22.0)  18 (43.9)  4 (9.8)  10 (24.4) | 36 (85.7)  11 (26.2)  17 (40.5)  4 (9.5)  10 (23.8) |
| Concomitant OC at Week 10, n (%) | 13 (31.7) | 15 (35.7) |
| Prior TNFα antagonist, n (%) | 17 (41.5) | 14 (33.3) |
| Prior failure of TNFα antagonist, n (%)  Inadequate response^a^  Loss of response^a^  Intolerance^a^ | 16 (39.0)  6 of 16 (37.5)  8 of 16 (50.0)  2 of 16 (12.5) | 14 (33.3)  7 of 14 (50.0)  7 of 14 (50.0)  0 of 14 (0.0) |
| Prior failure of corticosteroid therapy, n (%) | 30 (73.2) | 26 (61.9) |
| Prior failure of immunomodulator therapy, n (%) | 22 (53.7) | 28 (66.7) |

5-ASA, 5-aminosalicylic acid; BMI, body mass index; OC, oral corticosteroids; SD, standard deviation; TNFα, tumor necrosis factor alpha; UC, ulcerative colitis.

^a^Percentage calculated based on prior failure of TNFα antagonist: n=16 for vedolizumab and n=14 for placebo. The breakdown of the reasons for treatment failure was calculated using the number of patients with each of the treatment failures as the denominator.
