# Supplementary material for: Vedolizumab in Japanese patients with ulcerative colitis: A Phase 3, randomized, double-blind, placebo-controlled study
Source: PLoS One. 2019 Feb 26;14(2):e0212989. doi: 10.1371/journal.pone.0212989 (PMC6391030; doi:10.1371/journal.pone.0212989)
Supplement: S2 Table — (DOCX) [file pone.0212989.s003.docx]

## S2 Table. Subgroup analysis of clinical response in the induction phase (at Week 10).

|  | **Vedolizumab** | | **Placebo** | | **p-value^a^** |
| --- | --- | --- | --- | --- | --- |
|  | n | Clinical response, n (%) | n | Clinical response, n (%) |  |
| All patients | 164 | 65 (39.6) | 82 | 27 (32.9) | 0.2722 |
| *In patients without prior use of TNFα antagonist* | 79 | 42 (53.2) | 41 | 15 (36.6) | – |
| Mayo Endoscopic Subscore  2  3 | 63  16 | 35 (55.6)  7 (43.8) | 27  14 | 11 (40.7)  4 (28.6) | –  – |
| CRP at baseline  <3 mg/L  ≥3 mg/L | 43  36 | 25 (58.1)  17 (47.2) | 25  16 | 8 (32.0)  7 (43.8) | –  – |
| *In patients with prior use of TNFα antagonist* | 85 | 23 (27.1) | 41 | 12 (29.3) | – |
| Mayo Endoscopic Subscore  2  3 | 47  38 | 15 (31.9)  8 (21.1) | 27  14 | 11 (40.7)  1 (7.1) | –  – |
| CRP at baseline  <3 mg/L  ≥3 mg/L | 33  52 | 8 (24.2)  15 (28.8) | 25  16 | 9 (36.0)  3 (18.8) | –  – |

^a^Cochran-Mantel-Haenszel estimates and test with stratification according to prior TNFα antagonist use (yes or no)
