# Supplementary material for: Vedolizumab in Japanese patients with ulcerative colitis: A Phase 3, randomized, double-blind, placebo-controlled study
Source: PLoS One. 2019 Feb 26;14(2):e0212989. doi: 10.1371/journal.pone.0212989 (PMC6391030; doi:10.1371/journal.pone.0212989)
Supplement: S2 File — (PDF) [file pone.0212989.s005.pdf]

治験実施計画書

中等症又は重症の日本人潰瘍性大腸炎患者の導入療法及び維持療法における、MLN0002（300 mg）を点滴静注したときの有効性、安全性及び薬物動態を検討する、プラセボ対照の第 3 相多施設共同無作為化二重盲検並行群間比較試験

潰瘍性大腸炎の治療における、MLN0002（300 mg）の第 3 相試験

**治験依頼者**	武田薬品工業株式会社<br>大阪府大阪市中央区道修町四丁目 1 番 1 号		
**治験実施計画書番号**	MLN0002/CCT-101		
**版数**	初版		
**IND 番号**	該当せず	**EudraCT 番号**	該当せず
**治験薬**	Vedolizumab		
**作成日**	2013 年 11 月 11 日		

1.0 治験管理情報及び治験の原則

1.1 連絡先及び治験関連業務の責任

別紙参照

1.2 治験の原則

本治験は、本治験実施計画書及び以下の要件を遵守し、参加者個人に配慮して行うものとする。

- ヘルシンキ宣言に基づく倫理的原則
- 医薬品の臨床試験の実施の基準に関する ICH ガイドライン E6
- 適用されるすべての法令及び規則（データ機密保持に関する法令、臨床試験の情報公開に関する法令及び規則を含む）

目次

1.0 治験管理情報及び治験の原則 2
1.1 連絡先及び治験関連業務の責任 2
1.2 治験の原則 3
2.0 要約 10
3.0 略語 15
3.1 本治験における用語の定義 16
4.0 はじめに 17
4.1 背景 17
4.2 臨床試験成績 17
4.2.1 海外第 1 相及び第 2 相試験成績の概要 18
4.2.2 海外第 3 相試験成績の概要 18
4.2.3 国内第 1 相試験成績の概要 19
4.3 本治験を計画した根拠 19
5.0 治験の目的及び評価項目 21
5.1 目的 21
5.1.1 導入期の主目的 21
5.1.2 導入期の副目的 21
5.1.3 維持期の主目的 21
5.1.4 維持期の副目的 21
5.2 評価項目 21
5.2.1 導入期の有効性評価項目 21
5.2.2 維持期の有効性評価項目 22
5.2.3 薬物動態の評価項目 22
5.2.4 安全性評価項目 23
5.2.5 免疫原性の評価項目 23
6.0 治験のデザイン 24
6.1 治験デザイン 24
6.2 治験デザイン、投与量、評価項目の設定根拠 26
6.2.1 治験対象集団の設定根拠 26
6.2.2 デザイン及び計画被験者数の設定根拠 27

6.2.3 プラセボ対照群の設定根拠 27
6.2.4 用法・用量及び評価期間の設定根拠 28
6.2.5 評価項目の設定根拠 29
6.3 治験全体の中止又は治験実施医療機関での治験中止 29
6.3.1 治験全体の中止基準 29
6.3.2 治験実施医療機関での治験中止の基準 29
6.3.3 治験全体又は治験実施医療機関での治験中断及び中止に関する手順 29
7.0 被験者の選定及び中止の基準 30
7.1 選択基準 30
7.2 除外基準 31
7.3 併用禁止薬及び併用禁止治療 34
7.3.1 救済治療 35
7.3.2 ステロイドの漸減投与計画 35
7.4 被験者の管理 36
7.5 被験者ごとの中止基準 37
7.6 被験者の治験中止の手続き 39
8.0 薬剤及び補助器材管理 40
8.1 薬剤及び補助器材 40
8.1.1 剤型、製造、包装及び表示 40
8.1.2 貯法 41
8.1.3 投与量及び投与方法 42
8.1.4 過量投与 42
8.2 治験薬の割付と処方及び投与手順 42
8.3 割付表作成と保管 43
8.4 盲検性の維持 43
8.5 盲検解除の手順 43
8.6 治験依頼者が提供する薬剤の管理及び廃棄 43
9.0 治験実施計画 45
9.1 治験実施手順 45
9.1.1 同意取得 45

9.1.2 人口統計学的データ、既往歴及び前治療薬 45
9.1.3 診察 46
9.1.4 体重、身長及び BMI 46
9.1.5 バイタルサイン 46
9.1.6 併用薬及び併用療法 47
9.1.7 合併症 47
9.1.8 患者日誌 47
9.1.9 完全 Mayo スコアと部分 Mayo スコア 48
9.1.10 炎症性腸疾患質問票（IBDQ） 49
9.1.11 UC 関連イベント 50
9.1.12 臨床検査 50
9.1.13 避妊 51
9.1.14 妊娠 51
9.1.15 心電図 52
9.1.16 PML 評価 52
9.1.17 結核菌検査 52
9.1.18 胸部画像検査 53
9.1.19 ファーマコゲノミクス検討のための検体の収集 53
9.1.20 薬物動態評価のための検体の収集及び解析 53
9.1.21 HAHA 及び中和抗体評価のための検体の収集 53
9.1.22 導入期への組み入れ前に中止した症例の記録 54
9.1.23 導入期への組み入れ 54
9.1.24 導入期の有効性主要評価結果の登録／維持期への組み入れ 55
9.1.25 非盲検コホートへの組み入れ 55
9.2 被験者への投与状況 56
9.3 検査・観察項目の実施時期 56
9.3.1 スクリーニング期（VISIT 1） 56
9.3.2 導入期 56
9.3.3 維持期 58
9.3.4 非盲検コホート 60
9.3.5 予定外の来院時 61

9.3.6 計画している治験全体の終了時 62
9.3.7 治験薬最終投与 16 週後 62
9.3.8 治験終了後の対応 62
9.4 生体試料の保存と廃棄 63
9.5 追跡問診 63
10.0 PRETREATMENT EVENT 及び有害事象 64
10.1 定義 64
10.1.1 Pretreatment Event 64
10.1.2 有害事象 64
10.1.3 Pretreatment Event 及び有害事象に関して考慮すべき事項 64
10.1.4 重篤な有害事象 66
10.1.5 Pretreatment Event 及び有害事象の程度 67
10.1.6 有害事象の因果関係 68
10.1.7 治験手順との因果関係 68
10.1.8 発現日 68
10.1.9 消失日 69
10.1.10 頻度 69
10.1.11 治験薬に関する処置 69
10.1.12 転帰 69
10.2 手順 70
10.2.1 有害事象等の収集及び報告 70
10.2.2 重篤な有害事象の収集及び報告 71
10.2.3 重症肝障害につながる可能性のある薬剤起因性肝機能検査異常の報告 72
10.3 重篤な有害事象の追跡調査 72
10.3.1 重篤な有害事象の治験責任医師、治験審査委員会及び規制当局への報告 72
11.0 本治験のために設置された委員会 73
11.1 PML 独立判定委員会 73
11.2 中央判定委員会 73
12.0 データマネジメント及び記録の保存 74

12.1 症例報告書 74
12.2 記録の保存 75
13.0 統計手法 76
13.1 統計及び解析計画 76
13.1.1 解析対象集団 76
13.1.2 人口統計学的及び他の基準値の特性の解析 77
13.1.3 有効性の解析 77
13.1.4 薬物動態の解析 80
13.1.5 安全性の解析 80
13.1.6 免疫原性の評価項目の解析 82
13.2 中間解析及び早期中止に関する基準 82
13.3 計画被験者数の決定 83
14.0 品質管理及び品質保証 84
14.1 治験実施医療機関へのモニタリング 84
14.2 治験実施計画書からの逸脱 84
14.3 品質保証及び規制当局による調査 84
15.0 治験の倫理的な実施 86
15.1 治験審査委員会の承認 86
15.2 同意・説明文書及び被験者の同意 87
15.3 被験者の機密保持 88
15.4 公表、開示及び臨床試験登録の指針 88
15.4.1 公表及び開示 88
15.4.2 臨床試験登録 89
15.4.3 治験成績の開示 89
15.5 損害に対する保険／補償 89
16.0 参考文献 90

本文中の図一覧

図 6.a 治験デザインの概略図 25
図 6.b 組み入れ、投与、来院時点の概略図 26
図 8.a バイアルのラベル 40
図 8.b 外箱のラベル 41

本文中の表一覧

表 7.a 併用禁止薬及び併用禁止治療の一覧表 34
表 8.a 治験薬 40
表 8.b 補助器材 41
表 8.c 投与量及び投与方法 42
表 9.a Mayo スコア 48
表 9.b 臨床検査 50
表 10.a Takeda Medically Significant AE List 67

付録一覧

付録 A 治験スケジュール 92
付録 B 治験責任医師の責務 96
付録 C 潰瘍性大腸炎診断基準（平成 25 年 1 月 17 日改訂） 97
付録 D PML チェックリスト 101
付録 E 追跡問診用紙 106

2.0 要約

<table>
<tr><td>治験依頼者：
武田薬品工業株式会社</td><td colspan="2">治験薬：
MLN0002</td></tr>
<tr><td>課題名：中等症又は重症の日本人潰瘍性大腸炎患者の導入療法及び維持療法における、MLN0002（300 mg）を点滴静注したときの有効性、安全性及び薬物動態を検討する、プラセボ対照の第 3 相多施設共同無作為化二重盲検並行群間比較試験</td><td>IND 番号：
該当せず</td><td>EudraCT 番号：
該当せず</td></tr>
<tr><td>治験実施計画書番号：MLN0002/CCT-101</td><td colspan="2">開発のフェーズ：3</td></tr>
<tr><td colspan="3">治験デザイン：
本治験は、中等症又は重症の日本人潰瘍性大腸炎患者の導入療法及び維持療法における MLN0002 の有効性、安全性及び薬物動態を検討するプラセボ対照の第 3 相多施設共同無作為化二重盲検並行群間比較試験である。本治験は、スクリーニング期、導入期、維持期及び非盲検コホートによって構成される。
スクリーニング期では、同意取得後、治験薬投与開始の 21 日前～3 日前に被験者来院のうえ、スクリーニング検査を実施する。治験薬投与開始日（Day 1）に選択・除外基準に基づき適格と判定された被験者が導入期に組み入れられる。
導入期は 2 つのコホートで構成され、コホート 1 の組み入れ完了後にコホート 2 の組み入れを開始する。コホート 1 の被験者はプラセボ群又は MLN0002 群に 1：2 の割合で無作為割付され、プラセボ又は MLN0002 300 mg を 0、2 及び 6 週目に二重盲検下で投与される。コホート 2 の被験者は MLN0002 300 mg を 0、2 及び 6 週目に非盲検下で投与される。導入期の有効性主要評価は 10 週目に実施する。
10 週目改善例は 14 週目に維持期に組み入れられる。導入期プラセボ投与例は、引き続きプラセボを 14、22、30、38、46、54 週目に二重盲検下で投与される。導入期 MLN0002 投与例は、プラセボ群又は MLN0002 群に 1：1 の割合で無作為割付され、プラセボ又は MLN0002 300 mg を 14、22、30、38、46、54 週目に二重盲検下で投与される。維持期の有効性主要評価は 60 週目に実施する。
10 週目非改善例は 10 週目に非盲検コホートへの組み入れを可とする。非盲検コホートに組み入れられた被験者は、組み入れ時を 0x 週目として、MLN0002 300 mg を 0x、2x、6x 週目及び以降 8 週おきに、最短 46 週間、最長 94 週間非盲検下で投与される。また、維持期における疾患悪化例、救済治療例、60 週目完了例も非盲検コホートへの組み入れを可とし、同様のスケジュールで継続して投与される。非盲検コホートに組み入れられた最後の被験者の 46x 週目の時点で全症例の投与を終了する。
すべての治験薬投与例について、治験薬最終投与後 16 週目に終了時検査を実施し、さらに治験薬の最終投与日から 6 ヵ月ごとに、最長 2 年間又は本剤の製造販売承認日のいずれか早い日まで追跡問診を実施する。</td></tr>
<tr><td colspan="3">導入期の主目的：
中等症又は重症の日本人潰瘍性大腸炎患者における MLN0002 の導入療法の有効性を検討する。</td></tr>
<tr><td colspan="3">導入期の副目的：
中等症又は重症の日本人潰瘍性大腸炎患者における MLN0002 の導入療法の安全性を検討する。</td></tr>
<tr><td colspan="3">維持期の主目的：
中等症又は重症の日本人潰瘍性大腸炎患者における MLN0002 の維持療法の有効性を検討する。</td></tr>
</table>

**維持期の副目的**：<br>中等症又は重症の日本人潰瘍性大腸炎患者における MLN0002 の維持療法の安全性を検討する。	
**対象**：中等症又は重症の日本人潰瘍性大腸炎患者	
**計画被験者数**：合計約 278 例。<br>導入期の無作為化例数を 246 例（MLN0002 群 164 例、プラセボ群 82 例）とする。維持期の組み入れ例数（約 140 例）を満たすため、導入期コホート 2 の組み入れ被験者数を調整できることとする。なお、抗 TNFα 抗体前治療歴のない患者を計画被験者数の 50%を目安に組み入れることとする。	**医療機関数**：<br>約 100 医療機関
**用法用量**：<br>MLN0002 300 mg 又はプラセボを 0、2、6 週目及び以降 8 週ごとに点滴静注する。	**投与経路**：<br>点滴静注
**投与期間**：54 週間（最長 154 週間）	**評価期間**：70 週間（最長 170 週間）<br>さらに治験薬の最終投与日から 6 ヵ月ごとに、最長 2 年間又は本剤の製造販売承認日のいずれか早い日まで追跡問診を実施する。
**主な選択基準**：<br>• 同意取得時の満年齢が 15 歳以上、80 歳以下の者<br>• 治験薬投与開始の 6 ヵ月以上前に、「難治性炎症性腸管障害に関する調査研究」班の潰瘍性大腸炎診断基準 改訂版（平成 24 年度）に準じて、全大腸炎又は左側大腸炎の潰瘍性大腸炎と診断された者<br>• 治験薬投与開始時の完全Mayoスコアが 6 以上、12 以下であり、かつ粘膜所見サブスコアが 2 以上として定義される中等症又は重症の活動期である者<br>• 同意取得時点で、全大腸炎又は左側大腸炎の罹病歴が 8 年以上の者、年齢が 50 歳以上の者、大腸癌の一親等以内の家族歴がある者のいずれかに該当する場合、治験薬投与開始時に全大腸内視鏡検査を実施し、大腸癌又はdysplasiaの合併が否定された者（同意取得前 1 年以内に全大腸内視鏡検査を実施している場合は、その結果を利用してもよい）<br>• 同意取得前 5 年以内に使用した前治療薬のうち、少なくとも以下の 1 剤について、治療失敗基準のいずれかを満たす者：<br>副腎皮質ホルモン薬（ステロイド）<br>・ステロイド抵抗例<br>・ステロイド依存例<br>・ステロイド不耐例<br>免疫調節薬（アザチオプリン又は 6-メルカプトプリン）<br>・免疫調節薬不応例<br>・免疫調節薬不耐例<br>抗TNFα抗体<br>・抗TNFα抗体一次無効例<br>・抗TNFα抗体二次無効例<br>・抗TNFα抗体不耐例	

主な除外基準：
- 治験薬投与開始時の部分Mayoスコアが、スクリーニング検査時から 3 ポイント以上減少した者
- 膿瘍又は中毒性巨大結腸症がある者、又はその疑いがある者
- 大腸の亜全摘又は全摘をした者
- 人工肛門、瘻孔又は高度の腸管狭窄がある者
- 治験薬投与開始前 13 日以内に 5-アミノサリチル酸経口剤、プロバイオティクス又は 30 mg/日以下のステロイド経口剤を開始した者。治験薬投与開始 14 日以上前からこれらの薬剤を使用している場合、治験薬投与開始前 13 日以内に投与量変更又は中止した者
- 治験薬投与開始前 13 日以内に 5-アミノサリチル酸又はステロイドの注腸剤・坐剤、ステロイド静注剤、30 mg/日超のステロイド経口剤、下痢型過敏性腸症候群治療薬又は潰瘍性大腸炎治療を目的とした漢方薬（大建中湯等）を使用した者
- 治験薬投与開始前 13 日以内に止瀉薬を連続 4 日以上使用した者、又は治験薬投与開始前 7 日以内に止瀉薬を使用した者
- 治験薬投与開始前 27 日以内にアザチオプリン又は 6-メルカプトプリンを使用した者。ただし、治験薬投与開始 83 日以上前からこれらの薬剤を使用し、かつ治験薬投与開始 27 日以上前から投与量不変で投与継続している場合は除く
- 治験薬投与開始前 27 日以内にシクロスポリン、タクロリムス、メトトレキサート、トファシチニブ又は潰瘍性大腸炎治療を目的とした低分子化合物の治験薬を使用した者
- 治験薬投与開始前 27 日以内にアダリムマブを使用した者、又は治験薬投与開始前 55 日以内にその他の生物学的製剤を使用した者。ただし、局所投与（加齢黄斑変性症治療のための眼内注入等）は除く
- 治験薬投与開始前 27 日以内に生ワクチン接種をした者
- 治験薬投与開始前 27 日以内に腸管切除をした者、又は治験期間中に腸管切除が必要になると予測される者
- 治験薬投与開始前 27 日以内に血球成分除去療法をした者
- 治験薬投与開始時に治療を要する大腸腺腫性ポリープがある者
- 大腸のdysplasiaの既往又は合併がある者

導入期の主な有効性評価項目：

主要評価項目：

10 週目改善率

副次評価項目：

10 週目寛解率、10 週目粘膜治癒率

維持期の主な有効性評価項目：

主要評価項目：

60 週目寛解率

副次評価項目：

持続改善率、60 週目粘膜治癒率、持続寛解率、ステロイドフリー60 週目寛解率

薬物動態の評価項目：

MLN0002 の血清中薬物濃度

安全性評価項目：

有害事象、体重、バイタルサイン、心電図、臨床検査（血液生化学検査、血液学的検査、尿検査）

免疫原性の評価項目：

HAHA、中和抗体

統計手法：

(1) 導入期の解析方法

「導入期の最大の解析対象集団（導入期に無作為化され、導入期に治験薬を少なくとも 1 回以上投与された被験者）」を対象として以下の集計解析を行う。

主解析として、導入期の主要評価項目である「10 週目改善率」について、導入期の投与群別に頻度集計を行う。「抗 TNFα 抗体前治療歴の有無」により層別したうえで同様の集計を行い、「抗 TNFα 抗体前治療歴の有無」を層別因子とした Cochran-Mantel-Haenszel 検定を行う。MLN0002 の導入療法の有効性を検討するため、導入期の主解析の結果を以下のとおり解釈する。

- 導入期の主解析における 10 週目改善率の検定結果が有意であった場合に、10 週目改善率について MLN0002 投与のプラセボ投与に対する優越性が検証されたとする。

副解析として、10 週目寛解率について導入期の主解析と同様の解析を行う。MLN0002 の導入療法の有効性を検討するため、導入期の副解析の結果を以下のとおり解釈する。

- 導入期の主解析における 10 週目改善率の検定結果が有意であり、かつ、10 週目寛解率の検定結果が有意であった場合に、10 週目寛解率について MLN0002 投与のプラセボ投与に対する優越性が検証されたとする。

(2) 維持期の解析方法

「維持期の最大の解析対象集団（維持期に無作為化され、維持期に治験薬を少なくとも 1 回以上投与された被験者）」を対象として以下の集計解析を行う。

主解析として、維持期の主要評価項目である「60 週目寛解率」について、維持期の投与群ごとに頻度集計を行う。「抗 TNFα 抗体前治療歴の有無」により層別したうえで同様の集計を行い、「抗 TNFα 抗体前治療歴の有無」を層別因子とした Cochran-Mantel-Haenszel 検定を行う。MLN0002 の維持療法の有効性を検討するため、維持期の主解析の結果を以下のとおり解釈する。

- 維持期の主解析における 60 週目寛解率の検定結果が有意であった場合に、60 週目寛解率について MLN0002 投与のプラセボ投与に対する優越性が検証されたとする。

(3) 有意水準

導入期と維持期では治験の目的が異なるため、導入期及び維持期の有意水準をそれぞれ 5%とし、導入期と維持期の間の多重性の調整は行わない。

計画被験者数の設定根拠：

海外で実施した中等症から重症の潰瘍性大腸炎患者の導入療法及び維持療法におけるプラセボ対照第 3 相二重盲検比較試験（C13006）の成績を参考に、本治験の MLN0002 投与とプラセボ投与による 10 週目改善率を 57.1%と 35.5%、10 週目寛解率を 16.9%と 5.4%、60 週目寛解率を 43.3%と 15.9%と想定した。

導入期に関して、10 週目改善率について有意水準を両側 5%として Pearson のカイ二乗検定を適用する場合、MLN0002 投与群とプラセボ投与群の例数の比を 2:1 として 90%以上の検出力を確保するためには、MLN0002 投与群に 164 例、プラセボ投与群に 82 例、合計 246 例必要となることから、導入期の主要評価項目の評価可能例数（「導入期に無作為化され、導入期に治験薬を少なくとも 1 回以上投与された被験者」の例数）としてこれらの例数を設定した。10 週目寛解率について、本例数のもとで同様の解析を行ったときの 10 週目改善率との同時検出力は 69%である。

維持期に関して、60 週目寛解率について有意水準を両側 5%として Pearson のカイ二乗検定を適

用する場合、80%以上の検出力を確保するためには、各群 42 例、合計 84 例必要となる。同様に、90%以上の検出力を確保するためには、各群 55 例、合計 110 例必要となることから、維持期の主要評価項目の評価可能例数（「維持期に無作為化され、維持期に治験薬を少なくとも 1 回以上投与された被験者」の例数）としてこれらの例数を設定した。導入期の盲検性維持のため、維持期に組み入れられた被験者には「導入期にプラセボ群に割り付けられた被験者」が含まれることから、維持期の組み入れ例数としては、維持期の主要評価項目の検出力 80%、90%を確保する例数としてそれぞれ 110 例、140 例が必要となる。

3.0 略語

5-ASA	5-aminosalicylic acid	5-アミノサリチル酸
6-MP	6-mercaptopurine	6-メルカプトプリン
ALT	alanine aminotransferase	アラニンアミノトランスフェラーゼ
ALP	alkaline phosphatase	アルカリホスファターゼ
AST	aspartate aminotransferase	アスパラギン酸アミノトランスフェラーゼ
AZA	Azathioprine	アザチオプリン
BMI	body mass index	肥満指数
BUN	blood urea nitrogen	血中尿素窒素
CD	Crohn’s disease	クローン病
CDAI	Crohn’s Disease Activity Index	クローン病活動性指数
CRP	C-reactive protein	C 反応性蛋白
ELISA	Enzyme-Linked ImmunoSorbent Assay	酵素結合免疫吸着法
FDA	Food and Drug Administration	米国食品医薬品局
GCP	Good Clinical Practice	医薬品の臨床試験の実施に関する基準
γ-GTP	γ-glutamyl transpeptidase	ガンマグルタミルトランスペプチダーゼ
HAHA	human anti-human antibody	ヒト抗ヒト抗体
HBs	hepatitis B surface antigen	B 型肝炎ウイルス表面抗原
hCG	human chorionic gonadotropine	ヒト絨毛性性腺刺激ホルモン
HCV	hepatitis C virus	C 型肝炎ウイルス
HIV	human immunodeficiency virus	ヒト免疫不全ウイルス
IBDQ	Inflammatory Bowel Disease Questionnaire	炎症性腸疾患質問票
ICH	International Conference on Harmonisation	日米 EU 医薬品規制調和国際会議
INR	International normalized ratio	国際標準比
MedDRA	Medical Dictionary for Regulatory Activities	医薬品規制用語集
MHRA	Medicines and Healthcare products Reguratory Agency	英国医薬品庁
PMDA	Pharmaceutical and Medical Devices Agency	医薬品医療機器総合機構
PML	progressive multifocal leukoencephalopathy	進行性多巣性白質脳症
SUSARs	suspected serious unexpected adverse reactions	未知の重篤な副作用
TNF	Tumor Necrosis Factor	腫瘍壊死因子
UC	Ulcerative colitis	潰瘍性大腸炎
WHO	World Health Organization	世界保健機関

3.1 本治験における用語の定義

本治験では、以下のように定義する。

用語	定義
寛解	完全Mayoスコアが2以下かつすべてのサブスコアが1以下
改善	下記の2条件をともに満たした場合：<br>• 完全Mayoスコアがベースラインから3ポイント以上減少かつ30%以上減少（完全Mayoスコアが実施されないVISITの場合は、部分Mayoスコアがベースラインから2ポイント以上減少かつ25%以上減少）<br>• 血便サブスコアがベースラインから1ポイント以上減少又は血便サブスコアが1以下
粘膜治癒	完全Mayoスコアの粘膜所見サブスコアが1以下
持続改善	10週目及び60週目の両時点で改善
持続寛解	10週目及び60週目の両時点で寛解
ステロイドフリー<br>60週目寛解	治験薬投与開始時にステロイドを使用していたが、60週目の時点でステロイド不使用かつ寛解状態
疾患悪化	維持期において、部分Mayoスコアが2回の来院*で連続して10週目の値から3ポイント以上増加、かつ部分Mayoスコアが5以上（10週目の値が6を超えている場合、2回の来院*で連続して9）<br>又は、非盲検コホートにおいて、部分Mayoスコアが2回の来院*で連続して10x週目の値から3ポイント以上増加、かつ部分Mayoスコアが5以上（10x週目の値が6を超えている場合、2回の来院*で連続して9）
非改善状態の持続	治験薬初回投与から20週間、改善基準を満たさず、かつ部分Mayoスコアが5以上
救済治療	UC治療のために必要とされる新たな薬剤の投与、治験薬投与開始時から使用している薬剤の増量（止瀉薬は除く）又は併用療法の実施（膿瘍に対する排膿、肛門部病変に対するシートン法は除く）<br>非盲検コホートで使用可能な薬剤については、非盲検コホート組み入れ後に増量又は新たに投与を開始しても救済治療とみなさない
治療失敗	疾患悪化、救済治療又は治験薬に関連した有害事象による中止

* 2回の来院には、UCが悪化した場合等の予定外の来院も含まれる。来院と来院の間隔は、可能な限り1週間以上空けることが望ましい。

4.0 はじめに

4.1 背景

潰瘍性大腸炎（UC）は再燃と寛解を繰り返す炎症性腸疾患である。病変は直腸から口側に向かって連続的に広がり、最大で直腸から結腸に及ぶ。特徴的な症状は慢性の粘血便、血便であり、その他にも頻回の下痢、腹痛、発熱、食欲不振、貧血等を認める。頻回の下痢の場合は外出も不自由になり、Quality of Life（QOL）も低下する。

原因はまだ明らかになっていないが、最近の研究により、腸内細菌をはじめとした管腔内抗原に対する免疫寛容の破綻と、サイトカインの異常が関与していると考えられている。根本治療は確立していないため、内科的治療戦略の基本は、薬物療法により速やかな寛解導入を図り、寛解を長期に維持すること（寛解維持）である。厚生労働省の難治性炎症性腸管障害研究班で改訂されたガイドラインや治療指針[1)]において薬物療法の指針が示されているが、病型や重症度に応じた細やかな対応が必要である。

MLN0002 は、ヒトリンパ球$\alpha_4\beta_7$インテグリンに特異的に結合する遺伝子組換えヒト化免疫グロブリンG_1モノクローナル抗体[2)]である。現在、UC及びクローン病（CD）を対象とした開発が国内外で進められている。

MLN0002 はリンパ球の細胞膜蛋白である$\alpha_4\beta_7$インテグリンに結合することにより、その主要リガンドであるmucosal addressin cell adhesion molecule-1（MAdCAM-1；パイエル板や腸間膜リンパ節の高内皮細静脈、腸管の粘膜固有層の細静脈の血管内皮細胞に強く発現する膜貫通型糖蛋白）との接着を阻害してリンパ球の腸管粘膜及び腸管関連リンパ系組織への遊走を阻害する[2)]。このことから、全身免疫抑制を伴わない消化管選択的な免疫調節作用により病的な腸管炎症を抑制することで、UC及びCDに対し、従来の療法やその他の生物学的製剤よりも優れた安全性で効果を発揮すると期待されている[3), 4)]。

4.2 臨床試験成績

海外では 14 の第 1 相及び第 2 相試験を実施している他、UC 及び CD 患者を対象とした以下の 4 つの第 3 相試験（GEMINI プログラム）を実施した。2013 年 10 月時点で、UC 及び CD 患者を対象とした長期投与試験（C13008）を除く試験が終了しており、欧州及び米国で承認申請中である。国内では UC 患者を対象とした第 1 相試験を実施した。

試験番号	試験名
C13006	中等症から重症の UC 患者の導入療法及び維持療法におけるプラセボ対照第 3 相二重盲検比較試験（GEMINI 1）
C13007	中等症から重症の CD 患者の導入療法及び維持療法におけるプラセボ対照第 3 相二重盲検比較試験（GEMINI 2）
C13011	抗 TNFα 抗体治療に失敗した中等症から重症の CD 患者の導入療法におけるプラセボ対照第 3 相二重盲検比較試験（GEMINI 3）
C13008	MLN0002 の第 2 相及び第 3 相試験に参加した UC 又は CD 患者の第 3 相非盲検長期安全性及び有効性試験（GEMINI LTS）

4.2.1 海外第 1 相及び第 2 相試験成績の概要

海外で実施された第 1 相及び第 2 相試験における主な結果の概要を以下に示した。

- MLN0002 の薬物動態は、2～10 mg/kg の用量で線形であった。MLN0002 の消失半減期は 15～22 日であった [5)]。
- 2～10 mg/kgの用量で初回投与後から、$\alpha_4\beta_7$受容体の飽和が認められた。$\alpha_4\beta_7$受容体の飽和は最終投与後数ヵ月間持続し、その持続期間は用量依存的に長くなった [5)]。
- MLN0002 に対するヒト抗ヒト抗体（抗MLN0002 抗体、以下、HAHA）はMLN0002 を血中から速やかに消失させ、その結果、MLN0002 の曝露量が減少するとともに$\alpha_4\beta_7$受容体飽和が失われることが示唆された。HAHA発現率は用量依存的に減少した。
- UC における 6 週目寛解率は 32%､CD における 8 週目寛解率は 37%であり、いずれの対象においても MLN0002 のプラセボに対する優越性が確認された [6), 7)]。安全性上の問題は特になく、有害事象発現頻度はプラセボと大きな相違は見られなかった。

4.2.2 海外第 3 相試験成績の概要

海外で実施された 3 つの第 3 相試験（C13006、C13007 及び C13011）では、いずれも MLN0002 300 mg 又はプラセボが投与された。C13006 試験では主要評価項目に設定した導入期の改善率及び維持期の寛解率のそれぞれにおいてプラセボに対する優越性が認められた [8)]。C13007 試験では導入期に 2 つの主要評価項目が設定されており、クローン病活動性指数（CDAI）-100 改善率では有意差が

認められなかったが、導入期及び維持期のそれぞれの寛解率においてプラセボに対する優越性が認められた[9)]。C13011 試験では主要評価項目に設定した抗 TNFα 抗体治療に失敗した部分集団（全集団の 75%）における 6 週目寛解率ではプラセボに対する優越性は認められなかったものの、全集団における 6 週目寛解率ではプラセボに対する有意差が見られた。また、10 週目寛解率では抗 TNFα 抗体治療に失敗した部分集団及び全集団のいずれにおいてもプラセボに対する有意差が見られた[10)]。

また、有害事象及び重篤な有害事象の発現頻度は、MLN0002 群全体とプラセボ群で大きな違いはなかった。完了した又は継続中の臨床開発プログラム全症例を通じて進行性多巣性白質脳症（PML）は認められなかった。

4.2.3 国内第 1 相試験成績の概要

国内では、UC患者を対象とした第 1 相反復投与試験（CPH-001）を実施し、MLN0002 150 mg又は 300 mgを 0、2、6 週目に投与したときの安全性、薬物動態及び薬力学的作用を検討した。300 mgまでの忍容性は良好で、注射部位反応やアナフィラキシー反応は認められなかった。また、血清中MLN0002 濃度は用量比に応じて増加し、薬力学的作用の指標となる$\alpha_4\beta_7$受容体飽和度は投与初日から概ね継続して飽和が認められた[11)]。

4.3 本治験を計画した根拠

- 海外で実施された 3 つの第 3 相試験（C13006、C13007 及び C13011）では、抗 TNFα 抗体を含む既存の薬物治療に失敗した、中等症から重症の UC 又は CD 患者を対象とし、導入期及び維持期のそれぞれにおいて、MLN0002 300 mg の有効性及び安全性をプラセボを対照として検討した。その結果、MLN0002 の有効性評価では、いずれの試験においても、プラセボに対する有意差が見られた。また、安全性評価でも特に問題は見られなかった。これらの結果は、第 2 相試験までに得られた成績と一貫していた。
- 国内では、UC患者を対象とし、MLN0002 150 mg及び 300 mgを 0、2、6 週目に投与したときの安全性、血清中MLN0002 濃度及び$\alpha_4\beta_7$受容体飽和度を検討した。その結果、血清中MLN0002 濃度は国内外で類似しており、$\alpha_4\beta_7$受容体飽和度は投与初日から概ね継続して飽和が認められた。また、安全性についても問題は見られなかった。

以上のことから、中等症から重症の日本人 UC 患者における MLN0002 300 mg の有効性及び安全性を検討する第 3 相試験として本治験を計画した。

薬物の有効性及び安全性等の薬物反応における遺伝子多型の寄与を評価するために、ファーマコゲノミクス解析を実施する可能性がある。被験者がファーマコゲノミクス検討用検体の採取に参加することは必須とはしない。

ファーマコゲノミクスは発展途上の分野であるため、現在多くの遺伝子及びそれらの機能については、まだ完全に解明されていない。将来得られるデータによっては、薬物反応又は疾患における一部遺伝子の役割が示唆されることも考えられ、その場合にはファーマコゲノミクス検討用検体を利用して探索的調査を行う可能性がある。

5.0 治験の目的及び評価項目

5.1 目的

5.1.1 導入期の主目的

中等症又は重症の日本人 UC 患者における MLN0002 の導入療法の有効性を検討する。

5.1.2 導入期の副目的

中等症又は重症の日本人 UC 患者における MLN0002 の導入療法の安全性を検討する。

5.1.3 維持期の主目的

中等症又は重症の日本人 UC 患者における MLN0002 の維持療法の有効性を検討する。

5.1.4 維持期の副目的

中等症又は重症の日本人 UC 患者における MLN0002 の維持療法の安全性を検討する。

5.2 評価項目

5.2.1 導入期の有効性評価項目

5.2.1.1 主要評価項目

- 10 週目改善率

5.2.1.2 副次評価項目

- 10 週目寛解率
- 10 週目粘膜治癒率

5.2.1.3 探索的評価項目

- 抗 TNFα 抗体前治療歴のない患者部分集団及び抗 TNFα 抗体治療失敗と定義される患者部分集団における 10 週目改善率、10 週目寛解率及び 10 週目粘膜治癒率

- ステロイドのみの治療失敗、免疫調節薬治療失敗（抗 TNFα 抗体治療失敗を除く）と定義される患者部分集団における 10 週目改善率、10 週目寛解率及び 10 週目粘膜治癒率
- 10 週目 IBDQ スコアのベースラインからの変化量
- 部分 Mayo スコアの経時的推移

5.2.2 維持期の有効性評価項目

5.2.2.1 主要評価項目

- 60 週目寛解率

5.2.2.2 副次評価項目

- 持続改善率
- 60 週目粘膜治癒率
- 持続寛解率
- ステロイドフリー60 週目寛解率

5.2.2.3 探索的評価項目

- 経口ステロイド使用量の 10 週目からの変化量及び変化率の経時的推移
- 疾患悪化までの期間
- 治療失敗までの期間
- 抗 TNFα 抗体前治療歴のない患者部分集団及び抗 TNFα 抗体治療失敗と定義される患者部分集団における 60 週目寛解率及び 60 週目粘膜治癒率
- ステロイドのみの治療失敗、免疫調節薬治療失敗（抗 TNFα 抗体治療失敗を除く）と定義される患者部分集団における 60 週目寛解率及び 60 週目粘膜治癒率
- 38 週目及び 60 週目 IBDQ スコアの 14 週目からの変化量
- 部分 Mayo スコアの経時的推移
- 主要な UC 関連イベント（UC に関連する入院、腸管切除）までの期間

5.2.3 薬物動態の評価項目

- MLN0002 の血清中薬物濃度

5.2.4 安全性評価項目

- 有害事象
- 体重
- バイタルサイン
- 心電図
- 臨床検査（血液生化学検査、血液学的検査、尿検査）

5.2.5 免疫原性の評価項目

- HAHA、中和抗体

6.0 治験のデザイン

6.1 治験デザイン

本治験は、中等症又は重症の日本人 UC 患者の導入療法及び維持療法における MLN0002 の有効性、安全性及び薬物動態を検討するプラセボ対照の第 3 相多施設共同無作為化二重盲検並行群間比較試験である。本治験は、スクリーニング期、導入期、維持期及び非盲検コホートによって構成される。

スクリーニング期では、同意取得後、治験薬投与開始の 21 日前～3 日前に被験者来院のうえ、スクリーニング検査を実施する。治験薬投与開始日（Day 1）に選択・除外基準に基づき適格と判定された被験者が導入期に組み入れられる。

導入期は 2 つのコホートで構成され、コホート 1 の組み入れ完了後にコホート 2 の組み入れを開始する。コホート 1 の被験者はプラセボ群又はMLN0002 群に 1 : 2 の割合で無作為割付[1]され、プラセボ又はMLN0002 300 mgを 0、2 及び 6 週目に二重盲検下で投与される。コホート 2 の被験者はMLN0002 300 mgを 0、2 及び 6 週目に非盲検下で投与される。導入期の有効性主要評価は 10 週目に実施する。

10 週目改善例は 14 週目に維持期に組み入れられる。導入期プラセボ投与例は、引き続きプラセボを 14、22、30、38、46、54 週目に二重盲検下で投与される。導入期MLN0002 投与例は、プラセボ群又はMLN0002 群に 1 : 1 の割合で無作為割付[2]され、プラセボ又はMLN0002 300 mgを 14、22、30、38、46、54 週目に二重盲検下で投与される。維持期の有効性主要評価は 60 週目に実施する。

10 週目非改善例は 10 週目に非盲検コホートへの組み入れを可とする。非盲検コホートに組み入れられた被験者は、組み入れ時を 0x 週目として、MLN0002 300 mg を 0x、2x、6x 週目及び以降 8 週おきに、最短 46 週間、最長 94 週間非盲検下で投与される。また、維持期における疾患悪化例、救済治療例、60 週目完了例も非盲検コホートへの組み入れを可とし、同様のスケジュールで継続して投与される。非盲検コホートに組み入れられた最後の被験者の 46x 週目の時点で全症例の投与を終了する。

すべての治験薬投与例について、治験薬最終投与後 16 週目に終了時検査を実施する。さらに治験薬の最終投与日から 6 ヵ月ごとに、最長 2 年間又は本剤の製造販売承認日のいずれか早い日まで追跡問診を実施する（9.5 項参照）。

[1] 抗 TNFα 抗体前治療歴の有無、免疫調節薬併用の有無、ステロイド併用の有無及び治験実施医療機関による動的割付を実施する。

[2] 抗 TNFα 抗体前治療歴の有無、免疫調節薬併用の有無、ステロイド併用の有無、治験実施医療機関、導入期コホート及び 10 週目寛解／非寛解による動的割付を実施する。

計画被験者数は合計約 278 例であり、導入期の無作為化例数を 246 例（MLN0002 群 164 例、プラセボ群 82 例）とする。維持期の組み入れ例数を 110～140 例とし、それを満たすため、導入期のコホート 2 に組み入れる被験者数を調整できることとする。なお、抗 TNFα 抗体前治療歴のない患者を計画被験者数の 50%を目安に組み入れることとする。

治験デザインの概略図を図 6.a、組み入れ、投与、来院時点の概略図を図 6.b に示す。検査・観察・評価のスケジュールは付録 A 参照。

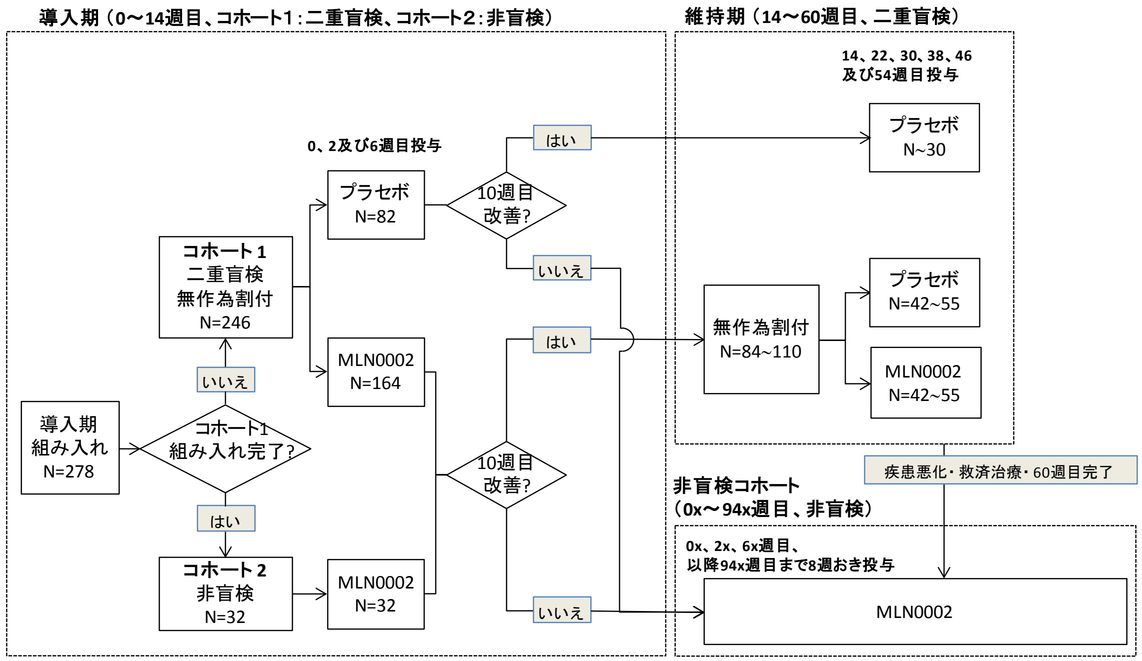

図 6.a　　治験デザインの概略図

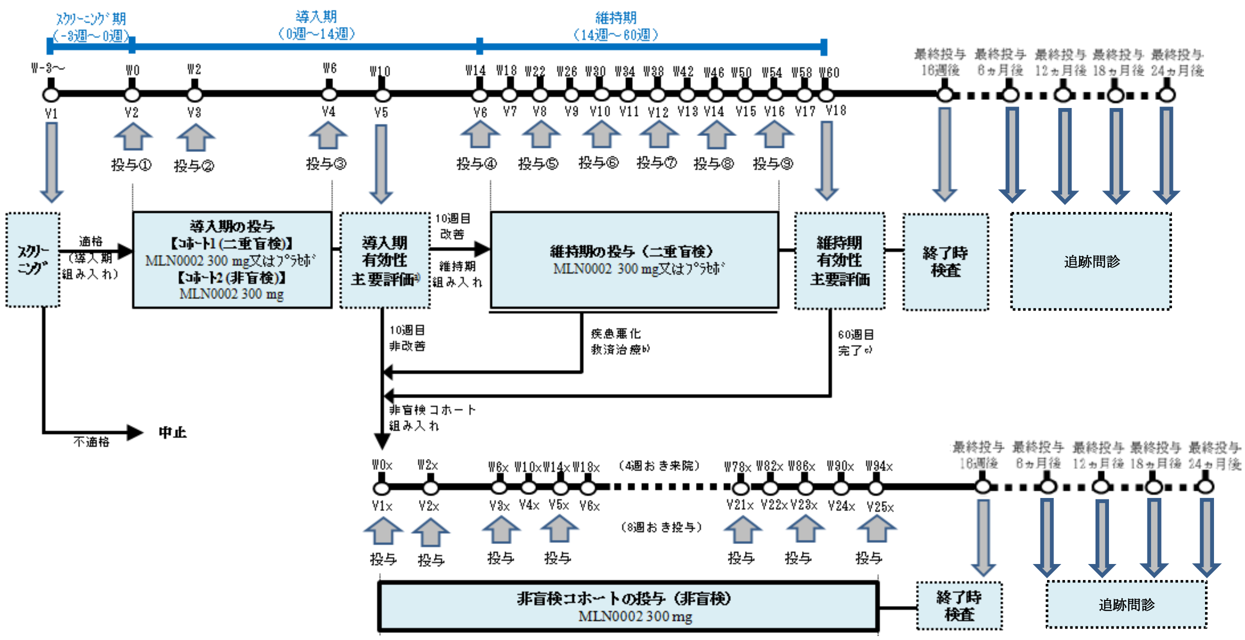

W：Week（週）、V：VISIT（来院）

a)　10 週目（導入期有効性主要評価）改善例を 14 週目に維持期に組み入れる。10 週目非改善例は、10 週目の検査終了後に非盲検コホートへの組み入れを可とする。

b)　維持期における疾患悪化例及び救済治療例は、維持期中止時検査終了後、非盲検コホートへの組み入れを可とする。ただし、維持期の最終投与から非盲検コホート組み入れまでは、3～9 週間の間隔を空けること。

c)　60 週目（維持期有効性主要評価）完了例は、60 週目の検査終了後に非盲検コホートへの組み入れを可とする。

図 6.b　　　組み入れ、投与、来院時点の概略図

6.2　　治験デザイン、投与量、評価項目の設定根拠

6.2.1　　治験対象集団の設定根拠

「難治性炎症性腸管障害に関する調査研究」班の潰瘍性大腸炎治療指針[1]（平成 24 年度）では、薬物療法の指針として、軽症から中等症のUC患者の寛解導入療法には、サラゾスルファピリジンや 5-アミノサリチル酸（5-ASA）の経口剤を中心にステロイドの坐剤や注腸剤、サラゾスルファピリジン坐剤、5-ASA注腸剤等を推奨している。また、これらの治療で効果不十分な場合や重症例には、ステロイドの経口剤を推奨している。

一方で、ステロイドの適切な使用にもかかわらず効果不十分な場合（ステロイド抵抗例）やステロイド投与中は症状が安定しているがステロイドの減量に伴い再燃増悪する場合（ステロイド依存例）を難治例と定義している。難治例に対しては、免疫調節薬や抗 TNFα 抗体が推奨されているが、これらの治療薬でも

効果不十分な場合や、投与継続中の効果消失が問題となっている。MLN0002 は、既存治療薬とは異なる作用機序を有しており、このような患者にも有効であることが海外試験成績から示唆されている。

以上のことから、本治験では、中等症から重症の日本人 UC 患者で、既存の薬物治療（ステロイド／免疫調節薬／抗 TNFα 抗体のいずれか）に失敗した患者を導入期の対象集団とした。

6.2.2 デザイン及び計画被験者数の設定根拠

潰瘍性大腸炎治療指針 [1)]では、寛解導入後も再燃を予防するため寛解維持療法を行うこととしている。しかし、導入療法で有効な薬剤が必ずしも維持療法に有効とは限らない。導入療法及び維持療法それぞれの有効性及び安全性を客観的に検討するため、導入期及び維持期のそれぞれにおいて、プラセボ対照の無作為化二重盲検比較デザインを設計した。また、維持療法における有効性及び安全性を検討するため、維持期の無作為化集団には導入期 MLN0002 投与改善例のみを組み入れるエンリッチメントデザインとした。

導入期はプラセボ対照の無作為化二重盲検並行群間比較を行うコホート 1 に加え、維持期の症例数を確保するため、非盲検下で MLN0002 を投与するコホート 2 を設定した。

盲検性を維持するため、導入期の割付薬剤にかかわらず、すべての 10 週目改善例を維持期に組み入れることとした。導入期 MLN0002 投与改善例は、維持療法の有効性及び安全性評価のためプラセボ群又は MLN0002 群に無作為割付を行い、導入期プラセボ投与改善例は、引き続きプラセボ群に割り付けることとした。

倫理的な配慮から非盲検コホートを設定し、導入期非改善例、維持期における疾患悪化例、救済治療例、60 週目完了例は、最短 46 週間、最長 94 週間の MLN0002 継続投与が可能なデザインとした。

計画被験者数の設定根拠については 13.3 項を参照。

6.2.3 プラセボ対照群の設定根拠

本治験は、中等症から重症の UC 患者で、既存の薬物治療（ステロイド／免疫調節薬／抗 TNFα 抗体のいずれか）に失敗した患者を対象としているため、適切な実薬対照が設定できない。有効性及び安全性を客観的に評価するため、プラセボを対照群として設定した。

6.2.4 用法・用量及び評価期間の設定根拠

UC患者を対象とした海外第 3 相試験（C13006）では、MLN0002 300 mgを 0、2、6 週目及び以降 8 週ごと又は 4 週ごとに投与したとき、いずれの用法でも同程度の有効性が認められ、安全性にも問題はなかった [8)]。血清中MLN0002 濃度は国内外で類似しており、$\alpha_4\beta_7$受容体飽和度は投与初日から概ね継続して飽和が認められた。以上のことから、本治験では、C13006 試験と同様、MLN0002 300 mg を 0、2、6 週目及び以降 8 週ごとに投与する用法・用量とした。

また、C13006 試験の結果から、導入療法の延長による有効性の向上が期待された。そこで、導入期の有効性主要評価を投与 10 週目に実施することとした。14 週目投与以降を維持期とし、C13006 試験同様、維持期組み入れから 46 週後（60 週目）に維持期の有効性主要評価を実施することとした。

一方、10 週目非改善例は、本剤の再導入及び維持療法を実施するため、非盲検コホートへの組み入れを可とし、MLN0002 300 mg を 0x、2x、6x 週目及び以降 8 週おきに、最短 46 週間、最長 94 週間投与することとした。また、維持期における疾患悪化例、救済治療例、60 週目完了例も非盲検コホートへの組み入れを可とし、同様のスケジュールで投与を継続してよいこととした。C13006 試験では 0、2 週目及び以降 4 週ごとに投与する用法における安全性が確認されており、非盲検コホートへの組み入れは安全性上問題ないと判断した。

MLN0002 の消失半減期は、2～10 mg/kgの用量において 15～22 日であった [5)]。また$\alpha_4\beta_7$受容体の飽和は、300 mgの用量において最終投与後 16 週間持続し、それ以降は経時的に飽和度が低下した。治験薬を投与されたすべての被験者について、最終投与後の安全性を評価するため、海外試験同様、消失半減期の 5 倍以上の期間に相当し、かつ$\alpha_4\beta_7$受容体の飽和が持続する最終投与 16 週後に終了時検査を実施することとした。

α_4インテグリン拮抗薬のナタリズマブが投与された患者においてPMLの症例が報告されている [12)]。MLN0002 は腸管指向性$\alpha_4\beta_7$インテグリンに特異的に結合するため、PMLリスク上昇をもたらす可能性は低いと考えられ、これまでの国内外の臨床試験においてPMLは認められていない。しかしながら、現時点では、MLN0002 が治験段階にあることから投与例数が多くはなく、潜在的なPMLリスクを否定することが困難である。そこで本剤の潜在的なPMLリスクを評価するため、治験薬最終投与から 2 年間、6 ヵ月ごとに追跡問診を実施することとした。

6.2.5 評価項目の設定根拠

C13006 試験では、UC に対する疾患活動性評価指標として Mayo スコアが使用された [8)]。Mayo スコアは国内外を問わず、UC に対する有効性評価指標として広く用いられており、本治験における評価指標に設定した。Mayo スコアに基づく改善及び寛解の定義は、C13006 試験と同様に設定した。

6.3 治験全体の中止又は治験実施医療機関での治験中止

6.3.1 治験全体の中止基準

治験依頼者は、以下の基準に一つ以上該当する場合には、治験を速やかに中止する。

- 治験薬の安全性又は有効性に関して、当該化合物の既知のリスク／ベネフィットプロファイルの変化を示す新しい情報又は別の評価が得られ、被験者の治験参加に関してリスク／ベネフィットがもはや許容できなくなった場合。
- 治験の主目的の達成又は被験者の安全性を危うくする重大な Good Clinical Practice（GCP）違反があった場合。

6.3.2 治験実施医療機関での治験中止の基準

治験実施医療機関（治験責任医師を含む）が GCP、治験実施計画書又は契約書に重大な違反を犯したことが判明した場合、適正な治験の実施ができなくなった場合又は契約上の合意により認められた場合には、当該治験実施医療機関は治験依頼者から治験の中止を求められることがある。

6.3.3 治験全体又は治験実施医療機関での治験中断及び中止に関する手順

治験依頼者、治験審査委員会又は規制当局が、治験全体又はある治験実施医療機関での治験の中断又は中止を決定した場合には、当該治験に対して定められた手順が治験依頼者によって指示される。治験の中断又は中止に際して、当該治験実施医療機関はその手順に従うものとする。

7.0 被験者の選定及び中止の基準

導入期への組み入れ前に、検査結果を含むすべての選択・除外基準を確認する必要がある。

7.1 選択基準

以下の基準に基づいて被験者の適格性を判定する。

1. 治験の内容を理解し、それを遵守する能力があると治験責任医師又は治験分担医師が判断した者
2. 治験手順が行われる前に、同意・説明文書に被験者、又は該当する場合に被験者の代諾者による署名及び日付記入ができる者
3. 性別不問、同意取得時の満年齢が 15 歳以上、80 歳以下の者
4. 妊娠する可能性のある女性パートナーを持つ、避妊手術を受けていない男性の場合、同意取得時から治験薬最終投与後 6 ヵ月まで、適切な避妊の実施に同意する者
5. 避妊治療を受けていない男性パートナーを持つ、妊娠する可能性のある女性（避妊手術を受けていない者、又は最終月経から 2 年未満の者）の場合、同意取得時から治験薬最終投与後 6 ヵ月まで、適切な避妊の実施に同意する者
6. 治験薬投与開始の 6 ヵ月以上前に、「難治性炎症性腸管障害に関する調査研究」班の潰瘍性大腸炎診断基準 改訂版（平成 24 年度）（付録 C 参照）に準じて、全大腸炎又は左側大腸炎の UC と診断された者
7. 治験薬投与開始時の完全 Mayo スコアが 6 以上、12 以下であり、かつ粘膜所見サブスコアが 2 以上として定義される中等症又は重症の活動期である者
8. 同意取得時点で、全大腸炎又は左側大腸炎の罹病歴が 8 年以上の者、年齢が 50 歳以上の者、大腸癌の一親等以内の家族歴がある者のいずれかに該当する場合、治験薬投与開始時に全大腸内視鏡検査を実施し、大腸癌又は dysplasia の合併が否定された者（同意取得前 1 年以内に全大腸内視鏡検査を実施している場合は、その結果を利用してもよい）
9. 同意取得前 5 年以内に使用した前治療薬のうち、少なくとも以下の 1 剤について、治療失敗基準のいずれかを満たす者：

- 副腎皮質ホルモン薬（ステロイド）
  - ステロイド抵抗例：40 mg/日以上[3]（経口又は静注）を 1 週間以上、又は 30 mg/日以上 40 mg/日未満（経口又は静注）を 2 週間以上投与したが、効果不十分であった者
  - ステロイド依存例 : 経口又は静注のステロイド漸減中に症状が再燃し、10 mg/日未満への減量が困難な者
  - ステロイド不耐例 : 副作用（クッシング症候群、骨減少症/骨粗鬆症、高血糖、不眠症、感染症等）により投与を継続できなかった者
- 免疫調節薬（アザチオプリン（AZA）又は 6-メルカプトプリン（6-MP））
  - 免疫調節薬不応例：12 週間以上投与したが、効果不十分であった者
  - 免疫調節薬不耐例：副作用（悪心／嘔吐、腹痛、膵炎、肝機能検査異常、リンパ球減少症、TPMT 遺伝子変異、感染症等）により投与を継続できなかった者
- 抗 TNFα 抗体
  - 抗 TNFα 抗体一次無効例 : 添付文書に記載の用法用量で導入療法を実施したが、効果不十分であった者
  - 抗 TNFα 抗体二次無効例 : 効果を認めた後の計画的維持療法中に症状が再燃した者（再燃していないにもかかわらず投与中止した場合は該当しない）
  - 抗 TNFα 抗体不耐例：副作用（投与時反応、脱髄疾患、うっ血性心不全、感染症等）により投与を継続できなかった者

7.2 除外基準

以下の基準のいずれかに該当する被験者は本治験の対象としない。

1. 治験薬投与開始時の部分 Mayo スコアが、スクリーニング検査時から 3 ポイント以上減少した者
2. 膿瘍又は中毒性巨大結腸症がある者、又はその疑いがある者
3. 大腸の亜全摘又は全摘をした者
4. 人工肛門、瘻孔又は高度の腸管狭窄がある者
5. ナタリズマブ、エファリズマブ又はリツキシマブの投与歴がある者
6. 治験薬投与開始前 13 日以内に 5-ASA 経口剤、プロバイオティクス又は 30 mg/日以下のステロイド経口剤を開始した者。治験薬投与開始 14 日以上前からこれらの薬剤を使用している場合、治験薬投与開始前 13 日以内

[3] ステロイド投与量はプレドニゾロン換算量として表記する。以下、同様とする。

に投与量変更又は中止した者

7. 治験薬投与開始前 13 日以内に 5-ASA 又はステロイドの注腸剤・坐剤、ステロイド静注剤、30 mg/日超のステロイド経口剤、下痢型過敏性腸症候群治療薬又は UC 治療を目的とした漢方薬（大建中湯等）を使用した者
8. 治験薬投与開始前 13 日以内に止瀉薬を連続 4 日以上使用した者、又は治験薬投与開始前 7 日以内に止瀉薬を使用した者
9. 治験薬投与開始前 27 日以内に AZA 又は 6-MP を使用した者。ただし、治験薬投与開始 83 日以上前からこれらの薬剤を使用し、かつ治験薬投与開始 27 日以上前から投与量不変で投与継続している場合は除く
10. 治験薬投与開始前 27 日以内にシクロスポリン、タクロリムス、メトトレキサート、トファシチニブ又は UC 治療を目的とした低分子化合物の治験薬を使用した者
11. 治験薬投与開始前 27 日以内にアダリムマブを使用した者、又は治験薬投与開始前 55 日以内にその他の生物学的製剤を使用した者。ただし、局所投与（加齢黄斑変性症治療のための眼内注入等）は除く
12. 治験薬投与開始前 27 日以内に生ワクチン接種をした者
13. 治験薬投与開始前 27 日以内に腸管切除をした者、又は治験期間中に腸管切除が必要になると予測される者
14. 治験薬投与開始前 27 日以内に血球成分除去療法をした者
15. クロストリジウム・ディフィシル、サイトメガロウイルス又はその他の腸病原体に治験薬投与開始前 27 日以内に感染した者
16. 治験薬投与開始時に治療を要する大腸腺腫性ポリープがある者
17. 大腸の dysplasia の既往又は合併がある者
18. UC 以外の腸炎の疑いがある者
19. スクリーニング検査で HBs 抗原陽性又は HCV 抗体陽性の者。HBs 抗原陰性であっても HBc 抗体又は HBs 抗体陽性の者。ただし、HBV ワクチン接種による HBs 抗体単独陽性の者、HBV-DNA 陰性の者、HCV 抗原陰性又は HCV-RNA 陰性の者は除く
20. 結核の既往又はその疑いがある者（スクリーニング検査時の胸部画像検査で結核の既往を示す所見が認められる者を含む）。ただし、過去にイソニアジドの予防投与を完了した者、又は治験薬投与開始 21 日以上前からイソニアジドの予防投与を行っている者は除く（後者の場合、スクリーニング期間を最長 28 日間まで延長し、21 日間以上の予防投与を行った後に治験薬投与を開始してもよい）
21. スクリーニング検査でT-SPOT又はクォンティフェロン検査陽性の者
22. 先天性又は後天性免疫不全症候群（分類不能な免疫不全症、HIV 感染、

臓器移植等）の既往又は合併がある者

23. 治験薬投与開始前 27 日以内に重篤な腸管外感染症（肺炎、敗血症、活動性肝炎、腎盂腎炎等）を罹患した者
24. MLN0002 の投与歴がある者
25. スクリーニング期間中に授乳中の女性、スクリーニング検査時又は治験薬投与開始前のいずれかの尿妊娠検査が陽性である女性
26. 重篤な心疾患系、肺、肝、腎、代謝性、消化器系、泌尿器系、内分泌系又は血液の疾患の合併がある者
27. 治験薬投与開始前 27 日以内に全身麻酔を要する手術を受けた、又は治験期間中に入院を伴う手術を受ける予定がある者
28. 悪性腫瘍の合併又は既往がある者。ただし、以下の場合は除く：
    - 限局性皮膚基底細胞癌の治癒切除又は放射線根治療法を受けた場合
    - 皮膚扁平上皮癌の治癒切除又は放射線根治療法を受け、治療後、再発なく 1 年以上経過している場合
    - 子宮頚部上皮内癌の治癒切除又は放射線根治療法を受け、治療後、再発なく 3 年以上経過している場合

    かなり以前の悪性腫瘍の既往（治療後、再発なく 10 年以上経過等）がある患者の場合は、悪性腫瘍の種類及び受けた治療の性質に基づき、治験責任医師と治験依頼者が協議し、適格性を判断する
29. 脳卒中、多発性硬化症、脳腫瘍、神経変性疾患等の中枢神経障害の既往又は合併がある者
30. スクリーニング検査又は治験薬投与開始前の PML 自覚症状チェックリストにおいて、何らかの自覚症状が認められた者
31. スクリーニング検査において、以下のいずれかの臨床検査値異常が認められた者：
    - ヘモグロビン 8 g/dL 以下
    - 白血球数 3,000/µL 以下
    - リンパ球数 500/µL 以下
    - 血小板数 10 万/µL 以下、又は 120 万/µL 以上
    - ALT 又は AST 基準値上限の 3 倍以上
    - ALP 基準値上限の 3 倍以上
    - クレアチニン 基準値上限の 2 倍以上
32. 治験薬投与開始前 1 年以内に、アルコール依存症又は不正薬剤使用の既往又は合併がある者
33. 治験手順の遵守を妨げるおそれのある精神病性障害の既往又は合併がある者

7.3 併用禁止薬及び併用禁止治療

併用禁止薬及び併用禁止治療の一覧表を表 7.a に示す。なお、そのうちの一部の薬剤については条件付での併用を可とし、使用条件を表中に示す。治験責任医師又は治験分担医師は、被験者に対して服薬を指示した薬剤以外の市販薬を含むいかなる薬剤も、事前の相談なしに服薬しないよう指示する。

表 7.a 併用禁止薬及び併用禁止治療の一覧表

薬剤／治療の種類	併用禁止期間 注 1)	併用する場合の条件	
		スクリーニング期／導入期／維持期	非盲検コホート
5-ASA 経口剤<br>プロバイオティクス 注 2)	Day-13 ～ 治験薬最終投与 16 週後	Day-14 以前から使用し、かつ Day-13 から投与量不変で継続すること。なお、副作用のための減量又は中止のみ許容する。	使用可<br>（制限なし）
ステロイド経口剤<br>（30 mg/日以下）	Day-13 ～ 治験薬最終投与 16 週後	Day-14 以前から使用し、かつ Day-13 から投与量不変で継続すること。ステロイドの漸減投与計画（7.3.2 項参照）に従うこと。	同左
5-ASA 注腸剤<br>5-ASA 坐剤	Day-13 ～ 治験薬最終投与 16 週後	－	使用可<br>（制限なし）
ステロイド注腸剤<br>ステロイド坐剤<br>（内視鏡検査前のステロイド散布を含む）	Day-13 ～ 治験薬最終投与 16 週後	－	使用可<br>（制限なし）
ステロイド静注剤	Day-13 ～ 治験薬最終投与 16 週後	－	－
ステロイド経口剤<br>（30 mg/日超）	Day-13 ～ 治験薬最終投与 16 週後	－	－
下痢型過敏性腸症候群治療薬	Day-13 ～ 治験薬最終投与 16 週後	－	－
UC 治療を目的とした漢方薬<br>（大建中湯等）	Day-13 ～ 治験薬最終投与 16 週後	－	－
止瀉薬	Day-13 ～ 治験薬最終投与 16 週後	Mayo スコア評価日前 7 日以内は使用しないこと。4 日以上連続で使用しないこと。	使用可<br>（制限なし）
AZA<br>6-MP	Day-27 ～ 治験薬最終投与 16 週後	Day-83 以前から使用し、かつ Day-27 からは投与量不変で継続すること。なお、副作用のための減量又は中止のみ許容する。	同左
タクロリムス<br>シクロスポリン<br>メトトレキサート<br>トファシチニブ	Day-27 ～ 治験薬最終投与 16 週後	－	－
UC 治療を目的とした低分子化合物の治験薬	Day-27 ～ 治験薬最終投与 16 週後	－	－
アダリムマブ	Day-27 ～ 治験薬最終投与 16 週後	－	－
ナタリズマブ<br>エファリズマブ<br>リツキシマブ<br>MLN0002	(過去) ～ 治験薬最終投与 16 週後	－	－
上記以外の生物学的製剤<br>（インフリキシマブ等。局所投与は除く）	Day-55 ～ 治験薬最終投与 16 週後	－	－
生ワクチン	Day-27 ～ 治験薬最終投与 6 ヵ月後	－	－

薬剤／治療の種類	併用禁止期間 注 1)	併用する場合の条件	
		スクリーニング期／導入期／維持期	非盲検コホート
腸管切除（治験期間中に発現した膿瘍に対する排膿、肛門部病変に対するシートン法等腸管切除以外の外科的処置は実施可）	Day-27 ～ 治験薬最終投与 16 週後	－	－
血球成分除去療法	Day-27 ～ 治験薬最終投与 16 週後	－	－
全身麻酔を要する手術	Day-27 ～ 治験薬最終投与 16 週後	－	－

注 1) 治験薬初回投与日を Day 1、その前日を Day-1 として起算する。
注 2) VSL#3 等。ビフィズス菌製剤及び食品は該当しない。

7.3.1 救済治療

表 7.a で併用が認められていない薬剤又は治療は、UC の悪化等で被験者の安全性が担保できないと判断された場合のみ、救済治療（定義は 3.1 項参照）として緊急的に使用できる。

導入期において救済治療を実施したときは治験を中止する。維持期において救済治療を実施したときは維持期を中止し、非盲検コホートへの移行を可とするが、非盲検コホートで使用不可の治療を希望する場合は、治験中止を検討する。非盲検コホートにおいて救済治療を実施したときは、治験中止を検討する（7.5 項参照）。

7.3.2 ステロイドの漸減投与計画

ステロイドを併用する場合は、原則として、治験薬投与開始後 10 週目まで用量を変更しない。10 週目又はそれ以降の時点で改善（定義は 3.1 項参照）が認められた場合、可能な限り、改善が認められた来院日から、以下のスケジュールに従いステロイドの漸減を開始する。

漸減スケジュール：

- 10 mg/日超の場合、10 mg/日に達するまで 5～10 mg/2 週の割合で漸減
- 10 mg/日以下の場合、又は漸減により 10 mg/日以下に達した場合、ステロイドフリーに達するまで 2.5～5 mg/2 週の割合で漸減

ステロイドの副作用のため、10 週目よりも早期に漸減を開始する必要がある場合は、5 mg/2 週を超えない緩徐な漸減にとどめる。

ステロイドを漸減したときに症状の再燃が認められる場合は、治験薬投与開始時の用量まで戻してもよい（救済治療には該当しない）。その後、再度改善が認められた場合、可能な限り、改善が認められた来院日から速やかに上記スケジュールに従い漸減を開始する。

なお、症状の再燃に対し、治験薬投与開始時の用量を超えて投与を行った場合は救済治療とみなす（救済治療を実施した場合の対応は 7.3.1 項参照）。

7.4 被験者の管理

1. 来院日時を守り、医師の診察や定められた検査を必ず受けるように被験者に指示すること。また、来院できないときは速やかに治験責任医師、治験分担医師又は治験協力者に連絡するように被験者に指示すること。
2. 同意取得時から治験薬最終投与までの期間を通して、患者日誌を記録し、来院ごとに持参するように被験者に指示すること。
3. 血液生化学検査を実施する VISIT は、可能な限り絶食下で来院するように被験者に指示すること。
4. 予定来院日以外に症状の悪化が見られた場合には速やかに治験責任医師、治験分担医師又は治験協力者に電話等で報告し、指示を仰ぐように被験者に指示すること。
5. 自覚症状・他覚所見について、その内容、発現日、程度、転帰及び転帰日を来院の際に治験責任医師又は治験分担医師に報告するように被験者に指示すること。
6. 同意取得時から治験薬最終投与 16 週後までの間に被験者が他の医療機関を受診した場合は、その経緯及び治療内容について治験責任医師又は治験分担医師に報告するように被験者に指示すること。
7. 同意取得時から治験薬最終投与 16 週後までの間に被験者が他の医療機関を受診する場合は、本治験に参加していることをその医療機関の医師に伝達するように被験者に指示すること。
8. 治験責任医師又は治験分担医師が服薬を指示した薬剤以外の市販薬を含むいかなる薬剤も、事前の相談なしに服薬しないように被験者に指示すること（ただし、緊急の場合を除く）。
9. 妊娠する可能性のある女性パートナーを持つ、避妊治療を受けていない男性被験者については、同意取得時から治験薬最終投与 6 ヵ月後まで、適切な避妊を行うよう被験者に指示すること。
10. 避妊治療を受けていない男性パートナーを持つ、妊娠する可能性のある女性被験者については、同意取得時から治験薬最終投与 6 ヵ月後まで、適切な避妊を行うよう被験者に指示すること。

11. 同意取得時から治験薬最終投与までの期間を通して、暴飲暴食、極端な食事内容の変更、過度の運動又は行為をしないように被験者に指示すること。治験期間中に症状が変化した場合、食事内容が変化することがあるため、特に注意して指導すること。
12. 同意取得時から治験薬最終投与 6 ヵ月後まで、献血を行わないよう被験者に指示すること。
13. 治験薬注入に伴う反応（発疹、そう痒症、潮紅、蕁麻疹等）を感じたら直ちに治験責任医師又は治験分担医師に報告するように被験者に指示すること。

7.5 被験者ごとの中止基準

治験責任医師又は治験分担医師は、治験中止の主な理由を以下の分類に従って症例報告書に記録する。導入期への組み入れ前に中止した症例については、9.1.22 項参照。

1. **Pretreatment Event 又は有害事象**
   治験の継続により被験者の健康への容認できないリスクを避けるため、早期の中止が必要な Pretreatment Event 又は有害事象が発現した場合、若しくは被験者が Pretreatment Event 又は有害事象により治験の継続を望まない場合。

- 肝機能検査値異常

  治験薬投与中に以下のいずれかの基準を満たす肝機能検査異常が発現した場合には、治験薬の投与中止を検討し、被験者の臨床検査プロファイルが正常若しくは同意取得後の最初の検査値（Pretreatment Event の場合）あるいは治験薬投与前値（有害事象の場合）に回復するまで、適切な追跡検査を実施する（9.1.12 項参照）。

  – ALT 又は AST が基準値上限の 8 倍を超えた場合
  – ALT 又は AST が基準値上限の 5 倍を超え、かつ 2 週間以上継続した場合
  – ALT 又は AST が基準値上限の 3 倍を超え、かつ総ビリルビン値が基準値上限の 2 倍を超えた又は国際標準比（INR）が 1.5 を超えた場合
  – ALT 又は AST が基準値上限の 3 倍を超え、かつ疲労、悪心、嘔吐、右上腹部痛若しくは圧痛、発熱、発疹又は好酸球増加症が認められた場合

2. **治験実施計画書からの重大な逸脱**

導入期への組み入れ後に、被験者が治験実施計画書の組み入れ基準を満たさないことが判明したこと又は治験実施計画書を遵守しなかったことにより、治験の継続により被験者の健康へ容認できないリスクを与える場合。

注：導入期有効性主要評価（VISIT 5）の来院日について、許容範囲を著しく超える逸脱（±15 日以上）の場合は、治験実施計画書からの重大な逸脱とする。

3. **追跡不能**

被験者が来院せず、連絡も取れなかった場合。この場合は連絡を取ろうとしたことを原資料に記録する。

4. **自発的な中止**

被験者が、治験への参加中止を希望する場合。中止の理由が聴取できた場合には、症例報告書に記録する。

注：自発的な中止に関する理由を可能な限り明らかにするよう試みる（有害事象又は有効性の欠如による中止は「自発的な中止」には分類しない）。

5. **治験全体の中止**

治験依頼者、治験審査委員会又は規制当局が治験を中止するよう決定した場合。

6. **妊娠**

女性被験者の妊娠が判明した場合。

注：被験者の妊娠が判明した場合には、直ちに治験への参加を中止する。その際の手順は、9.1.14 項参照。

7. **有効性の欠如**

治験責任医師又は治験分担医師が、治験薬の有効性が認められず、治験の継続によって、被験者に容認できないリスクを及ぼすと判断した場合。

注：導入期に救済治療を実施した場合は、有効性の欠如により、直ちに治験への参加を中止する。非盲検コホートにおいて救済治療を実施した場合、非改善状態の持続又は疾患悪化に該当した場合は、有効性の欠如による中止を検討する。

8. **その他**

注：その詳細を症例報告書に記録する。

7.6 被験者の治験中止の手続き

治験責任医師又は治験分担医師は、被験者が 7.5 項に記載する基準に抵触した場合には、当該被験者の治験への参加を中止する。また、被験者は治験実施中のいつでもその理由を説明することなく治験への参加を中止することができる。治験責任医師又は治験分担医師は、被験者の治験参加が中止される場合には、その主な理由を症例報告書に記録する。その際は、中止時に実施すべきすべての検査・観察・評価を可能な限り行う。

8.0 薬剤及び補助器材管理

本項では、臨床試験の薬剤及び補助器材管理を説明した重要な項を示す。治験実施計画書で規定の、治験依頼者が直接提供及び／又は他の手段で調達するすべての薬剤及び補助器材に関する情報を記載する。

8.1 薬剤及び補助器材

8.1.1 剤型、製造、包装及び表示

8.1.1.1 治験薬

本治験実施計画書では、治験薬という用語は、以下に定義する薬剤のことをいう。

表 8.a 治験薬

治験薬名	有効成分の化学名<br>（一般名）	剤型	含量
MLN0002<br>300 mg 注	ヒト$\alpha_4\beta_7$インテグリンに対する<br>ヒト化IgG_1モノクローナル抗体<br>（vedolizumab (INN)）	注射剤	1 バイアル中に<br>MLN0002 として<br>300 mg*を含有
プラセボ注	なし	－	空バイアル

*凍結乾燥製剤

治験薬バイアルの外観は同一である。1 バイアルずつラベルを貼付して外箱に収める。外箱には治験薬である旨の表記、治験薬名、数量、治験依頼者名及び住所、製造番号、貯法、使用期限を表示する。バイアル及び外箱のラベルの表示例をそれぞれ図 8.a 及び図 8.b に示した。なお、治験薬の製造番号、使用期限は、別途定める手順書に従う。

MLN0002 300 mg 注バイアル用	プラセボ注バイアル用
MLN0002/CCT-101<br>MLN0002 300 mg 注<br>製造番号：Z ●●●●	MLN0002/CCT-101<br><u>本バイアルは被験者に</u><br><u>投与しないこと</u>

図 8.a バイアルのラベル

治験薬　1ﾊﾞｲｱﾙ<br>MLN0002　DB 注<br>MLN0002/CCT-101<br><br>薬剤番号：<br><br>被験者識別コード：<br><br>投与時点：<br><br>週目	製造番号：DB ●●●●<br>使用期限：治験薬管理手順書に記載<br>貯　　法：冷蔵（2-8℃）<br><br>注意<br>空箱は治験依頼者が回収致しますので、それまで保管して下さい<br><br>武田薬品工業株式会社<br>大阪市中央区道修町四丁目 1 番 1 号

図 8.b　　外箱のラベル

8.1.1.2　補助器材

治験薬の投与に用いる補助器材として、原則として、以下のものを使用する。なお、補助器材の管理は、別途定める手順書に従う。

表 8.b　　補助器材

使用目的	補助器材	例示
治験薬調製	調製用シリンジ	ラテックスフリーの 5 cc 又は 10 cc シリンジ
	注射用水	容量 10 mL 以上の容器入り注射用水
	調製用注射針	18G のステンレス鋼注射針
	生理食塩液	100 mL 規格の滅菌 0.9% NaCl 水溶液
治験薬投与	点滴静注セット	注射部位を点滴筒直下とする静脈内注入セット
	投与用注射針	静注用翼状針
	生理食塩液（静注ラインのプライミング／フラッシング用）	50 mL、100 mL 又は 250 mL 規格の滅菌 0.9%NaCl 水溶液

8.1.2　貯法

冷蔵（2～8℃）で保管する。ただし、-70～40℃までの一時的な逸脱（7 日間）を許容する。

治験薬はそれを使用するまで、あるいは治験依頼者又はその指名する者に返却するまで、立ち入りが制限された適切かつ安全な場所に保管されなければならない。治験依頼者が提供する治験薬はすべて、ラベル表示に記載の条件下で保管し、調剤するまでは元の容器に入れた状態としなければならない。治験薬保管場所の日単位の温度を確認し、その結果を毎就業日記録しなければならない。

8.1.3 投与量及び投与方法

表 8.c 投与量及び投与方法

治験薬	治験薬調製及び投与方法
プラセボ注	注射用水 5 mLを約 100 mLの生理食塩液に加え、30 分*かけて点滴静注する
MLN0002 300 mg注	MLN0002 300 mg注 1 バイアルあたり注射用水 4.8 mLで溶解し、5.0 mLを約 100 mLの生理食塩液に加えて希釈し、30 分*かけて点滴静注する

*最大 60 分まで許容する。

治験薬の調製は、治験薬調製担当者が実施する。治験薬調製担当者は、導入期及び維持期を通して評価を実施しない者とする。治験薬調製担当者は、治験責任医師又はその指名する者が処方した薬剤番号の治験薬を調製する。治験薬の詳細な調製方法については、別途定める手順書に従う。

重篤な注入に伴う反応が生じたときは、エピネフリン及び非経ロジフェンヒドラミンを直ちに使用する等適切な処置を行う。

8.1.4 過量投与

過量投与とは、判明している故意又は過失により治験薬が治験実施計画書に規定される量を超えて投与されることと定義する。

過量投与に関する重要な安全性情報をデータベースに一貫して入力するため、治験責任医師又は治験分担医師は、過量投与のすべての事例（有害事象の有無に係わらず）を症例報告書の過量投与の頁に記録する。過量投与に伴う有害事象は、10.0 PRETREATMENT EVENT 及び有害事象の項に従って、症例報告書の有害事象の頁に記録する。

また、過量投与に伴う重篤な有害事象については、10.2.2 重篤な有害事象の収集及び報告の項に記載された手順に従って報告する。

治験薬が過量投与された場合には、治験責任医師又は治験分担医師は症状に基づいて治療する。

8.2 治験薬の割付と処方及び投与手順

投与ごとに使用する治験薬の薬剤番号が異なる。治験責任医師又はその指名する者は、被験者に治験薬を割り付けるため、投与VISITごとに登録センターのウェブサイトにアクセスし、登録センターから 治験薬の薬剤番号を入手する。治験責任医師又はその指名する者は、入手した薬剤番号に基づき治験薬を処方する。また、投与VISITごとに薬剤番号を症例報告書に記録する。

登録センターのウェブサイトの利用方法の詳細については、別途定める手順書に従う。

8.3 割付表作成と保管

割付責任者（治験依頼者の指名する者）が割付表を作成する。割付の情報は安全な場所に保管され、権限を持つ者以外が入手できないようにする。

8.4 盲検性の維持

エマージェンシー・キーは、エマージェンシー・キー管理センターが緊急開封時又は全症例のデータ固定時まで保管する。

本治験では、治験薬調製過程において、治験薬調製担当者は割付内容を知り得るが、割付内容を治験薬割付表の開封まで他者に開示してはならない。

また、本治験では血清中薬物濃度の測定結果、HAHA・中和抗体の測定結果により、盲検性の維持に支障をきたす可能性があることから、測定実施機関はこれらの最終測定結果を治験薬割付表の開封まで外部に開示することなく保管し、開封後に治験依頼者を通じて治験責任医師に報告する。ただし、開封前に測定結果を開示する場合は、測定実施機関の担当者が被験者を特定できないように、薬剤番号を付け替える等、盲検性維持のための処置を施し、治験依頼者等へ報告することができるものとする。手順の詳細は、別途作成する手順書に定める。

8.5 盲検解除の手順

治験責任医師又は治験分担医師は、被験者に対する医療処置のために治験薬に関する情報が必要な場合を除き、治験薬の盲検を解除しない。

治験責任医師又は治験分担医師は盲検を解除する際、治験薬の割付情報はエマージェンシー・キー管理センター（別紙の連絡先参照）に連絡を取ることによって取得することができる。

治験責任医師又は治験分担医師は、治験薬の盲検を解除する場合には、盲検を解除した日時、理由をエマージェンシー・キー開封の記録に記載し、治験依頼者に提出する。同じ情報（時刻以外）を症例報告書にも記録する。

盲検を解除する場合には、治験責任医師又は治験分担医師は、治験薬投与を直ちに中止するとともに、当該被験者の治験への参加を中止する。

8.6 治験依頼者が提供する薬剤の管理及び廃棄

治験薬管理者は、治験依頼者が作成した治験薬の取扱い及び保管・管理に関する手順書を受領し、その手順書に従って治験依頼者が提供する治験薬及び補助

器材を適切に管理する。治験責任医師も同様に、治験依頼者から当該手順書を受領する。当該手順書には、治験依頼者が提供する治験薬及び補助器材の受領、取扱い、保管、管理、処方及び未使用治験薬及び補助器材の治験依頼者への返却又はその他の処分が、適切で確実に行われるために必要な手順が記載されている。

治験薬管理者は、当該治験実施医療機関での治験終了後速やかに未使用治験薬及び補助器材を治験依頼者に返却する。

9.0 治験実施計画

9.1 治験実施手順

治験責任医師又は治験分担医師は、以下の手順に従ってデータを収集する。被験者の検査・観察・評価は、原則として同一の治験責任医師又は治験分担医師が行う。治験スケジュールは付録 A に示す。

9.1.1 同意取得

同意取得の方法については、15.2 項に示す。

治験手順が行われる前に被験者の同意を取得する。

治験への組み入れのために前治療薬の投与量変更及び投与中止を行う場合には、治験参加の同意を取得した後に実施する。

同意取得のための説明時に各被験者に被験者識別コードを付与する。この被験者識別コードは、治験期間を通して使用される。

ファーマコゲノミクスのための同意取得

本治験のファーマコゲノミクス検討用血液検体を収集する前に、検体の保管及びバンキングに関する同意を別途取得しなければならない。ファーマコゲノミクス検討用検体の収集及び解析に対する同意は、本治験の内容に対する同意とは別のものである。

9.1.2 人口統計学的データ、既往歴及び前治療薬

人口統計学的データとして、生年月日、性別及び喫煙歴を調査する。また、UC に関する以下の項目を調査する。

- 発症時期
- 「難治性炎症性腸管障害に関する調査研究」班の潰瘍性大腸炎診断基準改訂版（平成 24 年度）（付録 C）に定義される以下の項目
  - UC 病変の広がりによる病型分類（全大腸炎／左側大腸炎）
- 同意取得前 5 年以内の以下の薬剤の治療失敗歴の分類
  - ステロイド（抵抗性／依存性／不耐性）
  - 免疫調節薬（AZA 又は 6-MP）（不応／不耐性）
  - 抗 TNFα 抗体*（一次無効／二次無効／不耐性）

* 一次無効及び二次無効については、使用薬剤名、投与開始日及び投与終了日を併せて記録する。不耐性については、使用薬剤名及び投与中止の原因となった副作用を併せて記録する。

既往歴としては、同意取得から過去 1 年間に消失又は終了した、腸管外合併症、回腸嚢炎、感染性腸炎、中毒性巨大結腸症、狭窄、瘻孔を含む、臨床的に問題となる疾患又は症状で、医師が、本治験における評価に影響を与えるため既往歴とすべきと判断したものを調査する。また、過去すべての悪性腫瘍、UC に対する腸管切除を調査する。症状又は疾患が継続している場合は、合併症とする（9.1.7 項参照）。

前治療薬としては、以下の薬剤をすべて調査する。

- 同意取得時までに使用を終了した UC 治療のための生物学的製剤
- 同意取得 4 週間前から同意取得時までに使用を終了した UC 治療のための低分子化合物の薬剤

なお、内視鏡検査等のための検査処置薬は、前治療薬に該当しないこととし、症例報告書への記載も必要としない。

9.1.3 診察

以下の身体部位について、診察を行う。治験薬投与日の場合は、治験薬投与前に実施する。
(1)眼、(2)耳鼻咽喉、(3)心血管、(4)呼吸器、(5)消化器、(6)皮膚、(7)四肢、(8)筋骨格、(9)神経系、(10)リンパ節、(11)その他。治験薬注入中及び注入 1 時間後まで、注入に伴う反応（発疹、そう痒症、潮紅、蕁麻疹等）を確認するとともに、治験薬投与前の診察結果と比較して臨床的に問題と考えられる異常の有無を評価する。

9.1.4 体重、身長及び BMI

体重及び身長を測定する。治験薬投与日の場合は、治験薬投与前に実施する。
また、BMI は治験依頼者において以下の数式を用いて算出する。

評価指数： BMI＝体重（kg）／（身長（m））^{2}

身長は整数のみのセンチメートルを単位として測定し、体重は小数点第 1 位までのキログラムを単位として測定する。BMI の結果は小数点第 1 位まで示す。

9.1.5 バイタルサイン

バイタルサインとして、体温（腋窩）、坐位の血圧（5 分間以上安静）及び脈拍（bpm）を測定する。治験薬投与日の場合は、治験薬投与前に実施する。

9.1.6 併用薬及び併用療法

併用薬とは、治験薬に加えて投与されるすべての薬剤のことである。医師が処方した薬剤又は被験者が購入した市販薬を含む。併用薬には治験依頼者が提供する薬剤は含まれない。被験者の来院の度に、同意取得時から終了時検査時までの間に使用した治験薬以外の薬剤（ビタミン剤、市販薬、漢方薬を含む）の使用状況を聴取する。また、UC 治療を目的とした併用薬の場合は、併用薬の分類（5-ASA 製剤、ステロイド、免疫調節薬、その他の 4 分類）、併用薬が救済治療に該当するかどうかを症例報告書に記録する。

併用療法とは、UC に関する治療のうち、薬物治療を除くすべての治療のことである。腸管切除及びその他の外科的治療（膿瘍に対する排膿、肛門部病変に対するシートン法等）を含む。同意取得時から終了時検査時までの間に実施したすべての併用療法を調査する。また、併用療法が救済治療に該当するかどうかを症例報告書に記録する。

なお、内視鏡検査等のための検査処置薬は、併用薬に該当しないこととし、症例報告書への記載も必要としない。

9.1.7 合併症

合併症とは、同意取得時に有する症状又は疾患のことをいう。同意取得後最初の検査及び診察で認められた臨床的に問題となる臨床検査値、心電図所見、診察所見の異常は治験責任医師又は治験分担医師の判断により合併症とする。合併症の内容（診断名）を調査する。また、UC の腸管外合併症の有無を症例報告書に記録する。

9.1.8 患者日誌

患者日誌は Mayo スコアの算出に使用する。来院ごとに、今回来院時から次回来院時までの患者日誌を被験者に交付する。以下の項目を記録し、次回来院時に持参するよう指導する。

- 排便回数
- 排便時の出血スコア
- 止瀉薬の使用有無

患者日誌は来院ごとに記載内容を確認し、記載漏れ・不整合等があれば、当該被験者に対して問い合わせを行い、記載内容を整備する。記載内容を最終確認した後、患者日誌を回収し保管する。

9.1.9 完全 Mayo スコアと部分 Mayo スコア

完全 Mayo スコアは、**表** 9.a に示すように、以下の 4 項目のサブスコアの合計値から算出される。部分 Mayo スコアは、粘膜所見を除く 3 項目のサブスコアの合計値から算出される。

表 9.a Mayoスコア 13)

排便回数<br>0=正常回数<br>1=正常回数より 1～2 回/日多い<br>2=正常回数より 3～4 回/日多い<br>3=正常回数より 5 回/日以上多い
血便<br>0=なし<br>1=排便時の半分以下でわずかに血液が付着する<br>2=ほとんどの排便時に明らかな血液の混入<br>3=大部分が血液
粘膜所見<br>0=正常又は非活動性所見<br>1=軽症（発赤、血管透見像の減少、軽度脆弱）<br>2=中等症（著明に発赤、血管透見像の消失、脆弱、びらん）<br>3=重症（自然出血、潰瘍）
医師の全般的評価<br>0=正常<br>1=軽症<br>2=中等症<br>3=重症

スクリーニング検査時（VISIT 1）では、部分 Mayo スコアを算出するため、治験責任医師又は治験分担医師が、来院前 3 日間の平均的な排便回数、血便の状況、全身症状について問診を行い、聴取結果に基づいて各サブスコアを評価する。排便回数、血便及び医師の全般的評価の各サブスコアを登録センターのウェブサイトに入力し、部分 Mayo スコアを算出する。

治験薬投与開始時（VISIT 2）以降では、完全 Mayo スコア又は部分 Mayo スコアを算出するために必要な患者日誌データ、粘膜所見サブスコア及び医師の全般的評価のサブスコアを登録センターのウェブサイトに入力する。このときの各サブスコアの算出方法を次に示す。

9.1.9.1 排便回数及び血便スコアの算出

排便回数及び血便スコアを算出するため、評価日前 7 日間の患者日誌の記載内容を登録センターのウェブサイトに入力する。各サブスコアは以下を除く評価日直近 3 日分の患者日誌の内容に基づき自動的に算出される。

- 内視鏡検査のための前処置日
- 内視鏡検査日及びその翌日
- 止瀉薬が使用されていた場合、止瀉薬の使用日及びその翌日

なお、ベースライン評価（VISIT 2）では、完全 Mayo スコアを算出するため、少なくとも、上記の除外日を除く 3 日分の患者日誌を登録センターのウェブサイトに入力する必要がある。患者日誌の必要日数を満たすため、被験者の来院日を調整する。

9.1.9.2 粘膜所見スコアの算出

完全 Mayo スコア評価日の 9 日前から評価当日までに直腸及び S 状結腸の内視鏡検査を実施する。治験責任医師又は治験分担医師は、ベースライン時に最も活動性が高い部位を特定し、治験期間を通して同一部位について、表 9.a に基づき粘膜所見のスコアリングを行う。粘膜所見スコアの判定は可能な限り、治験期間を通して同一の医師が実施する。評価した粘膜所見スコアを登録センターのウェブサイトに入力する。

また、本治験では中央判定委員会（11.2 項参照）を設置し、独立して粘膜所見スコアの判定を行う。そのため、治験責任医師又は治験分担医師は、中央判定委員会で粘膜所見スコアの判定ができる鮮明な画像を治験依頼者に提出する。

なお、ベースライン評価（VISIT 2）では、検査の侵襲性を考慮し、同意取得前に実施された評価日前 9 日以内の内視鏡検査結果がある場合は、その利用を可とする。

9.1.9.3 医師の全般的評価スコアの算出

治験責任医師又は治験分担医師が、評価日に問診（腹部不快感、全身状態、主治医所見及び被験者の印象等）を行い、それらの結果と排便回数、血便及び粘膜所見（実施した場合）を参考に評価する。

9.1.10 炎症性腸疾患質問票（IBDQ）

IBDQ は腹部症状（10 項目）、全身症状（5 項目）、情緒（12 項目）、社会機能（5 項目）の計 32 項目（各項目 1～7 点で評価）で構成され、全項目の総和を IBDQ スコアとする。治験薬投与日の場合は、治験薬投与前に実施する。各項目のスコアを症例報告書に記録する。

9.1.11　UC 関連イベント

治験薬初回投与から治験終了時までに発生した UC 関連イベントの有無を症例報告書に記録する。イベントが発生した場合は、発生した年月日とともに、イベントの種類を次の分類で症例報告書に記録する。

- UC に関連する入院
- 腸管切除

9.1.12　臨床検査

表 9.b に示す臨床検査項目を測定する。治験薬投与日の場合は、治験薬投与前に検体を採取する。検体は、別途定めた手順に基づき取り扱う。各 VISIT での最大採血量は、約 19 mL である。

表 9.b　　　臨床検査

**血液生化学検査**			
アルブミン		総コレステロール	
アスパラギン酸アミノトランスフェラーゼ（AST）		中性脂肪 注1）	
アラニンアミノトランスフェラーゼ（ALT）		クレアチニン	
アルカリホスファターゼ（ALP）		血中尿素窒素（BUN）	
アミラーゼ		尿酸	
グルコース 注1）		カリウム	
総ビリルビン		ナトリウム	
総蛋白		カルシウム	
ガンマグルタミルトランスペプチダーゼ（γ-GTP）		リン	
		マグネシウム	
		クロール	
**血液学的検査**		**尿検査**	
赤血球数	好中球	定性（糖、蛋白、潜血、ビリルビン、ケトン体）	
白血球数	好酸球	尿 pH	
ヘモグロビン量	好塩基球	尿比重	
ヘマトクリット値	リンパ球		
血小板数	単球		
**血清免疫学的検査（スクリーニング検査時のみ）注2）**		**尿妊娠検査（妊娠する可能性のある女性被験者のみ）**	
HBs 抗原、HBs 抗体、HBc 抗体、HCV 抗体		ヒト絨毛性性腺刺激ホルモン（hCG）	
HIV 抗体			
**炎症性マーカー検査**			
C 反応性蛋白（CRP）			

注 1）　可能な限り絶食下で検体採取する。
注 2）　血清免疫学的検査の検査結果は、治験責任医師又は治験分担医師が被験者に直接報告し、治験依頼者は入手しないこととする。

臨床検査測定機関において、尿妊娠検査を除く上記の臨床検査を実施する。また、治験実施医療機関において、尿妊娠検査を実施する。治験責任医師又は治験分担医師は、報告された臨床検査結果を評価するとともに、保存する。

ALT 又は AST が基準値上限の 3 倍を超えた場合には、再検査（少なくとも、ALP、ALT、AST、総ビリルビン、γ-GTP、INR）を採血後 48～72 時間以内、遅くとも 7 日以内には実施する（7.5 項及び 10.2.3 項参照）。

2 回連続して ALT 又は AST が基準値上限の 3 倍を超えた場合には、治験責任医師又は治験分担医師は、治験依頼者に連絡し、追加検査、詳細なモニタリング、治験薬投与中止、被験者の詳細情報、治験薬以外の他要因について協議する。

治験責任医師は、臨床検査の基準値を履歴を含め保管する。

9.1.13 避妊

同意取得時から治験薬最終投与 6 ヵ月後まで、妊娠する可能性のある女性（避妊手術を受けていない者、又は最終月経から 2 年未満の者）は、適切な避妊を実施する。また、同意取得時から治験薬最終投与 6 ヵ月後まで、妊娠する可能性のある女性パートナーを持つ、避妊治療を受けていない男性は、避妊しなければならない。該当する被験者の同意取得にあたっては、同意・説明文書を用いて適切な避妊法及び治験中は妊娠を避ける必要がある旨を説明し、十分に理解を得たうえで同意を取得する。治験中は、定められた尿妊娠検査を行うとともに、被験者に対し、継続して避妊に関する指導を行う。

スクリーニング検査時及び治験薬初回投与直前の尿妊娠検査が陰性であることを確認する。

さらに、同意取得時から治験薬最終投与 6 ヵ月後まで、男性被験者が精子提供しないよう指導する。

9.1.14 妊娠

治験中に被験者が妊娠していることが判明した場合には、治験依頼者が提供した治験薬の投与を直ちに中止する。

治験薬初回投与（VISIT 2）以降又は治験薬最終投与後 6 ヵ月以内の妊娠については、所定の報告様式を用いて別紙の連絡先に報告する。さらに、男性被験者のパートナーが、VISIT 2 以降又は治験薬最終投与後 6 ヵ月以内に妊娠した場合には、被験者のパートナーから承諾を得たうえで、報告様式を用いて記録する。

盲検性が維持された治験薬の投与中又は投与後に妊娠した場合には、治験責任医師又は治験分担医師は、当該被験者に対し、治験薬の割付内容を知る権利が

あることを説明する。被験者が希望した場合には、治験責任医師又は治験分担医師がエマージェンシー・キーを開封する。治験期間中に MLN0002 を投与されていないことが判明した被験者は追跡調査する必要はない。

治験責任医師又は治験分担医師は、女性被験者及び／又は男性被験者の女性パートナーの同意のもとに、被験者／被験者の女性パートナーが妊娠したときに治験に参加していたこと及び治験薬の詳細（必要に応じて、盲検／非盲検について）を、被験者／被験者の女性パートナーの主治医（産婦人科医）に報告する。

治験責任医師又は治験分担医師は、妊娠が報告されたすべての被験者について、被験者の同意のもとに、早産等の結果も含め出産まで追跡調査し、所定の追跡調査の様式を使用して治験依頼者に報告する。出生後の評価も実施する。

9.1.15　心電図

12 誘導心電図を測定する。治験責任医師又は治験分担医師（又は各治験実施医療機関の専門医）は次の分類で判定する：正常範囲内、異常だが臨床的に問題なし、異常かつ臨床的に問題あり。

9.1.16　PML 評価

PML チェックリスト（付録 D）による評価を実施する。治験薬投与日の場合は、治験薬投与前に実施する。

まず、PML チェックリスト（自覚症状）を用いて、自覚症状の有無を確認する。何らかの自覚症状が認められる場合は、PML チェックリスト（他覚所見）を用いて、対応する他覚所見の有無を確認する。他覚所見でも異常が認められる場合は、PML チェックリストの症例評価アルゴリズムに従い、検査・観察・診察を行う。

PML チェックリストによる評価結果を症例報告書に記録する。PML チェックリストで何らかの自覚症状又は他覚所見が認められる場合、その症状については、有害事象としても収集する。

9.1.17　結核菌検査

結核菌検査用検体として全血 7 mLを採取する。検体は臨床検査測定機関が回収し、T-SPOTを用いた結核菌検査を実施する。医療機関において通常実施している場合は、クォンティフェロンを用いた結核菌検査の実施も許容する。検査結果に基づき、陽性、陰性、判定保留、判定不能を症例報告書に記録する。

9.1.18 胸部画像検査

胸部X線検査又は胸部CT検査を実施し、結核所見の有無を確認する。治験以外の一般診療で評価日前3ヵ月以内に撮影した検査画像がある場合は、それを利用してもよい。検査日及び結核所見の有無を症例報告書に記録する。

なお、スクリーニング検査では、同意取得前の検査画像を利用することも可とする。

9.1.19 ファーマコゲノミクス検討のための検体の収集

ファーマコゲノミクス検討について同意した被験者で、導入期に組み入れられた被験者について、薬物応答性を予測できるマーカーを探索的に検討するため、ファーマコゲノミクス検討のための検体をVISIT 2以降、治験終了時までの期間のうち、できる限り早い時期に1回、全血として5 mL採取する。

なお、同種骨髄移植を受けたことがある者についてはファーマコゲノミクス検討のための検体採取は行わない。

ファーマコゲノミクス検討のための検体の採取、取扱い及び保管に関しては別途作成される手順書を参照。

9.1.20 薬物動態評価のための検体の収集及び解析

薬物動態評価のための検体の収集は冷却遠心機のある治験実施医療機関でのみ実施する。これらの医療機関では、MLN0002の血清中濃度を測定するため、3 mLの静脈血を採取する。治験薬投与日の場合は、可能な限り投与直前に採取する。採血日時を症例報告書に記録する。

採取した血液は、室温にて約30分間静置後、4℃、3000 rpmで10分間遠心分離し、得られた血清を回収時まで-20℃以下に設定された冷凍庫で冷凍保存する。検体については、別途定めた手順に基づき取り扱う。

血清中のMLN0002濃度は、[redacted]にて、酵素結合免疫吸着法(ELISA)を用い測定する。なお、導入期及び維持期を通じてMLN0002が投与されなかった被験者の検体については測定しない。

9.1.21 HAHA及び中和抗体評価のための検体の収集

HAHA及び中和抗体評価のための検体の収集は冷却遠心機のある治験実施医療機関でのみ実施する。これらの医療機関では、HAHA及び中和抗体を測定するため、5 mLの静脈血を採取する。治験薬投与日の場合は、可能な限り治験薬投与直前に採取する。採血日時を症例報告書に記録する。

採取した血液は、室温にて約30分間静置後、4℃、3000 rpmで10分間遠心分離し、得られた血清を回収時まで-20℃以下に設定された冷凍庫で冷凍保存する。検体については、別途定めた手順に基づき取り扱う。

血清中のHAHAは、[redacted]にて、ELISAを用い測定する。なお、治験期間を通じてMLN0002が投与されなかった被験者の検体については測定しない。

また、HAHAが陽性であった検体のみ、血清中の中和抗体を測定する。血清中の中和抗体は、[redacted]にて、Cell based assay法を用い測定する。

9.1.22 導入期への組み入れ前に中止した症例の記録

同意書に署名し、導入期への組み入れ前に中止した全被験者について症例報告書を作成する。導入期への組み入れ前に中止した被験者の主な理由を次の分類で症例報告書に記録する。

- Pretreatment Event／有害事象
- 選択基準を満たさない又は除外基準に抵触＜理由を記載する。＞
- 治験実施計画書からの重大な逸脱
- 追跡不能
- 自発的な中止＜理由を記載する。＞
- 治験全体の中止
- 妊娠
- その他＜理由を記載する。＞

導入期への組み入れ前に中止した症例の被験者識別コードは再度使用しない。

9.1.23 導入期への組み入れ

すべての選択基準を満たし、除外基準のいずれにも抵触しない被験者のみを0週目（VISIT 2）に導入期へ組み入れる。導入期への組み入れの際、登録センターのウェブサイトに下記情報を登録し、0週目（VISIT 2）に投与する治験薬の薬剤番号の指示を受ける。

- 抗TNFα抗体前治療歴の有無
- 免疫調節薬（AZA又は6-MP）併用の有無
- ステロイド併用の有無
- 0週目（VISIT 2）における完全Mayoスコア算出のために必要な患者日誌データ、粘膜所見及び医師の全般的評価のサブスコア

9.1.24 導入期の有効性主要評価結果の登録／維持期への組み入れ

10 週目（VISIT 5）の完全Mayoスコア算出のために必要な患者日誌データ、粘膜所見及び医師の全般的評価のサブスコアを登録センターのウェブサイトに登録し、改善／非改善の判定結果を入手する。10 週目改善例のみを 14 週目（VISIT 6）に維持期へ組み入れる。維持期への組み入れの際、登録センターのウェブサイトにアクセスし、14 週目（VISIT 6）に投与する治験薬の薬剤番号の指示を受ける。

10 週目非改善例については、9.1.25 項参照。

9.1.25 非盲検コホートへの組み入れ

10 週目非改善例は、10 週目（VISIT 5）に非盲検コホートへの組み入れを可とする。また、維持期における疾患悪化例、救済治療例、60 週目完了例のいずれかに該当する場合も、非盲検コホートへの組み入れを可とする。なお、10 週目（VISIT 5）の検査前に中止した場合、又は 10 週目改善例が 14 週目（VISIT 6）の維持期組み入れ前に中止した場合は、非盲検コホートへの組み入れを不可とする。

非盲検コホートへの組み入れの手順は、以下のとおりである：

- 10 週目非改善例：
  10 週目非改善例については、10 週目（VISIT 5）の検査終了後、非盲検コホートに組み入れるかどうかを判断する。非盲検コホートに組み入れる場合、登録センターのウェブサイトにアクセスし、0x 週目（VISIT 1x）に投与する治験薬の薬剤番号の指示を受ける。

- 維持期における疾患悪化例・救済治療例：
  維持期における疾患悪化例・救済治療例については、維持期中止時検査終了後、非盲検コホートに組み入れるかどうかを判断する。ただし、維持期の最終投与から非盲検コホート組み入れまでは、3〜9 週間の間隔を空けることとする。非盲検コホートに組み入れる場合、登録センターのウェブサイトにアクセスし、0x 週目（VISIT 1x）に投与する治験薬の薬剤番号の指示を受ける。

- 60 週目完了例：
  60 週目完了例については、60 週目（VISIT 18）の検査終了後、非盲検コホートに組み入れるかどうかを判断する。非盲検コホートに組み入れる場合、登録センターのウェブサイトにアクセスし、0x 週目（VISIT 1x）に投与する治験薬の薬剤番号の指示を受ける。

9.2 被験者への投与状況

被験者に投与された治験薬について、投与日、投与開始時刻及び投与終了時刻を症例報告書に記録する。治験薬の注入が完了しなかった場合は、投与量及び完了できなかった理由を症例報告書に記録する。

9.3 検査・観察項目の実施時期

すべての検査・観察・評価のスケジュールを付録 A に示す。治験責任医師又は治験分担医師は、以下に記載する検査・観察・評価を規定された時点に実施する。

9.3.1 スクリーニング期（VISIT 1）

治験薬投与開始 21 日前～3 日前に診察・検査を実施する。7.0 項の選択・除外基準に基づき被験者の適格性を確認する。スクリーニング期に中止した被験者に関する記録の作成については、9.1.22 項を参照のこと。

スクリーニング期（VISIT 1）に実施する検査・観察・評価項目を以下に示す。

- 同意取得
- 人口統計学的データ、身長、既往歴、前治療薬、合併症
- 診察、バイタルサイン
- 体重
- 心電図
- 胸部画像検査*
- 結核菌検査
- PML チェックリスト
- 臨床検査（検査項目は付録 A 参照）
- 部分 Mayo スコア
- 併用薬及び併用療法
- Pretreatment Event の評価

* 3 ヵ月以内に撮影した検査画像が入手できる場合は実施しない（治験外の診療時及び／又は同意取得前の画像でも可）。

9.3.2 導入期

9.3.2.1 ベースライン評価／導入期への組み入れ（VISIT 2）

7.0 項の選択・除外基準に基づき被験者の適格性を確認する。すべての選択基準を満たし、除外基準に抵触しない被験者を 0 週目（VISIT 2）に 9.1.23 項に従い

導入期に組み入れる。導入期に組み入れできなかった場合の被験者の記録に関する手順は、9.1.22 項を参照のこと。

0 週目（VISIT 2）に実施する検査・観察・評価項目を以下に示す。

- 診察、バイタルサイン
- 体重
- 心電図
- PML チェックリスト
- 臨床検査（検査項目は付録 A 参照）
- 完全 Mayo スコア（直腸及び S 状結腸の内視鏡検査を含む。詳細は 9.1.9 項参照）
- 患者日誌の確認
- IBDQ
- HAHA・中和抗体測定用採血
- ファーマコゲノミクス検討用検体の採血（同意が得られた被験者のみ）
- 併用薬及び併用療法
- Pretreatment Event／有害事象の評価
- 治験薬薬剤番号の発番／投与

9.3.2.2 **導入期の投与**（VISIT 3 及び 4）

2 週目（VISIT 3）及び 6 週目（VISIT 4）に実施する検査・観察・評価項目を以下に示す。

- 診察、バイタルサイン
- 体重
- PML チェックリスト
- 臨床検査（検査項目は付録 A 参照）
- 部分 Mayo スコア
- 患者日誌の確認
- UC 関連イベント
- 血清中薬物濃度測定用採血
- 併用薬及び併用療法
- 有害事象の評価
- 治験薬薬剤番号の発番／投与

9.3.2.3 導入期有効性主要評価（VISIT 5）／導入期中止時

10 週目（VISIT 5）に実施する検査・観察・評価項目を以下に示す。導入期の途中で中止した場合も、可能な限り速やかに来院のうえ、同様の検査・観察・評価を実施する。

- 診察、バイタルサイン
- 体重
- 心電図
- 臨床検査（検査項目は付録 A 参照）
- 完全 Mayo スコア（直腸及び S 状結腸の内視鏡検査を含む。詳細は 9.1.9 項参照。導入期中止時は内視鏡検査を実施せず、部分 Mayo スコアでも可）
- 患者日誌の確認
- IBDQ
- UC 関連イベント
- HAHA・中和抗体測定用採血
- 血清中薬物濃度測定用採血
- 併用薬及び併用療法
- 有害事象の評価

10 週目（VISIT 5）における改善／非改善の判定結果を登録センターから入手する。改善例については、14 週目（VISIT 6）に維持期に組み入れる。非改善例については 10 週目（VISIT 5）に非盲検コホートへの組み入れを可とする。

9.3.3 維持期

9.3.3.1 維持期への組み入れ（VISIT 6）

10 週目（VISIT 5）改善例を 14 週目（VISIT 6）に 9.1.24 項に従い維持期に組み入れる。

14 週目（VISIT 6）に実施する検査・観察・評価項目を以下に示す。

- 診察、バイタルサイン
- 体重
- PML チェックリスト
- 臨床検査（検査項目は付録 A 参照）
- 部分 Mayo スコア
- 患者日誌の確認
- IBDQ
- UC 関連イベント

- 血清中薬物濃度測定用採血
- 併用薬及び併用療法
- 有害事象の評価
- 治験薬薬剤番号の発番／投与

9.3.3.2 維持期の投与（VISIT 7～17）

18 週目（VISIT 7）～58 週目（VISIT 17）に実施する検査・観察・評価項目を以下に示す。

- 診察、バイタルサイン
- 体重
- PML チェックリスト（VISIT 8、10、12、14 及び 16 のみ）
- 臨床検査（VISIT 8、10、12、14 及び 16 のみ。検査項目は付録 A 参照）
- 部分 Mayo スコア
- 患者日誌の確認
- IBDQ（VISIT 12 のみ）
- UC 関連イベント
- HAHA・中和抗体測定用採血（VISIT 10 のみ）
- 血清中薬物濃度測定用採血（VISIT 8 及び 10 のみ）
- 併用薬及び併用療法
- 有害事象の評価
- 治験薬薬剤番号の発番／投与（VISIT 8、10、12、14 及び 16 のみ）

9.3.3.3 維持期有効性主要評価（VISIT 18）／維持期中止時

60 週目（VISIT 18）に実施する検査・観察・評価項目を以下に示す。維持期の途中で中止した場合も、可能な限り速やかに来院のうえ、同様の検査・観察・評価を実施する。

- 診察、バイタルサイン
- 体重
- 心電図
- 臨床検査（検査項目は付録 A 参照）
- 完全 Mayo スコア（直腸及び S 状結腸の内視鏡検査を含む。詳細は 9.1.9 項参照。維持期中止時は内視鏡検査を実施せず、部分 Mayo スコアでも可）
- 患者日誌の確認
- IBDQ
- UC 関連イベント

- HAHA・中和抗体測定用採血
- 血清中薬物濃度測定用採血
- 併用薬及び併用療法
- 有害事象の評価

60 週目（VISIT 18）の検査完了例は 60 週目（VISIT 18）に非盲検コホートへの組み入れを可とする。また、疾患悪化例又は救済治療例は維持期中止時検査終了後に非盲検コホートへの組み入れを可とする。

9.3.4 非盲検コホート

9.3.4.1 非盲検コホートへの組み入れ（VISIT 1x）

9.1.25 項に従い、非盲検コホートへの組み入れが決まった被験者を非盲検コホートに組み入れる。0x 週目（VISIT 1x）に実施する検査・観察・評価項目を以下に示す。

- PML チェックリスト
- 併用薬及び併用療法
- 有害事象の評価
- 治験薬薬剤番号の発番／投与

9.3.4.2 非盲検コホートの投与（VISIT 2x～24x）

2x 週目（VISIT 2x）～90x 週目（VISIT 24x）に実施する検査・観察・評価項目を以下に示す。

- 診察、バイタルサイン
- 体重
- PML チェックリスト（VISIT 2x、3x、5x、7x、9x、11x、13x、15x、17x、19x、21x 及び 23x のみ）
- 臨床検査（VISIT 2x、3x、5x、7x、9x、11x、13x、15x、17x、19x、21x 及び 23x のみ。検査項目は付録 A 参照）
- 部分 Mayo スコア
- 患者日誌の確認
- UC 関連イベント
- HAHA・中和抗体測定用採血（VISIT 4x、9x 及び 17x のみ）
- 併用薬及び併用療法
- 有害事象の評価

- 治験薬薬剤番号の発番／投与（VISIT 2x、3x、5x、7x、9x、11x、13x、15x、17x、19x、21x 及び 23x のみ）

9.3.4.3 非盲検コホートの最終評価（VISIT 25x）／非盲検コホート中止時

94x 週目（VISIT 25x）に実施する検査・観察・評価項目を以下に示す。非盲検コホートの途中で中止した場合も、可能な限り速やかに来院のうえ、同様の検査・観察・評価を実施する。

- 診察、バイタルサイン
- 体重
- 心電図
- PML チェックリスト
- 臨床検査（検査項目は付録 A 参照）
- 部分 Mayo スコア
- 患者日誌の確認
- UC 関連イベント
- HAHA・中和抗体測定用採血
- 併用薬及び併用療法
- 有害事象の評価
- 治験薬薬剤番号の発番／投与（非盲検コホート中止時は実施しない）

9.3.5 予定外の来院時

治験薬を投与されたすべての被験者について、UC の悪化等のため、予定外の来院があった場合には、可能な限り以下の検査・観察・評価を実施する。

- 部分 Mayo スコア
- 患者日誌の確認
- UC 関連イベント
- 併用薬及び併用療法
- 有害事象の評価

上記の他、必要に応じて検査を追加することができることとする。

患者が疾患悪化（定義は 3.1 項参照）の基準を満たすかどうかを判断する場合、予定外の来院時の評価結果を含めて判断する。その場合、来院と来院の間隔は、可能な限り 1 週間以上空けることが望ましい。

9.3.6 計画している治験全体の終了時

最終被験者が非盲検コホートに組み入れられてから 46 週後の時点に計画している治験全体の終了時は、94x 週目（VISIT 25x）と同様の検査・観察・評価を実施する。

治験依頼者は、治験全体の終了予定日を治験責任医師に連絡する。この連絡は治験全体の終了予定日の十分前に行うこととする。

治験責任医師は、治験全体の終了予定日の連絡を受けた後に、被験者ごとの終了 VISIT を決定し、94x 週目（VISIT 25x）と同様の検査・観察・評価を実施する。

9.3.7 治験薬最終投与 16 週後

治験薬を投与されたすべての被験者について、治験薬最終投与 16 週後に以下の検査・観察・評価を実施する。治験薬最終投与後 16 週間が経過する前に終了する場合も原則として、同一の検査・観察・評価を実施する。

治験薬を投与されたすべての被験者に対して、治験の終了状態を症例報告書に記録する。

- 診察、バイタルサイン
- 体重
- 心電図
- 胸部画像検査*
- PML チェックリスト
- 臨床検査（検査項目は付録 A 参照）
- UC 関連イベント
- HAHA・中和抗体測定用採血
- 併用薬及び併用療法
- 有害事象の評価

* 3 ヵ月以内に撮影した検査画像が入手できる場合は実施しない（治験外の診療時に撮影した画像でも可）。

9.3.8 治験終了後の対応

治験薬投与終了後は、治験薬は提供されない。

9.4 生体試料の保存と廃棄

ファーマコゲノミクス検討のために採取した全血 5 mL の検体を、[redacted]にて凍結保存する。

保存期間は、本治験において最初にファーマコゲノミクス検討のための検体を採取した日から 20 年間とする。

検体の保存期間中に、被験者が検体の廃棄を希望した場合には、治験実施医療機関は、手順に従い治験依頼者を通じて[redacted]に検体の廃棄を依頼する。[redacted]は、手順に従って該当する検体を廃棄し、その旨治験実施医療機関及び治験依頼者に報告する。ただし、治験実施終了後、被験者を特定できる資料（診療録等）が廃棄されており、被験者と検体の連結が不可能な場合には廃棄しない。

なお、連結が可能な場合でも、既にファーマコゲノミクス検討が実施されている場合には、残余検体は廃棄するが、匿名化したファーマコゲノミクス検討の結果は、治験依頼者が保持する。

治験依頼者は、被験者の個人に関する情報の保護に必要な管理体制を整備し、検体の採取、保存、廃棄に関して、その取扱い基準をあらかじめ定め、必要な手順書を整備する。

9.5 追跡問診

本治験では、治験薬が投与されたすべての被験者（中止した被験者を含む）を対象として、治験薬最終投与日から 6 ヵ月ごとに、最長 2 年間又は本剤の製造販売承認日のいずれか早い日まで追跡問診用紙（付録 E）を用いて電話等による追跡問診を実施する。追跡問診項目を以下に示す。なお、追跡問診用紙は治験依頼者が回収する。

治験薬最終投与 6、12、18、24 ヵ月後[4]に実施する追跡問診項目：

- 大腸の dysplasia、癌、リンパ腫、あるいはその他の種類の癌の診断
- PML 診断
- 腸管切除

治験薬最終投与 6 ヵ月後 [4] の 1 時点でのみ実施する追跡問診項目：

- 入院を必要とする感染症の診断
- 女性被験者：妊娠
- 男性被験者：女性パートナーの妊娠

[4] 前後 1 ヵ月以内の実施を許容する。

10.0 PRETREATMENT EVENT 及び有害事象

10.1 定義

10.1.1 Pretreatment Event

Pretreatment Event（PTE）とは、同意取得後、治験薬の投与開始までに患者又は被験者に生じたあらゆる好ましくない医療上のできごと。必ずしも治験の手順との因果関係が明らかなもののみを示すものではない。

10.1.2 有害事象

有害事象とは、医薬品（治験薬を含む）が投与された患者又は被験者に生じたあらゆる好ましくない医療上のできごと。必ずしも当該医薬品（治験薬を含む）の投与との因果関係が明らかなもののみを示すものではない。

つまり有害事象とは、医薬品（治験薬を含む）が投与された際に起こる、あらゆる好ましくない、あるいは意図しない徴候（臨床検査値の臨床的に問題のある異常を含む）、症状又は病気のことであり、当該医薬品（治験薬を含む）との因果関係の有無は問わない。

10.1.3 Pretreatment Event 及び有害事象に関して考慮すべき事項

一般的に好ましくない所見には以下を含む：

- 新たに診断された疾患又は既存症状の予期せぬ悪化を示すもの（既存症状の間欠的事象はPTE又は有害事象とはみなさない）

  本治験における既存症状の間欠的事象とは、UCに伴う下痢及び腹痛等の特徴的な症状が含まれる。これらの症状は被験者ごとの病歴に基づいて治験期間中に持続又は変動するが、試験対象の病態について医学的に予想できる臨床症状とみなし、有害事象として収集しない。疾患活動性に該当するこれらの症状はMayoスコアで評価される。ただし、疾患活動性の増悪（例えば、腹痛の程度が患者の通常の変動域を超える水準まで悪化した場合や、新たな症状や所見が認められた場合）は有害事象として収集する。腸管外合併症が治験期間中に発現又は悪化した場合は有害事象として収集する。

- 処置又は診療行為を必要とするもの
- 侵襲性の診断的処置が必要なもの
- 治験薬又は併用薬の中止又は投与量の変更を必要とするもの
- 治験責任医師が好ましくないとみなしたもの

診断名と徴候・症状：

PTE 又は有害事象（以下、有害事象等とする）は診断名で記載する。随伴する徴候（臨床検査値の異常、心電図の異常所見を含む）及び症状は有害事象等としない。診断名がつかない有害事象等の場合には、徴候や症状を有害事象等とする。

臨床検査値及び心電図所見：

臨床検査値異常や心電図所見については、推移より臨床的に問題があると治験責任医師又は治験分担医師が判断した場合には、有害事象等とする（すなわち、何らかの処置又は診療行為を必要とする場合、又は治験責任医師又は治験分担医師が、被験者の正常な生理的変動値域を超えた変化であると判断した場合）。再検査及び／又は異常値の継続的なモニタリングは診療行為とはみなさない。さらに、検証、評価、異常値のモニタリングの目的で非侵襲性検査を繰返し実施すること又は追加で実施することも診療行為とはみなさない。

ただし、臨床検査値異常や心電図所見が、有害事象等としての診断名がつく疾患に随伴する場合（例：腎機能障害によるクレアチニン増加等）には、その診断名を有害事象等とする。

既存症状（同意取得前から存在する疾患、症状）：
同意取得前から存在する疾患、症状については合併症とし、有害事象等としない。また、同意取得後の最初の検査・観察（例：臨床検査、心電図、X 線検査等）によって認められた異常所見は、治験手順に関連する場合を除き PTE としない。ただし、最初の検査・観察の手順によって生じた異常所見（例：採血時の内出血等）は PTE とし、症例報告書に記録する。合併症が悪化した場合には、その悪化を有害事象等とし、治験責任医師又は治験分担医師はその有害事象等が合併症の悪化であることを症例報告書に記録する（例 :「高血圧の悪化」等）。

被験者が一過性の既存症状（例：喘息、てんかん）を有する場合にその症状の発現頻度が増加、あるいは重篤又は高度となる場合には、有害事象等として記録する。また、被験者が慢性的な疾患（例：白内障、関節リウマチ）を有する場合、予想以上に症状の悪化が見られる場合には、有害事象等として記録する。治験責任医師又は治験分担医師は、報告する有害事象名がベースラインからの症状の変化（例：～の悪化）であることがわかるように記録する。

有害事象等の悪化：
被験者が治験薬投与開始後に PTE が悪化した場合、あるいは PTE によって二次的に徴候・症状が発現した場合には、有害事象とし、症例報告書に記録する。

治験責任医師又は治験分担医師は、報告する有害事象名が症状の変化（例：～の悪化）であることがわかるように記録する。

治験薬が変更された後に有害事象が悪化した場合、あるいはその有害事象により二次的に徴候・症状が発現した場合には、新たな有害事象とし、症例報告書に記録する。治験責任医師又は治験分担医師は、報告する有害事象名が症状の変化（例：～の悪化）であることがわかるように記録する。

有害事象／重篤な PTE の重症度の変化：
被験者の有害事象／重篤な PTE の重症度に変化がある場合には、重症度が最大となったときの一回を記録する。

あらかじめ計画されていた手術又は処置：
同意取得前にあらかじめ計画されていた手術又は処置は、有害事象等としない。ただし、その手術又は処置を緊急的に実施する必要が生じるような既存症状の悪化が生じた場合には、その状態や事象を有害事象等とする。あらかじめ計画されていた手術による合併症は、有害事象として報告する。

急を要しない手術又は処置：
被験者の病状に変化を与えない急を要しない手術又は処置（美容整形等）は有害事象等としない。ただし、原資料に記録しておく。急を要しない手術による合併症は、有害事象として報告する。

臨床的な反応の不足（有効性の欠如）：
臨床的な反応、有効性、薬理作用の不足は有害事象としない。治験責任医師又は治験分担医師は、既存症状の悪化と有効性の欠如を区別して取り扱う。

過量投与：
過量投与は有害事象としないが、過量投与を症例報告書の過量投与頁に記録する。過量投与により事象が発現した場合には、有害事象として症例報告書の有害事象頁に記録する。

10.1.4 重篤な有害事象

重篤な有害事象とは、医薬品が投与された（投与量に係わらない）際に生じたあらゆる好ましくない医療上のできごとのうち、以下のものをいう。

1. 死に至るもの
2. 生命を脅かすもの*
3. 入院又は入院期間の延長が必要となるもの
4. 永続的又は顕著な障害若しくは機能不全に陥るもの

5. 先天異常をきたすもの
6. その他の医学的に重大な状態
   即座に生命を脅かしたり、死や入院には至らなくとも、被験者を危険にさらしたり、上記 1〜5 のような結果に至らぬように処置や治療を必要とするような重大な医学的事象。なお、Takeda Medically Significant AE List（表 10.a）に記載されているものは本項に含まれる。

*「生命を脅かす」とは、その事象が起こった際に被験者が死の危険にさらされていたという意味であり、その事象がもっと重症なものであったなら死に至っていたかもしれないという仮定的な意味ではない。

表 10.a　Takeda Medically Significant AE List

急性呼吸不全／急性呼吸窮迫症候群（ARDS）	肝壊死
	急性肝不全
トルサード　ド　ポアン／心室細動／心室性頻脈	アナフィラキシーショック
	急性腎不全
悪性高血圧	肺高血圧症
痙攣性発作（痙攣、てんかんを含む）	肺線維症（間質性肺炎を含む）
無顆粒球症	悪性症候群／悪性高熱
再生不良性貧血	自然流産／死産及び胎児死
中毒性表皮壊死融解症／皮膚粘膜眼症候群（スティーブンス・ジョンソン症候群）	薬剤を介した感染症の伝播又は伝播の疑い
	エンドトキシンショック又はその疑い

なお、Pretreatment Event が上記重篤の基準に該当する場合には、重篤な有害事象と同様の手順で報告する（10.2.2 項及び 10.3 項参照）。

10.1.5　Pretreatment Event 及び有害事象の程度

有害事象等の程度については次のように分類・定義する。

軽　度	一過性で容易に耐えられるもの
中等度	通常の活動に支障をきたす程度のもの
高　度	通常の活動を不可能にする程度のもの

10.1.6　有害事象の因果関係

有害事象の治験薬との因果関係については次のように分類・定義する。

関連あり	時間的な相関関係（投与中止後の経過を含む）がある。又は、原疾患、合併症、併用薬、併用処置等の他要因も推定されるが、当該被験薬、対照薬による可能性も考えられる有害事象。
関連なし	当該被験薬、対照薬と時間的な相関関係がない。又は、原疾患、合併症、併用薬、併用処置等の他要因によると十分に考えられる有害事象。

10.1.7　治験手順との因果関係

有害事象等について、治験責任医師又は治験分担医師が、治験の手順に起因すると判断する場合には、因果関係を「有」とし、治験の手順に起因しないと判断する場合には「無」とする。

10.1.8　発現日

有害事象等の発現日は、以下の基準で判断する。

有害事象等	発現日
徴候、症状、疾患（診断名）の場合	被験者又は治験責任医師又は治験分担医師が最初に有害事象の徴候、症状に気付いた日を記載する。
無症候性の疾患の場合	診断のために検査が実施され、診断が確定した日を記載する。検査所見から陳旧性の所見が見られる場合や、発生時期がおおよそ推定できる場合でも、診断が確定した日を記載する。
合併症又は Pretreatment Event の悪化の場合	被験者又は治験責任医師又は治験分担医師が最初に疾患、症状の悪化に気付いた日を記載する。
同意取得後の最初の検査で正常であったものが、その後の検査で異常となった場合（PTE の場合）<br>治験薬投与開始後の検査で異常となった場合（有害事象の場合）	臨床的に問題があると判断される検査値異常が認められた検査日を記載する。
同意取得後の最初の検査で異常が見られ、その後の検査で悪化した場合（PTE の場合）<br>治験薬投与開始時点の検査で異常が見られ、その後の検査で悪化した場合（有害事象の場合）	検査値の推移より医学的判断で明らかな上昇、低下、増加、減少が認められた検査日を記載する。

10.1.9　消失日

有害事象等が、回復した日（回復したが後遺症ありを含む）とする。当該有害事象等で死亡した場合には、死亡日とする。治験終了時に回復が確認できない場合には、継続中と判定する。

10.1.10 頻度

見かけ上、消失と発現を繰り返す有害事象等（例：便秘、下痢、嘔吐等）について、治験責任医師又は治験分担医師が最初の発現から最終の消失までを一連の一つの事象と判断する場合は「間欠的」とする。それ以外の場合は「継続的」とする。

10.1.11 治験薬に関する処置

治験薬に関する処置については次のように分類・定義する。

投与中止	当該有害事象に対する処置として治験薬の投与が中止された場合（被験者の自己判断による中止も含む）を「投与中止」とする。
投与量変更せず	当該有害事象発現後も投与量を変更しなかった場合を「投与量変更せず」とする。<br>当該有害事象以外の有害事象により治験薬の投与が中止・減量・増量された場合は「投与量変更せず」とする。<br>また、被験者の不注意等の有害事象への対処以外の理由によって服薬を止めたり、減量する等した場合には「投与量変更せず」とする。
不明	被験者と連絡が取れず、発現日以降の経過が把握できない場合等。
該当せず	当該有害事象の発現を認めた時点で、治験薬の投与が既に終了又は中止されていた場合等。

10.1.12 転帰

有害事象等の転帰については次のように分類する。

区分	判断基準
回復	・症状、所見の消失あるいは回復<br>・検査値の正常化あるいは投与前の検査値（有害事象の場合）若しくは同意取得後最初の検査値（PTE の場合）への回復
軽快	・程度が 1 段階以上軽減<br>・症状、所見がほぼ消失<br>・検査値の改善が認められたが、正常化あるいは投与前の検査値（有害事象の場合）若しくは同意取得後最初の検査値（PTE の場合）に回復していない<br>・死亡例で、当該有害事象等が直接の死因でない場合で、当該有害事象等が

	軽快のまま死亡（この場合は死亡日の記載は不要）
未回復	・症状、所見や検査値に変化がない<br>・観察できた期間の最後の日の症状、所見や検査値が発現時の程度より悪化<br>・不可逆性の先天異常<br>・死亡例で、当該有害事象等が直接の死因でない場合で、当該有害事象等が未回復のまま死亡（この場合は死亡日の記載は不要）
回復したが後遺症あり	・日常生活に支障をきたす程度の機能不全が起きた
死亡	・死亡と当該有害事象等との間に直接の関連性が認められた<br>「直接の関連性が認められた」とは、当該有害事象等が死亡の原因になった、又は当該有害事象等が明らかに死亡に寄与したことをさす<br>・同一症例で見られた直接の死因ではないと判定（判断、推定）される有害事象等の転帰については、死亡としない<br>・転帰が、死亡の場合には、死亡日を記載する
不明	・発現日以降の経過が、転院、転居等により治験実施計画書に記載されている追跡が不可能となった

10.2 手順

10.2.1 有害事象等の収集及び報告

10.2.1.1 有害事象等の収集期間

Pretreatment Event の収集は、同意取得後から開始し、治験薬の初回投与（VISIT 2）まで継続して行う。なお、治験薬初回投与までに中止が決定した場合には、その時点までとする。

有害事象の収集は、被験者への治験薬投与後（VISIT 2）より開始し、治験薬最終投与 16 週後に実施する終了時検査時まで継続して行う。

10.2.1.2 有害事象等の報告

治験責任医師又は治験分担医師は、被験者来院の際、自覚症状の発現の有無を確認する。「前回の来院以降どうでしたか」等の質問を行い、来院時以外に発現した有害事象等を被験者から聴取する。

Pretreatment Event が重篤の基準に該当する場合には、治験責任医師又は治験分担医師は、症状が消失するまで、臨床的に問題がある検査値の異常が同意取得後の最初の検査値に回復するまで、そうでない場合（永続的・不可逆的な Pretreatment Event）には、観察された変化について十分な説明がつくまで、すべての被験者を追跡調査する。Pretreatment Event が重篤の基準に該当しない場合

には、治験手順との関連性に係わらず、治験実施計画書上の追跡調査は必要としない。

有害事象については、治験責任医師又は治験分担医師は、治験薬との関連性に係わらず、症状が消失するまで、臨床的に問題がある検査値の異常が治験薬投与前値に回復するまで、そうでない場合（永続的・不可逆的な有害事象）には、観察された変化について十分な説明がつくまで、すべての被験者を追跡調査する。すべての有害事象等を症例報告書に記録する。有害事象等の名称、発現日、消失日、発現時期、頻度、程度、治験薬との因果関係（関連なし、関連あり）、治験薬に関する処置、転帰、治験手順との因果関係（さらに因果関係が有の場合には原因と考えられる手順）及び重篤性について、記録する。

治験薬注入開始から治験薬注入中及び治験薬注入 1 時間後までに発現した有害事象については、発現時刻及び注入に伴う反応かどうかを症例報告書に記録する。

有害事象及び重篤の基準に該当する Pretreatment Event の追跡期間は、有害事象等が回復するまで、あるいは治験責任医師又は治験分担医師がこれ以上の追跡は不要と判断するまでの期間とする。

10.2.2 重篤な有害事象の収集及び報告

有害事象の収集期間に重篤な有害事象が発現した場合には、以下の手順に従って報告する。なお、Pretreatment Event が 10.1.4 項に記載の重篤の基準に該当する場合には、重篤な有害事象と同様の手順で報告する。

治験責任医師又は治験分担医師は、重篤な有害事象の発現時又は被験者等からの事象発現の通知後、1 就業日以内に、治験依頼者（連絡先は別紙参照）に報告する。さらに、治験責任医師は 10 暦日以内に正式な詳細報告書を提出する。

なお、1 就業日以内に報告する内容は以下を必須とし、その他の項目についても可能な限り連絡する。

- 有害事象の簡潔な記述及び重篤であると判断した理由
- 被験者識別コード
- 治験責任医師名又は治験分担医師名
- 治験薬名
- 因果関係（治験薬及び治験手順）の判定

治験責任医師又は治験分担医師は、有害事象の収集期間の後、追跡問診期間中を含め、自発的に報告された重篤な有害事象についても治験依頼者へ報告する。

10.2.3 重症肝障害につながる可能性のある薬剤起因性肝機能検査異常の報告

ALT 又は AST が基準値上限の 3 倍を超え、かつ総ビリルビン値が基準値上限の 2 倍を超えた場合には、治験責任医師又は治験分担医師は、治験依頼者に報告するとともに、当該被験者の詳細情報及び可能性のある治験薬以外の要因（急性ウイルス性 A 型又は B 型肝炎、その他急性肝疾患等の有無、既往歴・合併症）について速やかに調査を行う。また、追跡検査を実施する（9.1.12 項参照）。これらの肝機能検査異常が他の要因では説明できないと判断した場合には、重篤な有害事象として報告する（10.2.2 項参照）。

10.3 重篤な有害事象の追跡調査

詳細報告の際に報告されなかった情報を後日入手した場合には、治験責任医師又は治験分担医師は、重篤な有害事象の報告書の写しに記入するか別の文書を作成し、別紙に示す連絡先に提出する。治験依頼者又は治験審査委員会から要求された場合には、治験実施医療機関の関連データ（例：心電図、臨床検査値、退院報告の要約、検死結果等）を提供する。

治験責任医師又は治験分担医師は、すべての重篤な有害事象が回復するまで、又は最終の転帰が確定するまで追跡調査する。

10.3.1 重篤な有害事象の治験責任医師、治験審査委員会及び規制当局への報告

治験依頼者は、治験実施国の規制に従って未知の重篤な副作用（SUSARs）及び緊急報告対象となるその他の重篤な有害事象を規制当局、治験責任医師及び治験実施医療機関の長に報告する。治験依頼者又は治験依頼者が委託した開発業務受託機関がその事象を最初に知った時点又は追加情報を入手した時点から、SUSARs のうち、死亡又は生命を脅かす有害事象は 7 日以内、それ以外の重篤な有害事象は 15 日以内に規制当局に緊急報告する。また、治験依頼者は治験薬のリスクベネフィット、治験薬の投与継続、治験の継続に大きく影響を与えると考えられるその他重大な安全性情報についても同様に緊急報告を行う。治験実施医療機関は、緊急報告された書類の写しを治験審査委員会に提出する。

11.0 本治験のために設置された委員会

11.1 PML 独立判定委員会

本治験では、PML のリスクを評価するために設置された PML 独立判定委員会を利用する。付録 D に定める PML チェックリストに従い、神経科医の診察により PML の可能性が排除できない場合に、PML 独立判定委員会に当該症例のデータを提出し、その判断を仰ぐ。本手順の詳細は、別途定める手順書に定める。

11.2 中央判定委員会

中央判定委員会は、評価項目の判定に関して適切な経験を持つ独立した専門家で構成する。委員は、評価項目である粘膜所見スコアを確定するため、治験実施医療機関から報告される内視鏡検査結果をすべて確認し、判定結果を治験依頼者に報告する。中央判定委員会の評価結果は臨床試験のデータベースに入力し、評価項目の解析に使用する。中央判定委員会の手順書に目的及び責務を記載する。

12.0 データマネジメント及び記録の保存

データマネジメントに関する詳細な手順については、データマネジメント計画書に記載する。有害事象、Pretreatment Event、既往歴及び合併症は MedDRA を用いて読み替える。薬剤については、WHO Drug 及び医療用医薬品名データファイルを用いて読み替える。

12.1 症例報告書

治験責任医師又は治験分担医師は、同意を取得したすべての被験者について症例報告書を作成する。

治験依頼者又はその指名する者は、治験実施医療機関に 電子症例報告書システム へのアクセス権を提供する。治験依頼者は、電子症例報告書システムの利用に際し、治験責任医師、治験分担医師及び治験協力者を対象にトレーニングを提供する。症例報告書は治験実施の際に収集された情報を治験依頼者及び規制当局に報告するために使用される。症例報告書 は日本語で作成する。症例報告書の作成にあたっては、データを直接入力する。

症例報告書の変更又は修正は、変更又は修正前後の情報、変更又は修正者、変更又は修正日及びその理由を記録した監査証跡として記録される。

治験責任医師は、症例報告書が正確かつ完全に記録されているかを確認し、症例報告書の該当頁に電子署名する。治験責任医師は、症例報告書 に記録された全データに関する正確性と信頼性について全責任を負う。

以下は症例報告書 に直接記録されないデータである。

1) 臨床検査値（尿妊娠検査を除く）
2) MLN0002 の血清中濃度
3) HAHA 評価
4) 中和抗体評価
5) 完全 Mayo スコア及び部分 Mayo スコア（各サブスコアを含む）
6) 10 週目（VISIT 5）における改善／非改善の判定結果
7) 追跡問診項目
8) 中央判定委員会による内視鏡判定結果

臨床試験データベースを固定した後に、治験責任医師又は治験分担医師が症例報告書の入力内容を変更又は修正する場合には、治験依頼者から提供される症例報告書の変更及び修正記録（Data Clarification Form）を用いて行う。治験責任医師は、症例報告書の変更及び修正記録が正確かつ完全に記載されているかを確認後、署名又は記名・捺印し、日付を記載する。

治験依頼者又はその指名する者は、治験実施医療機関への訪問の際、症例報告書が正確かつ完全に作成されていることを確認する。治験依頼者又はその指名する者は、症例報告書の正確性を確保するため、治験に関連する被験者の医療記録及び院内記録を閲覧する。作成が完了した症例報告書は治験依頼者の所有物であり、治験責任医師又は治験分担医師は、その情報を治験依頼者の書面による許可なしに、規制当局を除く第三者に開示してはならない。

12.2 記録の保存

治験責任医師又は治験実施医療機関の長は、規制当局及び治験依頼者又はその指名する者による調査又は監査のため、12.1 項に明記した記録及び治験固有の文書を含む以下の資料を保管する。資料には、被験者スクリーニング名簿、医療記録、治験ワークシート、署名・日付入り同意書原本、監査証跡を含む電子症例報告書の電子的コピー、薬剤管理記録等がある。また、治験責任医師及び治験実施医療機関の長は、保管すべき必須文書を、以下の 1）又は 2）のいずれか遅い日までの間保存しなければならない。ただし、治験依頼者がこれよりも長期間の保存を必要とする場合には、治験実施医療機関の長は、保存期間及び保存方法について治験依頼者と協議する。

1） 当該被験薬に係る医薬品についての製造販売承認日（開発が中止された場合にはその旨の通知を受けた日から 3 年が経過した日）
2） 治験の中止又は終了後 3 年が経過した日

さらに、治験責任医師及び治験実施医療機関の長は、保存すべき必須文書についてその保存の必要がなくなった旨治験依頼者から通知を受けるまで保存する。

13.0 統計手法

13.1 統計及び解析計画

本治験において、導入期と維持期又は非盲検コホートでは治験の目的が異なるため、導入期と維持期又は非盲検コホートで解析を分けて行う。

解析担当者は、治験薬の割付を明らかにする前に統計解析計画書を作成し、最終化する。統計解析計画書には、すべての治験の目的に対処するため、評価項目の定義及び解析方法の詳細を記載する。

盲検下レヴューは治験薬の割付を明らかにする前に行う。盲検下レヴューでは、治験データの正確性及び完全性、被験者の評価可能性及び計画した解析方法の妥当性を評価する。

解析は、承認申請のために設定されるカットオフ日までの全症例のデータを固定し、その承認申請用データを用いて行う。また、全症例が治験を終了した時点で、非盲検コホートについて改めて解析を行う。

13.1.1 解析対象集団

本治験では、導入期の解析対象集団として「導入期の最大の解析対象集団」、「導入期の治験実施計画書に適合した対象集団」、「導入期の安全性データの解析対象集団」の 3 種類の解析対象集団を設ける。

導入期の有効性の解析の主たる解析対象集団として用いる「導入期の最大の解析対象集団」は、「導入期に無作為化され、導入期に治験薬を少なくとも 1 回以上投与された被験者」として定義する。

維持期の解析対象集団として「維持期の最大の解析対象集団」、「維持期の治験実施計画書に適合した対象集団」、「維持期の安全性データの解析対象集団」の 3 種類の解析対象集団を設ける。

維持期の有効性の解析の主たる解析対象集団として用いる「維持期の最大の解析対象集団」は、「維持期に無作為化され、維持期に治験薬を少なくとも 1 回以上投与された被験者」として定義する。

非盲検コホートの解析対象集団として「非盲検コホートの最大の解析対象集団」、「非盲検コホートの安全性データの解析対象集団」の 2 種類の解析対象集団を設ける。

安全性の解析の解析対象集団として用いる「非盲検コホートの安全性データの解析対象集団」は、「非盲検コホートにおいて治験薬を少なくとも 1 回以上投与された被験者」として定義する。

解析対象集団の詳細な定義は、別途「解析上のデータハンドリング・ルール」に定める。

治験依頼者は、データ固定前に、必要に応じて医学専門家と協議のうえ、解析対象集団の定義及びその集団における症例データの解析上の取扱いルールの妥当性を確認するとともに、計画段階で規定されていない問題事項に対する取扱いを補足したうえで「解析上のデータハンドリング・ルール」を最終確定する。

13.1.2 人口統計学的及び他の基準値の特性の解析

「導入期に無作為化された全例」及び「導入期のコホート 2 に組み入れられた全例」を対象として、主要な被験者背景項目について「導入期のコホート及び投与群」別及び「導入期のコホート及び投与群」を併合して集計を行う。

「維持期に無作為化された全例」を対象として、主要な被験者背景項目について「維持期の投与群」別及び「維持期の投与群」を併合して集計を行う。

「非盲検コホートに組み入れられた全例」を対象として、主要な被験者背景項目について集計を行う。

「導入期にプラセボを投与され、維持期に組み入れられた全例」を対象として、主要な被験者背景項目について集計を行う。

13.1.3 有効性の解析

(1) 導入期の主要評価項目とその解析方法

〔主要評価項目〕

10 週目改善率

〔主解析〕

「導入期の最大の解析対象集団」を対象として以下の集計解析を行う。

導入期の主要評価項目である「10 週目改善率」について、導入期の投与群別に頻度集計を行う。「抗 TNFα 抗体前治療歴の有無」により層別したうえで同様の集計を行い、「抗 TNFα 抗体前治療歴の有無」を層別因子とした Cochran-Mantel-Haenszel 検定を行う。なお、粘膜所見スコアは実施医療機関の判定結果を用いる。

MLN0002 の導入療法の有効性を検討するため、導入期の主解析の結果を以下のとおり解釈する。

- 導入期の主解析における 10 週目改善率の検定結果が有意であった場合に、10 週目改善率について MLN0002 投与のプラセボ投与に対する優越性が検証されたとする。

〔副解析〕

感度分析の考え方から、「導入期の治験実施計画書に適合した対象集団」を対象として、13.1.3 (1)の主解析と同様の解析を行い、結果の安定性を検討する。また、「導入期の最大の解析対象集団」を対象として、中央判定委員会の判定に基づく粘膜所見スコアを用いて、13.1.3 (1)の主解析と同様の解析を行う。

(2) 導入期の副次評価項目とその解析方法

〔副次評価項目〕

10 週目寛解率

10 週目粘膜治癒率

〔副解析〕

「導入期の最大の解析対象集団」を対象として、10 週目寛解率について 13.1.3 (1)の主解析と同様の解析を行う。なお、粘膜所見スコアは実施医療機関の判定結果を用いる。

MLN0002 の導入療法の有効性を検討するため、導入期の副解析の結果を以下のとおり解釈する。

- 導入期の主解析における 10 週目改善率の検定結果が有意であり、かつ、10 週目寛解率の検定結果が有意であった場合に、10 週目寛解率について MLN0002 投与のプラセボ投与に対する優越性が検証されたとする。

〔その他の解析〕

「導入期の最大の解析対象集団」を対象として、10 週目粘膜治癒率について 13.1.3 (1)の主解析と同様の解析を行う。なお、粘膜所見スコアは実施医療機関の判定結果を用いる。

(3) 導入期の探索的評価項目

5.2.1.3 項を参照。

(4) 維持期の主要評価項目とその解析方法

〔主要評価項目〕

60 週目寛解率

〔主解析〕

「維持期の最大の解析対象集団」を対象として以下の集計解析を行う。

維持期の主要評価項目である「60 週目寛解率」について、維持期の投与群ごとに頻度集計を行う。「抗 TNFα 抗体前治療歴の有無」により層別したうえで同様の集計を行い、「抗 TNFα 抗体前治療歴の有無」を層別因子とした Cochran-Mantel-Haenszel 検定を行う。なお、粘膜所見スコアは実施医療機関の判定結果を用いる。

MLN0002 の維持療法の有効性を検討するため、維持期の主解析の結果を以下のとおり解釈する。

- 維持期の主解析における 60 週目寛解率の検定結果が有意であった場合に、60 週目寛解率について MLN0002 投与のプラセボ投与に対する優越性が検証されたとする。

〔副解析〕

感度分析の考え方から、「維持期の治験実施計画書に適合した対象集団」を対象として、13.1.3 (4)の主解析と同様の解析を行い、結果の安定性を検討する。また、「維持期の最大の解析対象集団」を対象として、中央判定の粘膜所見スコアを用いて、13.1.3 (4)の主解析と同様の解析を行う。

(5) 維持期の副次評価項目とその解析方法

〔副次評価項目〕

持続改善率

60 週目粘膜治癒率

持続寛解率

ステロイドフリー60 週目寛解率

〔解析方法〕

「維持期の最大の解析対象集団」を対象として、持続改善率、60 週目粘膜治癒率、持続寛解率及びステロイドフリー60 週目寛解率について、13.1.3 (4)の主解

析と同様の解析を行う。なお、粘膜所見スコアは実施医療機関の判定結果を用いる。

(6) 維持期の探索的評価項目

5.2.2.3 項を参照。

(7) データの変換方法及び欠測データの取扱い

詳細は別途「解析上のデータハンドリング・ルール」及び「統計解析計画書」に定める。

改善率、寛解率、粘膜治癒率に関する有効性の評価項目について、当該評価時点の判定が欠測の場合は非改善、非寛解、非粘膜治癒として取扱う。

(8) 有意水準、信頼係数

- 有意水準：5%（両側検定）
  本治験において、導入期と維持期では治験の目的が異なるため、導入期及び維持期の有意水準をそれぞれ 5%とし、導入期と維持期の間の多重性の調整は行わない。
- 信頼係数：95%（両側推測）

13.1.4 薬物動態の解析

〔評価項目〕

MLN0002 の血清中濃度

〔解析方法〕

「導入期の最大の解析対象集団」のうち、MLN0002 の血清中濃度が適切に測定された被験者を対象として、導入期における各評価時点の MLN0002 の血清中濃度について、導入期のコホート別及びコホートを併合して要約統計量を算出し、平均・標準偏差推移図を作成する。

「維持期の最大の解析対象集団」のうち、MLN0002 の血清中濃度が適切に測定された被験者を対象として、維持期における各評価時点の MLN0002 の血清中濃度について、維持期の投与群別に要約統計量を算出し、平均・標準偏差推移図を作成する。

13.1.5 安全性の解析

(1) 導入期の有害事象

「導入期の安全性データの解析対象集団」を対象として、以下の解析を行う。

導入期の TEAE（Treatment-emergent adverse event）を、維持期又は非盲検コホートに組み入れられた被験者については、導入期の治験薬投与開始後から維持期又は非盲検コホートの治験薬投与開始までに発現した有害事象と定義する。維持期又は非盲検コホートに組み入れられなかった被験者については、導入期の治験薬投与開始後に発現した有害事象と定義する。

導入期の TEAE について､以下の解析を導入期のコホート及び投与群別に行う。コホート 1 の MLN0002 投与群とコホート 2 を併合して同様の解析を行う｡TEAE は MedDRA を用いて読み替え、器官別大分類（System Organ Class）及び基本語（Preferred Term）で要約する。

- すべての TEAE の頻度集計
- 治験薬との因果関係が「関連あり」である TEAE の頻度集計
- すべての TEAE の程度別頻度集計
- 治験薬との因果関係が「関連あり」である TEAE の程度別頻度集計
- 治験薬に関する処置が「投与中止」である TEAE の頻度集計
- 重篤な TEAE の頻度集計
- すべての TEAE の発現時期別頻度集計

(2) 維持期の有害事象

維持期の TEAE を、非盲検コホートに組み入れられた被験者については、維持期の治験薬投与開始後から非盲検コホートの治験薬投与開始までに発現した有害事象と定義する。非盲検コホートに組み入れられなかった被験者については、維持期の治験薬投与開始後に発現した有害事象と定義する。

「維持期の安全性データの解析対象集団」を対象として、維持期の投与群別に維持期の TEAE について 13.1.5 (1)と同様の解析を行う。

「導入期にプラセボ群に割り付けられ、維持期にプラセボを投与された被験者」を対象として、維持期の TEAE について 13.1.5 (1)と同様の解析を行う。

(3) 非盲検コホートの有害事象

非盲検コホートの TEAE を、非盲検コホートの治験薬投与開始後に発現した有害事象と定義する。

「非盲検コホートの安全性データの解析対象集団」を対象として、非盲検コホートの TEAE について 13.1.5 (1)と同様の解析を行う。

(4) MLN0002 投与後に発現した有害事象

MLN0002 投与後に発現した TEAE を、MLN0002 投与開始後に発現した有害事象と定義する。

「MLN0002 を少なくとも 1 回以上投与された被験者」を対象として、MLN0002 投与後に発現した TEAE について 13.1.5 (1)と同様の解析を行う。

(5) 臨床検査値、12 誘導心電図、バイタルサイン、体重

解析対象集団及び解析する群は、原則として 13.1.5 (1)、(2)、(3)と同様とする。

計量値について、検査値及び投与前後差の要約統計量を評価時点ごとに算出する。検査値の個別推移図を作成する。

計数値について、投与前後のシフトテーブルを作成する。

13.1.6 免疫原性の評価項目の解析

〔評価項目〕

HAHA、中和抗体

〔解析方法〕

「導入期の最大の解析対象集団」のうち、HAHA 及び中和抗体の測定が適切に行われた被験者を対象として、HAHA 及び中和抗体の頻度について、導入期のコホート別及びコホートを併合して集計を行う。

「維持期の最大の解析対象集団」のうち、HAHA 及び中和抗体の測定が行われた被験者を対象として、HAHA 及び中和抗体の頻度について、維持期の投与群別に集計を行う。

「非盲検コホートの最大の解析対象集団」のうち、HAHA 及び中和抗体の測定が行われた被験者を対象として、HAHA 及び中和抗体の頻度集計を行う。

13.2 中間解析及び早期中止に関する基準

承認申請のために設定されるカットオフ日までの全症例のデータを固定し、その承認申請用データを用いて解析を行う。本解析による治験の継続・中止の判断は行わない。

13.3 計画被験者数の決定

導入期の無作為化例数：246 例

導入期の評価可能例数（「導入期に無作為化され、導入期に治験薬を少なくとも 1 回以上投与された被験者」の例数）

導入期の MLN0002 投与群：164 例

導入期のプラセボ投与群：82 例

合計：246 例

維持期の組み入れ例数：約 110～140 例

維持期の評価可能例数（「維持期に無作為化され、維持期に治験薬を少なくとも 1 回以上投与された被験者」の例数）

維持期の MLN0002 投与群 42 例～55 例

維持期のプラセボ投与群 42 例～55 例

合計：84 例～110 例

〔計画被験者数の設定根拠〕

海外で実施した中等症から重症の UC 患者の導入療法及び維持療法におけるプラセボ対照第 3 相二重盲検比較試験（C13006）の成績を参考に、本治験の MLN0002 投与とプラセボ投与による 10 週目改善率を 57.1%と 35.5%、10 週目寛解率を 16.9%と 5.4%、60 週目寛解率を 43.3%と 15.9%と想定した。

導入期に関して、10 週目改善率について有意水準を両側 5%として Pearson のカイ二乗検定を適用する場合、MLN0002 投与群とプラセボ投与群の例数の比を 2:1 として 90%以上の検出力を確保するためには、MLN0002 投与群に 164 例、プラセボ投与群に 82 例、合計 246 例必要となることから、導入期の主要評価項目の評価可能例数としてこれらの例数を設定した。10 週目寛解率について、本例数のもとで同様の解析を行ったときの 10 週目改善率との同時検出力は 69%である。

維持期に関して、60 週目寛解率について有意水準を両側 5%として Pearson のカイ二乗検定を適用する場合、80%以上の検出力を確保するためには、各群 42 例、合計 84 例必要となる。同様に、90%以上の検出力を確保するためには、各群 55 例、合計 110 例必要となることから、維持期の主要評価項目の評価可能例数としてこれらの例数を設定した。導入期の盲検性維持のため、維持期に組み入れられた被験者には「導入期にプラセボ群に割り付けられた被験者」が含まれることから、維持期の組み入れ例数としては、維持期の主要評価項目の検出力 80%、90%を確保する例数としてそれぞれ 110 例、140 例が必要となる。

14.0 品質管理及び品質保証

14.1 治験実施医療機関へのモニタリング

治験依頼者又はその指名する者は、治験実施計画書に記載されているすべての内容に従って治験が実施されているかを確認するため、治験実施医療機関へのモニタリングを治験期間を通して定期的に行う。症例報告書に記録されているデータを確認するため、原資料と照合する。原資料とは元となる文書、データ及び記録のことをいう。治験責任医師及び治験実施医療機関の長は、治験依頼者又はその指名する者及び治験審査委員会が原資料を閲覧できることを保証する。

治験依頼者又はその指名する者は、治験責任医師ファイル、薬剤、被験者の医療記録、同意書を含む記録を閲覧し、治験が治験実施計画書を遵守して適切に行われていることを確認する。また、症例報告書と関連する原資料との整合性を確認する。治験責任医師又は治験分担医師及びその他治験担当職員は、モニタリングのための治験実施医療機関への訪問が行われる際は作業進行のために十分な時間を割いて、対応するよう努める。

14.2 治験実施計画書からの逸脱

治験責任医師又は治験分担医師は、被験者の緊急の危険を回避するためのものである等医療上やむを得ない事情のために、治験依頼者との事前の文書による合意及び治験審査委員会の事前の承認なしに治験実施計画書からの逸脱又は変更を行うことができる。その際には、治験責任医師は、逸脱又は変更の内容及び理由を治験依頼者及び治験実施医療機関の長に文書で通知するとともにその写しを保管し、必要に応じ、治験実施計画書の改訂について治験依頼者と協議し、合意する。改訂する場合には、その案を可能な限り早急に治験実施医療機関の長に提出し、治験審査委員会の承認を得る。

治験責任医師又は治験分担医師は、治験実施計画書から逸脱した行為をすべて記録する。

14.3 品質保証及び規制当局による調査

治験依頼者又はその指名する者は、必要に応じて治験実施医療機関において監査を実施する。この場合には、治験依頼者が指名した監査担当者は事前に治験実施医療機関と連絡を取り、監査のための訪問を取り決める。監査担当者は、臨床検査検体の保管場所、薬剤の保管及び調剤の場所及びその他治験期間中使用する施設への訪問を要求できる。また、本治験は、海外政府機関（例：米国食品医薬品局（FDA）、英国医薬品庁（MHRA））を含む規制当局によって調

査されることもある。治験実施医療機関が規制当局から調査を要求する連絡を受けた場合には、治験依頼者に速やかに通知する。治験責任医師及び治験実施医療機関の長は、監査担当者が 14.1 項に記載された治験関連文書をすべて閲覧できることを保証する。

15.0 治験の倫理的な実施

本治験は、治験参加者（すなわち被験者）個人に配慮し、治験実施計画書、ヘルシンキ宣言に基づく倫理的原則及び医薬品の臨床試験の実施の基準に関するICH 三極統一ガイドラインに従って実施する。各治験責任医師は、規制要件に則り、付録 B の「治験責任医師の責務」に従って、治験を実施する。

15.1 治験審査委員会の承認

治験審査委員会は、各治験に参加する地域の規制に従って構成される。治験依頼者又はその指名する者は、各治験審査委員会委員の氏名、職名を記載した文書を入手する。治験審査委員会の委員が本治験に直接参加している場合には、審議、採決に参加していない旨の文書を入手する。

治験依頼者又はその指名する者は、治験実施計画書の審査と承認のために各治験審査委員会に関連書類を提供する。また、本治験実施計画書の他に、治験薬概要書、同意・説明文書の写し及び必要に応じて被験者募集に関する資料、広告、その他各地域の規制によって求められる文書を、中央又は各治験実施医療機関の治験審査委員会に提出し、承認を求める。治験依頼者又はその指名する者は、治験開始前（すなわち治験依頼者が提供する薬剤の搬入前）に治験実施計画書及び同意・説明文書に対する治験審査委員会の承認文書を入手する。治験審査委員会の承認文書には、当該治験について、治験課題名、治験実施計画書番号、作成／改訂日及びその他審査された文書（例：同意・説明文書）の版番号及び承認日が記載されていなければならない。治験依頼者が治験実施医療機関の規制文書の妥当性を確認した後に、薬剤を発送する。治験実施医療機関が薬剤を受領するまで、スクリーニング期等の治験実施計画書の手順を開始しない。

治験実施医療機関は、治験審査委員会が規定するすべての要件を遵守する。これには、治験実施計画書の改訂、同意・説明文書の改訂、被験者募集に関する資料の改訂、各地域の規制要件に従った安全性に関する報告、治験審査委員会によって設定された間隔での治験の実施状況報告及び治験終了報告の提出等、治験審査委員会への通知が含まれる。治験依頼者又はその指名する者は、上記の事項に関する治験審査委員会の承認文書及びその関連資料をすべて入手する。

被験者に支払われる負担軽減費は、参加に対する期待が過度にならない程度とする。負担軽減費は、治験審査委員会及び治験依頼者による承認を要する。

採取、保存された検体を用いたファーマコゲノミクス検討については、内容が定まった時点で解析する。その際に、治験依頼者はファーマコゲノミクス検討の研究計画書を作成し、治験依頼者において審査を実施する。

15.2 同意・説明文書及び被験者の同意

同意・説明文書には、ヘルシンキ宣言、医薬品の臨床試験の実施の基準に関するICH 三極統一ガイドライン及びすべての適用される法規制で求められている内容が、具体的に記載されている。同意・説明文書には、被験者の個人情報及び個人医療情報に関して、本治験における利用方法（国内外を問わず：第三者提供）、開示について記述されている。同意・説明文書には、治験の主旨、目的、起こり得る危険と利益が詳しく説明されている。また、同意・説明文書には治験参加に関する条件と被験者がいつでも理由を説明することなく、治療にあたって不利益を受けることなく治験参加を中止することができる旨が記載されている。

治験責任医師は、同意・説明文書の作成、内容、治験審査委員会の承認に関して責任を負う。同意・説明文書は、使用する前に治験審査委員会から承認を受ける。

同意・説明文書には、被験者が理解できる表現を使用する。治験責任医師又は治験分担医師は、同意・説明文書の内容を被験者に詳しく説明する責任がある。情報は可能な限り、口頭及び書面で、治験審査委員会が適切であると認める方法で提供する。被験者本人からの書面による同意取得が不可能な場合には、法令に従って被験者の代諾者から同意を取得する。

治験責任医師又は治験分担医師は、被験者又はその代諾者に(1)治験に関する問合せの機会及び(2)治験に参加するかどうかを決定するための十分な時間を与える。被験者又はその代諾者が治験への参加を決定した場合には、治験に参加する前に本人又はその代諾者は、同意書に署名又は記名・捺印し、日付を記載する。治験責任医師又は治験分担医師は、黒又は青のボールペンを用いて通称ではなく、正式な氏名で署名又は記名・捺印するよう被験者又はその代諾者に求める。治験責任医師又は治験分担医師も被験者が治験に参加する前に、同意書に署名又は記名・捺印、日付の記載を行う。

治験責任医師又は治験分担医師は、署名又は記名・捺印された同意書の原本を保管する。治験責任医師又は治験分担医師は、被験者が同意書に署名又は記名・捺印した日付を被験者の医療記録に記録する。また、署名又は記名・捺印入りの同意書の写しを被験者に提供する。

治験責任医師又は治験分担医師は、同意・説明文書を改訂した場合には、初回同意取得時と同様の手順で、当該被験者又はその代諾者から再度同意を取得する。再同意を取得した日付を被験者の医療記録に記録し、改訂した同意書の写しを被験者に提供する。

本治験の同意・説明文書を用いて説明を行った被験者に対して、「MLN0002 の治験におけるファーマコゲノミクス検討についての同意・説明文書」を用いてファーマコゲノミクス検討について説明する。本治験の同意及びファーマコゲノミクス検討についての同意を取得した被験者について、ファーマコゲノミクス検討のための検体採取を行う。

被験者が、保存している検体の廃棄を希望した場合には、9.4 項に従う。

15.3 被験者の機密保持

治験依頼者及びその指名する者は、プライバシーの侵害に対する被験者の権利保護の原則を遵守する。本治験では、被験者識別コードにより治験依頼者の臨床試験データベース及び治験関連文書と被験者の原データを関連付ける。被験者の特定及び被験者識別コードの正確性確認のため、すべての適用される法令及び規則の範囲内で、性別、年齢、生年月日等の限定的な被験者の情報を用いることができる。

ICH-GCP ガイドラインに従い、本治験実施計画書が遵守され、治験が実施されていることを確認するため、治験依頼者は、モニター又は治験依頼者が指名する者、各規制当局（例：FDA、MHRA、PMDA）の代表者、治験依頼者の指名する監査担当者及び治験審査委員会が、臨床検査成績、心電図、治験参加期間中の入退院記録、検死結果等の医療記録の原本（原データ又は資料）を閲覧することの許可を治験責任医師に要請する。治験責任医師又は治験分担医師は、被験者の同意を取得する際、モニター及び各規制当局の代表者等による医療記録原本の閲覧について、被験者から承諾を得る（15.2 項参照）。

治験依頼者に原資料の写しを提供する場合には、治験責任医師又は治験分担医師は、個人が特定できる情報（被験者氏名、住所、その他被験者の症例報告書に記録されない個人識別情報）を削除する。

15.4 公表、開示及び臨床試験登録の指針

15.4.1 公表及び開示

治験責任医師は、治験から得たすべての結果及びすべてのデータを治験依頼者に提供する。治験期間中は、法令又は規制で求められる場合を除き、治験依頼者だけが他の治験責任医師、治験分担医師又は規制当局に治験情報を開示することができる。治験契約書において他に許容されている場合を除いて、治験実施計画書、治験結果に関連した公開（公開ウェブサイトを含む）は、治験依頼者が責任を持って行う。

治験依頼者は、治験から得たデータ及び情報（治験責任医師から提供されたデータ及び情報を含む）のいずれも、治験責任医師の同意を得ることなく公表することができる。

治験責任医師又は治験分担医師は、本治験により得られた情報を専門の学会等外部に公表する場合には、事前に文書により治験依頼者の承諾を得る。

15.4.2 臨床試験登録

武田薬品工業株式会社は、臨床試験の情報をタイムリーに公表することを保証し、適用される法令、規制及び指針に準拠するため、世界各地で実施している患者対象の臨床試験はすべて、臨床試験開始前に、Clinical Trials.gov 及び公開ウェブサイト（JAPIC-CTI）に最低限登録する。治験を実施する市、州（米国の場合）、国及び被験者募集状況と共に武田薬品工業株式会社の問合せ先を登録し、一般閲覧を可能とする。

15.4.3 治験成績の開示

武田薬品工業株式会社は、結果に関係なく、適用される法令及び／又は規制で定めるところにより、Clinical Trials.gov 及び公開ウェブサイト（JAPIC-CTI）に臨床試験の成績を掲示する。

15.5 損害に対する保険／補償

本治験に参加している被験者は、健康被害が生じた場合には、被験者が参加している治験実施医療機関に適用される各地域の規制に従って補償される。治験を実施する地域で必要な場合には、治験依頼者又は指名された者は、被験者の健康被害の補償のため保険に加入する。

健康被害を生じた被験者への補償については、治験契約書に定めるところによる。治験責任医師又は治験分担医師は、補償に関して質問がある場合には、治験依頼者又は指名された者に連絡する。

16.0 参考文献

1) 「潰瘍性大腸炎·クローン病診断基準·治療指針」難治性炎症性腸管障害に関する調査研究班（渡辺班）平成 24 年度分担研究報告書　別冊

2) Soler D, Chapman T, Yang LL, Wyant T, Egan R, Fedyk ER. The binding specificity and selective antagonism of vedolizumab, an anti-alpha4beta7 integrin therapeutic antibody in development for inflammatory bowel diseases. J Pharmacol Exp Ther. 2009;330:864-75.

3) Edula RG, Picco MF. An evidence-based review of natalizumab therapy in the management of Crohn's disease. Ther Clin Risk Manag. 2009;5:935-42.

4) Tilg H, Kaser A. Vedolizumab, a humanized mAb against the α4β7 integrin for the potential treatment of ulcerative colitis and Crohn's disease. Curr Opin Investig Drugs. 2010;11:1295-304.

5) Parikh A, Leach T, Wyant T, Scholz C, Sankoh S, Mould DR, et al. Vedolizumab for the treatment of active ulcerative colitis: A randomized controlled phase 2 dose-ranging study. Inflamm Bowel Dis. 2012;18:1470-9.

6) Feagan BG, Greenberg GR, Wild G, Fedorak RN, Paré P, McDonald JW, et al. Treatment of ulcerative colitis with a humanized antibody to the alpha4beta7 integrin. N Engl J Med. 2005;352:2499-507.

7) Feagan BG, Greenberg GR, Wild G, Fedorak RN, Paré P, McDonald JW, et al. Treatment of active Crohn's disease with MLN0002, a humanized antibody to the alpha4beta7 integrin. Clin Gastroenterol Hepatol. 2008;6:1370-7.

8) Feagan BG, Rutgeerts P, Sands BE, Hanauer S, Colombel JF, Sandborn WJ, et al. Vedolizumab as induction and maintenance therapy for ulcerative colitis. N Engl J Med. 2013;369(8):699-710.

9) Sandborn WJ, Feagan BG, Rutgeerts P, Hanauer S, Colombel JF, Sands BE, et al. Vedolizumab as induction and maintenance therapy for Crohn's disease. N Engl J Med. 2013;369(8):711-21.

10) Sands B; Feagan B, Rutgeerts P, Colombel J; Sandborn W; Sy R, et al. Vedolizumab induction therapy for patients with Crohn’s disease and prior anti-TNF failure: a randomized, placebo-controlled, double-blind, multicenter trial. Presented at: Crohn’s & Colitis Foundation of America – Advances in Inflammatory Bowel Diseases Clinical and Research Conference. December 13-15, 2012. Hollywood, FL.

11) 小林清典、鈴木康夫、渡辺憲治、迎美幸、山田哲弘、山上博一. VDZ の日本人 UC 患者を対象とした第 1 相多施設共同非盲検用量漸増反復投与試験. 第 21 回日本消化器関連学会週間、東京、2013

12) Kappos L, Bates D, Edan G, Eraksoy M, Garcia-Merino A, Grigoriadis N, et al. Natalizumab treatment for multiple sclerosis: updated recommendations for patient selection and monitoring. Lancet Neurol. 2011;10:745-58.

13) 潰瘍性大腸炎の診断. 久部高司, 平井郁仁, 松井敏幸. 日本大腸肛門病会誌 64：807—816，2011

付録 A　治験スケジュール（スクリーニング・導入期・維持期）

		スクリーニング	導入期				維持期													最終投与16 週後	予定外の来院[l]
試験手順	Week[a]		0	2	6	10/ET[j]	14	18	22	26	30	34	38	42	46	50	54	58	60/ET[k]		
試験手順	Day[a]	-21〜-3[i]	1	15	43	71/ET[j]	99	127	155	183	211	239	267	295	323	351	379	407	421/ET[k]		
試験手順	許容範囲（日）[a]	—	—	±3	±3	±3/ET[j]	±7	±7	±7	±7	±7	±7	±7	±7	±7	±7	±7	-7 ~ +6	±7/ET[k]	±14	
試験手順	VISIT	1	2	3	4	5/ET[j]	6	7	8	9	10	11	12	13	14	15	16	17	18/ET[k]	-	
	同意取得	X																			
	選択基準・除外基準	X[m]	X																		
	人口統計学的データ、身長、既往歴、前治療薬、合併症	X[m]																			
	無作為化		X				X														
	治験薬薬剤番号の発番/投与		X	X	X		X		X		X		X		X		X				
	診察、バイタルサイン[b]	X[m]	X	X	X	X	X	X	X	X	X	X	X	X	X	X	X	X	X	X	
	体重[b]	X[m]	X	X	X	X	X	X	X	X	X	X	X	X	X	X	X	X	X	X	
	心電図[b]	X[m]	X			X													X	X	
	胸部画像検査[c]	X[m]																		X	
	結核菌検査	X[n]																			
	PML チェックリスト[b]	X[m]	X	X	X		X		X		X		X		X		X			X	
臨床検査[b]	血液生化学検査	X[n]	X	X	X	X	X		X		X		X		X		X		X	X	
臨床検査[b]	血液学的検査	X[n]	X	X	X	X	X		X		X		X		X		X		X	X	
臨床検査[b]	尿検査	X[n]	X			X	X												X	X	
臨床検査[b]	尿妊娠検査[d]	X[m]	X	X	X	X	X		X		X		X		X		X		X	X	
臨床検査[b]	HBs 抗原、HCV 抗体、HIV 抗原・抗体検査	X[n]																			
臨床検査[b]	炎症性マーカー検査	X[n]	X	X	X	X	X		X		X		X		X		X		X		
	完全Mayoスコア[e]		X[o]			X[p]													X[p]		
	部分Mayoスコア	X[m]		X	X		X	X	X	X	X	X	X	X	X	X	X	X			X
	患者日誌の確認		X	X	X	X	X	X	X	X	X	X	X	X	X	X	X	X	X		X
	IBDQ[b]		X			X	X						X						X		
	UC 関連イベント			X	X	X	X	X	X	X	X	X	X	X	X	X	X	X	X	X	X
	HAHA・中和抗体測定用採血[f]		X			X					X								X	X	
	PK測定用採血[f]			X	X	X	X		X		X								X		
	PGx検討用検体の採血 [g]		X																		
	併用薬・併用療法	X[m]	X	X	X	X	X	X	X	X	X	X	X	X	X	X	X	X	X	X	X
	PTE/有害事象[h]	X[m]	X	X	X	X	X	X	X	X	X	X	X	X	X	X	X	X	X	X	X

付録 A　治験スケジュール（非盲検コホート）

		非盲検コホート													
試験手順	**Week**[q]	**0x**	**2x**	**6x**	**10x**	**14x**	**18x**	**22x**	**26x**	**30x**	**34x**	**38x**	**42x**	**46x**	**50x**
	**Day**[q]	**1x**	**15x**	**43x**	**71x**	**99x**	**127x**	**155x**	**183x**	**211x**	**239x**	**267x**	**295x**	**323x**	**351x**
	**許容範囲（日）**[q]	—	**±3**	**±3**	**±3**	**±7**	**±7**	**±7**	**±7**	**±7**	**±7**	**±7**	**±7**	**±7**	**±7**
	**VISIT**[q]	**1x**	**2x**	**3x**	**4x**	**5x**	**6x**	**7x**	**8x**	**9x**	**10x**	**11x**	**12x**	**13x**	**14x**
同意取得															
選択基準・除外基準															
人口統計学的データ、身長、既往歴、前治療薬、合併症															
無作為化															
治験薬薬剤番号の発番/投与		X[s]	X	X		X		X		X		X		X	
診察、バイタルサイン[b]			X	X	X	X	X	X	X	X	X	X	X	X	X
体重[b]			X	X	X	X	X	X	X	X	X	X	X	X	X
心電図[b]															
胸部画像検査[c]															
結核菌検査															
PML チェックリスト[b]		X[s]	X	X		X		X		X		X		X	
臨床検査[b]	血液生化学検査		X	X		X		X		X		X		X	
	血液学的検査		X	X		X		X		X		X		X	
	尿検査														
	尿妊娠検査[d]		X	X		X		X		X		X		X	
	HBs 抗原、HCV 抗体、HIV 抗原・抗体検査														
	炎症性マーカー検査		X	X		X		X		X		X		X	
完全 Mayo スコア[e]															
部分Mayoスコア			X	X	X	X	X	X	X	X	X	X	X	X	X
患者日誌の確認			X	X	X	X	X	X	X	X	X	X	X	X	X
IBDQ[b]															
UC 関連イベント			X	X	X	X	X	X	X	X	X	X	X	X	X
HAHA・中和抗体測定用採血[f]					X					X					
PK測定用採血[f]															
PGx検討用検体の採血[g]															
併用薬・併用療法		X	X	X	X	X	X	X	X	X	X	X	X	X	X
PTE/有害事象[h]		X	X	X	X	X	X	X	X	X	X	X	X	X	X

次頁に続く

付録 A　　治験スケジュール（非盲検コホート; 続き）

試験手順		非盲検コホート											最終投与16 週後	予定外の来院[l]
	Week[q]	54x	58x	62x	66x	70x	74x	78x	82x	86x	90x	94x/ET[r]		
	Day[q]	379x	407x	435x	463x	491x	519x	547x	575x	603x	631x	659x/ET[r]		
	許容範囲（日）[q]	±7	±7	±7	±7	±7	±7	±7	±7	±7	±7	±7/ET[r]	±14	
	VISIT[q]	15x	16x	17x	18x	19x	20x	21x	22x	23x	24x	25x/ET[r]	-	
同意取得														
選択基準・除外基準														
人口統計学的データ、身長、既往歴、前治療薬、合併症														
無作為化														
治験薬薬剤番号の発番/投与		X		X		X		X		X		X[t]		
診察、バイタルサイン[b]		X	X	X	X	X	X	X	X	X	X	X	X	
体重[b]		X	X	X	X	X	X	X	X	X	X	X	X	
心電図[b]												X	X	
胸部画像検査[c]													X	
結核菌検査														
PML チェックリスト[b]		X		X		X		X		X		X	X	
臨床検査[b]	血液生化学検査	X		X		X		X		X		X	X	
	血液学的検査	X		X		X		X		X		X	X	
	尿検査											X	X	
	尿妊娠検査[d]	X		X		X		X		X		X	X	
	HBs 抗原、HCV 抗体、HIV 抗原・抗体検査													
	炎症性マーカー検査	X		X		X		X		X		X		
完全 Mayo スコア[e]														
部分Mayoスコア		X	X	X	X	X	X	X	X	X	X	X		X
患者日誌の確認		X	X	X	X	X	X	X	X	X	X	X		X
IBDQ[b]														
UC 関連イベント		X	X	X	X	X	X	X	X	X	X	X	X	X
HAHA・中和抗体測定用採血[f]				X								X	X	
PK測定用採血[f]														
PGx検討用検体の採血[g]														
併用薬・併用療法		X	X	X	X	X	X	X	X	X	X	X	X	X
PTE/有害事象[h]		X	X	X	X	X	X	X	X	X	X	X	X	X

ET=中止時、PK=薬物動態、IBDQ=炎症性腸疾患質問票、HAHA=ヒト抗ヒト抗体、PTE=Pretreatment Event
a. 導入期における治験薬初回投与日を Week 0 (Day 1)として起算する。許容範囲は予定来院日からのずれとして記載
b. 治験薬投与日の場合は、治験薬投与前に実施する
c. 3 ヵ月以内に撮影した検査画像が入手できる場合は実施しない（スクリーニング期は同意取得前の画像でも可）
d. 妊娠する可能性のある女性のみ
e. 完全 Mayo スコアのための内視鏡検査は、評価日の 9 日前から評価当日までに実施する
f. 治験薬投与を行う来院日の場合は投与直前に採取する。治験薬投与を行わない来院日の場合は任意のタイミングで採取する。本検査は、冷却遠心機のある治験実施医療機関でのみ実施する
g. 同意が得られた患者のみ実施する。VISIT 2 以降、治験終了時までの期間のうち、できる限り早い時期に 1 回実施する
h. 治験薬初回投与前は PTE、治験薬初回投与後は有害事象を収集する
i. 結核の既往又はその疑いが認められる者で、組み入れに先立ってイソニアジドの予防投与を実施する場合は、スクリーニング期間を Day -28～Day -3 に延長できる。治験薬投与はイソニアジドの予防投与を 21 日以上行った後に開始する
j. 治験薬の投与を受けた被験者が、導入期において中止した場合は、可能な限り速やかに来院のうえ、中止時の検査を実施する
k. 維持期において中止した場合は、可能な限り速やかに来院のうえ、中止時の検査を実施する。非盲検コホートへの組み入れ適格例については、維持期の最終投与から 3～9 週間の間隔を空けたうえで、非盲検コホートへの組み入れを行う
l. UC の悪化等で予定外の来院があった場合に実施する。必要に応じて検査を追加する
m. Day -21～Day -1 での実施を許容する。なお結核の既往又はその疑いが認められる者で、組み入れに先立ってイソニアジドの予防投与を実施する場合は、Day -28～Day -1 での実施を許容する。治験薬投与はイソニアジドの予防投与を 21 日以上行った後に開始する
n. VISIT 2 の来院日までに検査結果を入手できるようにする
o. 同意取得前に実施された評価日前 9 日以内の内視鏡検査結果がある場合は、その利用を可とする
p. 中止時の検査の場合は、部分 Mayo スコアでも可
q. 非盲検コホート組み入れ時を Week 0x (Day 1x) とし、そのときの来院を VISIT 1x として起算する。許容範囲は予定来院日からのずれとして記載
r. 非盲検コホートにおいて中止した場合は、可能な限り速やかに来院のうえ、中止時の検査を実施する。最終被験者が非盲検コホートに組み入れられてから 46 週後の時点に計画している治験全体の終了時も同様の検査・観察･評価を行う
s. 非盲検コホート組み入れ後に実施する
t. 中止時の検査の場合は、治験薬薬剤番号の発番/投与は実施しない

付録 B　治験責任医師の責務

1. 本治験実施計画書及び GCP を遵守し、被験者の人権、安全及び福祉に配慮して適切に治験を実施する。
2. 治験関連の重要な業務の一部を治験分担医師又は治験協力者に分担させる場合には、分担させる業務と分担させる者のリストを作成し、あらかじめ治験実施医療機関の長に提出し、その了承を受ける。
3. 同意・説明文書を作成し、必要に応じて改訂する。
4. 治験の契約内容を確認する。
5. 治験分担医師及び治験協力者等に治験実施計画書、薬剤及び各人の業務について十分な情報を与え、指導及び監督する。
6. 本治験実施計画書に合致した被験者を選定し、文書により説明し、文書で同意を取得する。
7. 治験に関連する医療上のすべての判断に責任を負う。
8. 被験者の治験参加期間中及びその後を通じ、治験に関連した臨床上の問題となるすべての有害事象に対して、治験実施医療機関の長と共に十分な医療が被験者に提供されることを保証する。
9. 被験者が他の医療機関や他の診療科を受診している場合には、被験者の同意のもとに、当該医療機関又は他の診療科の医師に被験者の治験への参加を、また治験の終了・中止について文書で連絡するとともに、その記録を作成する。
10. 重篤な有害事象等の緊急報告が必要な場合には、治験実施医療機関の長及び治験依頼者に直ちに文書にて報告する。
11. 緊急時に該当症例のエマージェンシー・キーの開封の必要性について判断する
12. 正確かつ完全な症例報告書を作成し、電子署名のうえ、治験依頼者に提出する。
13. 治験分担医師が作成、又は治験協力者が原データから転記した症例報告書の記載内容を点検、確認し、電子署名のうえ、治験依頼者に提出する。
14. 治験依頼者から治験実施計画書等の改訂について提案があった場合には協議する。
15. 治験が終了した場合には、治験実施医療機関の長に文書で報告する。

付録 C　濱瘍性大腸炎診断基準（平成 25 年 1 月 17 日改訂）

1. 定義

主として粘膜を侵し、しばしばびらんや潰瘍を形成する大腸の原因不明のびまん性非特異性炎症である。WHOのCouncil for International Organization of MedicalScience（CIOMS）医科学国際組織委員会で定められた名称と概念は、つぎの通りである。（1973）

特発性大腸炎　idiopathic proctocolitis
An idiopathic, non-specific inflammatory disorder involving primarily the mucosa and submucosa of the colon, especially the rectum. It appears mainly in adults under the age of 30, but may affect children and adults over the age of 50.Its aetiology remains unknown, but immunopathological mechanisms and predisposing psychological factors are believed to be involved. It usually produces a bloody diarrhoea and various degrees of systemic involvement,liability to malignant degeneration, if of long duration and affecting the entire colon.

（訳）主として粘膜と粘膜下層をおかす、大腸とくに直腸の特発性、非特異性の炎症性疾患。30歳以下の成人に多いが、小児や50歳以上の年齢層にもみられる。原因は不明で、免疫病理学的機序や心理学的要因の関与が考えられている。通常血性下痢と種々の程度の全身症状を示す。長期にわたり、かつ大腸全体をおかす場合には悪性化の傾向がある。

2. 診断の手順

慢性の粘血・血便などがあり本症が疑われるときには、放射線照射歴、抗生剤服用歴、海外渡航歴などを聴取するとともに、細菌学的・寄生虫学的検査を行って感染性腸炎を除外する。次に直腸あるいはS状結腸内視鏡検査を行って本症に特徴的な腸病変を確認する。このさい、生検を併用する。これだけの検査で多くは診断が可能であるが、必要に応じて注腸X線検査や全大腸内視鏡検査などを行って、腸病変の性状や程度、罹患範囲などを検査し、同時に他の疾患を除外する。

3. 診断基準

次のa）のほか、b）のうちの1項目、およびc）を満たし、下記の疾患が除外できれば、確診となる。

a）臨床症状：持続性または反復性の粘血・血便、あるいはその既往がある。
b）①内視鏡検査：ｉ）粘膜はびまん性におかされ、血管透見像は消失し、粗ぞうまたは細顆粒状を呈する。さらに、もろくて易出血性（接触出血）を伴い、粘血膿性の分泌物が付着しているか、ⅱ）多発性のびらん、潰瘍あるいは偽ポリポーシスを認める。
　②注腸X線検査：ｉ）粗ぞうまたは細顆粒状の粘膜表面のびまん性変化、ⅱ）多発性のびらん、潰瘍、ⅲ）偽ポリポーシスを認める。その他、ハウストラの消失（鉛管像）や腸管の狭小・短縮が認められる。
c）生検組織学的検査： 活動期では粘膜全層にびまん性炎症性細胞浸潤、陰窩膿瘍、高度な杯細胞減少が認められる。いずれも非特異的所見であるので、総合的に判断する。寛解期では腺の配列異常（蛇行・分岐）、萎縮が残存する。上記変化は通常直腸から連続性に口側にみられる。

b）c）の検査が不十分、あるいは施行できなくとも切除手術または剖検により、肉眼的および組織学的に本症に特徴的な所見を認める場合は、下記の疾患が除外できれば、確診とする。

除外すべき疾患は、細菌性赤痢、アメーバ性大腸炎、サルモネラ腸炎、キャンピロバクタ腸炎、大腸結核、クラミジア腸炎などの感染性腸炎が主体で、その他にクローン病、放射線照射性大腸炎、薬剤性大腸炎、リンパ濾胞増殖症、虚血性大腸炎、腸型ベーチェットなどがある。

〈注1〉 まれに血便に気付いていない場合や、血便に気付いてすぐに来院する（病悩期間が短い）場合もあるので注意を要する。

〈注2〉 所見が軽度で診断が確実でないものは「疑診」として取り扱い、後日再燃時などに明確な所見が得られた時に本症と「確診」する。

〈注3〉 Indeterminate colitis
クローン病と潰瘍性大腸炎の両疾患の臨床的、病理学的特徴を合わせ持つ、鑑別困難例。経過観察により、いずれかの疾患のより特徴的な所見が出現する場合がある。

4. 病態（病型・病期・重症度）

A. 病変の拡がりによる病型分類

全大腸炎	total colitis
左側大腸炎	left-sided colitis
直腸炎	proctitis
右側あるいは区域性大腸炎	right-sided or segmental colitis

〈注4〉 左側大腸炎は、病変の範囲が脾彎曲部を越えていないもの。

〈注5〉 直腸炎は、前述の診断基準を満たしているが、内視鏡検査により直腸S状部（RS）の口側に正常粘膜を認めるもの。

〈注6〉 右側あるいは区域性大腸炎は、クローン病や大腸結核との鑑別が困難で、診断は経過観察や切除手術または剖検の結果を待たねばならないこともある。

〈注7〉 胃十二指腸にびまん性炎症が出現することがある。

B. 病期の分類

活動期 active stage
寛解期 remission stage

〈注8〉 活動期は血便を訴え、内視鏡的に血管透見像の消失、易出血性、びらん、または潰瘍などを認める状態。

〈注9〉 寛解期は血便が消失し、内視鏡的には活動期の所見が消失し、血管透見像が出現した状態。

C. 臨床的重症度による分類

軽　症 mild
中等症 moderate
重　症 severe

診断基準は下記の如くである。

	重　症	中等症	軽　症
1）排便回数	6回以上	重症と軽症との中間	4回以下
2）顕血便	（＋＋＋）		（＋）～（－）
3）発熱	37.5℃以上		（－）
4）頻脈	90/分以上		（－）
5）貧血	Hb10g/dL以下		（－）
6）赤沈	30mm/h以上		正常

〈注10〉 軽症の3）、4）、5）の（－）とは37.5℃以上の発熱がない。90/分以上の頻脈がない。Hb10g/dL以下の貧血がない、ことを示す。
〈注11〉 重症とは1）および2）の他に全身症状である3）または4）のいずれかを満たし、かつ6項目のうち4項目以上を満たすものとする。軽症は6項目すべて満たすものとする。
〈注12〉 左記の重症と軽症との中間にあたるものを中等症とする。
〈注13〉 重症の中でも特に症状が激しく重篤なものを劇症とし、発症の経過により、急性劇症型と再燃劇症型に分ける。劇症の診断基準は以下の5項目をすべて満たすものとする。
①重症基準を満たしている。
②15回/日以上の血性下痢が続いている。
③38℃以上の持続する高熱がある。
④10,000/mm3以上の白血球増多がある。
⑤強い腹痛がある。

D. 活動期内視鏡所見による分類

軽　度　mild
中等度　moderate
強　度 severe

診断基準は下表の如くである。

炎　症	内視鏡所見
軽　度	血管透見像消失<br>粘膜細顆粒状<br>発赤、アフタ、小黄色点
中等度	粘膜粗ぞう、びらん、小潰瘍<br>易出血性（接触出血）<br>粘血膿性分泌物付着<br>その他の活動性炎症所見
強　度	広汎な潰瘍<br>著明な自然出血

〈注14〉内視鏡的に観察した範囲で最も所見の強いところで診断する。内視鏡検査は前処置なしで短時間に施行し、必ずしも全大腸を観察する必要はない。

E. 臨床経過による分類

再燃寛解型	relapse-remitting type
慢性持続型	chronic continuous type
急性劇症型（急性電撃型）	acute fulminating type
初回発作型	first attack type

〈注15〉慢性持続型は初回発作より6 ヶ月以上活動期にあるもの。
〈注16〉急性劇症型（急性電撃型）はきわめて激烈な症状で発症し、中毒性巨大結腸症、穿孔、敗血症などの合併症を伴うことが多い。
〈注17〉初回発作型は発作が1回だけのもの、しかし将来再燃をきたし、再燃寛解型となる可能性が大きい。

F. 病変の肉眼所見による特殊型分類

偽ポリポーシス型
萎縮性大腸炎型

G. 治療反応性に基づく難治性潰瘍性大腸炎の定義

1. 厳密なステロイド療法にありながら、次のいずれかの条件を満たすもの。
①ステロイド抵抗例（プレドニゾロン1-1.5mg/kg/日の1-2週間投与で効果がない）
②ステロイド依存例（ステロイド漸減中の再燃）
2. ステロイド以外の厳密な内科的治療下にありながら、頻回に再燃をくりかえすあるいは慢性持続型を呈するもの。

H. 回腸嚢炎の診断基準

Ⅰ. 概念
回腸嚢炎（pouchitis）は、自然肛門を温存する大腸（亜）全摘術を受けた患者の回腸嚢に発生する非特異的炎症である。原因は不明であるが、多くは潰瘍性大腸炎術後に発生し、家族性大腸腺腫症術後の発生は少ないことより、潰瘍性大腸炎の発症機序との関連が推定されている。

Ⅱ. 回腸嚢炎の診断
1. 項目
a）臨床症状
1）排便回数の増加　2）血便　3）便意切迫または腹痛　4）発熱（37.8℃以上）
b）内視鏡検査所見
軽　度：浮腫、顆粒状粘膜、血管透見像消失、軽度の発赤
中等度：アフタ、びらん、小潰瘍＃、易出血性、膿性粘液
重　度：広範な潰瘍、多発性潰瘍＃、びまん性発赤、自然出血
＃：staple line ulcerのみの場合は、回腸嚢炎の内視鏡所見とは区別して所見を記載する。

2. 診断基準
少なくとも1つの臨床症状を伴い中等度以上の内視鏡所見を認める場合。また、臨床症状に関わらず内視鏡的に重症の所見を認める場合は回腸嚢炎と診断する。除外すべき疾患は、感染性腸炎（サルモネラ腸炎、キャンピロバクタ腸炎、腸結核などの細菌性腸炎、サイトメガロウィルス腸炎などのウィルス腸炎、寄生虫疾患）、縫合不全、骨盤内感染症、術後肛門機能不全、クローン病などがある。

付録 D PML チェックリスト

- 治験実施計画書に規定の来院時あるいは患者が症状を訴えた場合に実施する。
- 本チェックリストの目的は、以前の評価からの患者の神経学的な明確な変化を同定することである。
- 自覚症状が同定された場合、対応する他覚所見のテストを行わなければならない。本チェックリストで得られたすべての症状・所見は有害事象として記録すること。
- 神経症状に明確な変化があった場合、神経科医を受診させること。必要であれば、造影剤を用いた MRI 等を行い、神経症状の変化の裏付け情報を収集すること。
- 自覚症状あるいは他覚所見で陽性になった場合は、直ちに治験依頼者に連絡すること。

症例評価アルゴリズム

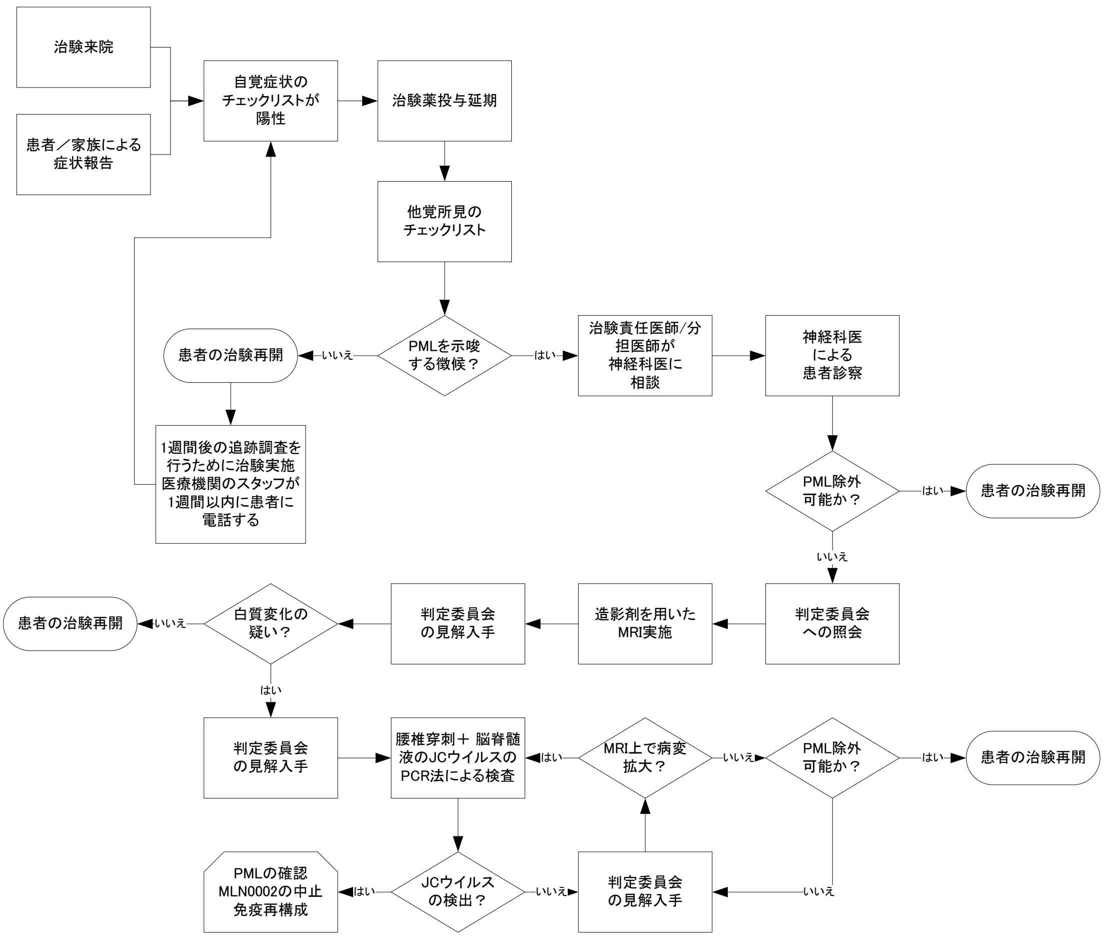

PML チェックリスト（自覚症状）

実施日：	年　　月　　日
被験者識別コード：	

症状	以下の中で、いつもと有意に異なるものはありますか？		「はい」の場合、具体的な症状を記載してください。	適用される客観的テスト:PML 客観的チェックリストに結果を記録
	はい	いいえ		
1. 継続的な視力低下、複視を最近経験しましたか？読書障害を最近経験しましたか？				視野と眼球運動をテストしてください。
2. 継続的にうまくしゃべることができなかったり、あなたの会話が他者に理解してもらえなかったりしたことが続いたことが最近ありましたか？				構音障害もしくは失語症について発話に対する自然な観察を行ってください。いくつかのものの名称を尋ね、複数の単語からなるフレースを繰り返すように患者に依頼してください。
3. 継続的な腕や足の筋力の低下を最近感じましたか？				回内筋偏移テスト（Barré 手技）及び／又は腕を回転させたまま保持できるかのテスト。片足跳びできるかどうか;足と指でタッピングできるかどうか。対称性筋力テスト。
4. よくものにぶつかったり、字を書くのが難しく感じたりしたことが最近ありますか？				サンプルの自発書字を求め、指鼻テスト、踵脛テスト、継足歩行（一方のかかとにもう一方のつま先をつけて一直線上を歩く方法）を観察する。

実施日： 年 月 日	
被験者識別コード：	

症状	以下の中で、いつもと有意に異なるものはありますか？		「はい」の場合、具体的な症状を記載してください。	適用される客観的テスト:PML 客観的チェックリストに結果を記録
	はい	いいえ		
5. 他者が言っていることが理解できなかったことが最近しばしばありましたか？				一連の指示に従う能力（目を閉じて、舌を突き出して、左の指で左耳を触る。）
6. 記憶や思考で継続的な問題はありますか？				1 分以上気をそらした後、3 つの対象を思い出させる。; 指示に従う能力。
7. 継続的な感覚鈍麻やその他の感覚の喪失などを経験しましたか？				隣り合う針刺し刺激で感覚をテストする。

チェックリスト実施者（署名または記名・捺印）

＿＿＿＿＿＿＿＿＿＿＿＿＿ 印 ＿＿＿＿ 年 ＿＿ 月 ＿＿ 日

PML チェックリスト（他覚所見）

自覚症状のある患者を対象に実施してください。

自覚症状のある項目に対応した他覚所見のテストを実施してください。

実施日： 年 月 日	
被験者識別コード：	

陽性の自覚症状	適用される客観的テスト	テスト結果		もし異常が見られた場合は、以下に簡潔に記載してください。
		正常	異常	
1.<br>視覚及び読書の困難	視野と眼球運動をテストしてください			
2.<br>発話困難	構音障害もしくは失語症について発話に対する自然な観察を行って下さい。いくつかのものの名称を尋ね、複数の単語からなるフレースを繰り返すように患者に依頼してください。			
3.<br>上肢又は下肢の筋力低下	回内筋偏移テスト（Barré 手技）及び／又は腕を回転させたまま保持できるかのテスト。片足跳びできるかどうか；足と指でタッピングできるかどうか。対称性筋力テスト。			
4.<br>物への衝突又は書字困難	サンプルの自発書字を求める。指鼻テスト、踵脛テスト、継足歩行（一方のかかとにもう一方のつま先をつけて一直線上を歩く方法）を観察する。			

実施日： 年 月 日	
被験者識別コード：	

陽性の自覚症状	適用される客観的テスト	テスト結果		もし異常が見られた場合は､以下に簡潔に記載してください。
		正常	異常	
5.<br>他者の理解困難	一連の指示に従う能力（目を閉じて、舌を突き出して、左の指で左耳を触る。）			
6.<br>記憶あるいは思考の問題	1 分以上気をそらした後、3 つの対象を思い出させる。；指示に従う能力。			
7.<br>感覚鈍麻の問題	隣り合う針刺し刺激で感覚をテストする。			

- 報告された症状が客観的テストによって確認された場合、患者に神経内科医を受診させてください。その他の場合は、客観的チェックリストを実施した 1 週間後に被験者を追跡調査し、報告された自覚症状が再現されないことを確認してください。

- 客観的チェックリストの陽性所見は何でも、治験依頼者に連絡してください。

チェックリスト実施者（署名または記名・捺印）

____________________ 印 ______ 年 月 日

付録 E 追跡問診用紙

被験者識別コード : ________________________________
治験薬の最終投与からの経過月数 :

6 ヵ月☐ 12 ヵ月☐ 18 ヵ月☐ 24 ヵ月☐

追跡問診実施日 : 20____年____月____日

	はい	いいえ
1. 前回の治験連絡/来院以降、大腸の dysplasia（前癌病変のひとつ）、癌、リンパ腫、あるいはその他の種類の癌と診断されましたか?<br>「はい」の場合、次の質問に答えてください<br>• 大腸の dysplasia はい/いいえ 診断日 : 20___/___/____<br>• 大腸の癌 はい/いいえ 診断日 : 20___/___/____<br>• リンパ腫 はい/いいえ 診断日 : 20___/___/____<br>• その他 : （明記） はい/いいえ 診断日 : 20___/___/____	☐	☐
2. 前回の治験連絡/来院以降、進行性多巣性白質脳症（PML としても知られています）と診断されましたか?<br>「はい」の場合、次の質問に答えてください<br>• 診断日 : 20___/___/____	☐	☐
3. 前回の治験連絡/来院以降、腸管切除を受けましたか?<br>「はい」の場合、次の質問に答えてください<br>• 大腸切除 はい/いいえ 診断日 : 20___/___/____<br>• 小腸切除 はい/いいえ 診断日 : 20___/___/____	☐	☐
以下 3 つの追跡問診項目については治験薬最終投与 6 ヵ月後の 1 時点でのみ実施する		
4. 前回の治験来院以降、感染症と診断され、入院を必要としましたか?<br>「はい」の場合、次の質問に答えてください<br>• 入院/診断の理由を明記してください 理由 : ______________________________<br>• 診断日 : 20___/___/____	☐	☐
5. 女性被験者に対して : 前回の治験来院以降、妊娠しましたか?<br>「はい」の場合、次の質問に答えてください<br>• 妊娠報告書に記入する必要があります<br>• 出産予定日を明記してください 予定日 : 20___/___/____	☐<br>該当せず ☐	☐
6. 男性被験者に対して : 前回の治験来院以降、女性パートナーが妊娠しましたか?<br>「はい」の場合、次の質問に答えてください<br>• 妊娠報告書に記入する必要があります<br>• 出産予定日を明記してください 予定日 : 20___/___/____	☐<br>該当せず ☐	☐

治験責任医師（署名または記名・捺印）

______________________________印

20　　年　　月　　日
