# Supplementary material for: Vedolizumab in Japanese patients with ulcerative colitis: A Phase 3, randomized, double-blind, placebo-controlled study
Source: PLoS One. 2019 Feb 26;14(2):e0212989. doi: 10.1371/journal.pone.0212989 (PMC6391030; doi:10.1371/journal.pone.0212989)
Supplement: S3 File — (PDF) [file pone.0212989.s006.pdf]

TAKEDA PHARMACEUTICALS

PROTOCOL

<Title>

Phase 3, multicenter, randomized, double-blinded, placebo-controlled, parallel-group study to evaluate the efficacy, safety, and pharmacokinetics of intravenous MLN0002 (300 mg) infusion in induction and maintenance therapy in Japanese subjects with moderate or severe ulcerative colitis

<Short Title>

Phase 3 study of MLN0002 (300 mg) in treatment of ulcerative colitis

**Sponsor:**	Takeda Pharmaceutical Company Limited<br>1-1, Doshomachi 4-chome, Chuo-ku, Osaka-shi, Osaka		
**Study Number:**	MLN0002/CCT-101		
**Version Number:**	Initial version		
**IND Number:**	Not Applicable	**EudraCT Number:**	Not Applicable
**Compound:**	Vedolizumab		
**Date:**	November 11, 2013		

1.0 ADMINISTRATIVE INFORMATION AND PRINCIPLES OF CLINICAL STUDIES

1.1 Contacts and Responsibilities of Study-Related Activities

See the Annex.

1.2 Principles of Clinical Studies

This study will be conducted with the highest respect for the individual participants in accordance with the requirements of this study protocol and also in accordance with the following:

- The ethical principles that have their origin in the Declaration of Helsinki.
- International Conference on Harmonisation E6 Good Clinical Practice: Consolidated Guideline.
- All applicable laws and regulations, including, without limitation, data privacy laws, clinical trial disclosure laws, and regulations.

TABLE OF CONTENTS

1.0 ADMINISTRATIVE INFORMATION AND PRINCIPLES OF CLINICAL STUDIES 2
1.1 Contacts and Responsibilities of Study-Related Activities 2
1.2 Principles of Clinical Studies 3
2.0 STUDY SUMMARY 10
3.0 LIST OF ABBREVIATIONS 15
3.1 Definitions in the Study 16
4.0 INTRODUCTION 17
4.1 Background 17
4.2 Outcome of Clinical Studies 17
4.2.1 Summary of Outcome of Phase 1 and 2 Studies Outside Japan 18
4.2.2 Summary of Outcome of Phase 3 Studies Outside Japan 18
4.2.3 Summary of Outcome of Phase 1 Study in Japan 19
4.3 Rationale for the Proposed Study 19
5.0 STUDY OBJECTIVES AND ENDPOINTS 20
5.1 Objectives 20
5.1.1 Primary Objective for the Induction Phase 20
5.1.2 Secondary Objective for the Induction Phase 20
5.1.3 Primary Objective for the Maintenance Phase 20
5.1.4 Secondary Objective for the Maintenance Phase 20
5.2 Endpoints 20
5.2.1 Efficacy Endpoints for the Induction Phase 20
5.2.1.1 Primary Endpoint 20
5.2.1.2 Secondary Endpoints 20
5.2.1.3 Exploratory Endpoints 20
5.2.2 Efficacy Endpoints for the Maintenance Phase 21
5.2.2.1 Primary Endpoint 21
5.2.2.2 Secondary Endpoints 21
5.2.2.3 Exploratory Endpoints 21
5.2.3 Pharmacokinetic Endpoint 21
5.2.4 Safety Endpoints 21
5.2.5 Immunogenicity Endpoint 22
6.0 STUDY DESIGN AND DESCRIPTION 23
6.1 Study Design 23

6.2 Justification for Study Design, Dose, and Endpoints 25
6.2.1 Justification for Study Population....... 25
6.2.2 Justification of Study Design and Sample Size 26
6.2.3 Justification of the Placebo Group....... 26
6.2.4 Justification of the Dosage and the Evaluation Period 26
6.2.5 Justification of Endpoints....... 27
6.3 Premature Termination or Suspension of Study or Investigational site 28
6.3.1 Criteria for Premature Termination or Suspension of the Study....... 28
6.3.2 Criteria for Premature Termination or Suspension of Investigational Sites 28
6.3.3 Procedures for Premature Termination or Suspension of the Study or the Participation of Investigational Site(s)....... 28
7.0 SELECTION AND DISCONTINUATION/WITHDRAWAL OF SUBJECTS....... 29
7.1 Inclusion Criteria 29
7.2 Exclusion Criteria 30
7.3 Excluded Medications and Treatments....... 33
7.3.1 Rescue Treatments....... 34
7.3.2 Dose Reduction Schedule of Corticosteroids....... 35
7.4 Subject Management....... 35
7.5 Criteria for Discontinuation or Withdrawal of a Subject....... 36
7.6 Procedures for Discontinuation or Withdrawal of a Subject....... 38
8.0 CLINICAL STUDY MATERIAL MANAGEMENT....... 39
8.1 Study Medication and Materials....... 39
8.1.1 Dosage Form, Manufacturing, Packaging, and Labeling....... 39
8.1.1.1 Study Drug 39
8.1.1.2 Materials 40
8.1.2 Storage....... 41
8.1.3 Dose and Regimen 41
8.1.4 Overdose....... 41
8.2 Procedures for Allocation, Prescription and Infusion of Study Drugs 42
8.3 Preparation and Retention of the Allocation Table 42
8.4 Preservation of Blindness....... 42
8.5 Procedures for Blind-Breaking....... 42
8.6 Control and Disposal of Sponsor-Supplied Drugs 43
9.0 STUDY PLAN 44
9.1 Study Procedures 44

9.1.1 Informed Consent Procedure....44
9.1.2 Demographics, Medical History, and Medication History Procedure....44
9.1.3 Physical Examination Procedure....45
9.1.4 Weight, Height and BMI....45
9.1.5 Vital Sign Procedure....45
9.1.6 Documentation of Concomitant Medications and Non-drug Therapy....46
9.1.7 Documentation of Concurrent Medical Conditions....46
9.1.8 The Patient Diary....46
9.1.9 Complete Mayo Score and Partial Mayo Score....47
9.1.9.1 Calculation of Subscores for Stool Frequency and Rectal Bleeding....48
9.1.9.2 Calculation of the Subscore for Findings on Endoscopy....48
9.1.9.3 Calculation of Physician's Global Assessment....48
9.1.10 Inflammatory Bowel Disease Questionnaire (IBDQ)....48
9.1.11 UC-related events....49
9.1.12 Laboratory Test....49
9.1.13 Contraception and Pregnancy Avoidance Procedure....50
9.1.14 Pregnancy....50
9.1.15 ECG Procedure....51
9.1.16 PML Evaluation....51
9.1.17 Tuberculosis test....51
9.1.18 Chest Imaging Procedure....52
9.1.19 Pharmacogenomic Sample Collection....52
9.1.20 Pharmacokinetic Sample Collection and Analysis....52
9.1.21 Sample Collection for the HAHA/Neutralizing Antibody Test....52
9.1.22 Record of Subjects Who Withdrew during the Screening Phase....53
9.1.23 Enrollment into the induction Phase....53
9.1.24 Registration of the Results of the Primary Efficacy Endpoint in the Induction Phase/Enrollment into the Maintenance Phase....54
9.1.25 Enrollment into the Open-label Cohort....54
9.2 Study Drug Compliance....55
9.3 Schedule of Observations and Procedures....55
9.3.1 The Screening Phase (Visit 1)....55
9.3.2 Induction Phase....55
9.3.2.1 Baseline Evaluation/Enrollment into the induction Phase (Visit 2)....55
9.3.2.2 Administration in the Induction Phase (Visits 3 and 4)....56

9.3.2.3 Evaluation of Primary Efficacy Endpoint (Visit 5)/Withdrawal during the Induction Phase 57
9.3.3 Maintenance Phase....57
9.3.3.1 Enrollment into the maintenance Phase (Visit 6)....57
9.3.3.2 Administration in the Maintenance Phase (Visits 7 through 17)....58
9.3.3.3 Evaluation of Primary Efficacy Endpoint in the Maintenance Phase (Visit 18)/Withdrawal during the Maintenance Phase 58
9.3.4 Open-label Cohort....59
9.3.4.1 Enrollment into the open-label cohort (Visit 1x) 59
9.3.4.2 Administration in the Open-label Cohort (Visits 2x through 24x) 59
9.3.4.3 The Final Evaluation of the Open-label Cohort (VIST 25x)/Withdrawal during the Open-label Cohort....60
9.3.5 The Unscheduled Visits 61
9.3.6 Predefined Study Completion....61
9.3.7 At 16 weeks after the Last Dose....61
9.3.8 Post Study Care....62
9.4 Retention and Disposal of Biological Samples 62
9.5 Follow-up survey....62
10.0 PRETREATMENT EVENTS AND ADVERSE EVENTS 64
10.1 Definitions....64
10.1.1 PTEs....64
10.1.2 AEs....64
10.1.3 Additional Points to Consider for PTEs and AEs....64
10.1.4 SAEs....66
10.1.5 Intensity of PTEs and AEs 67
10.1.6 Causality of AEs 67
10.1.7 Relationship to Study Procedures....67
10.1.8 Start Dates 68
10.1.9 Stop Date 68
10.1.10 Frequency 68
10.1.11 Action Concerning Study Drug 68
10.1.12 Outcome....69
10.2 Procedures....69
10.2.1 Collection and Reporting of AEs....69
10.2.1.1 PTE and AE Collection Period....69

10.2.1.2 PTE and AE Reporting 70
10.2.2 Collection and Reporting of SAEs..70
10.2.3 Drug-induced Liver Function Test Abnormal Possibly Leading to Sever Liver Disorder..71
10.3 Follow-up of SAEs 71
10.3.1 SAE Reports to Investigators, the IRBs and Regulatory Authorities 71
11.0 STUDY-SPECIFIC COMMITTEES 72
11.1 Independent Adjudication Committee for PML..72
11.2 Clinical Endpoint Committee..72
12.0 DATA HANDLING AND RECORDKEEPING..73
12.1 CRFs (Electronic and Paper)..73
12.2 Record Retention 74
13.0 STATISTICAL METHODS..75
13.1 Statistical and Analytical Plans 75
13.1.1 Analysis Sets..75
13.1.2 Analysis of Demographics and Other Baseline Characteristics 76
13.1.3 Efficacy Analysis..76
13.1.4 Pharmacokinetic Analysis 79
13.1.5 Safety Analysis 79
13.1.6 Analysis of Immunogenicity Endpoints..81
13.2 Interim Analysis and Criteria for Early Termination 81
13.3 Determination of Sample Size..81
14.0 QUALITY CONTROL AND QUALITY ASSURANCE.......................................83
14.1 Study-Site Monitoring Visits 83
14.2 Protocol Deviations..83
14.3 Quality Assurance Audits and Regulatory Agency Inspections 83
15.0 ETHICAL ASPECTS OF THE STUDY 84
15.1 IRB Approval 84
15.2 Subject Information, Informed Consent, and Subject Authorization 84
15.3 Subject Confidentiality 86
15.4 Publication, Disclosure, and Clinical Trial Registration Policy................................86
15.4.1 Publication and Disclosure..86
15.4.2 Clinical Trial Registration..86
15.4.3 Clinical Trial Results Disclosure 87
15.5 Insurance and Compensation for Injury..87

16.0 REFERENCES....88

LIST OF IN-TEXT TABLES

Table 7.a List of Excluded Drugs and Treatments....33
Table 8.a Study drug....39
Table 8.b Materials....40
Table 8.c Dose and Regimen....41
Table 9.a Mayo Scoring System for the Assessment of UC Activity[13]....47
Table 9.b Laboratory tests....49
Table 10.a Takeda Medically Significant AE List....67

LIST OF IN-TEXT FIGURES

Figure 6.a Schematic of Study Design....24
Figure 6.b Schematic of Enrollments, Administrations, and Visits....25
Figure 8.a Labels on Vials....39
Figure 8.b A Label on Outer Packages....40

LIST OF APPENDICES

Appendix A Schedule of Study Procedures....90
Appendix B Responsibilities of the Investigator....94
Appendix C Diagnostic Criteria for UC (Revised on January 17, 2013)....95
Appendix D PML Checklists....100
Appendix E Follow-Up Survey Form....105

2.0 STUDY SUMMARY

**Sponsor:**<br>Takeda Pharmaceutical Company Limited	**Compound:**<br>MLN0002	
**Study title:** Phase 3, multicenter, randomized, double-blinded, placebo-controlled, parallel-group study to evaluate the efficacy, safety, and pharmacokinetics of intravenous MLN0002 (300 mg) infusion in induction and maintenance therapy in Japanese subjects with moderate or severe ulcerative colitis	**IND No.:**<br>Not Applicable	**EudraCT No.:**<br>Not Applicable
**Study Number**: MLN0002/CCT-101	**Phase** :3	
**Study Design:**<br>This is a phase 3, multicenter, randomized, double-blinded, placebo-controlled, parallel-group study to evaluate the efficacy, safety, and pharmacokinetics of MLN0002 in induction and maintenance therapies in Japanese subjects with moderate or severe ulcerative colitis (UC). This study consists of the screening, induction and maintenance phases, and the open-label cohort.<br>The screening phase involves screening tests of consenting subjects who visit a study site between 21 and 3 days before the start of treatment. Subjects meeting the eligibility criteria based on the inclusion and exclusion criteria on the first day of treatment (Day 1) will be enrolled into the induction phase.<br>The induction phase consists of two cohorts and enrollment into Cohort 2 will start only after completion of enrollment into Cohort 1. Subjects enrolled into Cohort 1 will be randomized 1:2 to the placebo or MLN0002 group to receive the placebo or 300 mg of MLN0002 at Weeks 0, 2, and 6 in a double-blinded manner. Subjects enrolled into Cohort 2 will receive 300 mg of MLN0002 at Weeks 0, 2, and 6 in an unblinded manner. The primary efficacy endpoint in the induction phase will be evaluated at Week 10.<br>Subjects showing a clinical response to the study drug at Week 10 will be enrolled into the maintenance phase at Week 14. Subjects who received the placebo in the induction phase will continue to receive the placebo at Weeks 14, 22, 30, 38, 46, and 54 in a double-blinded manner. Subjects who received MLN0002 in the induction phase will be randomized 1:1 to the placebo or MLN0002 group, and will receive the placebo or 300 mg of MLN0002 at Weeks 14, 22, 30, 38, 46, and 54 in a double-blinded manner. The primary efficacy endpoint in the maintenance phase will be evaluated at Week 60.<br>Subjects not showing a clinical response to the study drug at Week 10 can be enrolled into the open-label cohort. Setting the enrollment day to the open-label cohort as Week 0x, those subjects will receive 300 mg of MLN0002 at Weeks 0x, 2x, 6x and then every 8 weeks thereafter for 46 weeks (minimum) to 94 weeks (maximum) in an unblinded manner. Subjects who experienced disease worsening or received rescue treatments during the maintenance phase, or those who completed Week 60 of the maintenance phase can be enrolled into the open-label cohort to receive the study drug in the same manner. Administration of MLN0002 will be terminated in all subjects at Week 46x of the last subject enrolled into the open-label cohort.<br>The end-of-study examination will be performed at 16 weeks after the last dose in all subjects who received the study drug. In addition, the follow-up survey will be performed every 6 months from the last dose of the study drug up to 2 years or until the date of marketing approval of the study drug, whichever comes earlier.		
**Primary objective for the induction phase:**<br>To evaluate efficacy of MLN0002 in induction therapy in Japanese subjects with moderate or severe UC.		
**Secondary objective for the induction phase:**<br>To evaluate safety of MLN0002 in induction therapy in Japanese subjects with moderate or severe UC.		
**Primary objective for the maintenance phase:**<br>To evaluate efficacy of MLN0002 in maintenance therapy in Japanese subjects with moderate or severe UC.		

<table>
<tr><td colspan="2">Secondary objective for the maintenance phase:
To evaluate safety of MLN0002 in maintenance therapy in Japanese subjects with moderate or severe UC.</td></tr>
<tr><td colspan="2">Subjects: Japanese subjects with moderate or severe UC</td></tr>
<tr><td>Number of Subjects:
Approximately 278 subjects in total.
An initial cohort of 246 subjects (Cohort 1) will be randomized in the induction phase to receive MLN0002 (n=164) or the placebo (n=82). The number of subjects in Cohort 2 in the induction phase can be adjusted to provide the sample size (n=140) to be enrolled into the maintenance phase. The target number of subjects without a history of anti-TNFα antibody therapy is approximately 50% of the total.</td><td>Number of Sites:
Approximately 100 sites</td></tr>
<tr><td>Dose Level(s):
MLN0002 (300 mg) or the placebo will be administered by intravenous infusion at Weeks 0, 2, 6 and every 8 weeks thereafter.</td><td>Route of Administration:
Intravenous infusion (IV)</td></tr>
<tr><td>Duration of Treatment:
54 weeks (maximum 154 weeks)</td><td>Period of Evaluation:
70 weeks (maximum 170 weeks)
The follow-up survey will be performed every 6 months from the last dose of the study drug for up to 2 years or until the date of marketing approval of the study drug, whichever comes earlier.</td></tr>
<tr><td colspan="2">Main Criteria for Inclusion:
• A subject aged 15 to 80 (inclusive) at the time of signing the informed consent.
• Subjects with diagnosis of total or left-sided UC based on the Revised Diagnostic Criteria for UC issued by “Research Group for Intractable Inflammatory Bowel Disease” Designated as Specified Disease by the Ministry of Health, Labor and Welfare (MHLW) of Japan (2012) at least 6 months before the start of administration of the study drug.
• A subject with moderately or severely active UC as determined by baseline complete Mayo score of 6 to 12 (inclusive) with an endoscopic subscore of ≥2.
• Subjects whose complication of colon cancer or dysplasia had to be ruled out by total colonoscopy at the start of the study drug administration (or the results from total colonoscopy performed within 1 year before giving consent are available), if subjects met any of the following criteria; subjects with ≥8-year history of total or left-sided colitis, subjects aged ≥50 years, or subjects with a first-degree family history of colon cancer.
• Subjects meeting the following treatment failure criteria with at least one of the following agents within 5 year before the time of signing on the informed consent form:
Corticosteroids (steroids)
- Resistance
- Dependence
- Intolerance
Immunomodulators [azathioprine (AZA) or 6-mercaptopurine (6-MP)]
- Refractory
- Intolerance
Anti-TNFα antibodies
- Inadequate response
- Loss of response
- Intolerance</td></tr>
</table>

Main Criteria for Exclusion:

- Subjects whose partial Mayo score decreased by 3 points or more between screening and the start of the study drug administration.
- Subjects having or suspected to have abdominal abscess or toxic megacolon.
- Subjects with a history of subtotal or total colectomy.
- Subjects with ileostomy, colostomy, fistula or severe intestinal stenosis.
- Subjects who started oral 5-aminosalicylic acids (5-ASAs), probiotics, or oral corticosteroids (≤30 mg/day) within 13 days before the first dose of the study drug. Subjects who have used these drugs for at least 14 days before the first dose of the study drug, and who changed dosage of or discontinued these drugs within 13 days before the first dose of the study drug.
- Subjects who have used 5-ASAs, corticosteroid enemas/suppositories, corticosteroid IV infusion, oral corticosteroid at >30 mg/day, drugs for diarrhea-predominant irritable bowel syndrome, or Chinese herbal medicine for the treatment of UC (e.g., Daikenchuto) within 13 days before the first dose of the study drug.
- Subjects who have used an antidiarrheal drug for 4 or more consecutive days within 13 days before the first dose of the study drug or within 7 days before the first dose of the study drug.
- Subjects who have used AZA or 6-MP within 27 days before the first dose of the study drug However, this will not apply to subjects who have used these drugs for 83 or more days before the first dose of the study drug and continued the steady dose administration of the drugs for 27 or more days before the first dose of the study drug.
- Subjects who have received cyclosporine, tacrolimus, methotrexate, tofacitinib or any study drugs of low-molecular compound for UC treatment within 27 days before the first dose of the study drug.
- Subjects who have received adalimumab within 27 days before the first dose of the study drug or any biologic agents other than adalimumab within 55 days before the first dose of the study drug. However, this will not apply to subjects who have topically received these drugs (e.g., intraocular injection for treatment of age-related macular degeneration).
- Subjects who have received any live-vaccinations within 27 days before the first dose of the study drug.
- Subjects who have undergone an enterectomy within 27 days before the first dose of the study drug or those anticipated to require the enterectomy during the study.
- Subjects who have received leukocytapheresis or granulocyte apheresis within 27 days before the first dose of the study drug.
- Subjects with an evidence of adenomatous colonic polyps that need to be removed at the start of the study drug administration.
- Subjects with a history or a complication of colonic mucosal dysplasia.

Main efficacy endpoints of the induction phase:

Primary endpoint:

Clinical response at Week 10

Secondary endpoints:

Clinical remission at Week 10 and mucosal healing at Week 10

Main efficacy endpoints of the maintenance phase:

Primary endpoint:

Clinical remission at Week 60

Secondary endpoints:

Durable response, mucosal healing at Week 60, durable remission, and corticosteroid-free remission at Week 60

Pharmacokinetic endpoints:

Serum MLN0002 concentration

Safety endpoints:

Adverse events (AEs), body weight, vital signs, electrocardiogram (ECG), clinical laboratory test (blood biochemistry, hematology, and urinalysis)

Immunogenicity endpoints:

HAHA, neutralizing antibody

Statistical analysis plan:

(1) Statistical analysis in the induction phase

The following descriptive calculations will be performed in the “Full Analysis Set in the induction phase (subjects randomized and received the study drug at least once in the induction phase).”

For the primary efficacy analysis, “clinical response at Week 10” (primary endpoint of the induction phase) will be calculated by treatment group of the induction phase. Also clinical response at Week 10 will be calculated with stratification according to a history of anti-TNFα antibody therapy, and the comparison between treatment groups will be made with the Cochran-Mantel-Haenszel (CMH) test using a history of anti-TNFα antibody therapy as a stratification factor. The results of the primary analysis of the induction phase will be interpreted as below to determine the efficacy of MLN0002 induction therapy.

- The superiority of MLN0002 over the placebo on clinical response at Week 10 in the primary analysis of the induction phase is demonstrated when a statistically significant difference is observed in the clinical response at Week 10.

For the secondary efficacy analysis, clinical remission at Week 10 will be calculated in the same manner as those in the primary analysis in the induction phase. The results of the secondary analysis will be interpreted as below to determine the efficacy of MLN0002 induction therapy.

- The superiority of MLN0002 over the placebo on clinical remission at Week 10 in the primary analysis of the induction phase is demonstrated when a statistically significant difference is observed in both the clinical response at Week 10 and clinical remission at Week 10.

(2) Statistical analysis in the maintenance phase

The following descriptive calculations will be performed in the “Full Analysis Set of the maintenance phase (subjects randomized and received the study drug at least once in the maintenance phase).”

For the primary efficacy analysis, “clinical remission at Week 60” (primary endpoint of the maintenance phase)” will be calculated by treatment group of the maintenance phase. Also clinical remission at Week 60 will be calculated with stratification according to a history of anti-TNFα antibody therapy, and the comparison will be made with the Cochran-Mantel-Haenszel (CMH) test using a history of anti-TNFα antibody therapy as a stratification factor. The results of the primary analysis of the maintenance phase will be interpreted as below to determine the efficacy of MLN0002 maintenance therapy.

- The superiority of MLN0002 over the placebo on clinical remission at Week 60 in the primary analysis of the maintenance phase is demonstrated when a statistically significant difference is observed in the clinical remission at Week 60.

(3) Significance level

Since the study has different objectives in the induction and maintenance phases, no adjustments for multiplicity will be made between the analyses of the two phases, setting the significance level at 5% each.

Sample size justification:

On the basis of the findings from the phase 3 double-blinded, placebo-controlled study of MLN0002 in induction and maintenance therapy in non-Japanese subjects with moderate to severe UC (C13006), the clinical response at Week 10 and clinical remission at Week 60 for MLN0002 and the placebo in this study was estimated to be 57.1% and 35.5%, and 43.3% and 15.9%, respectively.

When the clinical response at Week 10 in the induction phase is analyzed using Pearson's chi-square test with 2-sided significance level of 5%, 164 and 82 subjects (at a ratio of 2:1, total 246 subjects) will be required respectively for the MLN0002 and placebo group to detect significance with a power of at least 90%. These numbers were therefore

selected as numbers of evaluable subjects of the primary endpoint of the induction phase (number of "subjects randomized in the induction phase and who received at least one dose of the study drug in the induction phase"). When the clinical remission at Week 10 in the induction phase is analyzed on these numbers of subjects, statistical significances in both the clinical response at Week 10 and clinical remission at Week 10 are detected with a power of 69%.<br>When the clinical remission at Week 60 in the maintenance phase is analyzed using Pearson's chi-square test with 2-sided significance level of 5%, 42 subjects each for the MLN0002 and placebo group (total 84 subjects) will be required to detect significance with a power of at least 80%. Similarly, 55 subjects each (total 110subjects) will be required to detect significance with a power of at least 90%. These numbers were therefore selected as numbers of evaluable subjects of the primary endpoint of the maintenance phase (number of "subjects randomized in the maintenance phase and who received at least one dose of the study drugs in the maintenance phase"). Subjects enrolled into the maintenance phase include "those assigned to the placebo group in the induction phase" to maintain the blindness in the induction phase. To detect significance with a power of 80 and 90% for the analysis of the primary endpoint of the maintenance phase, therefore, 110 and 140 subjects will be respectively necessary for subjects randomized in the maintenance phase.

3.0 LIST OF ABBREVIATIONS

5-ASA	5-aminosalicylic acid
6-MP	6-mercaptopurine
ALT	alanine aminotransferase
ALP	alkaline phosphatase
AST	aspartate aminotransferase
AZA	Azathioprine
BMI	body mass index
BUN	blood urea nitrogen
CD	Crohn’s disease
CDAI	Crohn’s Disease Activity Index
CRP	C-reactive protein
ELISA	Enzyme-Linked ImmunoSorbent Assay
FDA	Food and Drug Administration
GCP	Good Clinical Practice
γ-GTP	γ-glutamyl transpeptidase
HAHA	human anti-human antibody
HBs	hepatitis B surface antigen
hCG	human chorionic gonadotropine
HCV	hepatitis C virus
HIV	human immunodeficiency virus
IBDQ	Inflammatory Bowel Disease Questionnaire
ICH	International Conference on Harmonisation
INR	International normalized ratio
MedDRA	Medical Dictionary for Regulatory Activities
MHRA	Medicines and Healthcare products Regulatory Agency
PMDA	Pharmaceutical and Medical Devices Agency
PML	progressive multifocal leukoencephalopathy
SUSARs	suspected serious unexpected adverse reactions
TNF	Tumor Necrosis Factor
UC	Ulcerative colitis
WHO	World Health Organization

3.1 Definitions in the Study

The definitions in the study are provided below;

Terms	Definitions
Clinical remission	≤2 in the complete Mayo score and ≤1 in all subscores
Clinical response	The following 2 conditions should be fulfilled;<br>• Decrease of the complete Mayo score by ≥3 points and by ≥30% from baseline (decrease of the partial Mayo score by ≥2 points and by ≥25% from baseline at the visits not for evaluation of the complete Mayo score)<br>• Decrease of the subscore of rectal bleeding by ≥1 point from baseline, or ≥1 in the subscore of rectal bleeding
Mucosal healing	≤1 in the subscore for findings on endoscopy in the complete Mayo score
Durable response	Clinical response was observed both at Week 10 and Week 60
Durable remission	Clinical remission was observed both at Week 10 and Week 60
Corticosteroid-free remission at Week 60	Clinical remission without corticosteroid at Week 60, though a corticosteroid was used at the first dose of the study drug
Disease worsening	In the maintenance phase, increase of the partial Mayo score by ≥3 points from that of Week 10 in 2 successive visits* , and ≥5 in the partial Mayo score (or 9 in the 2 successive visits* in the case of >6 at Week 10)<br>or in the open-label cohort, increase of the partial Mayo score by ≥3 points from that of Week 10x in 2 successive visits* , and ≥5 in the partial Mayo score (or 9 in the 2 successive visits* in the case of >6 at Week 10x)
Sustained non-response	No-clinical response and ≥5 in the partial Mayo score during 20 weeks from the first dose of the study drug
Rescue treatments	Any new drug for UC treatment, increase of dosage of a drug used from the first dose of the study drug (except antidiarrheal drug), or a combination therapy (except abscess drainage or the seton method for the anal lesion)<br>Any increase of dosage or a new dose of drugs permitted in the open-label cohort, when initiated after enrollment into the open-label cohort, should not be considered as the rescue treatments.
Treatment failure	Disease worsening, rescue medications, or study discontinuation due to study drug-related AEs

* The 2 visits includes the unscheduled visit due to UC worsening, etc. The interval between visits will not to be less than 1 week, where possible.

4.0 INTRODUCTION

4.1 Background

Ulcerative Colitis (UC) is an inflammatory bowel disease repeatedly recur and remit. The lesion, involving the rectum toward the mouth sequentially, extends from the rectum through the colon at maximum. Specific symptoms are chronic viscous bloody stool or blood stool, and other symptoms including frequent diarrhea, abdominal pain, fever, anorexia or anemia. Frequent diarrhea limits going out, so that the Quality of Life (QOL) may deteriorate.

Though etiology remains unknown yet, recent studies have revealed that breakdown of immune tolerance to intraluminal antigens including enterobacteria and abnormal cytokines may be a contributing factor. A curative treatment has not been established, therefore the medical approach intends basically to induce remission promptly to be maintained for a longer period (remission maintenance) with pharmacotherapy. The guidance of pharmacotherapy is provided in the Revised Guideline or Guidance[1] issued by Research Group for Intractable Inflammatory Bowel Disease Designated as Specified Disease by the MHLW of Japan, however treatment should be detailed based on disease types or severities.

MLN0002 is a recombinant human immunoglobulin G1 monoclonal antibody[2] that specifically binds to human lymphocyte $\alpha_4\beta_7$ integrin, and is currently being developed in UC and Crohn's disease (CD) inside/outside Japan.

MLN0002 binds and inhibits $\alpha_4\beta_7$ integrin, a cell membrane protein of lymphocytes, to bind its principal ligand of mucosal addressin cell adhesion molecule-1 (MAdCAM-1; a transmembrane glycoprotein markedly expressing on vascular endothelial cells in the high endothelial venule in the Peyer patch or the mesenteric lymph node and the arteriole in the intestinal lamina propria), leading to suppression of lymphocytes to migrate toward the intestinal mucosa and the gut-related lymphoid tissue[2]. From the above, MLN0002 is anticipated to exert efficacy with superior safety to conventional treatments or other biological drugs in UC and CD through suppression of intestinal inflammation by gastrointestinal tract-selective immunomodulatory effect not requiring systemic immunosuppression[3][4].

4.2 Outcome of Clinical Studies

Outside Japan, the following 4 Phase 3 studies (GEMINI Programs) were conducted in subjects with UC and CD in addition to the 14 Phase 1 and 2 studies. As of October, 2013, all studies were completed except a long-duration study in subjects with UC and CD (C13008), and applications have been submitted in Europe and US. In Japan, the Phase 1 study was conducted in subjects with UC.

Study Number	Study Title
C13006	Phase 3, double-blinded, placebo-controlled study of induction and maintenance therapies in moderate to severe UC patients (GEMINI 1).
C13007	Phase 3, double-blinded, placebo-controlled study of induction and maintenance therapies in moderate to severe CD patients (GEMINI 2).
C13011	Phase 3, double-blinded, placebo-controlled study of induction therapy in moderate to severe CD patients who had failed with anti-TNFα antibodies therapy (GEMINI 3).
C13008	Phase 3, long-term open-label safety and efficacy study in UC or CD patients who had been enrolled in the Phase 2 and Phase 3 study of MLN0002.

4.2.1 Summary of Outcome of Phase 1 and 2 Studies Outside Japan

Major outcomes in Phase 1 and 2 studies conducted outside Japan were summarized below;

- The pharmacokinetics of MLN0002 at the doses of 2 to 10 mg/kg was linear. The elimination half-life of MLN0002 was 15 to 22 days[5].
- The $\alpha_4\beta_7$ receptors were saturated after the first dose at the doses of 2 to 10 mg/kg. Saturation of $\alpha_4\beta_7$ receptors had been dose-dependently continued for several months after the last dose[5].
- It was suggested that the human anti-human antibody to MLN0002 (anti-MLN0002 antibody; hereinafter referred to as HAHA) may eliminate MLN0002 from the blood, resulting in decrease of MLN0002 exposure and loss of saturation of $\alpha_4\beta_7$ receptors. HAHA emergence was dose-dependently decreased.
- MLN0002 demonstrated superiority to the placebo in all subjects as clinical remission was 32% at Week 6 in UC and 37% at Week 8 in CD[6][7]. The frequency of AEs was not significantly different from that in the placebo with no particular safety issues.

4.2.2 Summary of Outcome of Phase 3 Studies Outside Japan

In all 3 Phase 3 studies conducted outside Japan (C13006, C13007 and C13011), 300 mg of MLN0002 or the placebo were administered. In C13006 study, superiorities were demonstrated to the placebo in the primary endpoints, i.e., the clinical response in the induction phase and the clinical remission in the maintenance phase[8]. Two primary endpoints were set in the induction phase of C13007 study. The difference in the clinical response in CD Activity Index (CDAI)-100 was not significant, however superiority to the placebo was demonstrated in the clinical remissions each in the induction phase and the maintenance phase[9]. In C13011 study, superiority to the placebo was not demonstrated in the primary endpoint of the clinical remission at Week 6 in the sub-population of subjects who had failed with anti-TNFα antibodies (75% of all population), however the clinical remission at Week 6 in the whole population was significantly different from the placebo. In addition, the significant difference from the placebo in the clinical remission at Week 10 was observed both in the sub-population who had failed an anti-TNFα antibody and the whole population[10].

Furthermore, incidence of AEs and serious adverse events (SAEs) were not significantly different between the whole MLN0002 group and placebo group. Progressive multifocal leukoencephalopathy (PML) was not observed in any subjects in completed or ongoing clinical development programs.

4.2.3 Summary of Outcome of Phase 1 Study in Japan

In Japan, the Phase 1 repeated-dose study in UC patients (CPH-001) was conducted to investigate safety, pharmacokinetics and pharmacodynamics of MLN0002 dosed at 150 or 300 mg in Weeks 0, 2 and 6. MLN0002 was well tolerated by the dose of 300 mg, and no injection site reaction or anaphylactic reaction was observed. In addition, serum MLN0002 concentration increased depending on dose ratio, and saturation of $\alpha_4\beta_7$ receptors of the pharmacodynamic index was observed mostly continuously from the first dose[11].

4.3 Rationale for the Proposed Study

- In the 3 Phase 3 studies conducted outside Japan (C13006, C13007 and C13011), efficacy and safety of 300 mg of MLN0002 were investigated against the placebo each in the induction phase and the maintenance phase in moderate to severe UC or CD patients who had failed with the existing pharmacotherapy including anti-TNFα antibodies. As a result, significant difference from the placebo was observed in efficacy evaluation of MLN0002 in all studies. In addition, no particular issues were found in the safety evaluation. These results were consistent with the outcome obtained until Phase 2 studies.
- In Japan, safety, serum MLN0002 concentration and saturability of $\alpha_4\beta_7$ receptors were investigated in UC patients dosed with 150 or 300 mg of MLN0002 at Weeks 0, 2 and 6. The serum MLN0002 concentration resulted in similarity inside/outside Japan, and saturation of $\alpha_4\beta_7$ receptors was observed mostly continuously from the first dose. In addition, no safety issues were found.

From the above, the study was proposed as a Phase 3 study to investigate efficacy and safety of 300 mg of MLN0002 in moderate to severe Japanese UC patients.

Pharmacogenomic analysis may be conducted to evaluate contribution of genetic variance on drug response, for example, its efficacy and safety. Participation of study subjects in pharmacogenomic sample collection is optional.

As pharmacogenomics is an evolving science, currently many genes and their function are not yet fully understood. Future data may suggest a role of some of these genes in drug response or diseases, which may lead to additional hypothesis-generating exploratory research on banked samples.

5.0 STUDY OBJECTIVES AND ENDPOINTS

5.1 Objectives

5.1.1 Primary Objective for the Induction Phase

To evaluate efficacy of MLN0002 in induction therapy in Japanese subjects with moderate or severe UC.

5.1.2 Secondary Objective for the Induction Phase

To evaluate safety of MLN0002 in induction therapy in Japanese subjects with moderate or severe UC.

5.1.3 Primary Objective for the Maintenance Phase

To evaluate efficacy of MLN0002 in maintenance therapy in Japanese subjects with moderate or severe UC.

5.1.4 Secondary Objective for the Maintenance Phase

To evaluate safety of MLN0002 in maintenance therapy in Japanese subjects with moderate or severe UC.

5.2 Endpoints

5.2.1 Efficacy Endpoints for the Induction Phase

5.2.1.1 Primary Endpoint

- Clinical response at Week 10

5.2.1.2 Secondary Endpoints

- Clinical remission at Week 10
- Mucosal healing at Week 10

5.2.1.3 Exploratory Endpoints

- Clinical response, clinical remission and mucosal healing at Week 10 in the sub-population of subjects without a history of anti-TNFα antibody therapy and in the sub-population of subjects who had failed with an anti-TNFα antibody
- Clinical response, clinical remission and mucosal healing at Week 10 in the sub-population of subjects who had failed with a corticosteroid monotherapy and an immunomodulator (except those failed with an anti-TNFα antibody)
- IBDQ score change at Week 10 from baseline

- Partial Mayo score change over time

5.2.2 Efficacy Endpoints for the Maintenance Phase

5.2.2.1 Primary Endpoint

- Clinical remission at Week 60

5.2.2.2 Secondary Endpoints

- Durable response
- Mucosal healing at Week 60
- Durable remission
- Corticosteroid-free remission at Week 60

5.2.2.3 Exploratory Endpoints

- Oral corticosteroid dosage change from Week 10 and rate of the change over time
- Time to disease worsening
- Time to treatment failure
- Clinical remission and mucosal healing at Week 60 in the sub-population of subjects without a history of anti-TNFα antibody therapy and in the sub-population of subjects who had failed with an anti-TNFα antibody therapy
- Clinical remission and mucosal healing at Week 60 in the sub-population of subjects who had failed with a corticosteroid monotherapy and an immunomodulator (except those failed with an anti-TNFα antibody)
- IBDQ score change at Week 38 and Week 60 from Week 14
- Partial Mayo score change over time
- Time to major UC-related events (UC-related hospitalization or enterectomy)

5.2.3 Pharmacokinetic Endpoint

- Serum MLN0002 concentration

5.2.4 Safety Endpoints

- Adverse events (AEs)
- Body weight
- Vital signs

- ECG
- Clinical laboratory test (blood biochemistry, hematology and urinalysis)

5.2.5 Immunogenicity Endpoint

- HAHA, neutralizing antibody

6.0 STUDY DESIGN AND DESCRIPTION

6.1 Study Design

This is a phase 3, multicenter, randomized, double-blinded, placebo-controlled, parallel-group study to evaluate the efficacy, safety, and pharmacokinetics of MLN0002 in induction and maintenance therapies in Japanese subjects with moderate or severe UC. This study consists of the screening, induction, maintenance phases and open-label cohort.

The screening phase involves screening tests of consenting subjects who visit a study site between 21 and 3 days before the start of treatment. Subjects meeting the eligibility criteria based on the inclusion and exclusion criteria on the first day of treatment (Day 1) will be enrolled into the induction phase.

The induction phase consists of two cohorts and enrollment into Cohort 2 will start only after completion of enrollment into Cohort 1. Subjects enrolled into Cohort 1 will be randomized 1:2 to the placebo or MLN0002 group[1], and will receive the placebo or 300 mg of MLN0002 at Weeks 0, 2, and 6 in a double-blinded manner. Subjects enrolled into Cohort 2 will receive 300 mg of MLN0002 at Weeks 0, 2, and 6 in an unblinded manner. The primary efficacy evaluation in the induction phase will be performed at Week 10.

Subjects showing a clinical response to the study drug at Week 10 will be enrolled into the maintenance phase at Week 14. Subjects who received the placebo in the induction phase will continue to receive the placebo at Weeks 14, 22, 30, 38, 46, and 54 in a double-blinded manner. Subjects who received MLN0002 in the induction phase will be randomized 1:1 to the placebo or MLN0002 group[2] to receive the placebo or 300 mg of MLN0002 at Weeks 14, 22, 30, 38, 46 and 54 in a double-blinded manner. The primary efficacy endpoint in the maintenance phase will be evaluated at Week 60.

Subjects not showing a clinical response to the study drug at Week 10 can be enrolled into the open-label cohort. Setting the enrollment day to the open-label cohort as Week 0x, those subjects will receive 300 mg of MLN0002 at Weeks 0x, 2x, 6x and then every 8 weeks thereafter for 46 weeks (minimum) to 94 weeks (maximum) in an unblinded manner. Subjects who experienced disease worsening or received rescue treatments during the maintenance phase, or those who completed Week 60 of the maintenance phase can be enrolled into the open-label cohort to receive the study drug in the same manner. Administration of MLN0002 will be terminated in all subjects at Week 46x of the last subject enrolled into the open-label cohort.

The end-of-study examination will be performed at 16 weeks after the last dose in all subjects who received the study drug. In addition, the follow-up survey will be performed every 6 months from

[1]The dynamic allocation with the treatment history with anti-TNFα antibodies, concomitant use of an immunomodulator or corticosteroid, and the study sites.
[2]The dynamic allocation with the treatment history with anti-TNFα antibodies, concomitant use of an immunomodulator or a corticosteroid, the study sites, the cohort in the induction phase and responder/non-responder at Week 10.

the last dose of the study drug up to 2 years or until the date of marketing approval of the study drug, whichever comes earlier (see Section 9.5).

Sample size is planned to be approximately 278 subjects in total, and 246 subjects (Cohort 1) will be randomized in the induction phase to receive MLN0002 (n=164) or the placebo (n=82). The number of subjects in Cohort 2 in the induction phase can be adjusted so that the planned sample size (n=110 to 140) may be enrolled into the maintenance phase. The target number of subjects without a history of an anti-TNFα antibody therapy is approximately 50% of the total.

A schematic of the study design and a schematic of enrollments, administrations and visits are shown in Figure 6.a and Figure 6.b, respectively. A schedule of assessments is listed in Appendix A.

Figure 6.a Schematic of Study Design

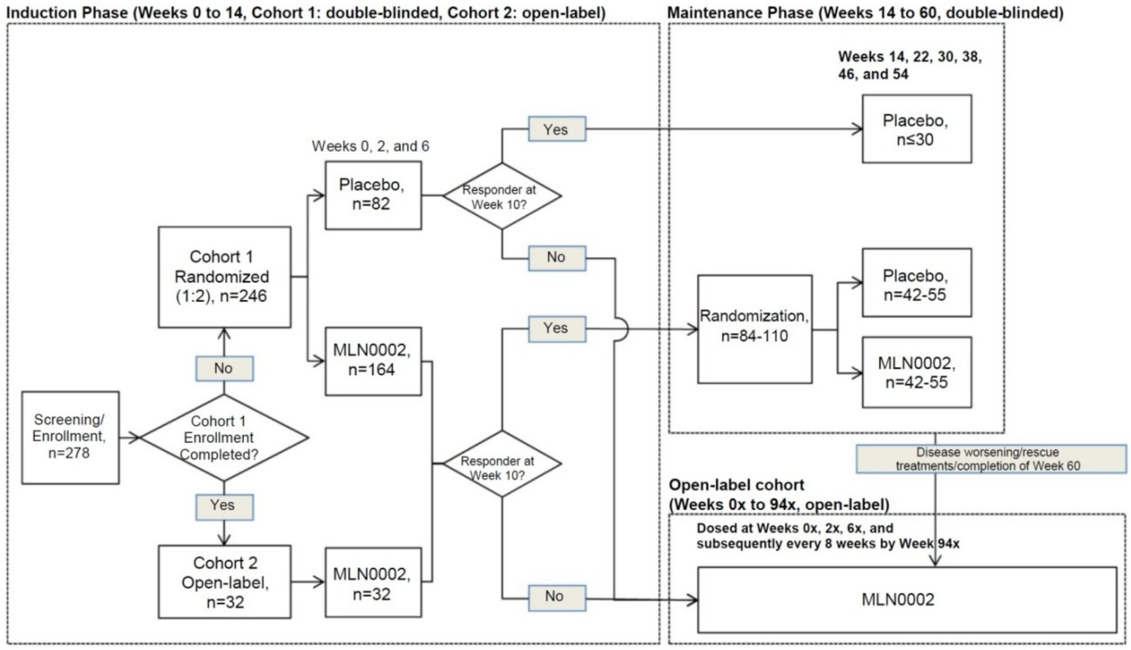

Figure 6.b Schematic of Enrollments, Administrations, and Visits

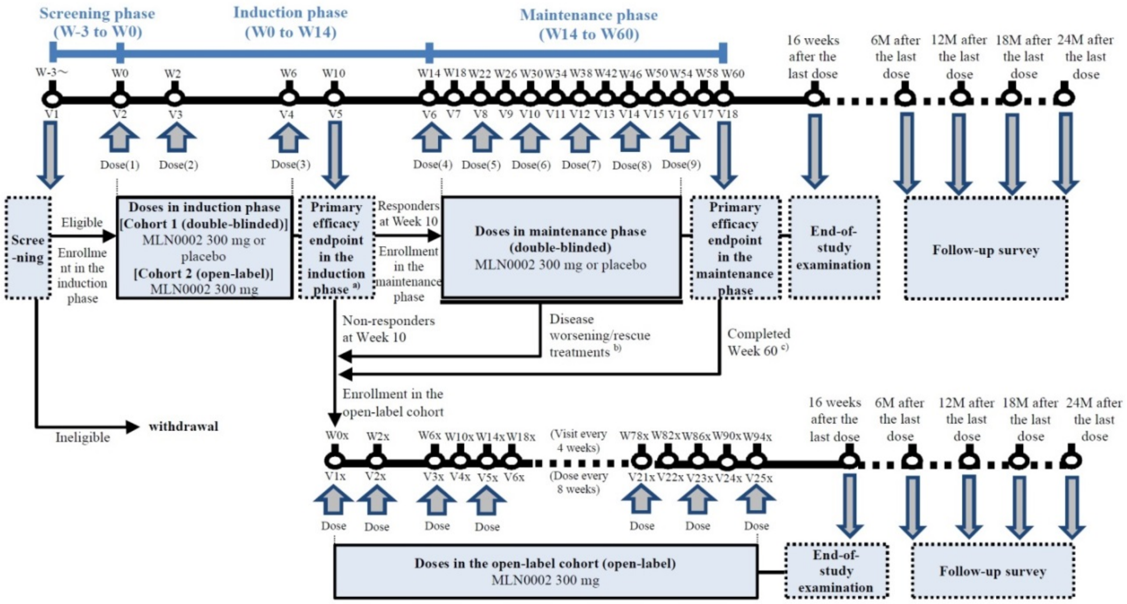

W: Week, V: Visit

a) Responders at Week 10 (evaluation of the primary efficacy endpoints in the induction phase) will be enrolled into the maintenance phase at Week 14. Non-responders at Week 10 may be enrolled into the open-label cohort after completion of the Week 10 evaluation.

b) Subjects who experienced disease worsening or received rescue treatments during the maintenance phase may be enrolled into the open-label cohort after completion of the evaluation at withdrawal of the maintenance phase. However, these subjects will be enrolled into the open-label cohort 3 to 9 weeks after the last dose in the maintenance phase.

c) Subjects who completed Week 60 (evaluation of the primary efficacy endpoint in the maintenance phase) may be enrolled into the open-label cohort after completion of the Week 60 evaluation.

6.2 Justification for Study Design, Dose, and Endpoints

6.2.1 Justification for Study Population

The Guideline for Treatment of UC[1] issued by “Research Group for Intractable Inflammatory Bowel Disease Designated as Specified Disease” by the MHLW of Japan (2012) recommends oral salazosulfapyridines or 5-ASAs in principle, or suppositories/enemas of corticosteroids, suppositories of salazosulfapyridines and enemas of 5-ASAs for remission induction therapy in mild to moderate UC patients. In addition, oral corticosteroids are recommended for patients who inadequately respond to the above treatments or severe patients.

The intractable patients is defined as those whose response is inadequate despite proper usage of corticosteroids (corticosteroid resistance), or those who experienced recurrence/worsening with dose reduction of corticosteroids while symptoms had been stable during use of corticosteroids (corticosteroid dependence). Whereas immunomodulators or anti-TNFα antibodies are recommended for these intractable patients, non-responders to these drugs or loss of response

during the course of treatment are still problematic. MLN0002, having a different mode of action from existing therapeutic drugs, has been suggested to be efficacious in such patients by outcome from studies outside Japan.

From the above, the study population in the induction phase in the study was set as moderate to severe Japanese UC patients who had failed with existing pharmacotherapy (either of corticosteroids/immunomodulators/anti-TNFα antibodies)

6.2.2 Justification of Study Design and Sample Size

The Guideline for Treatment of UC[1] recommends that the remission maintenance therapy should apply to prevent recurrence after achievement of remission induction. However, effective drugs in the induction therapy are not always efficacious in the maintenance therapy. The randomized, double-blinded, placebo-controlled study with an induction phase and maintenance phase was designed to objectively evaluate efficacy and safety both in the induction therapy and maintenance therapy. Additionally, the enrichment design was employed to evaluate efficacy and safety in the maintenance phase, in which only responders to MLN0002 in the induction phase will be enrolled in the population to be randomized in the maintenance phase.

In the induction phase, in addition to the Cohort 1 for randomized, double-blinded, placebo-controlled, parallel-group comparison, the Cohort 2 to receive unblinded dose of MLN0002 was set so that the sample size in the maintenance phase was secured.

For preservation of blindness, all responders at Week 10 will be enrolled into the maintenance phase irrespective of the drugs allocated in the induction phase. The responders who received MLN0002 in the induction phase will be randomized either to the placebo group or the MLN0002 group for evaluation of efficacy and safety of the maintenance therapy, and the responders who received the placebo in the induction phase will be continued to be allocated to the placebo groups.

In the ethically-established open-label cohort, it was designed that non-responders in the induction phase, patients who experienced disease worsening or received rescue treatments during the maintenance phase, or those completed Week 60 of the maintenance phase may continue to receive MLN0002 for 46 weeks (minimum) to 94 weeks (maximum).

See Section 13.3 for justification of the sample size.

6.2.3 Justification of the Placebo Group

Any active comparator is impossible to be used properly in the study in moderate to severe UC patients who had failed with existing pharmacotherapy (either of corticosteroids/immunomodulators/anti-TNFα antibodies). The placebo was selected as a comparator for objective evaluation of efficacy and safety.

6.2.4 Justification of the Dosage and the Evaluation Period

In the Phase 3 study outside Japan in UC patients (C13006) dosed 300 mg of MLN0002 at Weeks 0, 2, 6 and subsequently every 8 weeks or 4 weeks, similar efficacy was observed in either

dosage with no safety issues[8]. The serum MLN0002 concentration was similar inside/outside Japan, and saturation of $\alpha_4\beta_7$ receptors was observed mostly continuously from the first dose. From the above, the dosage in the study was selected as 300 mg of MLN0002 to be dosed at Weeks 0, 2, 6 and subsequently every 8 weeks in a similar manner as that in C13006 study.

Furthermore, efficacy was anticipated to be improved in association with extension of the period of the induction therapy based on the result from C13006. Therefore, the primary efficacy endpoint in the induction phase will be evaluated at Week 10. The maintenance phase is decided to be started from Week 14, and the primary efficacy endpoint in the maintenance phase will be evaluated at 46 weeks after enrollment into the maintenance phase (Week 60) in the similar manner as that in C13006 study.

Meanwhile, non-responders at Week 10 may be enrolled into the open-label cohort for re-induction of the drug and the maintenance therapy, and receive 300 mg of MLN0002 at Weeks 0x, 2x, 6x and subsequently every 8 weeks for 46 weeks (minimum) to 94 weeks (maximum). Subjects who experienced disease worsening or received rescue treatments during the maintenance phase, or those who completed Week 60 of the maintenance phase can be enrolled into the open-label cohort to receive the study drug in the same manner. As safety was demonstrated in the C13006 study when MLN0002 was dosed at Weeks 0, 2 and subsequently every 4 weeks, enrollment into the open-label cohort was judged to impose no safety issues.

The elimination half-life of MLN0002 was 15 to 22 days at the dose of 2 to 10 mg/kg[5]. Additionally, saturation of $\alpha_4\beta_7$ receptors was durable at the dose of 300 mg for 16 weeks after the last dose, and decreased subsequently over time. The end-of-study examination for safety evaluation after the last dose will be conducted in a similar manner as that in studies outside Japan in all subjects who received the study drugs at 16 weeks after the last dose that is almost ≥5-times longer period than the elimination half-life and by which saturation of $\alpha_4\beta_7$ receptors may be durable.

PML has been reported in subjects who received natalizumab, an α_4 integrin antagonist[12]. MLN0002 is considered unlikely to increase PML risk since it specifically binds to the intestine tropism $\alpha_4\beta_7$ integrin, and no PML was observed in clinical trials inside/outside Japan so far. However, the number of subjects who received MLN0002 was not large because of the clinical trial stage, and the potential PML risk is difficult to be ruled out. Therefore, the follow-up survey will be performed every 6 months up to 2 years after the last dose of the study drug for evaluation of potential PML risk of the drug.

6.2.5 Justification of Endpoints

The Mayo score was used as the disease activity index for UC in C13006 study[8]. The Mayo score, extensively used efficacy index for evaluation of UC inside/outside Japan, was selected as the evaluation index in the study. The clinical response and clinical remission based on the Mayo score were defined in the same manner as those in C13006.

6.3 Premature Termination or Suspension of Study or Investigational site

6.3.1 Criteria for Premature Termination or Suspension of the Study

The study will be completed as planned unless one or more of the following criteria are satisfied that require temporary suspension or early termination of the study.

- New information or other evaluations regarding the safety or efficacy of the study drug that indicates a change in the known risk/benefit profile for the compound, such that the risk/benefit is no longer acceptable for subjects participating in the study.
- Significant violation of Good Clinical Practice (GCP) that compromises the ability to achieve the primary study objectives or compromises subject safety.

6.3.2 Criteria for Premature Termination or Suspension of Investigational Sites

A study site may be terminated prematurely or suspended if the site (including the investigator) is found in significant violation of GCP, protocol, or contractual agreement, is unable to ensure adequate performance of the study, or as otherwise permitted by the contractual agreement.

6.3.3 Procedures for Premature Termination or Suspension of the Study or the Participation of Investigational Site(s)

When the sponsor, the IRB or regulatory authorities has decided to suspend or terminate the study or participation of a study site, the sponsor will designate a procedure to be applied in the study. The study site should follow the designated procedures to suspend or terminate the study.

7.0 SELECTION AND DISCONTINUATION/WITHDRAWAL OF SUBJECTS

All inclusion/exclusion criteria including test results must be confirmed prior to enrollment into the induction phase.

7.1 Inclusion Criteria

Subject eligibility is determined according to the following criteria:

1. In the opinion of the investigator, a subject is capable of understanding and complying with protocol requirements.
2. A subject who is capable of entering the signature and the date on the informed consent by himself/herself or by the subject's legally acceptable representative, if applicable, prior to initiation of study procedures.
3. A subject aged 15 to 80 at the time of signing the informed consent (regardless of sexes).
4. A male subject, who has no sterilization history and whose female partner has child-bearing potential, who agreed with taking proper contraception during the period from the time of signing on the informed consent form through 6 months after the last dose of the study drug.
5. A female subjects with child-bearing potential (having no history of sterilization or whose last menstruation was within 2 years) whose male partner is not receiving contraceptive treatment, and agreed to take proper contraception during the period from the time of signing on the informed consent form through 6 months after the last dose of the study drug.
6. Subjects with diagnosis of total or left-sided UC based on the Revised Diagnostic Criteria for UC issued by "Research Group for Intractable Inflammatory Bowel Disease Designated as Specified Disease" by the Ministry of Health, Labor and Welfare (MHLW) of Japan (2012) (see Appendix C) at least 6 months before the start of administration of the study drug.
7. A subject with moderately or severely active UC as determined by baseline complete Mayo score of 6 to 12 (inclusive) with an endoscopic subscore of ≥2.
8. Subjects whose complication of colon cancer or dysplasia had to be ruled out by total colonoscopy at the start of the study drug administration (or the results from total colonoscopy performed within 1 year before giving consent are available), if subjects met any of the following criteria; subjects with ≥8-year history of total or left-sided colitis, subjects aged ≥50 years, or subjects with a first-degree family history of colon cancer.
9. Subjects meeting the following treatment failure criteria with at least one of the following agents within 5 years before the time of signing on the informed consent form:

- Corticosteroids
  - Resistance: subjects whose response was inadequate after treatment of ≥40 mg/day for ≥1 week [3](oral or IV) or 30 to 40 mg/day for ≥2 weeks (oral or IV).
  - Dependence: subjects for which it is difficult to reduce the dosage to <10 mg/day due to recurrence during gradual dose reduction (oral or IV).
  - Intolerance: subjects who were unable to receive continuous treatment due to adverse reactions (e.g., Cushing's syndrome, osteopenia/osteoporosis, hyperglycaemia, insomnia, infection).
- Immunomodulators (AZA or 6-MP)
  - Refractory: subjects whose response was inadequate after treatment for ≥12 weeks.
  - Intolerance: subjects who were unable to receive continuous treatment due to adverse reactions (e.g., nausea/vomiting, abdominal pain, pancreatitis, LFT abnormalities, lymphopenia, TPMT genetic mutation, infection).
- Anti-TNFα antibodies
  - Inadequate response: subjects whose response was inadequate after the induction therapy in the dosage described in the package insert.
  - Loss of response: subjects who had recurrence during the scheduled maintenance therapy after achievement of clinical response (those who withdrew for other reasons than relapse are not applicable here).
  - Intolerance: subjects who were unable to receive continuous treatment due to adverse reactions (e.g., infusion-related reaction, demyelination disease, congestive heart failure, infection).

7.2 Exclusion Criteria

Subjects meeting any of the following criteria will not be eligible in the study:

1. Subjects whose partial Mayo score decrease by 3 points or more between screening and the start of study drug administration.
2. Subjects having or suspected to have abdominal abscess or toxic megacolon.
3. Subjects with a history of subtotal or total colectomy.
4. Subjects with ileostomy, colostomy, fistula or severe intestinal stenosis.
5. Subjects having a treatment history with natalizumab, efalizumab or rituximab.
6. Subjects who started oral 5-ASA, probiotics, or oral corticosteroids (≤30 mg/day) within 13 days before the first dose of the study drug. Subjects who have used these drugs for at least

[3]The dosages of corticosteroids represent, hereafter, the converted dosage of prednisolone.

14 days before the first dose of the study drug, and who changed dosage of or discontinued these drugs within 13 days before the first dose of the study drug.

7. Subjects who have received 5-ASA, corticosteroid enemas/suppositories, corticosteroid IV infusion, oral corticosteroid at >30 mg/day, drugs for diarrhea-predominant irritable bowel syndrome, or Chinese herbal medicine for the UC treatment (e.g., Daikenchuto) within 13 days before the first dose of the study drug.
8. Subjects who have used an antidiarrheal drug for 4 or more consecutive days within 13 days before the first dose of the study drug or within 7 days before the first dose of the study drug.
9. Subjects who have received AZA or 6-MP within 27 days before the first dose of the study drug However, this will not apply to subjects who have used these drugs for 83 or more days before the first dose of the study drug and continued the steady dose administration of the drugs for 27 or more days before the first dose of the study drug.
10. Subjects who have received cyclosporine, tacrolimus, methotrexate, tofacitinib or any study drugs of low-molecular compound for UC treatment within 27 days before the first dose of the study drug.
11. Subjects who have received adalimumab within 27 days before the first dose of the study drug or any biologic agents other than adalimumab within 55 days before the first dose of the study drug. However, this will not apply to subjects who have topically received these drugs (e.g., intraocular injection for treatment of age-related macular degeneration).
12. Subjects who have received any live-vaccinations within 27 days before the first dose of the study drug.
13. Subjects who underwent the enterectomy within 27 days before the first dose of the study drug or those anticipated to require an enterectomy during the study.
14. Subjects who have received leukocytapheresis or granulocyte apheresis within 27 days before the first dose of the study drug.
15. Subjects who have been infected with an intestinal pathogen including clostridium difficile or cytomegalovirus within 27 days before the first dose of the study drug.
16. Subjects with evidence of adenomatous colonic polyps that need to be removed at the start of study drug administration.
17. Subjects with a history or a complication of colonic mucosal dysplasia.
18. Subjects suspected to have enteritis other than UC.
19. Subjects indicated in the screening test as HBs antigen-positive or HCV antibody-positive Subjects indicated as HBc antibody-positive or HBs antibody positive even though HBs antigen-negative However, the criteria will not apply to those with only HBs antibody-positive due to HBV vaccination, HBV-DNA-negative, HCV antigen-negative or HCV-RNA-negative.

20. Subjects who have or are suspected to have a history of tuberculosis (including those whose findings in the chest imaging procedure at screening showing anamnesis of tuberculosis) However, the criteria will not apply to those who had completed prophylactic treatment with isoniazid, and who have been receiving prophylactic isoniazid for 21 days or longer before the first dose of the study drug (the latter may initiate study drug administration with screening phase extended to 28 days at maximum for prophylactic treatment to become 21 days or more).
21. Subjects indicated as positive in T-SPOT or QuantiFERON at screening test.
22. Subjects who have a history or complication of congenital or acquired immunodeficiency syndrome (e.g., not-classifiable immunodeficiency, HIV infection or organ transplantation).
23. Subjects who were affected by extraintestinal infection (e.g., pneumonia, sepsis, active hepatitis or pyelonephritis) within 27 days before the first dose of the study drug.
24. Subjects who have treatment history with MLN0002.
25. Nursing mothers during the screening phase, or female subjects indicated positive in urine pregnancy test either at the screening or baseline.
26. Subjects having serious complications in the heart, lung, liver, kidney, metabolism, gastrointestinal system, urinary system, endocrine system or blood.
27. Subjects with a history of an operation requiring general anesthesia within 27 days before the first dose of the study drug, or with a schedule of an operation requiring hospitalization during the study period.
28. Subjects having a complication or a history of malignancy However, it will not apply to the following subjects;
    - Subjects who had a curative resection of localized skin basal cell carcinoma or had completed curative radiotherapy.
    - Subjects who have not experienced recurrence for 1 year or longer since completion of curative resection or curative radiotherapy for skin squamous cell carcinoma.
    - Subjects who have not experienced recurrence for 3 year or longer since completion of curative resection or curative radiotherapy for intraepithelial carcinoma of uterine cervix.

    For subjects having a substantially distant history of malignancy (e.g., 10 years or longer without recurrence since treatment completion), the Investigator and the sponsor will discuss to decide eligibility on the basis of type of malignancy and treatment applied.
29. Subjects having a history or a complication of the central nervous disorder, including stroke, multiple sclerosis, brain tumor, or neurodegenerative disease.
30. Subjects for which any subjective symptoms in the Subjective PML checklist were found at the screening or baseline.
31. Subjects for which any of the following laboratory abnormalities were found at the screening;

- Hemoglobin ≤8 g/dL
- White blood cells ≤3,000/μL
- Lymphocytes ≤500/μL
- Platelets ≤100,000/μL, or ≥1,200,000/μL
- ALT and AST ≥3×upper limit of normal (ULN)
- ALP ≥3×ULN
- Creatinine ≥2×ULN

32. Subjects having a history or a complication of alcohol dependence or illicit drug use within one year before the first dose of the study drug.
33. Subjects having a history or a complication of psychotic disorder that may obstruct compliance with the study procedures.

7.3 Excluded Medications and Treatments

Excluded medications and treatments are listed in Table 7.a. However, some of the drugs may be used concurrently under the conditions provided in the table. Subjects will be instructed not to take any drugs, including over-the-counter products (OTCs), without first consulting with the investigator.

Table 7.a List of Excluded Drugs and Treatments

Drugs/Treatment	Prohibited Period [Note1)]			Conditions in Concurrent Use	
				Screening Phase/ Induction Phase/Maintenance Phase	Open-label Cohort
Oral 5-ASA<br>Probiotics [Note2)]	Day-13	–	16 weeks after the last dose	Should be initiated before Day-14 and continued at the same dosage from Day-13. Dose reduction or discontinuation will be allowed only due to adverse reactions.	Can be used (without limitation)
Oral corticosteroids (≤30 mg/day)	Day-13	–	16 weeks after the last dose	Should be initiated before Day-14 and continued at the same dosage from Day-13. As per the dose reduction schedule for corticosteroids (see Section 7.3.2).	Same as on the left
5-ASA enemas<br>5-ASA suppositories	Day-13	–	16 weeks after the last dose	–	Can be used (without limitation)
Corticosteroid enemas<br>Corticosteroid suppositories<br>(including corticosteroid pretreatment prior to endoscopy)	Day-13	–	16 weeks after the last dose	–	Can be used (without limitation)
Corticosteroid IV infusion	Day-13	–	16 weeks after the last dose	–	–
Oral corticosteroids (>30 mg/day)	Day-13	–	16 weeks after the last dose	–	–
Therapeutic drugs for diarrhea-predominant irritable bowel syndrome	Day-13	–	16 weeks after the last dose	–	–

Drugs/Treatment	Prohibited Period Note1)			Conditions in Concurrent Use	
				Screening Phase/ Induction Phase/Maintenance Phase	Open-label Cohort
Chinese herbal medicines for UC treatment (e.g., Daikenchuto)	Day-13	–	16 weeks after the last dose	–	–
Antidiarrheal drugs	Day-13	–	16 weeks after the last dose	Not used within 7 days before evaluation of the Mayo score. Not used for ≥4 consecutive days.	Can be used (without limitation)
AZA<br>6-MP	Day-27	–	16 weeks after the last dose	Should be initiated before Day-83 and continued at the same dosage from Day-27. Dose reduction or discontinuation will be allowed only due to adverse reactions.	Same as on the left
Tacrolimus<br>Cyclosporine<br>Methotrexate<br>Tofacitinib	Day-27	–	16 weeks after the last dose	–	–
Study drugs of low-molecular compounds for UC treatment	Day-27	–	16 weeks after the last dose	–	–
Adalimumab	Day-27	–	16 weeks after the last dose	–	–
Natalizumab<br>Efalizumab<br>Rituximab<br>MLN0002	(previous)	–	16 weeks after the last dose	–	–
Other biological drugs than the above (including infliximab and excluding topical administration)	Day-55	–	16 weeks after the last dose	–	–
Live-vaccinations	Day-27	–	6 months after the last dose	–	–
Enterectomy [Other surgical treatments including (treatment-emergent) abscess drainage or the seton method for the anal lesion may be performed than the enterectomy]	Day-27	–	16 weeks after the last dose	–	–
Leukocytapheresis or granulocyte apheresis	Day-27	–	16 weeks after the last dose	–	–
Surgeries requiring general anesthesia	Day-27	–	16 weeks after the last dose	–	–

Note 1) Day 1 is defined as the date of the first dose of the study drug and Day -1 is defined as the day before the Day 1 for calculation

Note 2) Such as VSL#3. The bifidobacteriam preparation or any foods are not applicable.

7.3.1 Rescue Treatments

Drugs or treatments not permitted in Table 7.a may be used as rescue treatments (see Section 3.1 for the definition) only in urgent cases such as worsening of UC otherwise safety of the subject is judged not to be secured.

The subjects who received any rescue treatments in the induction phase should withdraw from the study. The subjects who received any rescue treatments in the maintenance phase should discontinue the maintenance phase and may be enrolled into the open-label cohort, however

withdrawal should be considered in the subjects intending to receive any unavailable treatment in the open-label cohort. For the subjects who received any rescue treatments in the open-label cohort, withdrawal should be considered (see Section 7.5).

7.3.2 Dose Reduction Schedule of Corticosteroids

Dosage of a corticosteroid, if used concurrently, should not be modified until Week 10 after the first dose of the study drug in principle. When the clinical response (see Section 3.1 for the definition) was achieved at Week 10 or later, the dose reduction of the corticosteroid will be initiated as per the following schedule from the visit when the clinical response was observed, where possible.

Dose Reduction Schedule:

- When >10 mg/day was dosed, the dosage will be gradually reduced by 5 to 10 mg/2 weeks to reach 10 mg/day
- When ≤10 mg/day was dosed or achieved by gradual reduction, the dosage will be gradually reduced by 2.5 to 5 mg/2 weeks to achieve corticosteroid-free

When dose reduction is required earlier than Week 10 due to adverse reactions of the corticosteroid, only small doses of not more than 5 mg/2 weeks should be gradually reduced.

When any symptoms recur with the dose reduction of the corticosteroid, the dosage may be increased up to the initial dose (it will not apply to rescue treatments). Subsequently, when clinical response was observed again, the dose reduction will be initiated preferably as per the above schedule from the visit when the clinical response was observed.

Of note, higher dosage than the initial dosage, if used for recurrence, will be considered as a rescue treatment (see Section 7.3.1 for rescue treatments).

7.4 Subject Management

1. Subjects should be instructed to be sure to see the physician and take specified examinations at the specified date and time. In addition, subjects should be instructed to inform the investigator or the sponsor immediately in the case that they cannot visit.
2. Subjects should be instructed to make entries in the subject’s diary throughout the period from the time of signing on the informed consent form to the last dose of the study drug and to bring the diary with them at each visit.
3. Subjects should be instructed to preferably visit under fasted condition at visits for blood biochemistry test.
4. Subjects should be instructed to report any worsening of symptoms that occurred at any time other than visits, if any, to the investigator or the sponsor via telephone or the like to seek instructions.

5. Subjects should be instructed to report details, onset date, intensity, outcome and outcome date of subjective symptoms/objective findings to the investigator during their visit.
6. Subjects should be instructed to report course and details of the treatment to the investigator if they visited other medical institutions during the period from the time of signing on the informed consent form to 16 weeks after the last dose of the study drug.
7. Subjects should be instructed to inform a physician in a medical institution of his/her participation in the study, if he/she visited other medical institutions during the period from the time of signing on the informed consent form to 16 weeks after the last dose of the study drug.
8. Subjects should be instructed not to use any drugs including OTCs other than drugs instructed by the investigator without prior consultation (except urgent cases).
9. Male subjects without a sterilization history and whose female partner has child-bearing potential should be instructed to take proper contraception during the period from the time of signing on the informed consent form to 6 months after the last dose of the study drugs.
10. Female subjects with child-bearing potential whose male partner has no sterilization history should be instructed to take proper contraception during the period from the time of signing on the informed consent form to 6 months after the last dose of the study drugs.
11. Subjects should be instructed to avoid surfeit, extreme changes in diet, excessive exercise/action throughout the period from the time of signing on the informed consent form to the last dose of the study drug. Subjects should be instructed in an especially careful manner because treatment-emergent change of symptoms may lead to changes in diet.
12. Subjects should be instructed not to donate their blood during the period from the time of signing on the informed consent form to 6 months after the last dose of the study drug.
13. Subjects should be instructed to report any feelings of infusion reaction (e.g., rash, pruritus, flushing or urticaria) to the investigator immediately.

7.5 Criteria for Discontinuation or Withdrawal of a Subject

The primary reason for discontinuation or withdrawal of a subject from the study should be recorded in the case report form ([e]CRF) using the following categories. See Section 9.1.22 for subjects who withdrew during the screening phase.

1. **Pretreatment Events or AEs**
   The subject has experienced a PTE or AE that requires early termination because continued participation imposes an unacceptable risk to the subject's health or the subject is unwilling to continue because of the PTE or AE.
   - Liver Function Test Abnormalities
     Discontinuation of the study drug should be considered with appropriate clinical follow-up (including repeat laboratory tests, until a subject's laboratory profile has

returned to normal/baseline status, see Section 9.1.12), if the following circumstances occur at any time during study drug treatment:

- ALT or AST >8×ULN, or
- ALT or AST >5×ULN and persists for more than 2 weeks, or
- ALT or AST >3×ULN in conjunction with elevated total bilirubin >2×ULN or international normalized ratio (INR) >1.5, or
- ALT or AST >3×ULN with appearance of fatigue, nausea, vomiting, right upper quadrant pain or tenderness, fever, rash and/or eosinophilia.

2. **Major protocol deviation**
The discovery post-randomization in the induction phase that the subject failed to meet protocol inclusion criteria or did not adhere to protocol requirements, and continued participation poses an unacceptable risk to the subject's health.

Note: The visit for evaluation of the primary efficacy endpoint in the induction phase (Visit 5), when notably deviated beyond the acceptable window (≥±15 days), should be considered as a major protocol deviation.

3. **Lost to follow-up**
The subject did not return to the clinic and attempts to contact the subject were unsuccessful. Attempts to contact the subject must be documented.

4. **Voluntary withdrawal.**
The subject wishes to withdraw from the study. The reason for withdrawal, if provided, should be recorded in the (e)CRF.

Note: All attempts should be made to determine the underlying reason for the withdrawal and, where possible, the primary underlying reason should be recorded (i.e., withdrawal due to an AE or lack of efficacy should not be recorded in the "voluntary withdrawal" category).

5. **Study termination**
The sponsor, the IRB or regulatory authorities decided to terminate the study.

6. **Pregnancy**
The subject is found to be pregnant.

Note: If the subject is found to be pregnant, the subject must be withdrawn immediately. The procedure is described in Section 9.1.14.

7. **Lack of efficacy**
The investigator has determined that the subject is not benefiting from investigational treatment; and, continued participation would pose an unacceptable risk to the subject.

Note: A subject who received any rescue treatments due to lack of efficacy in the induction phase should withdraw from the study immediately. For a subject who received any rescue

treatments in the open-label cohort, and meets criteria for the sustained non-response or the disease worsening, withdrawal due to lack of efficacy will be considered

8. **Other**
   Note: The specific reasons should be recorded in the "specify" field of the (e)CRF.

7.6 Procedures for Discontinuation or Withdrawal of a Subject

The investigator may terminate a subject's study participation at any time during the study when the subject meets the study termination criteria described in Section 7.5. In addition, a subject may discontinue his or her participation without giving a reason at any time during the study. Should a subject's participation be discontinued, the primary criterion for termination must be recorded. In addition, efforts should be made to perform all procedures scheduled for the early termination visit.

8.0 CLINICAL STUDY MATERIAL MANAGEMENT

This section contains information regarding all drugs and materials provided directly by the sponsor, and/or by other means, that are required by the study protocol, including important sections describing the management of clinical trial materials.

8.1 Study Medication and Materials

8.1.1 Dosage Form, Manufacturing, Packaging, and Labeling

8.1.1.1 Study Drug

In this protocol, the term of study drugs refers to all or any of the drugs defined below.

Table 8.a Study drug

Name of the study drugs	Chemical name of active ingredients (Nonproprietary names)	Formulations	Contents
MLN0002<br>300 mg for infusion	Human IgG_1 monoclonal antibody [vedolizumab (INN)] against human $\alpha_4\beta_7$ integrin	Parenteral solution	Contains 300 mg* of MLN0002 in one vial
Placebo for infusion	Not applicable	–	Empty

*lyophilized preparation

Appearance is identical among vials of the study drugs. Each vial will be labeled to be packed into the outer package. The outer package will be labeled with the statement that contents are study drugs, the name of the study drug, quantity, the name and address of the sponsor, the serial number, the storage and the expiration date. Examples of labels on vials or outer packages are shown in the Figure 8.a and Figure 8.b, respectively. Of note, the serial numbers and expiration dates of the study drugs should be in accordance with the separately specified written procedure.

Figure 8.a Labels on Vials

MLN0002/CCT-101<br>MLN0002 (300 mg) for injection, for intravenous use<br>Serial number: Z	MLN0002/CCT-101<br>NOT for infusion to subjects
For vials of MLN0002 (300 mg)	For vials of placebo

Figure 8.b A Label on Outer Packages

Study drug 1 vial<br><br>MLN0002 DB for infusion<br>MLN0002/CCT-101<br><br>Drug number:<br><br>Subject Identification Code:<br><br>Timing for infusion:<br><br>Week ____________	Serial number: DB...........<br>Expiration date: See the procedure for study drug control<br>Storage: refrigerated (2 to 8°C)<br><br>Note<br>The outer package should be kept to be collected by the Sponsor.<br><br>Takeda Pharmaceutical Company Limited<br>1-1, Doshomachi 4-chome, Chuo-ku, Osaka-shi

8.1.1.2 Materials

The following materials will be used at the time of infusion of the study drugs in principle. However, the material control should be in accordance with the separately specified written procedures.

Table 8.b Materials

Intended use	Materials	Examples
Preparation of study drugs	A syringe for preparation	A 5 or 10 cc latex-free syringe
	Water for injection	Water for injection in a container with volume of 10 mL or larger
	An injection needle for preparation	An injection needle of 18G stainless steel
	Physiological saline	Sterilized 100 mL (specification) 0.9% NaCl solution
Infusion of study drugs	An IV infusion kit	An IV infusion kit to set the injection site beneath an infusion tube
	An injection needle for infusion	A winged needle for IV infusion
	Physiological saline (for priming/flushing IV infusion lines)	Sterilized 50, 100 or 250 mL (specification) 0.9% NaCl solution

8.1.2 Storage

Temperature during storage should be refrigerated (2 to 8°C). However, it may be deviated temporally (for 7 days) in the range from -70 to 40°C.

All study drugs must be kept in an appropriate, limited-access, secure place until used or returned to the sponsor or its designee for destruction. All sponsor-supplied study drugs must be stored under the conditions specified on the label, and remain in the original container until dispensed. A daily temperature of the study drug storage area must be determined and recorded every working day.

8.1.3 Dose and Regimen

Table 8.c Dose and Regimen

Study Drugs	Preparation and Regimen
Placebo for infusion	Add 5 mL of water for injection to approximately 100 mL of saline and infuse intravenously over 30 minutes*.
MLN0002 (300 mg) for infusion	One vial of MLN0002 (300 mg) for infusion is reconstituted in 4.8 mL of water for injection. Add 5.0 mL of the solution to approximately 100 mL of saline for dilution and infuse intravenously over 30 minutes*.

*May be infused over 60 minutes at maximum.

Study drug preparators should prepare study drugs. A study drug preparators should not be an evaluator throughout induction and maintenance phases. Study drug preparators will prepare study drugs in line with the drug numbers prescribed by the investigator or his/her designee. The details of the study drug preparation method should be in accordance with the separately specified written procedure.

Serious infusion reaction should be treated properly, if any, e.g., immediate use of epinephrine and parenteral diphenhydramine.

8.1.4 Overdose

Overdose is defined as a known deliberate or accidental administration of a study drug at a dose above the specification in the study protocol.

All cases of overdose (with or without associated AEs) will be documented on an Overdose page of the (e)CRF, in order to capture this important safety information consistently in the database. AEs associated with an overdose will be documented on AE (e)CRF(s) according to Section 10.0, PTEs and AEs.

SAEs associated with overdose should be reported in accordance with the procedure specified in Section 10.2.2 Collection and Reporting of SAEs.

Subjects overdosed should be treated by the investigator based on symptoms.

8.2 Procedures for Allocation, Prescription and Infusion of Study Drugs

A study drug number will not be identical at each time. For allocation of subjects to study drugs, Investigators or his/her designee will access the website of the registration center at each visit for infusion to obtain a study drug number from the registration center. Investigators or his/her designee will prescribe the study based on the obtained study drug number. The study drug number should be recorded in (e)CRF at each administration visit.

Details for using the website of the registration center will be in accordance with the separately specified written procedures.

8.3 Preparation and Retention of the Allocation Table

A person in charge of allocation (appointed by the sponsor) will prepare the allocation table. Information of allocation should be kept in a secure place with limited access by unauthorized people.

8.4 Preservation of Blindness

The emergency key will be kept in the emergency key control center until emergent key-breaking or completion of database lock of all subjects.

In the study, allocations must not be disclosed to any person until opening of the study drug allocation table, while the study drug preparators may be aware of allocations in the course of study drug preparation.

Furthermore, test results of serum drug concentration and HAHA/neutralizing antibody may be an obstacle in preservation of blindness in the study, therefore these final test results should be kept undisclosed outside in the test laboratory until opening of the study drug allocation table, and will be reported to the investigator through the sponsor upon opening. However, if a test result must be disclosed before opening, the test result may be reported to the sponsor or the like after necessary actions taken to preserve blindness (e.g., renumbering of study drugs), so that persons in charge in the test laboratory would be unable to identify subjects. Detailed procedures should be in accordance with the separately specified written procedure.

8.5 Procedures for Blind-Breaking

The study drug blind will not be broken by the investigator unless information concerning the study drug is necessary for the medical treatment of the subject.

In blind-breaking, the investigator may obtain study drug allocation information from the emergency key control center (see the Annex for contacts).

In blind-breaking, the investigator will enter the date/time and rationale of the blind-breaking on the record of emergency key opening to be submitted to the sponsor. The same information (except time) will be recorded on (e)CRF.

If any site personnel is unblinded, the study drug must be stopped immediately and the subject must be withdrawn from the study.

8.6 Control and Disposal of Sponsor-Supplied Drugs

The Study Drug Administrator will receive the written procedure prepared by the sponsor relating to handling, storing and controlling study drugs to properly control sponsor-supplied study drugs and materials in accordance with the written procedure. Similarly, the investigator will receive the same written procedures from the sponsor. The written procedure describes procedures to ensure proper receipt, handling, storing, controlling and prescription of the sponsor-provided study drugs and materials, as well as return of unused study drugs and materials to the sponsor or other disposition.

The Study Drug Administrator will return unused study drugs and materials to the sponsor immediately after study completion in the study site.

9.0 STUDY PLAN

9.1 Study Procedures

The following sections describe the study procedures and data to be collected. For each procedure, subjects should be assessed by the same investigator or site personnel, where possible. The Study schedule is provided in Appendix A.

9.1.1 Informed Consent Procedure

The requirements of the informed consent are described in Section 15.2.

The informed consent must be obtained prior to the subject entering into the study, and before any protocol-directed procedures are performed.

Dosage of prior therapeutic drugs will be modified or discontinued for the study enrollment, if needed, after acquisition of the informed consent for participation in the study.

Subject Identification Code will be provided for each subjects at the time of explanation of the informed consent, and used throughout the study.

Pharmacogenomic Informed Consent Procedure

A separate informed consent form pertaining to storage and banking of the sample must be obtained prior to collecting a blood sample for Pharmacogenomic Research for this study. The provision of consent to collect and analyze the pharmacogenomic sample is independent of consent to the other aspects of the study.

9.1.2 Demographics, Medical History, and Medication History Procedure

As demographic data, dates of birth, sex and smoking histories will be obtained. In addition, the following UC-related items will be obtained.

- Date Primary Disease Diagnosed
- The following item as defined in the Revised Diagnostic Criteria for UC (Appendix C) issued by “Research Group for Intractable Inflammatory Bowel Disease” Designated as Specified Disease by the MHLW of Japan (2012)
- Disease classification by extent of the UC lesion (total colitis or left-sided colitis)
- History of treatment failure with the following drugs within 5 years before acquisition of the informed consent
  - Corticosteroids (resistance/dependence/intolerance)
  - Immunomodulators (AZA or 6-MP) (refractory/intolerance)
  - Anti-TNFα antibodies* (inadequate response/loss of response/intolerance)

* For inadequate response and loss of response, the name and the first and last administration dates of the drug will be recorded as well. For intolerance, the name of the drug and the side-effect leading to discontinuation will be recorded as well.

For disease histories, any clinically relevant diseases or symptoms will be obtained, which were judged as disease histories in evaluation in the study by a physician, including extra-intestinal manifestations, pouchitis, infectious enteritis, toxic megacolon, stenosis, fistula that has recovered/resolved within 1 year before acquisition of the informed consent. In addition, any previous malignancies and enterectomy for UC will be obtained. Ongoing conditions or diseases are considered as concurrent medical conditions (see Section 9.1.7).

For prior therapeutic drugs, all of the following will be obtained.

- Biological drugs for UC treatment discontinued before acquisition of the informed consent.
- Low-molecular compounds for UC treatment discontinued within 4 weeks before acquisition of the informed consent.

Of note, agents applied for procedures including endoscopy will not be considered as prior therapeutic drugs and also not be documented in the (e)CRF.

9.1.3 Physical Examination Procedure

Physical examination in the following body sites will be performed before study drug administration if it is the day of study drug administration.
(1) eyes, (2) ears, nose, throat, (3) cardiovascular system, (4) respiratory system,
(5) gastrointestinal system, (6) dermatologic system, (7) extremities, (8) musculoskeletal system,
(9) nervous system, (10) lymph nodes, and (11) other. During infusion and 1 hour after infusion of the study drug, infusion reactions (e.g., rash, pruritus, flushing or urticaria) will be observed, and clinically relevant abnormalities will be assessed as compared to physical examination results performed before study drug administration.

9.1.4 Weight, Height and BMI

Body weight and height will be measured before administration of the study drug if it is the day of study drug administration. In addition, BMI will be calculated by the sponsor with the following formula;

$$\text{Metric: } \quad BMI = \text{weight (kg)}/\ [\text{height (m)}]^2$$

Height will be collected in centimeters without decimal places and weight will be collected in kilograms to 1 decimal place, results for BMI will be expressed with 1 decimal place.

9.1.5 Vital Sign Procedure

As vital signs, body temperature (axilla), blood pressure while sitting (rest for 5 minutes or longer), and pulse (bpm) will be measured before administration of the study drug if it is the day of study drug administration.

9.1.6 Documentation of Concomitant Medications and Non-drug Therapy

Concomitant medication is any drug given in addition to the study medication. These may be prescribed by a physician or obtained by the subject over the counter. Concomitant medication is not provided by Takeda. At each study visit, subjects will be asked whether they have taken any drug other than the study drug (used from the time of signing on the informed consent form through the end-of-study examination), and all drugs including vitamin supplements, OTC drugs, and oral herbal preparations, must be recorded in the (e)CRF. In addition, for concomitant drugs for UC treatment, class of the concomitant drug (5-ASA, corticosteroids, immunomodulators, or others), and applicability to a rescue treatment will be recorded in the (e)CRF.

Concomitant treatments are defined as any treatments for UC other than pharmacotherapy, including enterectomy and other surgical treatments (e.g., abscess drainage or the seton method for the anal lesion). All concomitant treatments that were conducted during the period from acquisition of the informed consent until the end-of-study examination will be obtained. Additionally, applicability of the concomitant treatment to a rescue treatment will be recorded in the (e)CRF.

Of note, agents applied for test procedures including endoscopy will not be considered as concomitant drugs and will also not be documented in the (e)CRF.

9.1.7 Documentation of Concurrent Medical Conditions

Concurrent medical conditions are those significant ongoing conditions or diseases that are present at the time of signing on the informed consent form. This includes clinically significant laboratory, ECG, or physical examination abnormalities noted at screening/baseline examination. The condition (i.e., diagnosis) should be described. In addition, UC-related extra-intestinal manifestations will be recorded in the (e)CRF.

9.1.8 The Patient Diary

The patient diaries are used for calculation of the Mayo scores. At each visit, patient diaries to be entered from the visit to the next visit will be handed to subjects. A subject will be instructed to record the following items in the diary and bring it at the next visit.

- Stool frequency
- Rectal Bleeding
- Use of antidiarrheal drugs

Contents of the patient diaries will be confirmed at each visit. The subject will be asked about omissions or inconsistencies for correction, if any. The patient diaries will be collected to be retained after final confirmation of the contents.

9.1.9 Complete Mayo Score and Partial Mayo Score

The complete Mayo scores will be calculated as total scores of the following 4 subscores in the Table 9.a. The partial Mayo score will be calculated as total scores of the following 3 subscores except the subscore for the findings on endoscopy.

Table 9.a Mayo Scoring System for the Assessment of UC Activity[13]

Stool Frequency
0: Normal no. of stools for this patient
1: 1 to 2 stools more than normal
2: 3 to 4 stools more than normal
3: 5 or more stools more than normal
Rectal Bleeding
0: No blood seen
1: Streaks of blood with stool less than half the time
2: Obvious blood with stool most of the time
3: Blood alone passes
Findings on Endoscopy
0: Normal or inactive disease
1: Mild disease (erythema, decreased vascular pattern, mild friability)
2: Moderate disease (marked erythema, lack of vascular pattern, friability, erosions)
3: Severe disease (spontaneous bleeding, ulceration)
Physician's Global Assessment
0: Normal
1: Mild disease
2: Moderate disease
3: Severe disease

At screening test (Visit 1), the investigator will interview about the average number of stools, condition of rectal bleeding and general condition during the previous 3 days, and evaluate each subscore based on the outcome of the interview to calculate the partial Mayo score. Each subscore for stool frequency, rectal bleeding and physician's global assessment will be entered on the website of the registration center to calculate the partial Mayo score.

Since the first dose of the study drug (Visit 2), data in the patient diary, the subscores for the findings on endoscopy and physician's global assessment will be entered on the website of the registration center to calculate the complete Mayo score or the partial Mayo score. The methodology for calculating each subscore is shown below.

9.1.9.1 Calculation of Subscores for Stool Frequency and Rectal Bleeding

For calculation of subscores for stool frequency and rectal bleeding, the contents in the patient diary during the previous 7 days before evaluation will be entered on the website of the registration center. Each subscore will be calculated automatically based on the contents in the patient diary during the last 3 days before evaluation except for the following.

- The day of pretreatment for endoscopy
- The day of endoscopy and the next day
- The day of usage of antidiarrheal drugs and the next day

Of note, for baseline evaluation (Visit 2), the contents of the patient diary during at least 3 days except for the above exception days should be entered on the website of the registration center for calculation of the complete Mayo score. The visit schedule will be adjusted to have the necessary number of days in the patient diary.

9.1.9.2 Calculation of the Subscore for Findings on Endoscopy

Endoscopy will be performed in the rectum and sigmoid colon between 9 days and on the day of evaluation of the complete Mayo score. The investigator will identify the most active site at baseline, and score the identical site for the findings on endoscopy throughout the study period based on Table 9.a. The findings on endoscopy will be scored by the same physician throughout the study, where possible. The obtained subscore for findings on endoscopy will be entered on the website of the registration center.

Additionally, the Clinical Endpoint Committee (CEC; see Section 11.2) will be established in the study, where subscores for findings on endoscopy will be assessed independently. Therefore, investigator should submit clear images to the Sponsor, by which the CEC may assess the subscore for findings on endoscopy.

Of note, the result of endoscopy performed within 9 days before evaluation (before acquisition of the informed consent) may be used for the base line assessment (Visit 2), if available, in the invasiveness point of view.

9.1.9.3 Calculation of Physician's Global Assessment

On the evaluation day, investigator will interview about abdominal discomfort, general condition, principal physician’s comments, subject’s impression or the like to decide the Physician's Global Assessment as referring the result of the interview, stool frequency and rectal bleeding and findings on endoscopy (if performed).

9.1.10 Inflammatory Bowel Disease Questionnaire (IBDQ)

The IBDQ, as composed of a total of 32 questions (each has 1 to 7 points) including abdominal symptoms (10 items), general condition (5 items), emotion (12 items) and social function (5 items),

will be calculated as the total scores, and performed before administration of the study drug if it is the day of study drug administration. Each score will be recorded in the (e)CRF.

9.1.11 UC-related events

UC-related events that occurred during the period from the first dose of the study drug to end of the study will be recorded in the (e)CRF. The types and onset dates of events will be recorded in the (e)CRF, if any, based on the following classification.

- UC-related hospitalization
- Enterectomy

9.1.12 Laboratory Test

Clinical laboratory test items as shown in Table 9.b will be determined. The test samples will be collected before administration of the study drug if it is the day of study drug administration. All samples will be handled in accordance with the separately specified procedures. The maximum amount of blood sampling will be approximately 19 mL at each visit.

Table 9.b Laboratory tests

**Blood biochemistry**		
Albumin<br>Aspartate aminotransferase (AST)<br>Alanine aminotransferase (ALT)<br>Alkaline phosphatase (ALP)<br>Amylase<br>Glucose [Note1)]<br>Total bilirubin<br>Total protein<br>g-glutamyl transpeptidase (γ-GTP)		Total cholesterol<br>Triglyceride [Note1)]<br>Creatinine<br>Blood urea nitrogen (BUN)<br>Uric acid<br>Potassium<br>Sodium<br>Calcium<br>Phosphorus<br>Magnesium<br>Chloride
**Hematology**		**Urinalysis**
Red blood cells<br>White blood cells<br>Hemoglobin<br>Hematocrit<br>Platelets	Neutrophils<br>Eosinophils<br>Basophils<br>Lymphocytes<br>Monocytes	Qualitative (glucose, protein, occult blood, bilirubin, ketone body)<br>Urine pH<br>Urine specific gravity
**Serum immunological test (only at screening)** [Note2)]		**Urine pregnancy test**<br>**(only in female subjects of childbearing potential)**
HBs antigen/antibody, HBc antibody, HCV antibody<br>HIV antibody		Human chorionic gonadotrophin (hCG)
**Inflammatory markers**		
C-reactive protein (CRP)		

Note 1) The samples should be preferably collected under fasted conditions.
Note 2) The test results of the serum immunological tests should be reported to subjects directly by investigator, and not be provided to the sponsor.

The above laboratory test items will be determined at the laboratory site for the study except urine pregnancy tests. The urine pregnancy tests will be performed at the study sites. Investigator will assess and retain the clinical laboratory test results reported.

If subjects experience ALT or AST >3×ULN, follow-up laboratory tests (at a minimum, ALP, ALT, AST, total bilirubin, γ-GTP, and INR) should be performed preferably within 48 to 72 hours or within 7 days at the latest after the blood sampling (see Section 7.5 and Section 10.2.3).

If the ALT or AST remains elevated >3×ULN on these 2 consecutive occasions, the investigator must contact the Medical Monitor for consideration of additional testing, close monitoring, possible discontinuation of the study drug, discussion of the relevant subject details and possible alternative etiologies.

Investigators will retain references of the clinical laboratory tests and their tracking.

9.1.13 Contraception and Pregnancy Avoidance Procedure

During the period from the time of signing on the informed consent form to 6 months after the last dose of the study drug, female subjects of childbearing potential (who have no history of sterilization or have menstruation within previous 2 years) must use proper contraception. In addition, during the period from the time of signing on the informed consent form to 6 month after the last dose of the study drug, male subjects without history of sterilization and whose female partner has child bearing potential must prevent pregnancy. Such subjects will be provided with information on acceptable methods of contraception as part of the subject informed consent process, and will be asked to sign a consent form stating that they understand the requirements for avoidance of pregnancy during the course of the study. During the course of the study, the specified urine pregnancy tests will be performed, and subjects will receive continued guidance with respect to avoiding pregnancy.

Urine pregnancy tests performed at screening and at immediately before the first dose of the study drug should be confirmed to show negative.

In addition, male subject must be advised not to donate sperm from the time of signing on the informed consent form to 6 months after the last dose of the study drug.

9.1.14 Pregnancy

If any subject is found to be pregnant during the study, she should be withdrawn and any sponsor-supplied study drugs should be immediately discontinued.

Any pregnancies occurred during the period from the first dose of the study drug (Visit 2) to 6 months after the last dose of the study drug should be reported, using the given report form, to the contact listed in the Annex. In addition, any pregnancies in the partner of a male subject during the

period from the first dose of the study drug (Visit 2) to 6 months after the last dose of the study drug should also be recorded following authorization from the subject's partner.

Should the pregnancy occur during or after administration of the blinded drug, the investigator must inform the subject of their right to receive treatment information. Upon subject's request, the investigator will open the Emergency Key. Subjects revealed not to have received MLN0002 in the study are not required to be followed.

Upon consent with the female subject or the female partner of the male subject, the investigator should inform their physician (an obstetrician) that the subject/female partner of the subject was participating in the clinical study at the time of conception with details of treatment the subject received (blinded or unblinded, as applicable).

All reported pregnancies will be followed up to final outcome, using the pregnancy form. The outcome, including any premature termination, must be reported to the sponsor. An evaluation after the birth of the child will also be conducted.

9.1.15 ECG Procedure

A standard 12-lead ECG will be recorded. The investigator (or a qualified observer at the study site) will interpret the ECG using 1 of the following categories: within normal limits, abnormal but not clinically significant, or abnormal and clinically significant.

9.1.16 PML Evaluation

PML will be evaluated based on the PML checklists (Appendix D), which will be performed before administration of the study drug if it is the day of study drug administration.

At first, the subjective symptoms will be checked by use of the Subjective PML checklist. When any subjective symptoms are recognized, the corresponding objective findings will be checked by use of the Objective PML checklist. When any abnormalities are found in the objective findings as well, the subject will be evaluated in accordance with the algorithm for subject evaluation in the PML checklists.

The evaluation result based on the PML checklists will be recorded in the (e)CRF. Any subjective symptoms or objective findings found in the PML checklists will be collected as AEs as well.

9.1.17 Tuberculosis test

As a sample for tuberculosis test, 7 mL of the whole blood will be collected. The laboratory site for the study will collect the sample to conduct tuberculosis test by means of T-SPOT. The tuberculosis test using QuantiFERON may be conducted, when routinely employed in the study site. The test results, i.e. positive, negative, indeterminate or indeterminable, will be recorded in the (e)CRF.

9.1.18 Chest Imaging Procedure

Existence of findings of tuberculosis will be confirmed by the chest X-ray or chest CT. The imaging performed within 3 months before evaluation as a part of general practice not as a test for the study may be used, if available. Date of Examination and existence of findings of tuberculosis will be recorded in the (e)CRF.

Of note, the imaging obtained before acquisition of the informed consent may be used at screening.

9.1.19 Pharmacogenomic Sample Collection

For exploratory research to identify a predictable marker of drug response, 5 mL of the whole blood will be once collected as a pharmacogenomic sample from subjects entered into the induction phase and who agreed with the pharmacogenomic research at the earliest possible time during the period from Visit 2 to the end of the study.

However, the pharmacogenomic samples will not be collected from subjects with a history of an allogeneic bone marrow transplantation.

See the separately specified written procedure for collecting, handling, and storing the pharmacogenomic samples.

9.1.20 Pharmacokinetic Sample Collection and Analysis

Pharmacokinetic samples will be collected only in study sites with a cooled centrifuge. In these study sites, 3 mL of the venous blood will be collected for determination of serum MLN0002 concentration. At the visit for study drug administration, the blood should be sampled immediately before administration, where possible. The date and time of the blood sampling will be recorded in the (e)CRF.

The collected blood will be left standing at room temperature for approximately 30 minutes and centrifuged at 3,000 rpm at 4°C for 10 minutes. The obtained serum will be cryopreserved in a preset freezer at a temperature of ≤-20°C until collection. The samples will be handled in accordance with the separately specified procedures.

Serum MLN0002 concentration will be determined in PPD by means of Enzyme-Linked ImmunoSorbent Assay (ELISA). However, samples of subject who have not received MLN0002 throughout the induction and maintenance phases will not be determined.

9.1.21 Sample Collection for the HAHA/Neutralizing Antibody Test

Samples for the HAHA/neutralizing antibody test will be collected only in study sites with a cooled centrifuge. In these study sites, 5 mL of the venous blood will be collected for the HAHA/neutralizing antibody test. At the visit for study drug administration, the blood should be sampled immediately before administration, where possible. The date and time of the blood sampling will be recorded in the (e)CRF.

The collected blood will be left standing at room temperature for approximately 30 minutes and centrifuged at 3,000 rpm at 4°C for 10 minutes. The obtained serum will be cryopreserved in a preset freezer at a temperature of ≤-20°C until collection. The samples will be handled in accordance with the separately specified procedures.

Serum concentration of HAHA will be determined in PPD [redacted] by means of ELISA. However, samples of subjects who have not received MLN0002 throughout the study will not be determined.

Furthermore, the serum neutralizing antibody will be determined only for the HAHA-positive samples. The serum neutralizing antibody will be determined in PPD [redacted] by means of the Cell Based Assay.

9.1.22 Record of Subjects Who Withdrew during the Screening Phase

The (e)CRFs will be documented for all subjects who signed the informed consent and withdrew during the screening phase. The primary reason for screen failure is recorded in the (e)CRF by the following categories:

- Pretreatment Event/AE
- Did not meet inclusion criteria or met exclusion criteria <specify the reason>
- Major protocol deviation
- Lost to follow-up
- Voluntary withdrawal <specify the reason>
- Study termination
- Pregnancy
- Other <specify the reason>

The Subject Identification Codes applied to subjects who withdrew during the screening phase will not be reused.

9.1.23 Enrollment into the induction Phase

Only subjects who meet all of the inclusion criteria and none of the exclusion criteria are eligible for enrollment into the induction phase at Week 0 (Visit 2). At enrollment into the induction phase, the following information will be registered on the website of the registration center where the study drug number to be dosed at Week 0 (Visit 2) will be provided.

- Treatment history with anti-TNFα antibodies
- Concomitant use of immunomodulators (AZA or 6-MP)
- Concomitant use of corticosteroids

- The subject diary data and subscores of findings on endoscopy and physician's global assessment for calculation of the complete Mayo score at Week 0 (Visit 2)

9.1.24 Registration of the Results of the Primary Efficacy Endpoint in the Induction Phase/Enrollment into the Maintenance Phase

The subject diary data and subscores of findings on endoscopy and physician's global assessment for calculation of the complete Mayo score at Week 10 (Visit 5) will be registered on the website of the registration center, by which adjudication results for clinical response/no-response may be obtained. Only responders at Week 10 will be enrolled into the maintenance phase at Week 14 (Visit 6). At enrollment into the maintenance phase, the study drug number to be dosed at Week 14 (Visit 6) will be obtained on to the website of the registration center.

See Section 9.1.25 for non-responders at Week 10.

9.1.25 Enrollment into the Open-label Cohort

Non-responders at Week 10 may be enrolled into the open-label cohort at Week 10 (Visit 5). In addition, either subjects who experienced disease worsening or received rescue treatments during the maintenance phase, or completed Week 60 of the maintenance phase may be enrolled into the open-label cohort. However, subjects who withdrew before the examination at Week 10 (Visit 5), or subjects who respond at Week 10 and withdrew before enrollment into the maintenance phase at Week 14 (Visit 6) are not allowed to be enrolled into the open-label cohort.

The procedures for enrollment into the open-label cohort is provided below;

- Non-responders at Week 10:
  Non-responders at Week 10 will be judged whether to be enrolled into the open-label cohort after completion of examination at Week 10 (Visit 5). For enrolled into the open-label cohort, the study drug number to be dosed at Week 0x (Visit 1x) will be obtained on the website of the registration center.
- Subject experienced disease worsening or received rescue treatments during the maintenance phase:
  Subjects who experienced disease worsening or received rescue treatments during the maintenance phase will be judged whether to be enrolled into open-label phase after completion of the evaluation at withdrawal of the maintenance phase. However, these subjects will be enrolled into open-label phase 3 to 9 weeks after the last dose in the maintenance phase. For enrollment into the open-label cohort, the study drug number to be dosed at Week 0x (Visit 1x) will be obtained on the website of the registration center.
- Subjects completed Week 60:
  Subjects who completed Week 60 will be judged whether to be enrolled into the open-label cohort after completion of examination at Week 60 (Visit 18). For enrollment into the open-label cohort, the study drug number to be dosed at Week 0x (Visit 1x) will be obtained on the website of the registration center.

9.2 Study Drug Compliance

The study drugs dosed to the subjects will be recorded in the (e)CRF for the dates, times of start/end of the infusion. When infusion of a study drug was not completed, dosage and reasons for dose incomplete will be recorded in the (e)CRF.

9.3 Schedule of Observations and Procedures

The schedule for all study-related procedures for all evaluations is shown in Appendix A. Assessments should be completed at the designated visit/time point(s).

9.3.1 The Screening Phase (Visit 1)

Physical examination and tests are conducted 21 to 3 days before the first dose of the study drug. Subjects will be screened in accordance with predefined inclusion and exclusion criteria as described in Section 7.0. See Section 9.1.22 for procedures for documenting screening failures.

Procedures to be completed at Screening Phase (Visit 1) include:

- Informed consent
- Demographic data, body height, medical history, pretreatment drugs, and complications
- Physical examination, vital signs
- Body weight
- ECG
- Chest Imaging Procedure*
- Tuberculosis test
- PML checklists
- Laboratory tests (see Appendix A for test items)
- Partial Mayo score
- Concomitant drugs and treatments
- PTEs assessment

  * Not required when a chest image performed within 3 months is available (the image taken outside the study and/or prior to the informed consent date is acceptable).

9.3.2 Induction Phase

9.3.2.1 Baseline Evaluation/Enrollment into the induction Phase (Visit 2)

Subjects will be screened in accordance with predefined inclusion and exclusion criteria as described in Section 7.0. Subjects who meet all of the inclusion criteria and none of the exclusion

criteria are eligible for enrollment into the induction phase at Week 0 (Visit 2) as per Section 9.1.23. See Section 9.1.22 for procedures for documenting subjects not eligible for enrollment into the induction phase.

Tests/observations/assessments to be performed at Week 0 (Visit 2) include:

- Physical examination, vital signs
- Body weight
- ECG
- PML checklists
- Laboratory tests (see Appendix A for test items)
- Complete Mayo score (including an endoscopy of the rectum and sigmoid colon. See Section 9.1.9 for details)
- Check of the patient diary
- IBDQ
- Blood sampling for HAHA /neutralizing antibody test
- Blood sampling for pharmacogenomics (in subjects providing agreements)
- Concomitant drugs and treatments
- Evaluation of Pretreatment Event/AE
- Issuance of study drug numbers/administration

9.3.2.2 Administration in the Induction Phase (Visits 3 and 4)

Procedures to be completed at Week 2 (Visit 3) and Week 6 (Visit 4) include:

- Physical examination, vital signs
- Body weight
- PML checklists
- Laboratory tests (see Appendix A for test items)
- Partial Mayo score
- Check of the patient diary
- UC-related events
- Blood sampling for serum drug concentration
- Concomitant drugs and treatments

- Evaluation of AEs
- Issuance of study drug numbers/administration

9.3.2.3 Evaluation of Primary Efficacy Endpoint (Visit 5)/Withdrawal during the Induction Phase

Procedures to be completed at Week 10 (Visit 5) include: The same procedures will be completed at the earliest possible visit for subjects who withdrew in the course of the induction phase.

- Physical examination, vital signs
- Body weight
- ECG
- Laboratory tests (see Appendix A for test items)
- Complete Mayo score (including an endoscopy of the rectum and sigmoid colon. See Section 9.1.9 for details. The partial Mayo score not including endoscopy may be accepted for subjects who withdrew in the course of the induction phase)
- Check of the patient diary
- IBDQ
- UC-related events
- Blood sampling for HAHA /neutralizing antibody test
- Blood sampling for serum drug concentration
- Concomitant drugs and treatments
- Evaluation of AEs

An adjudication result for clinical response/not-response at Week 10 (Visit 5) will be obtained from the registration center. Responders will be enrolled into the maintenance phase at Week 14 (Visit 6). Non-responders may be enrolled into the open-label cohort at Week 10 (Visit 5).

9.3.3 Maintenance Phase

9.3.3.1 Enrollment into the maintenance Phase (Visit 6)

Responders at Week 10 (Visit 5) will be enrolled into the maintenance phase at Week 14 (Visit 6) in accordance with Section 9.1.24.

Procedures to be completed at Week 14 (Visit 6) include:

- Physical examination, vital signs
- Body weight

- PML checklists
- Laboratory tests (see Appendix A for test items)
- Partial Mayo score
- Check of the patient diary
- IBDQ
- UC-related events
- Blood sampling for serum drug concentration
- Concomitant drugs and treatments
- Evaluation of AEs
- Issuance of study drug numbers/administration

9.3.3.2 Administration in the Maintenance Phase (Visits 7 through 17)

Procedures to be completed at Week 18 (Visit 7) through Week 58 (Visit 17) include:

- Physical examination, vital signs
- Body weight
- PML checklists (only at Visits 8, 10, 12, 14 and 16)
- Laboratory tests (only at Visits 8, 10, 12, 14 and 16. See Appendix A for test items)
- Partial Mayo score
- Check of the patient diary
- IBDQ (only at Visit 12)
- UC-related events
- Blood sampling for HAHA/neutralizing antibody test (only at Visit 10)
- Blood sampling for serum drug concentration (only at Visits 8 and 10)
- Concomitant drugs and treatments
- Evaluation of AEs
- Issuance of study drug numbers/administration (only at Visits 8, 10, 12, 14 and 16)

9.3.3.3 Evaluation of Primary Efficacy Endpoint in the Maintenance Phase (Visit 18)/Withdrawal during the Maintenance Phase

Procedures to be completed at Week 60 (Visit 18) include: The same procedures will be completed at earliest possible visit for subjects who withdrew in the course of the maintenance phase.

- Physical examination, vital signs
- Body weight
- ECG
- Laboratory tests (see Appendix A for test items)
- Complete Mayo score (including an endoscopy of the rectum and sigmoid colon. See Section 9.1.9 for details. The partial Mayo score not including endoscopy may be accepted for subjects who withdrew in the course of the maintenance phase)
- Check of the patient diary
- IBDQ
- UC-related events
- Blood sampling for HAHA /neutralizing antibody test
- Blood sampling for serum drug concentration
- Concomitant drugs and treatments
- Evaluation of AEs

Subjects who completed the examination at Week 60 (Visit 18) may be enrolled into the open-label cohort at Week 60 (Visit 18). Furthermore, subjects who experienced disease worsening or received rescue treatments during the maintenance phase may be enrolled into the open-label cohort after completion of the evaluation at withdrawal of the maintenance phase.

9.3.4 Open-label Cohort

9.3.4.1 Enrollment into the open-label cohort (Visit 1x)

Subjects who have been decided to be enrolled into the open-label cohort as per Section 9.1.25 will be enrolled into the open-label cohort. Procedures to be completed at Week 0x (Visit 1x) include:

- PML checklists
- Concomitant drugs and treatments
- Evaluation of AEs
- Issuance of study drug numbers/administration

9.3.4.2 Administration in the Open-label Cohort (Visits 2x through 24x)

Procedures to be completed at Week 2x (Visit 2x) through Week 90x (Visit 24x) include:

- Physical examination, vital signs
- Body weight

- PML checklists (only at Visits 2x, 3x, 5x, 7x, 9x, 11x, 13x, 15x, 17x, 19x, 21x and 23x)
- Laboratory tests (only at Visits 2x, 3x, 5x, 7x, 9x, 11x, 13x, 15x, 17x, 19x, 21x and 23x. See Appendix A for test items)
- Partial Mayo score
- Check of the patient diary
- UC-related events
- Blood sampling for HAHA /neutralizing antibody test (only at Visits 4x, 9x and 17x)
- Concomitant drugs and treatments
- Evaluation of AEs
- Issuance of study drug numbers/administration (only at Visits 2x, 3x, 5x, 7x, 9x, 11x, 13x, 15x, 17x, 19x, 21x and 23x).

9.3.4.3 The Final Evaluation of the Open-label Cohort (VIST 25x)/Withdrawal during the Open-label Cohort

Procedures to be completed at Week 94x (Visit 25x) include: The same procedures will be completed at the earliest possible visit for subjects who withdrew in the course of the open-label cohort.

- Physical examination, vital signs
- Body weight
- ECG
- PML checklists
- Laboratory tests (see Appendix A for test items)
- Partial Mayo score
- Check of the patient diary
- UC-related events
- Blood sampling for HAHA /neutralizing antibody test
- Concomitant drugs and treatments
- Evaluation of AEs
- Issuance of study drug numbers/administration (not for subjects who withdrew from the open-label cohort)

9.3.5 The Unscheduled Visits

The following procedures will be preferably completed at the unscheduled visits of any subjects who received the study drug due to worsening of UC, etc., if any.

- Partial Mayo score
- Check of the patient diary
- UC-related events
- Concomitant drugs and treatments
- Evaluation of AEs

Besides the above, additional tests may be performed as needed.

The subject's applicability to the disease worsening criteria (see Section 3.1 for definition) will be judged in consideration of test results performed at the unscheduled visit as well. In such cases, the interval between visits will be not less than 1 week, where possible.

9.3.6 Predefined Study Completion

The same examination as those to be performed at Week 94x (Visit 25x) will be performed at study completion predefined at 46 weeks after the last subject enrollment into the open-label cohort.

The sponsor will inform the investigator of the scheduled study completion date well before the date.

Upon receipt of notice of the scheduled study completion date, the investigator will decide the termination visits for each subjects to undergo the same evaluation as those scheduled at Week 94x (Visit 25x).

9.3.7 At 16 weeks after the Last Dose

The following evaluation will be conducted at 16 weeks after the last dose of the study drug for all subjects who received the study drug. Even when the study will be terminated before 16 weeks have passed after the last dose of the study drug, the same evaluation will also be conducted in principle.

For all subjects who received the study drug, the investigator must complete the End of Study (e)CRF page.

- Physical examination, vital signs
- Body weight
- ECG
- Chest Imaging Procedure*

- PML checklists
- Laboratory tests (see Appendix A for test items)
- UC-related events
- Blood sampling for HAHA /neutralizing antibody test
- Concomitant drugs and treatments
- Evaluation of AEs

  * Not required when a chest image performed within 3 months is available (the image taken outside the study is acceptable).

9.3.8 Post Study Care

The study drugs will not be provided after the last dose.

9.4 Retention and Disposal of Biological Samples

The samples of 5 mL of the whole blood collected for Pharmacogenomic Research will be cryopreserved in PPD (Annex 1).

The retention period should be 20 years from the first sampling day for Pharmacogenomic Research in the study.

Upon subject's request of disposal of his/her samples during the retention period, the study site will request PPD to dispose of the samples via the sponsor as per the procedures. PPD will dispose of the samples in accordance with the procedures, which will be reported to the study site and the sponsor. However, the above is not applicable, when all documents (e.g., medical records) to be used for identification of subjects have been disposed of after study termination, so that any samples and subjects are impossible to be linked together.

Of note, even though the above linkage may be possible, when Pharmacogenomic Research has been conducted already, the sponsor may keep the anonymized results of the Pharmacogenomic Research with the residual samples disposed.

The sponsor will create a necessary written procedure as establishing a necessary control system for protecting subjects' personal information and predefining the standards for collection, retention and disposal of samples.

9.5 Follow-Up Survey

In the study, the follow-up survey will be conducted via telephone, etc., by using the follow-up survey form (Appendix E) every 6 months up to 2 years (maximum) after the last dose of the study drug or until the marketing approval date of the drug, whichever comes earlier, for any subjects who received the study drug (including subjects who withdrew). The follow-up survey include the following items. Of note, the follow-up survey forms will be collected by the sponsor.

The survey to be completed at 6, 12, 18 and 24 months[4] after the last dose of the study drug:

- Diagnoses of colon dysplasia, cancer, lymphoma or other types of cancer
- Diagnoses of PML
- Enterectomy

The survey to be completed only once at 6 months[4] after the last dose of the study drug:

- Diagnoses of infection requiring hospitalization
- Female subjects: pregnancy
- Male subjects: pregnancy of his female partner

[4]The window is ±1 month.

10.0 PRETREATMENT EVENTS AND ADVERSE EVENTS

10.1 Definitions

10.1.1 PTEs

A PTE is defined as any untoward medical occurrence in a subject who has signed the informed consent to participate in a study but prior to administration of any study drug; it does not necessarily have to have a causal relationship with study participation.

10.1.2 AEs

An AE is defined as any untoward medical occurrence in a study subject who received a drug (including a study drug); it does not necessarily have to have a causal relationship with this treatment.

An AE can therefore be any unfavorable and unintended sign (e.g., a clinically significant abnormal laboratory finding), symptom, or disease temporally associated with the use of a drug whether or not it is considered related to the drug.

10.1.3 Additional Points to Consider for PTEs and AEs

An untoward finding generally may include:

- A new diagnosis or those representing unexpected worsening of a pre-existing condition (intermittent events of pre-existing conditions of an underlying disease should not be considered PTEs or AEs.)

  Intermittent events of pre-existing conditions of an underlying disease in the study include some UC-specific symptoms such as diarrhea or abdominal pain. These symptoms, which may be sustained or varied in the course of the study depending on medical histories of each subject, will be considered as medically-predictable clinical symptoms in the disease addressed in the study, and therefore, not be subjected for collection of AEs. These symptoms corresponding to disease activity will be assessed as a part of the Mayo score. However, worsening of disease activity (e.g., abdominal pain aggravated beyond a normal range, or newly-observed symptoms or findings) will be collected as AEs. Onset or worsening of extra-intestinal manifestations in the course of the study will be collected as AEs.

- Those requiring therapeutic intervention
- Those requiring an invasive diagnostic procedure
- Those requiring discontinuation or dose modification of the study drug or a concomitant drug
- Those considered as unfavorable by the investigator for any reason.

Diagnoses vs signs and symptoms:
PTEs and AEs (hereinafter called as PTEs/AEs) will be described as diagnoses. Accompanying signs (including abnormal laboratory values or ECG findings) or symptoms should NOT be

recorded as PTEs/AEs. If a diagnosis is unknown, sign(s) or symptom(s) should be recorded appropriately as PTEs/AEs.

Laboratory values and ECG parameters:
Changes in laboratory values or ECG parameters are considered as PTEs/AEs only when they are judged to be clinically significant (i.e., when some action or intervention is required or if investigator judges the change as being beyond the range of normal physiologic fluctuation). A laboratory re-test and/or continued monitoring of an abnormal value are not considered an intervention. In addition, repeated or additional noninvasive testing for verification, evaluation or monitoring of an abnormality is not considered an intervention.

If abnormal laboratory values or ECG findings are the result of pathology for which there is an overall diagnosis (e.g., increased creatinine in renal failure), the diagnosis only should be reported appropriately as a PTE or as an AE.

Pre-existing conditions:
Any pre-existing diseases or conditions prior to acquisition of the informed consent will be recorded as complications not as PTEs/AEs. Baseline evaluations (e.g., laboratory tests, ECG, X-rays etc.) should NOT be recorded as PTEs unless related to study procedures. However, abnormal findings caused by the baseline evaluation (e.g., internal hemorrhage at blood sampling) are recorded as PTEs to be entered in the (e)CRF. Investigators should ensure that the event term recorded captures the change in the condition (e.g., “worsening of…”).

If a subject has a pre-existing episodic condition (e.g., asthma, epilepsy), any occurrence of an episode should be captured as a PTE/AE only when the episodes become more frequent, serious or severe in nature, that is, investigators should ensure that the AE term recorded captures the change in the condition from Baseline (e.g., “worsening of…”). If a subject has a degenerative concurrent condition (e.g., cataracts, rheumatoid arthritis), worsening of the condition should be captured as a PTE/AE only when occurring to a greater extent to that which would be expected. Again, investigators should ensure that the AE term recorded captures the change in the condition (e.g., “worsening of…”).

Worsening of PTEs/AEs:
If the subject experiences a worsening or complication of a PTE after starting administration of the study drug, the worsening or complication should be recorded appropriately as an AE. Investigators should ensure that the AE term recorded captures the change in the condition (e.g., “worsening of…”).

If the subject experiences a worsening or complication of an AE after any change in the study drug, the worsening or complication should be recorded as a new AE. Investigators should ensure that the AE term recorded captures the change in the condition (e.g., “worsening of…”).

Changes in severity of AEs/Serious PTEs:
If the subject experiences changes in severity of an AE/serious PTE, the event should be captured once with the maximum severity recorded.

Preplanned surgeries or procedures:
Preplanned procedures (surgeries or therapies) that were scheduled prior to the time of signing on the informed consent form are not considered PTEs/AEs. However, if a preplanned procedure is performed early (e.g., as an emergency) due to a worsening of the pre-existing condition, the worsening of the condition should be captured appropriately as a PTE/AE. Complications resulting from any planned surgery should be reported as AEs.

Elective surgeries or procedures:
Elective procedures performed where there is no change in the subject's medical condition should not be recorded as PTEs/AEs, but should be documented in the subject's source documents. Complications resulting from an elective surgery should be reported as AEs.

Insufficient clinical response (lack of efficacy):
Insufficient clinical response, efficacy, or pharmacologic action should NOT be recorded as an AE. Investigator must make the distinction between exacerbation of pre-existing illness and lack of therapeutic efficacy.

Overdose:
Cases of overdose with any drug are NOT considered AEs, but instead will be documented on an Overdose page of the (e)CRF. Any manifested side effects at overdose will be considered AEs and will be recorded on the AE page of the (e)CRF.

10.1.4 SAEs

SAEs are defined as the following out of any untoward medical occurrence in a study subject who received a drug (regardless of dosages);

1. Results in DEATH.
2. Is LIFE THREATENING*
3. Requires inpatient HOSPITALIZATION or prolongation of existing hospitalization.
4. Results in persistent or significant DISABILITY/INCAPACITY.
5. Leads to a CONGENITAL ANOMALY/BIRTH DEFECT.
6. Other medically significant conditions
   May require intervention to prevent the above items 1 through 5, or may expose the subject to danger, even though the event is not immediately life-threatening or fatal or does not result in hospitalization. That includes any events or synonyms described in the Takeda Medically Significant AE List (Table 10.a).

*The "life-threatening" here represents that life of the subject was threatened by onset of the event, and does not represent hypothetically that a more sever event might cause death.

Table 10.a Takeda Medically Significant AE List

Acute respiratory failure / acute respiratory distress syndrome	Hepatic necrosis
Torsade de pointes / ventricular fibrillation / ventricular tachycardia	Acute liver failure
Malignant hypertension	Anaphylactic shock
Convulsive seizures	Acute renal failure
Agranulocytosis	Pulmonary hypertension
Aplastic anemia	Pulmonary fibrosis
Toxic epidermal necrolysis/Stevens-Johnson syndrome	Neuroleptic malignant syndrome / malignant hyperthermia
	Spontaneous abortion / stillbirth and fetal death
	Confirmed or suspected transmission of infectious agent by a medicinal product
	Confirmed or suspected endotoxin shock

PTEs that fulfill 1 or more of the serious criteria above are also to be considered SAEs and should be reported and followed up in the same manner (see Sections 10.2.2 and 10.2.3).

10.1.5 Intensity of PTEs and AEs

Intensity of PTEs/AEs will be classified/defined as per the following.

Mild:	The event is transient and easily tolerated by the subject.
Moderate:	The event causes the subject discomfort and interrupts the subject’s usual activities.
Severe:	The event causes considerable interference with the subject’s usual activities.

10.1.6 Causality of AEs

Causal relationship between AEs and study drugs will be classified/defined as per the following.

Related:	An AE that follows a temporal sequence from administration of a drug (including the course after withdrawal of the drug), or for which possible involvement of the drug can be argued, although factors other than the drug, such as underlying diseases, complications, concomitant drugs, and concurrent treatments, may also be responsible.
Not Related:	An AE that does not follow a temporal sequence from administration of a drug and/or that can reasonably be explained by other factors, such as underlying diseases, complications, concomitant drugs, and concurrent treatments.

10.1.7 Relationship to Study Procedures

Relationship (causality) to study procedures should be determined for all PTEs/AEs. The relationship should be assessed as Yes if the investigator considers that there is reasonable possibility that an event is due to a study procedure. Otherwise, the relationship should be assessed as Not Related.

10.1.8 Start Dates

Onset dates of PTEs/AEs will be decided as per the following criteria;

PTEs/AEs	Onset Dates
Signs, symptoms or disease (diagnoses)	The date when the subject or the investigator was firstly aware of the sign/symptom.
Asymptomatic disease	The date of the definitive diagnosis on or after the diagnostic examination, even when any obsolete findings or other estimated onset time was found in the examination.
Worsening of complications or PTEs	The date when the subject or the investigator was firstly aware of the worsening of the disease or symptoms.
Newly-indicated abnormal laboratory values in values shown to be normal in the baseline test after acquisition of the informed consent (PTEs)<br>Newly-indicated abnormal laboratory values after the first dose of the study drug (AEs)	The date of the examination in which the clinically relevant abnormality was found.
Worsening of abnormal laboratory values found in the baseline test after acquisition of the informed consent (PTEs)<br>Worsening of abnormal laboratory values found at the first dose of the study drug (AEs)	The date of the examination in which the medically-judged obvious increase/decrease/elevation/reduction was found by change of the test results.

10.1.9 Stop Date

The stop date is defined as the date when the PTE/AE was recovered/resolved (including recovered/resolved with sequelae), or the date of death when caused by the PTE/AE. PTEs/AEs, which may not be confirmed for recovery/resolution at the study termination, will be judged as ongoing.

10.1.10 Frequency

Episodic AEs/serious PTE (e.g., vomiting) or those which occur repeatedly over a period of consecutive days are “intermittent.” All other events will be recorded as “ongoing.”

10.1.11 Action Concerning Study Drug

Action concerning study drug will be classified/defined as per the following.

Drug withdrawn	A study drug is stopped due to the particular AE.
Dose not changed	When the dose was not changed after the AE, when the dose discontinuation/reduction/increase was due to other AEs than the AE, or when the dose suspension/reduction/etc., was due to other reason (e.g., carelessness of the subject) than AEs.
Unknown	Only to be used if it has not been possible to determine what action has been taken.

Not applicable	A study drug was stopped for a reason other than the particular AE eg, the study has been terminated, the subject died, dosing with study drug was already stopped before the onset of the AE.

10.1.12 Outcome

Outcome of PTEs/AEs will be classified/defined as per the following.

Classification	Criteria for judgment
Recovered/Resolved	− Recovery or resolution of the symptom/finding<br>− Recovery to the normal value, the value before the first dose (for AEs), or the value at baseline after acquisition of the informed consent (for PTEs).
Recovering/Resolving	− The severity is lowered by one or more degree(s)<br>− The diagnosis or signs/symptoms has almost disappeared<br>− The abnormal laboratory value improved, but has not returned to the normal range or to baseline.<br>− The subject died from a cause other than the particular AE/serious PTE with the condition remaining “recovering/resolving.”
Not recovered/<br>not resolved	− There is no change in the diagnosis, signs or symptoms;<br>− The intensity of the diagnosis, signs/ symptoms or laboratory value on the last day of the observed study period has got worse than when it started;<br>− Irreversible congenital anomaly<br>− The subject died from another cause with the particular AE/serious PTE state remaining “Not recovered/not resolved.”
Resolved with sequelae	− **-** Resulting in dysfunction obstructing daily activity
Fatal	− The PTE/AE are considered to have a direct relationship with the death.<br>− The “direct relationship” here represents that the PTE/AE was the cause of the death, or clearly contributed to the death.<br>− Outcome should not be recorded as death for the PTE/AE not adjudicated (judged/estimated) as the direct cause of death in the same subject.<br>− The date of death will be recorded for fatal outcomes.
Unknown	− The course of the AE/serious PTE cannot be followed up due to hospital change or residence change at the end of the subject’s participation in the study.

10.2 Procedures

10.2.1 Collection and Reporting of AEs

10.2.1.1 PTE and AE Collection Period

PTEs will be continuously collected during the period from acquisition of the informed consent to the first dose of the study drug (Visit 2), or until withdrawal when decided before the first dose of the study drug.

AEs will be continuously collected during the period from the first dose of the study drug (Visit 2) to the end-of-study examination at 16 weeks after the last dose of the study drug.

10.2.1.2 PTE and AE Reporting

Subjects may report PTEs/AEs occurring at any other time during the study. PTEs/AEs not emerging at visit will be obtained from subjects with questions such as “How was your condition after the last visit?.”

Investigators will follow all subjects who developed a serious PTE until recovery/resolution of the event or recovery of the clinically relevant abnormal test value to the baseline value after acquisition of the informed consent, or otherwise (for persistent/irreversible PTEs) until establishment of sufficient rationale for the observed change. Subjects who developed not-serious PTEs are not required to be followed irrespective of relationship with study procedures in the protocol.

Investigators will follow all subjects who developed an AE (with or without relationship with the study drug) until recovery/resolution of the event or recovery of the clinically relevant abnormal test value to the value before the first dose of the study drug, or otherwise (for persistent/irreversible AEs) until establishment of sufficient rationale for the observed change. All PTEs/AEs should be recorded in the (e)CRF. For a PTE/AE, the name, onset date, recovery/resolution date, start period, frequency, severity, causal relationship with the study drug (related/not-related), disposition of the study drug, outcome, causal relationship with the study procedures (if related, the possible causal procedure), and seriousness will be recorded.

For AEs that occurred during infusion of the study drug or the period from initiation to 1 hour after completion of infusion, onset time and association with infusion will be recorded in the (e)CRF.

AEs/serious PTEs will be followed until the PTE/AE is recovered/resolved or the investigator judges not to follow them any longer.

10.2.2 Collection and Reporting of SAEs

When a SAE occurs during the AE collection period, it should be reported according to the following procedure: PTEs that fulfill 1 or more of the serious criteria described in Section 10.1.4 are also considered as SAEs to be reported and followed in the same manner.

Investigators should report the SAE within 1 working day after onset of the SAE or receipt of the notice of the event onset from a subject, etc., to the sponsor (see the Annex for contacts). Additionally, the investigator should submit the detailed formal reports within 10 calendar days.

Of note, the following is specified as the essential items to be reported within 1 working day, however, other items should be provided as much as possible.

- A short description of the event and the reason why the event is categorized as serious.
- Subject Identification Code
- Investigator’s name.

- Name of the study drugs
- Causality assessment

Any SAE spontaneously reported to the investigator following the AE collection period (including the follow-up survey period) should be reported to the sponsor.

10.2.3 Drug-induced Liver Function Test Abnormal Possibly Leading to Sever Liver Disorder

If a subject is noted to have ALT or AST >3 ×ULN and total bilirubin >2 ×ULN for which an alternative etiology has not been identified, the event should be recorded as an SAE and reported as per Section 10.2.2. The investigator must contact the Medical Monitor for discussion of the relevant subject details and possible alternative etiologies, such as acute viral hepatitis A or B or other acute liver disease. Follow-up laboratory tests as described in Section 9.1.12 must also be performed. These abnormal liver function test values, when judged not to be explained by any other factors, should be reported as SAEs (see Section 10.2.2).

10.3 Follow-up of SAEs

For information obtained after submission of the detailed report, the investigator should describe them in a copy of the submitted SAE Report Form or as an independent document in order to be submitted to the contact listed in the Annex. Copies of any relevant data from the hospital notes (e.g., ECGs, laboratory tests, discharge summary, postmortem results) should be sent to the addressee, if requested.

All SAEs should be followed up until resolution or permanent outcome of the event.

10.3.1 SAE Reports to Investigators, the IRBs and Regulatory Authorities

The sponsor should report any suspected unexpected serious adverse reactions (SUSARs) and other SAEs subjected to the expedited report to regulatory authorities, investigators and the heads of the study sites in accordance with the laws/regulations in the countries where the study is conducted. Relative to the first awareness of the event by/or further provision to the sponsor or its designee, SUSARs will be submitted to regulatory authorities within 7 days for fatal and life-threatening events and 15 days for other serious events. Similarly, the sponsor will submit expedited reports for other significant safety information considered to substantially affect benefit/risk of the study drug, or continuation of treatment or the study itself. The copies of these expedited report-related documents will be submitted to the IRBs in the study sites.

11.0 STUDY-SPECIFIC COMMITTEES

11.1 Independent Adjudication Committee for PML

In the study, the Independent Adjudication Committee for PML established to evaluate the PML risk will be utilized. When any PML risk, which may not be ruled out in a neurologist's examination in accordance with the PML checklists in the Appendix D, will be adjudicated in the Independent Adjudication Committee for PML based on the subject data provided. Detailed procedures should be in accordance with the separately specified written procedure.

11.2 Clinical Endpoint Committee

The Clinical Endpoint Committee (CEC) will comprise independent experts with experience and training appropriate for reviews of the clinical endpoints. The members will check all results of endoscopy reported by the study sites for adjudication of endoscopic scores as one of the endpoints, which will be reported to the sponsor. The CEC's assessment of each potential endpoint will be documented in the clinical database and will be used in the endpoint analysis. The purpose and responsibility will be included in the written procedures for the CEC.

12.0 DATA HANDLING AND RECORDKEEPING

The full details of procedures for data handling will be documented in the Data Management Plan. AEs, PTEs, medical history, and concurrent conditions will be coded using the Medical Dictionary for Regulatory Activities (MedDRA). Drugs will be coded using the World Health Organization (WHO) Drug Dictionary and Japanese Drug Dictionary.

12.1 CRFs (Electronic and Paper)

Completed (e)CRFs are required for each subject signed the informed consent.

The sponsor or the its designee will provide the study sites with access authority to the eCRF. The sponsor will make arrangements to train appropriate site staff in the use of the eCRF. These forms are used to transmit the information collected in the performance of this study to the sponsor and regulatory authorities. Data will be entered directly to the (e)CRF in Japanese.

Reasons for significant corrections should additionally be included. All new additions are to be made with the date and signature or seal affixed.

The principal investigator will verify accuracy and completeness of the records in the (e)CRF and electrically sign on the applicable pages. Furthermore, the investigator must retain full responsibility for the accuracy and authenticity of all data entered on the (e)CRFs.

The following data will not be recorded directly into the (e)CRFs;

1) Laboratory test values (except urine pregnancy test)
2) Serum concentrations of MLN0002
3) HAHA assessment
4) Neutralizing antibody assessment
5) Complete Mayo score and partial Mayo score (including each subscore)
6) Adjudication of clinical response/no-response at Week 10 (Visit 5)
7) Items of follow-up survey
8) Adjudication of endoscopies by the CEC

After the database lock of clinical study database, any change of, modification of or addition to the data on the (e)CRFs should be made by the investigator with use of change and modification records of (e)CRFs (Data Clarification Form) provided by the sponsor. The principal investigator must review the Data Clarification Form for completeness and accuracy, and must sign, or sign and seal, and date the form.

The (e)CRFs will be reviewed for completeness and acceptability at the study site during periodic visits by study monitors. The sponsor or its designee will be permitted to review the subject's medical and hospital records pertinent to the study to ensure accuracy of the (e)CRFs. The completed (e)CRFs are the sole property of the sponsor and should not be made available in any

form to third parties, except for authorized representatives of appropriate governmental health or regulatory authorities, without written permission of the sponsor.

12.2 Record Retention

Investigators or the heads of the study sites should retain the following documents in addition to records clearly specified in Section 12.1 and the study-specific documents for inspections or audits by regulatory authorities and the sponsor or its designee. These documents include the subject list for screening, medical records, worksheets for the study, the original informed consents with the signatures/dates, electronic copies of the (e)CRFs including tracking of audit certifications, and the drug control records. In addition, investigators and the heads of the study site should retain the retentive essential documents until the following 1) or 2), whichever comes later. However, when the sponsor requires a longer retention period than the above, the retention period and manner will be discussed among the sponsor and the heads of the study sites.

1) The marketing approval day for the drug (when the study was terminated, for 3 years since receipt of the termination notice)
2) The same day after 3 years as the study termination or completion day

Furthermore, investigators and the heads of the study sites should retain the retentive essential documents until receipt of the notice stating that these documents should not be retained any more from the sponsor.

13.0 STATISTICAL METHODS

13.1 Statistical and Analytical Plans

Since the study has different objectives in the induction phase and maintenance phases/open-label cohort, analyses will be conducted separately among these.

The Statistician in the study will prepare and finalize the Statistical Analysis Plan prior to blind-breaking. The document will provide further details regarding the definition of analysis variables and analysis methodology to address all study objectives.

The double-blinded review should be conducted prior to blind-breaking. In the double-blinded review, accuracy and completeness of the study data, feasibility of subject evaluation, and acceptability of the planned analysis methodology will be evaluated.

The data for marketing application will be analyzed, which includes data of all subjects as fixed on the cut-off date for marketing application. Additionally, the open-label cohort will be analyzed separately at the time when the last subject completed the study.

13.1.1 Analysis Sets

In the study, 3 types of analysis sets will be used in the induction phase, i.e., the “Full Analysis Set of the induction phase,” “Per Protocol Set of the induction phase” and “Safety Analysis Set of the induction phase.”

The “Full Analysis Set of the induction phase,” a primary population for the efficacy analysis in the induction phase, is defined as “subjects who were randomized and received at least one dose of the study drug in the induction phase.”

Three types of analysis sets will be used in the maintenance phase, i.e., the “Full Analysis Set of the maintenance phase,” “Per Protocol Set of the maintenance phase” and “Safety Analysis Set of the maintenance phase.”

The “Full Analysis Set of the maintenance phase,” a primary population for the efficacy analysis in the maintenance phase, is defined as “subjects who were randomized and received at least one dose of the study drug in the maintenance phase.”

Two types of analysis sets will be used in the open-label cohort, i.e., the “Full Analysis Set in the open-label cohort” and “Safety Analysis Set in the open-label cohort.”

The “Safety Analysis Set in the open-label cohort” in the open-label cohort, a population for the safety analysis, is defined as “subjects who received at least one dose of the study drug in the open-label cohort.”

The detailed definitions of analysis sets are specified separately in the “Data-Handling Rules for Analyses.”

The sponsor will verify acceptability of definition of the analysis sets and the data-handling rules for analyses in consultation with medical specialists before database lock, if needed, and add matters not addressed in the plan for finalization as the "Data-Handling Rules for Analyses."

13.1.2 Analysis of Demographics and Other Baseline Characteristics

The major subject backgrounds will be calculated by each "cohort and treatment group in the induction phase" and in the consolidated "cohorts and treatment groups in the induction phase" in the "all subjects randomized in the induction phase" and "all subjects allocated in the Cohort 2 in the induction phase."

The major subject backgrounds will be calculated by each "treatment group in the maintenance phase" and in the consolidated "treatment groups in the maintenance phase" in the "all subjects randomized in the maintenance phase."

The major subject backgrounds will be calculated in the "all subjects enrolled into the open-label cohort."

The major subject backgrounds will be calculated in "all subjects who received the placebo in the induction phase and enrolled into the maintenance phase."

13.1.3 Efficacy Analysis

(1) Primary Endpoint and Analysis Methodology in the Induction Phase

[Primary endpoint]

Clinical response at Week 10

[Primary analysis]

The following descriptive calculations will be performed in the "Full Analysis Set in the induction phase."

"Clinical response at Week 10" (primary endpoint of the induction phase) will be calculated by treatment group of the induction phase. Also clinical response at Week 10 will be calculated with stratification according to a history of anti-TNFα antibody therapy and the comparison will be made with the Cochran-Mantel-Haenszel (CMH) test using a history of anti-TNFα antibody therapy as a stratification factor. Of note, the adjudication results in the study site will be used as the score of findings on endoscopy.

The results of the primary analysis of the induction phase will be interpreted as below to determine the efficacy of MLN0002 induction therapy.

- The superiority of MLN0002 over the placebo on clinical response at Week 10 in the primary analysis of the induction phase is demonstrated when a statistically significant difference is observed in the clinical response at Week 10.

[Secondary analysis]

For sensitivity point of view, robustness of the results will be confirmed in the same manner as those in the primary analysis described in Section 13.1.3 (1) in the “Per Protocol Set of the induction phase.” In addition, the score of findings on endoscopy based on adjudication of the CEC will be analyzed in the same manner as those in the primary analysis described in Section 13.1.3 (1) in the “Full Analysis Set of the induction phase.”

(2) Secondary Endpoints and Analysis methodologies in the Induction Phase

[Secondary endpoints]

Clinical remission at Week 10

Mucosal healing at Week 10

[Secondary analysis]

Clinical remission at Week 10 will be calculated in the same manner as those in the primary analysis described in Section 13.1.3 (1) in the “Full Analysis Set in the induction phase.” Of note, the adjudication results in the study site will be used as the score of findings on endoscopy.

The results of the secondary analysis will be interpreted as below to determine the efficacy of MLN0002 induction therapy.

- The superiority of MLN0002 over the placebo on clinical remission at Week 10 in the primary analysis of the induction phase is demonstrated when a statistically significant difference is observed in both the clinical response at Week 10 and clinical remission at Week 10.

[Other analyses]

Mucosal healing at Week 10 will be calculated in the same manner as those in the primary analysis described in Section 13.1.3 (1) in the “Full Analysis Set of the induction phase.” Of note, the adjudication results in the study site will be used as the score of findings on endoscopy.

(3) Exploratory Endpoints in Induction Phase

See Section 5.2.1.3.

(4) Primary Endpoint and Analysis Methodology in the Maintenance Phase

[Primary endpoint]

Clinical remission at Week 60

[Primary analysis]

The following descriptive calculations will be performed in the “Full Analysis Set in the maintenance phase.”

The “clinical remission at Week 60” (primary endpoint of the maintenance phase) will be calculated by treatment group in the maintenance phase. Also clinical remission at Week 60 will be calculated with stratification according to a history of anti-TNFα antibody therapy and the

comparison will be made with the Cochran-Mantel-Haenszel (CMH) test using a history of anti-TNFα antibody therapy as a stratification factor. Of note, the adjudication results in the study site will be used as the score of findings on endoscopy.

The results of the primary analysis of the maintenance phase will be interpreted as below to determine the efficacy of MLN0002 maintenance therapy.

- The superiority of MLN0002 over the placebo on clinical remission at Week 60 the primary analysis of the maintenance phase is demonstrated when a statistically significant difference is observed in the clinical remission at Week 60.

[Secondary analysis]

For sensitivity point of view, robustness of the results will be confirmed in the same manner as those in the primary analysis described in Section 13.1.3 (4) in the “Per Protocol Set of the maintenance phase.” In addition, the score of findings on endoscopy obtained from the CEC will be analyzed in the same manner as those in the primary analysis described in Section 13.1.3 (4) in the “Full Analysis Set of the maintenance phase.”

(5) Secondary Endpoints and Analysis Methodology in the Maintenance Phase

[Secondary endpoints]

Durable response

Mucosal healing at Week 60

Durable remission

Corticosteroid-free remission at Week 60

[Analysis methodology]

Durable response, mucosal healing at Week 60, durable remission and corticosteroid-free remission at Week 60 will be analyzed in the same manner as those in the primary analysis described in Section 13.1.3 (4) in the “Full Analysis Set in the maintenance phase.” Of note, the adjudication results in the study site will be used as the score of findings on endoscopy.

(6) Exploratory Endpoints in Maintenance Phase

See Section 5.2.2.3.

(7) Data Transformation and Missing Data

Details are specified in the “Data-Handling Rules for Analyses” and the “Statistical Analysis Plan.”

The efficacy endpoints of clinical response, clinical remission or mucosal healing will be considered as no-response, no-remission or no-mucosal healing, when adjudication for these endpoints are missing at the time of evaluation.

(8) Significance Level and Confidence Coefficient

- Significance level: 5% (2-sided test)
  Since the study has different objectives in the induction and maintenance phases, no adjustments for multiplicity will be made between the analyses of the two phases setting the significance level at 5% each.
- Confidence coefficient: 95% (2-sided estimate)

13.1.4 Pharmacokinetic Analysis

[Endpoints]

Serum concentrations of MLN0002

[Analysis methodology]

Summary statistics for serum MLN0002 concentrations at each evaluation in the induction phase will be calculated in the subjects who underwent proper determination of serum MLN0002 concentrations out of the "Full Analysis Set of the induction phase" by each cohort and in the consolidated cohorts in the induction phase, and subsequently figures showing transition of means/standard deviations will be plotted.

Summary statistics for serum MLN0002 concentrations at each evaluation in the maintenance phase will be calculated in the subjects who underwent proper determination of serum MLN0002 concentrations out of the "Full Analysis Set of the maintenance phase" by each treatment group in the maintenance phase, and subsequently figures showing transition of means/standard deviations will be plotted.

13.1.5 Safety Analysis

(1) AEs in the induction phase

AEs will be summarized in the "Safety Analysis Set in the induction phase."

Treatment-emergent AEs (TEAEs) in the induction phase are defined as AEs that emerged during the period from the first dose of the study drug in the induction phase through the first dose of the study drug in the maintenance phase or open-label cohort in the subjects enrolled into the maintenance phase or the open-label cohort, or defined as AEs that emerged since the first dose of the study drug in the induction phase in the subjects not enrolled into the maintenance phase or open-label cohort.

The following analyses will be conducted for TEAEs in the induction phase by each cohort or treatment group in the induction phase. The same analysis will be conducted in consolidation of the MLN0002 group in the Cohort 1 and Cohort 2. All TEAEs will be coded by use of MedDRA, and summarized based on the System Organ Class and Preferred Term.

- Calculation of overall TEAEs
- Calculation of "study drug-related" TEAEs

- Calculation of overall TEAEs by severity
- Calculation of "study drug-related" TEAEs by severity
- Calculation of TEAEs leading to "dose discontinuation" in disposition.
- Calculation of serious TEAEs
- Calculation of overall TEAEs by start period

(2) AEs in the maintenance phase

TEAEs in the maintenance phase are defined as AEs that emerged during the period from the first dose of the study drug in the maintenance phase through the first dose of the study drug in the open-label cohort in the subjects who enrolled into the open-label cohort, or defined as AEs emerged since the first dose of the study drug in the maintenance phase in the subjects not enrolled into the open-label cohort.

TEAEs in the maintenance phase will be analyzed in the same manner as those described in Section 13.1.5 (1) by each treatment group in the maintenance phase in the "Safety Analysis Set of the maintenance phase."

TEAEs in the maintenance phase will be analyzed in the same manner as those described in Section 13.1.5 (1) in "subject allocated to the placebo group in the induction phase and who received the placebo in the maintenance phase."

(3) AEs in the Open-label Cohort

TEAEs in the open-label cohort are defined as AEs emerged after the first dose of the study drug in the open-label cohort.

TEAEs in the open-label cohort will be analyzed in the same manner as those described in the Section 13.1.5 (1) in the "Safety Analysis Set of the open-label cohort."

(4) AEs Emerged after Treatment of MLN0002

TEAEs emerged after treatment of MLN0002 are defined as AEs emerged after the first dose of MLN0002.

TEAEs emerged after treatment of MLN0002 will be analyzed in the same manner as those described in Section 13.1.5 (1) in "subjects who received at least one dose of MLN0002."

(5) Laboratory tests, 12-Lead ECG, Vital Signs and Body Weights

The analysis sets and groups to be analyzed are the same as those described in (1), (2) and (3) in Section 13.1.5 in principle.

Summary statistics of measured values and difference between pre-/post-treatments will be calculated by each evaluation timing. Figures showing transition of each test value will be plotted.

Shift tables showing difference between pre-/post-treatments will be created.

13.1.6 Analysis of Immunogenicity Endpoints

[Endpoints]

HAHA, neutralizing antibody

[Analysis methodology]

HAHA /neutralizing antibody test results will be calculated by each cohort and in consolidated cohorts in the induction phase in the subjects who underwent proper HAHA /neutralizing antibody test out of the “Full Analysis Set of the induction phase.”

HAHA /neutralizing antibody test results will be calculated by each treatment group in the maintenance phase in the subjects who underwent a HAHA /neutralizing antibody test out of the “Full Analysis Set of the maintenance phase.”

HAHA /neutralizing antibody test results will be calculated in the subjects who underwent a HAHA /neutralizing antibody test out of the “Full Analysis Set of the open-label cohort.”

13.2 Interim Analysis and Criteria for Early Termination

The data for marketing application will be analyzed, which includes data of all subjects as fixed on the cut-off date for marketing application. Continuation/termination of the study will not be judged based on the analysis.

13.3 Determination of Sample Size

Subjects to be randomized in the induction phase: 246 subjects

Number of subjects to be evaluated in the induction phase (number of “subjects randomized and who received at least one dose of the study drug in the induction phase”)

MLN0002 group in the induction phase: 164 subjects

Placebo group in the induction phase: 82 subjects

Total: 246 subjects

Subjects to be enrolled into the maintenance phase: approximately 110 to 140 subjects

Number of subjects to be evaluated in the maintenance phase (number of “subjects randomized and who received at least one dose of the study drug in the maintenance phase”)

MLN0002 group in the maintenance phase: 42 to 55 subjects

Placebo group in the maintenance phase: 42 to 55 subjects

Total: 84 to 110 subjects

[Justification of the sample size]

On the basis of the findings from the phase 3 double-blinded, placebo-controlled study of MLN0002 in induction and maintenance therapy in non-Japanese subjects with moderate to severe

UC (C13006), the clinical response at Week 10 and clinical remission at Week 60 for MLN0002 and the placebo in this study was estimated to be 57.1% and 35.5%, and 43.3% and 15.9%, respectively.

When the clinical response at Week 10 in the induction phase is analyzed using Pearson's chi-square test with 2-sided significance level of 5%, 164 and 82 subjects (at a ratio of 2:1, total 246 subjects) will be required respectively for the MLN0002 and placebo group to detect significance with a power of at least 90%. These numbers were therefore selected as numbers of evaluable subjects of the primary endpoint of the induction phase. When the clinical remission at Week 10 in the induction phase is analyzed on these numbers of subjects, statistical significances in both the clinical response at Week 10 and clinical remission at Week 10 are detected with a power of 69%.

When the clinical remission at Week 60 in the maintenance phase is analyzed using Pearson's chi-square test with 2-sided significance level of 5%, 42 subjects each for the MLN0002 and placebo group (total 84 subjects) will be required to detect significance with a power of at least 80%. Similarly, 55 subjects each (total 110 subjects) will be required to detect significance with a power of at least 90%. These numbers were therefore selected as numbers of evaluable subjects of the primary endpoint of the maintenance phase. Subjects enrolled into the maintenance phase include “those assigned to the placebo group in the induction phase” to maintain the blindness in the induction phase. To detect significance with a power of 80 and 90% for the analysis of the primary endpoint of the maintenance phase, therefore, 110 and 140 subjects will be respectively necessary for subjects randomized in the maintenance phase.

14.0 QUALITY CONTROL AND QUALITY ASSURANCE

14.1 Study-Site Monitoring Visits

Monitoring visits to the study site will be made periodically during the study to ensure that all aspects of the protocol are followed. Source documents will be reviewed for verification of data recorded on the (e)CRFs. Source documents are defined as original documents, data, and records. The investigator and head of the study site should assure the sponsor (or its designee) or the IRB to review source documents.

The sponsor or its designee will review records including Investigator's files, drugs, subjects' medical records and informed consents to verify that the study is being conducted properly in compliance with the protocol. In addition, consistency will be verified between raw data and the (e)CRF. It is important that the investigator and other study personnel are available during the monitoring visits and that sufficient time is devoted to the process.

14.2 Protocol Deviations

Investigators may deviate from or modify the protocol without prior written agreement with the sponsor and prior approval from the IRB in medically inevitable occasions including any danger in subjects to be avoided urgently. After that, the investigator will give notice about details and reasons of the deviation or modification to the sponsor and the head of the study site in writing, retaining a copy, and discuss revisions of the protocol with the sponsor for agreement, if needed. If revision is needed, a draft of the revision will be submitted to the head of the study site at the earliest possible time to be approved by the IRB.

Investigators will record all deviations from the protocol.

14.3 Quality Assurance Audits and Regulatory Agency Inspections

The study site also may be subject to quality assurance audits by the sponsor or designees. In this circumstance, the sponsor-designated auditor will contact the site in advance to arrange an auditing visit. The auditor may ask to visit the facilities where laboratory samples are collected, where the drug is stored and prepared, and any other facility used during the study. In addition, there is the possibility that this study may be inspected by regulatory agencies, including those of foreign governments (e.g., the FDA, the United Kingdom Medicines and Healthcare products Regulatory Agency). If the study site is contacted for an inspection by a regulatory body, the sponsor should be notified immediately. The Investigator and the head of institution guarantee access for quality assurance auditors to all study documents as described in Section 14.1.

15.0 ETHICAL ASPECTS OF THE STUDY

This study will be conducted with the highest respect for the individual participants (i.e., subjects) according to the protocol, the ethical principles that have their origin in the Declaration of Helsinki, and the ICH Harmonised Tripartite Guideline for GCP. Each Investigator will conduct the study according to applicable local or regional regulatory requirements and align his or her conduct in accordance with the "Responsibilities of the Investigator" that are listed in Appendix B.

15.1 IRB Approval

IRBs will be established in accordance with local laws/regulations in the area where the study will be conducted. The sponsor or its designee will obtain a document describing names and titles of members of the IRB If any member of the IRB directly participates in this study, written notification regarding his or her abstinence from voting must also be obtained.

The sponsor or designee will supply relevant documents for submission to the respective IRB for the protocol's review and approval. This protocol, Investigator's Brochure, a copy of the informed consent form, and, if applicable, subject recruitment materials and/or advertisements and other documents required by all applicable laws and regulations, must be submitted to a central or local IRB for approval. The sponsor or its designee will obtain an approval document for the protocol and the informed consent form the IRB prior to the first dose of the study (i.e., before delivery of sponsor-supplied drugs). The IRB approval must refer to the study by exact protocol title, number, and version date; identify versions of other documents (e.g., informed consent form) reviewed; and state the approval date. The drugs will be dispatched after sponsor's validation of regulatory documents in the study site. Any procedures in the screening phase described in the protocol should not be started until the study site receives the drugs.

The study site should comply with all requirements specified by the IRB. This may include notification to the IRB regarding protocol amendments, updates to the informed consent form, recruitment materials intended for viewing by subjects, local safety reporting requirements, reports and updates regarding the ongoing review of the study at intervals specified by the respective IRB, and submission of the Investigator's final status report to the IRB. All IRB approvals and relevant documentation for these items must be provided to the sponsor or its designee.

Subject incentives should not exert undue influence for participation. Subject incentives should be approved by the IRB and the sponsor.

Regarding pharmacogenomic investigation using collected and stored specimens, analysis will be carried out at the time when detail is determined. The sponsor will create a research protocol for pharmacogenomics investigations and a research protocol will require prior approval of the company IRB in Japan.

15.2 Subject Information, Informed Consent, and Subject Authorization

Written consent documents will embody the elements of informed consent as described in the Declaration of Helsinki and the ICH Guidelines for GCP and will be in accordance with all

applicable laws and regulations. Written consent documents describe availability and disclosure (to any third-parties inside/outside Japan) of subjects' personal information and personal medical information in the study. The informed consent includes detailed explanation pertaining to primary intention, purpose, and possible risks/benefits. The informed consent form will detail the requirements of the participant and the fact that he or she is free to withdraw at any time without giving a reason and without prejudice to his or her further medical care.

Investigators have responsibility for preparation, contents and approval to be obtained from the IRB for the informed consent. The informed consent should be approved by the IRB prior to being effective.

The informed consent should be described in understandable wording for subjects. Investigators must explain the contents of the informed consent thoroughly to the subjects. Information should be provided in a proper manner as approved by the IRB both orally or in writing, where possible. In the event the subject is not capable of rendering adequate written informed consent, then the subject's legally acceptable representative may provide such consent for the subject in accordance with applicable laws and regulations.

The subject, or the subject's legally acceptable representative, must be given ample opportunity to: (1) inquire about details of the study and (2) decide whether or not to participate in the study. The subject or the subject's legally acceptable representative, when he/she has decided to participate in the study, should enter the signature (or name/seal) and date on the informed consent prior to participation. Investigators should request the subject or the subject's legally acceptable representative to enter his/her formal (not nicknames, etc.) signature or name/seal by use of a black or blue ballpoint pen. Investigators should enter the signature (or name/seal) and date on the informed consent prior to the subject's participation in the study, as well.

Investigators should retain originals of the informed consent with signatures or names/seals. Investigators should record the date of acquisition of the subject's signature or name/seal on the informed consent in the subject's medical record. Additionally, a copy of the informed consent with the signatures or names/seals will be provided to the subject.

All revised informed consent forms must be reviewed and signed by relevant subjects or the relevant subject's legally acceptable representative in the same manner as the original informed consent. The date the revised consent was obtained should be recorded in the subject's medical record, and the subject should receive a copy of the revised informed consent form.

The Pharmacogenomic Research will be explained based on "the informed consent for the Pharmacogenomic Research in the study for MLN0002" to the subjects who were given an explanation by use of the informed consent of the study. Sampling for Pharmacogenomic Research will be performed in the subjects who gave both informed consents for the study and the pharmacogenomic investigation.

When a subject requests disposal of his/her retained samples, it will be implemented according to Section 9.4.

15.3 Subject Confidentiality

The sponsor and designees affirm and uphold the principle of the subject's right to protection against invasion of privacy. Throughout this study, a subject's source data will only be linked to the sponsor's clinical study database or documentation via a unique identification number. As permitted by all applicable laws and regulations, limited subject attributes, such as sex, age, or date of birth may be used to verify the subject and accuracy of the subject's unique identification number.

To comply with ICH Guidelines for GCP and to verify compliance with this protocol, the sponsor requires the investigator to permit its monitor or designee's monitor, representatives from any regulatory authority (e.g., FDA, Medicines and Healthcare products Regulatory Agency, Pharmaceuticals and Medical Devices Agency), the sponsor's designated auditors, and the appropriate IRBs to review the subject's original medical records (source data or documents), including, but not limited to, laboratory test result reports, ECG reports, admission and discharge summaries for hospital admissions occurring during a subject's study participation, and autopsy reports. Access to a subject's original medical records requires the specific authorization of the subject as part of the informed consent process (see Section 15.2).

Copies of any subject source documents that are provided to the sponsor must have certain personally identifiable information removed (i.e., subject name, address, and other identifier fields not collected on the subject's [e]CRF).

15.4 Publication, Disclosure, and Clinical Trial Registration Policy

15.4.1 Publication and Disclosure

The investigator is obliged to provide the sponsor with complete test results and all data derived by the investigator from the study. During the study, only the sponsor may make study information available to other study investigators or to regulatory agencies, except as required by law or regulation. Except as otherwise allowable in the clinical study site agreement, any public disclosure (including publicly accessible websites) related to the protocol or study results is the sole responsibility of the sponsor.

The sponsor may publish any data and information from the study (including data and information generated by Investigators) without the consent of investigators.

The investigator, when intends to publicly present information acquired in the course of the study at a specialized academic society, etc., should obtain prior written approval from the sponsor.

15.4.2 Clinical Trial Registration

In order to ensure that information on clinical trials reach the public in a timely manner and to comply with applicable law, regulation and guidance, Takeda will, at a minimum, register all clinical trials conducted in subjects that it sponsors anywhere in the world on ClinicalTrials.gov or other publicly accessible websites (including JAPIC-CTI) before trial initiation. Takeda contact

information, along with Investigator's city, state (for US investigators), country, and recruiting status will be registered and available for public viewing.

15.4.3 Clinical Trial Results Disclosure

Takeda will post the results of this clinical trial, regardless of outcome, on ClinicalTrials.gov or other publicly accessible websites (including JAPIC-CTI), as required by applicable laws and/or regulations.

15.5 Insurance and Compensation for Injury

Any health damage occurred in subjects participating in the study will be compensated according to the local regulation applied to the study site. The sponsor or its designee will insure subjects be compensated for health damage, when required in the area where the study is conducted.

Subjects suffering/suffered health damage will be compensated in accordance with the study contract. Investigators will contact the sponsor or its designee for any questions pertaining to compensation.

16.0 REFERENCES

[1] Diagnostic Criteria and Treatment Guideline for Ulcerative Colitis and Crohn's Disease. Research Group for Intractable Inflammatory Bowel Disease Designated as Specified Disease by the MHLW of Japan (the Group of Dr. Watanabe), the additional volume of Assigned Study Report in 2012.

[2] Soler D, Chapman T, Yang LL, Wyant T, Egan R, Fedyk ER. The binding specificity and selective antagonism of vedolizumab, an anti-alpha4beta7 integrin therapeutic antibody in development for inflammatory bowel diseases. J Pharmacol Exp Ther. 2009;330:864-75.

[3] Edula RG, Picco MF. An evidence-based review of natalizumab therapy in the management of Crohn's disease. Ther Clin Risk Manag. 2009;5:935-42.

[4] Tilg H, Kaser A. Vedolizumab, a humanized mAb against the α4β7 integrin for the potential treatment of ulcerative colitis and Crohn's disease. Curr Opin Investig Drugs. 2010;11:1295-304.

[5] Parikh A, Leach T, Wyant T, Scholz C, Sankoh S, Mould DR, et al. Vedolizumab for the treatment of active ulcerative colitis: A randomized controlled phase 2 dose-ranging study. Inflamm Bowel Dis. 2012;18:1470-9.

[6] Feagan BG, Greenberg GR, Wild G, Fedorak RN, Paré P, McDonald JW, et al. Treatment of ulcerative colitis with a humanized antibody to the alpha4beta7 integrin. N Engl J Med. 2005;352:2499-507.

[7] Feagan BG, Greenberg GR, Wild G, Fedorak RN, Paré P, McDonald JW, et al. Treatment of active Crohn's disease with MLN0002, a humanized antibody to the alpha4beta7 integrin. Clin Gastroenterol Hepatol. 2008;6:1370-7.

[8] Feagan BG, Rutgeerts P, Sands BE, Hanauer S, Colombel JF, Sandborn WJ, et al. Vedolizumab as induction and maintenance therapy for ulcerative colitis. N Engl J Med. 2013;369(8):699-710.

[9] Sandborn WJ, Feagan BG, Rutgeerts P, Hanauer S, Colombel JF, Sands BE, et al. Vedolizumab as induction and maintenance therapy for Crohn's disease. N Engl J Med. 2013; 369(8):711-21.

[10] Sands B; Feagan B, Rutgeerts P, Colombel J; Sandborn W; Sy R, et al. Vedolizumab induction therapy for patients with Crohn’s disease and prior anti-TNF failure: a randomized, placebo-controlled, double-blind, multicenter trial. Presented at: Crohn’s & Colitis Foundation of America – Advances in Inflammatory Bowel Diseases Clinical and Research Conference. December 13-15, 2012. Hollywood, FL.

[11] Kobayashi K, Suzuki Y, Watanabe K, Mukae M, Yamada T, Yamagami H. The phase I, multicenter, open-label, dose-escalation, repeated-dose study for VDZ in Japanese UC patients. The 21st Japan Digestive Disease Week, Tokyo, 2013.

[12] Kappos L, Bates D, Edan G, Eraksoy M, Garcia-Merino A, Grigoriadis N, et al. Natalizumab treatment for multiple sclerosis: updated recommendations for patient selection and monitoring. Lancet Neurol. 2011;10:745-58.

[13] Hisabe T, Hirai F, Matsui T. Diagnosis of Ulcerative Colitis. J Jpn Soc Coloproctol. 2011;64(10):807-16.

Appendix A Schedule of Study Procedures

Screening, Induction Phase, Maintenance Phase

Study Procedures		Screening	Induction Phase				Maintenance Phase													16 weeks after final dose	Unscheduled visits[l]
	Week[a]		0	2	6	10/ET[j]	14	18	22	26	30	34	38	42	46	50	54	58	60/ET[k]		
	Day[a]	-21 to -3[i]	1	15	43	71/ET[j]	99	127	155	183	211	239	267	295	323	351	379	407	421/ET[k]		
	Visit Windows (Days)[a]	-	-	±3	±3	±3/ET[j]	±7	±7	±7	±7	±7	±7	±7	±7	±7	±7	±7	-7 to +6	±7/ET[k]	±14	
	VISIT	1	2	3	4	5/ET[j]	6	7	8	9	10	11	12	13	14	15	16	17	18/ET[k]	-	
Informed consent		X																			
Inclusion/exclusion criteria		X[m]	X																		
Demographics, body height, medical history, prior therapies, complications		X[m]																			
Randomization			X				X														
Issuance of study drug numbers/administration			X	X	X		X		X		X		X		X		X				
Physical examination, vital signs[b]		X[m]	X	X	X	X	X	X	X	X	X	X	X	X	X	X	X	X	X	X	
Body weight[b]		X[m]	X	X	X	X	X	X	X	X	X	X	X	X	X	X	X	X	X	X	
ECG[b]		X[m]	X			X													X	X	
Chest imaging procedure[c]		X[m]																		X	
Tuberculosis test		X[n]																			
PML checklists[b]		X[m]	X	X	X		X		X		X		X		X		X			X	
Laboratory tests[b]	Blood biochemistry	X[n]	X	X	X	X	X		X		X		X		X		X		X	X	
Laboratory tests[b]	Hematology	X[n]	X	X	X	X	X		X		X		X		X		X		X	X	
Laboratory tests[b]	Urinalysis	X[n]	X			X	X												X	X	
Laboratory tests[b]	Urine pregnancy test[d]	X[m]	X	X	X	X	X		X		X		X		X		X		X	X	
Laboratory tests[b]	HBs antigen, HCV antibody, HIV antigen/antibody tests	X[n]																			
Laboratory tests[b]	Inflammatory markers	X[n]	X	X	X	X	X		X		X		X		X		X		X		
Complete Mayo score[e]			X[o]			X[p]													X[p]		
Partial Mayo score		X[m]		X	X		X	X	X	X	X	X	X	X	X	X	X	X			X
Check of the patient diary			X	X	X	X	X	X	X	X	X	X	X	X	X	X	X	X	X		X
IBDQ[b]			X			X	X						X						X		
UC-related events				X	X	X	X	X	X	X	X	X	X	X	X	X	X	X	X	X	X
Blood sampling for HAHA/neutralizing antibody test[f]			X			X					X								X	X	
Blood sampling for PK[f]				X	X	X	X		X		X								X		
Blood sampling for PGx[f]			X																		
Concomitant drug/therapy		X[m]	X	X	X	X	X	X	X	X	X	X	X	X	X	X	X	X	X	X	X
PTEs/AEs[h]		X[m]	X	X	X	X	X	X	X	X	X	X	X	X	X	X	X	X	X	X	X

Footnotes are on last table page.

Appendix A Schedule of Study Procedures (continued)

Open-label Cohort

Study Procedures		Open-label Cohort													
	**Week[q]**	**0x**	**2x**	**6x**	**10x**	**14x**	**18x**	**22x**	**26x**	**30x**	**34x**	**38x**	**42x**	**46x**	**50x**
	**Day[q]**	**1x**	**15x**	**43x**	**71x**	**99x**	**127x**	**155x**	**183x**	**211x**	**239x**	**267x**	**295x**	**323x**	**351x**
	**Visit Windows (Days)[q]**	**-**	**±3**	**±3**	**±3**	**±7**	**±7**	**±7**	**±7**	**±7**	**±7**	**±7**	**±7**	**±7**	**±7**
	**VISIT[q]**	**1x**	**2x**	**3x**	**4x**	**5x**	**6x**	**7x**	**8x**	**9x**	**10x**	**11x**	**12x**	**13x**	**14x**
Informed consent															
Inclusion/exclusion criteria															
Demographics, body height, medical history, prior therapies, complications															
Randomization															
Issuance of study drug numbers/administration		X[s]	X	X		X		X		X		X		X	
Physical examination, vital signs[b]			X	X	X	X	X	X	X	X	X	X	X	X	X
Body weight[b]			X	X	X	X	X	X	X	X	X	X	X	X	X
ECG[b]															
Chest Imaging Procedure[c]															
Tuberculosis test															
PML checklists[b]		X[s]	X	X		X		X		X		X		X	
Laboratory tests[b]	Blood biochemistry		X	X		X		X		X		X		X	
	Hematology		X	X		X		X		X		X		X	
	Urinalysis														
	Urine pregnancy test[d]		X	X		X		X		X		X		X	
	HBs antigen, HCV antibody, HIV antigen/antibody tests														
	Inflammatory markers		X	X		X		X		X		X		X	
Complete Mayo score[e]															
Partial Mayo score			X	X	X	X	X	X	X	X	X	X	X	X	X
Check of the patient diary			X	X	X	X	X	X	X	X	X	X	X	X	X
IBDQ[b]															
UC-related events			X	X	X	X	X	X	X	X	X	X	X	X	X
Blood sampling for HAHA/neutralizing antibody test[f]					X					X					
Blood sampling for PK[f]															
Blood sampling for PGx[f]															
Concomitant drug/therapy		X	X	X	X	X	X	X	X	X	X	X	X	X	X
PTEs/AEs[h]		X	X	X	X	X	X	X	X	X	X	X	X	X	X

continued to the next page

Footnotes are on last table page.

Appendix A Schedule of Study Procedures (continued)

Open-label Cohort (continued)

Study Procedures		Open-label Cohort											16 weeks after final dose	Unscheduled visits[l]
	**Week[q]**	**54x**	**58x**	**62x**	**66x**	**70x**	**74x**	**78x**	**82x**	**86x**	**90x**	**94x/ET[r]**		
	**Day[q]**	**379x**	**407x**	**435x**	**463x**	**491x**	**519x**	**547x**	**575x**	**603x**	**631x**	**659x/ET[r]**		
	**Visit Windows (Days)[q]**	**±7**	**±7**	**±7**	**±7**	**±7**	**±7**	**±7**	**±7**	**±7**	**±7**	**±7/ET[r]**	**±14**	
	**VISIT[q]**	**15x**	**16x**	**17x**	**18x**	**19x**	**20x**	**21x**	**22x**	**23x**	**24x**	**25x/ET[r]**	**-**	
Informed consent														
Inclusion/exclusion criteria														
Demographics, body height, medical history, prior therapies, complications														
Randomization														
Issuance of study drug numbers/administration		X		X		X		X		X		X[t]		
Physical examination, vital signs[b]		X	X	X	X	X	X	X	X	X	X	X	X	
Body weight[b]		X	X	X	X	X	X	X	X	X	X	X	X	
ECG[b]												X	X	
Chest imaging procedure[c]													X	
Tuberculosis test														
PML checklists[b]		X		X		X		X		X		X	X	
Laboratory tests[b]	Blood biochemistry	X		X		X		X		X		X	X	
Laboratory tests[b]	Hematology	X		X		X		X		X		X	X	
Laboratory tests[b]	Urinalysis											X	X	
Laboratory tests[b]	Urine pregnancy test[d]	X		X		X		X		X		X	X	
Laboratory tests[b]	HBs antigen, HCV antibody HIV antigen/antibody tests													
Laboratory tests[b]	Inflammatory markers	X		X		X		X		X		X		
Complete Mayo score[e]														
Partial Mayo score		X	X	X	X	X	X	X	X	X	X	X		X
Check of the patient diary		X	X	X	X	X	X	X	X	X	X	X		X
IBDQ[b]														
UC-related events		X	X	X	X	X	X	X	X	X	X	X	X	X
Blood sampling for HAHA/neutralizing antibody test[f]				X								X	X	
Blood sampling for PK[f]														
Blood sampling for PGx[f]														
Concomitant drug/therapy		X	X	X	X	X	X	X	X	X	X	X	X	X
PTEs/AEs[h]		X	X	X	X	X	X	X	X	X	X	X	X	X

Footnotes are on the following page.

ET=early termination, IBDQ=Inflammatory Bowel Disease Questionnaire, HAHA=human anti-human antibody, PGx=pharmacogenomics, PK=pharmacokinetics, PTE=pretreatment event

a. The date of the first dose of the study drug in the induction phase is Week 0 (Day 1). The acceptable time windows are given as the number of days away from each scheduled visit.
b. Will be performed before administration of the study drug on the day of study drug administration.
c. Not required when a chest image performed within 3 months is available (the image taken before the informed consent date is acceptable at screening).
d. Will be performed only in women of childbearing potential.
e. Endoscopy for the assessment of total Mayo score will be performed within 9 days before day of the assessment of total Mayo score.
f. If the study drug is administrated in the visit, blood sample should be drawn immediately before study drug administration. If the study drug is not administrated in the visit, blood sample can be drawn at any time at visits. All blood sampling will be conducted only in study sites with a cooled centrifuge.
g. Will be collected only in subjects who have consented to participate in PGx research once at the earliest possible time between Visit 2 and study completion.
h. A PTE is an event that occurred before the first dose of the study drug. An AE is an event that occurred after the first dose of the study drug.
i. If subjects with a history of tuberculosis or those with suspicion of it start preventive isoniazid treatment before study enrollment, screening can be conducted between Day -28 and Day -3. Study drug administration must be conducted 21 or more days after isoniazid administration starts.
j. Subjects who will prematurely withdraw from the study in the induction phase after receiving at least one dose should visit the clinical site and have examinations for discontinuation as soon as possible.
k. Subjects who will prematurely withdraw from the study in the maintenance phase should visit the clinical site and have examinations for discontinuation as soon as possible. Eligible subjects will be enrolled into the open-label cohort 3 to 9 weeks after the last dose of the study drug in the maintenance phase.
l. Unscheduled visit will be made due to worsening of UC, etc. Additional tests will be performed as required.
m. Can be conducted between Day -21 and Day -1. If subjects with a history of tuberculosis or those with suspicion of it start preventive isoniazid treatment before study enrollment, screening can be conducted between Day -28 to Day -1. Study drug administration must be conducted 21 or more days after isoniazid administration starts.
n. The test results should be obtained by Visit 2.
o. Results of endoscopy performed within 9 days before evaluation (before the informed consent) can be used for the assessment if available.
p. Partial Mayo score can be acceptable at discontinuation of the study.
q. The date of the enrollment into the open-label cohort is Week 0x (Day 1x) and the study site visit on Day 1x is Visit 1x. The acceptable time windows are given as the number of days away from each scheduled visit.
r. Subjects who will prematurely withdraw from the study in the open-label cohort should visit the clinical site and have examinations for discontinuation as soon as possible. And the same examination will be conducted in the study termination scheduled 46 weeks after the last subject will be enrolled into the open-label cohort.
s. Will be performed after enrollment into the open-label cohort.
t. Will not be performed in subjects who prematurely withdraw from the study.

Appendix B Responsibilities of the Investigator

1. Execute the study properly as complying with the protocol and GCP in consideration of human rights, safety and welfare of subjects.
2. In order to assign some important study-related practices to subinvestigators or study collaborators, prepare lists of the assignments and persons in charge to be submitted to the head of the study site and obtain approval.
3. Prepare the informed consent form and revise it, when needed.
4. Verify the contents in the study contract.
5. Provide sufficient information about the protocol, drugs and individual assignments to subinvestigators and study collaborators, instruct and supervise them.
6. Select eligible subjects to the protocol, provide explanations to them in writing, and obtain the written informed consents from them.
7. Take responsibility for any study-related medical judgment.
8. Assure subjects receive sufficient medical care together with the head of the study site for any study-related clinically-relevant AEs throughout the participation and the subsequent period.
9. For a subject being treated in other institutions or other departments, provide information in writing of study participation and completion/withdrawal of the subject to the institution or the physician in other department upon subject’s approval, which should be documented.
10. Report any occasion requiring expedited reports including SAEs to the head of the study site and the sponsor immediately in writing.
11. Decide necessity for opening the emergency key of the particular subject in emergency situation.
12. Complete the (e)CRFs accurately and completely with the e-signature to be submitted to the sponsor.
13. Check, verify and e-sign (e)CRFs entered by subinvestigators or transcribed from raw data by study collaborators to be submitted to the sponsor.
14. Be consulted for revision of the protocol or the like, when proposed by the sponsor
15. Report study completion to the head of the study site in writing.

Appendix C Diagnostic Criteria for UC (Revised on January 17, 2013)

1. Definition

A non-specific diffuse inflammation in the colon, mainly involving the mucosa and often forming erosion or ulcer. The name and overview specified by Council for International Organization of Medical Science (CIOMS) in the WHO are provided below; (1973)

Idiopathic proctocolitis
An idiopathic, non-specific inflammatory disorder involving primarily the mucosa and submucosa of the colon, especially the rectum. It appears mainly in adults under the age of 30, but may affect children and adults over the age of 50. Its aetiology remains unknown, but immunopathological mechanisms and predisposing psychological factors are believed to be involved. It usually produces a bloody diarrhoea and various degrees of systemic involvement, liability to malignant degeneration, if of long duration and affecting the entire colon.

2. Diagnostic Procedures

For a suspected patient of the disease as having chronic (viscous) rectal bleeding or the like, infectious enteritis should be firstly excluded by interview of histories including radiotherapy, antibiotics and traveling abroad, and by microbiological/parasitological examination. Then, the disease-specific intestinal lesion should be confirmed by endoscopy in the rectum and sigmoid colon. At the time, biopsy will be accompanied. Most patient may be diagnosed from the above, however other examinations including the barium enema radiography or the endoscopy in the entire colon may be performed, as needed, to see description, intensity and extent of involvement of the intestinal lesion, and also to exclude other disease.

3. Diagnostic criteria

A patient who meets the following a), 1 item in b) and c) may be definitively diagnosed, when the following diseases are ruled out.

a) Clinical condition: continuous or repetitive (viscous) rectal bleeding or its history

b) [1] Endoscopy: i) shows diffusely-affected, rough or finely-granular mucosa with invisible vascular pattern, which are also friable and bleed easily (contact bleeding) with adhesion of viscous sanguinopurulent-secreted matters adhered, or ii) shows multiple erosion, ulcer or pseudopolyposis.
[2] The barium enema radiography: shows i) diffuse change on the rough or finely-granular mucosal surface, ii) multiple erosion or ulcer, and iii) pseudopolyposis. It also shows disappearance of colonic haustra (lead pipe appearance) or narrowing/shortening of the intestine.

c) Histological biopsy: shows diffuse inflammatory infiltration, crypt abscess and severe decrease of goblet cells in all layers of the mucosa during the active stage. As findings are each non-specific, a comprehensive judgment is required. Misalignment of the gland

(tortuosity or bifurcation) and atrophy remain during the remission stage. The above changes are usually observed from the rectum toward the mouth sequentially

A patient, in whom the examinations in the above b) and c) may not be performed partially or completely, but in whom the disease-specific findings are observed macroscopically or histologically in resective operation or autopsy, may be definitively diagnosed when the following diseases are ruled out.

The diseases that should be ruled out include mainly infectious enteritis such as shigellosis, amebic colitis, salmonella enteritis, campylobacter enteritis, colonic tuberculosis and chlamydial enteritis, and others such as CD, radiation-induced colitis, dug-induced colitis, lymphoid follicle-proliferative disorder, ischemic colitis, and intestinal Behcet's syndrome.

[Note 1] Some rare patients should be noted, who may not be aware of rectal bleeding or who visit immediately after awareness of rectal bleeding (shorter disease duration)

[Note 2] A patient who may not definitively be diagnosed with mild findings should be “suspected” and would be diagnosed later as “definite” at recurrence, etc., showing clear findings.

[Note 3] Indeterminate colitis
A patient, who is difficult to be definitively diagnosed as showing clinical and pathological findings of both CD and UC, may come to show more specific findings of either disease in the course of follow-up.

4. Pathology (Classification, Stages and Severities)

A. Classification by extent of the lesion

Total colitis

Left-sided colitis

Proctitis

Right-sided or segmental colitis

[Note 4] Left-sided colitis is not extending over the splenic flexure.

[Note 5] Proctitis is meeting the above diagnostic criteria, but presenting normal mucosa at the oral-sided rectosigmoid (RS) in the endoscopy.

[Note 6] Right-sided or segmental colitis is so difficult to be definitively differentiated from CD or colonic tuberculosis that it may be diagnosed in the course of follow-up or after acquisition of results of resection or autopsy.

[Note 7] Diffuse gastroduodenitis may emerge.

B. Classification of the disease stages

Active stage

Remission stage

[Note 8] The active stage is the condition where a patient may complain of rectal bleeding, the endoscopy may show an invisible vascular pattern, bleed easily, or have erosion, ulcer or the like.

[Note 9] The remission stage is the condition where rectal bleeding may be resolved, and findings seen during the active stage may not be observed in the endoscopy with visible vascular pattern.

C. Classification by clinical severity

Mild

Moderate

Severe

The diagnostic criteria is provided below;

	Severe	Moderate	Mild
1) Stool frequency	≥6	Intermediate between sever and mild	≤4
2) Overt bloody stool	(+ + +)		(+) to (-)
3) Fever	≥37.5°C		(-)
4) Tachycardia	≥90/min.		(-)
5) Anemia	≤Hb 10 g/dL		(-)
6) Erythrocyte sedimentation	≥30 mm/h		Normal

[Note 10] The (-) in the above 3), 4) and 5) in the Mild represents conditions without fever of ≥37.5°C, tachycardia of ≥90/min., and anemia of ≤Hb 10 g/dL, respectively.

[Note 11] The Severe should meet the above 1) and 2), and either 3) or 4) (for general condition), and at the same time meet ≥4 items in the 6 items. The Mild should meet all of the above 6 items.

[Note 12] The Moderate should be intermediate between the above Severe and Mild.

[Note 13] The notably severe and serious disease in the Severe should be fulminating and categorized either to the acute fulminating type and the recurrent fulminating type in the course of development. All of the following 5 items should be met as the diagnostic criteria of the fulminating.
(1) Fulfillment of the above severe criteria.
(2) Continuous bloody diarrhea of ≥15/day
(3) Continuous fever of ≥38°C

(4) Leukocytosis of $\geq$10,000/mm^3
(5) Severe abdominal pain

D. Classification by findings on endoscopy during the active stage

Mild

Moderate

Severe

The diagnostic criteria is provided in the following table;

Inflammation	Findings on Endoscopy
Mild	Invisible vascular pattern<br>Finely-granular mucosa<br>Redness, aphtha, small yellowish spots
Moderate	Rough, erosive or small ulcerative mucosa<br>Bleed easily (contact bleeding)<br>Adhesion of viscous sanguinopurulent-secreted matters<br>Other active inflammatory findings
Severe	Extensive ulcer<br>Notable spontaneous hemorrhage

[Note 14] The most severe lesion should be diagnosed in the entire endoscopically examined lesion. The endoscopy may be conducted in a short time without pretreatment and not necessarily examined over the entire colon.

E. Classification by clinical course

Relapse-remitting type

Chronic continuous type

Acute fulminating type

First attack type

[Note 15] The chronic continuous type is on the active stage for 6 month or more after the first attack.

[Note 16] The acute fulminating type occurs with extremely fulminating symptoms, and may often be associated with complications including toxic megacolon, perforation or sepsis.

[Note 17] The first attack type occurs only once, however, may recur in the future and mostly becomes the relapse-remitting type.

F. Particular classification by macroscopic findings

Pseudopolyposis type

Atrophic colitis type

G. Definition of intractable UC based on treatment response

1. A disease meeting either of the following despite a rigorous corticosteroid therapy.
   [1] Corticosteroid resistance (no response after treatment of 1 to 1.5 mg/kg/day of prednisolone for 1 to 2 weeks)
   [2] Corticosteroid dependence (recurrence during the course of dose reduction of the corticosteroid)
2. A disease frequently recurs or appears to be the chronic continuous type despite rigorous medical treatment other than corticosteroid.

H. Diagnostic criteria for pouchitis

I. Overview

Pouchitis is a non-specific inflammation occurring in the ileoanal pouch in patients who underwent innate anus-preserving total (subtotal) colectomy. Though etiology remains unknown, the disease may possibly be associated with pathogenesis of UC, as frequently occurs after UC-related surgeries and infrequently occurs after surgeries of familial adenomatous polyposis.

II. Diagnoses of pouchitis

1. Items

a) Clinical symptoms
1) Increase of stool frequency, 2) Rectal bleeding, 3) Defecation urgency or abdominal pain4) Fever (≥37.8°C)

b) Findings on Endoscopy
Mild: edema, granular mucosa, invisible vascular pattern, mild redness
Moderate: aphtha, erosion, small ulcer#, bleed easily, viscous mucus
Severe: extensive ulcer, multiple ulcer#, diffuse redness, spontaneous hemorrhage

#: Staple line ulcer should be entered separately from other findings on endoscopy of pouchitis, unless other findings were observed.

2. Diagnostic criteria

Pouchitis is diagnosed when moderate or greater findings on endoscopy are found with at least one clinical symptom, or when severe findings on endoscopy were found with or without clinical symptoms. The diseases that should be ruled out include infectious enteritis (bacterial enteritis including salmonella enteritis, campylobacter enteritis and intestinal tuberculosis, viral enteritis including cytomegalovirus enteritis, and parasitosis), anastomotic leakage, pelvic infection, postoperative anal dysfunction and CD.

Appendix D PML Checklists

- The PML checklists will be utilized in visits specified in the protocol, or for a subject complaining such symptoms.
- The purpose of the PML checklists is to identify any obvious neurological changes in a subject since the last evaluation.
- When any subjective symptoms were identified, the corresponding tests to see objective findings should be performed. Any symptoms/findings found on the checklists should be recorded as AEs.
- Subjects should be instructed to visit a neurologist, when any obvious neurological changes were observed. Examinations including MRI using a contrast agent should be performed for collection of information supporting the neurological change, if needed.
- Any positive subjective symptoms or positive objective findings should be provided to the sponsor immediately.

Algorithm for Subject Evaluation

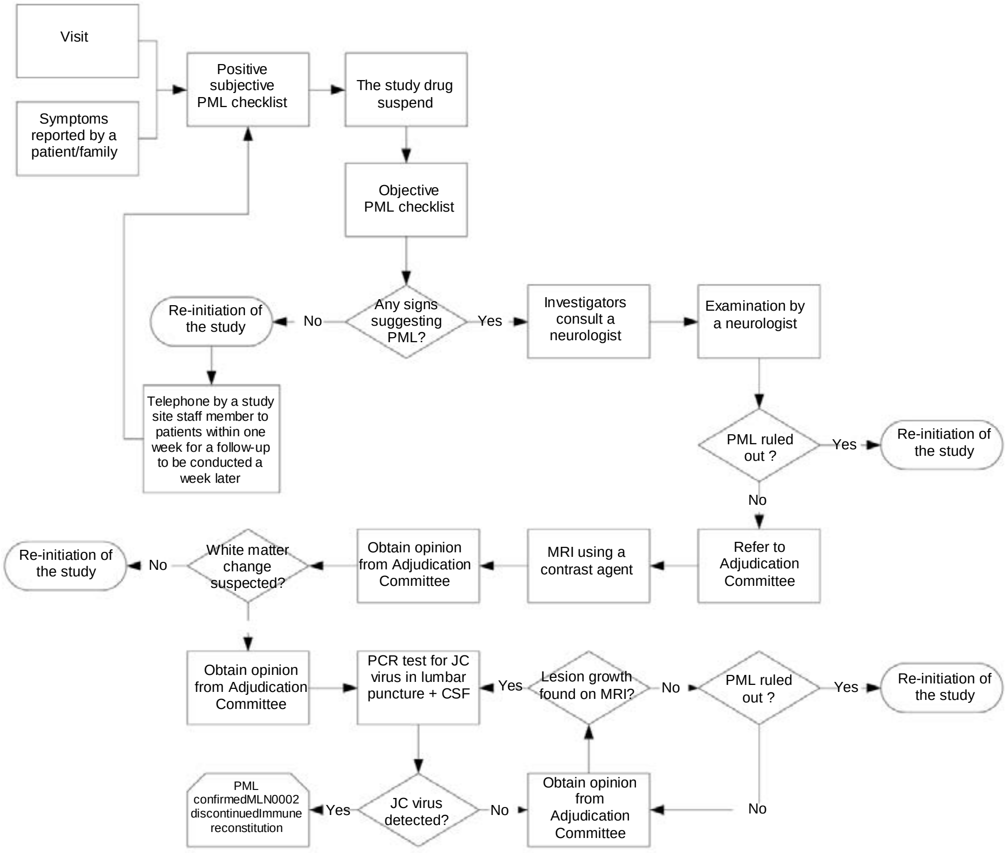

Subjective PML checklist

Date: month/ date/ year	
Subject Identification Code:	

Symptoms	Have you had a significantly unusual experience for the following?		If "yes," describe symptoms specifically	Objective test to be applied: results should be recorded in the Objective PML checklist
	Yes	No		
1. Have you experienced continuous loss of vision, multiple vision or reading disorder, recently?				Visual field and eye movement should be tested.
2. Have you felt any continuous difficulty in talking, or continuous inability of your speech to be understood by others, recently?				Natural observation should be performed for patient's speech for dysarthria or aphasia. The patient should be requested to answer names of some objects and to repeat some phrases with several words.
3. Have you felt any continuous muscle weakness in your arms or legs, recently?				Pronator deflection test (Barré procedure) and/or a test to see ability to keep the arm being rotated Ability to hop on one foot; to tap with the foot and finger. A symmetry test in the muscle.
4. Have you frequently banged into something, or felt any difficulty in writing characters, recently?				The patient should be required to show spontaneous writing and be observed in the finger-to-nose test and heel-to-shin test and while walking (in a straight line by putting the heel of one foot in front of the toe of the other foot alternately).

Date: month/ date/ year	
Subject Identification Code:	

Symptoms	Have you had a significantly unusual experience for the following?		If "yes," describe symptoms specifically	Objective test to be applied: results should be recorded in the Objective PML checklist
	Yes	No		
5. Have you often experienced an inability to understand wording someone has said recently?				Ability to follow a series of instruction (stick out the tongue and touch the left ear with the left fingers while keeping the eyes closed).
6. Do you have any continuous problems in your memory or thinking?				The patient should be reminded of the 3 objects after 1 minute or longer distraction ; ability to follow instructions
7. Have you experienced continuous hypesthesia or other loss of sensation?				Sensation should be tested by neighboring needle stimulation

Conducted by (signature or name/seal)

_______ seal ___ month/ date/ year

Objective PML checklist

For a patient with subjective symptoms.

Please perform the tests for objective findings corresponding to the observed subjective symptoms.

Date: month/ date/ year
Subject Identification Code:

Positive subjective symptoms	Objective tests applied	Test results		Brief description of the observed abnormality
		Normal	Abnormal	
1.<br>Difficulty in vision and reading	Visual field and eye movement should be tested.			
2.<br>Difficulty in speech	Spontaneous observation should be performed for patient's speech for dysarthria or aphasia. The patient should be requested to answer names of some objects and to repeat some phrases with several words.			
3.<br>Muscle weakness in the upper or lower limb	Pronator deflection test (Barré procedure) and/or a test to see ability to keep the arm being rotated Ability to hop on one foot; to tap with the foot and finger. A symmetry test in the muscle.			
4.<br>Bang into something or difficulty in writing	The patient should be required to show spontaneous writing and observed in the finger-to-nose test and heel-to-shin test and while walking (in a straight line by putting the heel of one foot in front of the toe of the other foot alternately).			

Date: month/ date/ year	
Subject Identification Code:	

Positive subjective symptoms	Objective tests applied	Test results		Brief description of the observed abnormality
		Normal	Abnormal	
5. Difficulty in understanding others	Ability to follow a series of instruction (stick out the tongue and touch the left ear with the left fingers while keeping the eyes closed).			
6. Problems in memory or thinking	The patient should be reminded of the 3 objects after 1 minute or longer distraction ; ability to follow instructions			
7. Problems of hypesthesia	Sensation should be tested by neighboring needle stimulation			

- The patient whose reported symptoms were confirmed in the Objective PML checklist should be instructed to visit a neurologist. The patient should be followed a week after the Objective PML checklist for confirmation of re-manifestation of the reported subjective symptoms.
- Any positive findings in the Objective PML checklist must be provided to the sponsor.

Conducted by (signature or name/seal)

_______ seal __ month/ date/ year

Appendix E Follow-Up Survey Form

Subject Identification Code:________________________
Number of months after the last dose of the study drug:
6 mo.☐ 12 mo.☐ 18 mo.☐ 24 mo.☐
Date: month/ date/ year

1. Have you been diagnosed with colon dysplasia (one of the precancerous lesions) or colon cancer, lymphoma, or other cancers since the last contact/visit in the study?<br>**If "yes," please answer to the following questions:.**<br>• Colon dysplasia Yes/No Date of the diagnosis: month/ date/ year<br>• Colonic cancer Yes/No Date of the diagnosis: month/ date/ year<br>• Lymphoma Yes/No Date of the diagnosis: month/ date/ year<br>• Other: (Specify) Yes/No Date of the diagnosis: month/ date/ year	Yes<br>☐	No<br>☐
2. Have you been diagnosed as having progressive multifocal leukoencephalopathy (also known as PML) since the last contact/visit in the study?<br>**If "yes," please answer to the following question:.**<br>• Date of the diagnosis: month/ date/ year	Yes<br>☐	No<br>☐
3. Have you undergone an enterectomy since the last contact/visit in the study?<br>**If "yes," please answer to the following questions:.**<br>• Colectomy Yes/No Date of the diagnosis: month/ date/ year<br>• Small bowel resection Yes/No Date of the diagnosis: month/ date/ year	Yes<br>☐	No<br>☐
The following 3 questions will be completed only once at 6 months after the last dose of the study drug.		
4. Have you been diagnosed as having an infection requiring hospitalization since the last study visit?<br>**If "yes," please answer to the following questions:.**<br>• Specify the reason for the hospitalization/diagnosis<br>Reason:______________________________<br>• Date of the diagnosis: month/ date/ year	Yes<br>☐	No<br>☐
5. For female patients: Have you become pregnant since the last study visit?<br>**If "yes," please answer to the following questions:.**<br>• The PREGNANCY REPORTING FORM should be completed<br>• Specify the estimated delivery date: month/ date/ year	Yes<br>☐<br>Not applicable ☐	No<br>☐
6. For male patients: Has your female partner become pregnant since the last study visit?<br>**If "yes," please answer to the following questions:.**<br>• The PREGNANCY REPORTING FORM should be completed<br>• Specify the estimated delivery date: month/ date/ year	Yes<br>☐<br>Not applicable ☐	No<br>☐

Investigator (signature or name/seal)

____________ seal

month/ date/ year
